# Nomenclatural changes in *Coleus* and *Plectranthus* (Lamiaceae): a tale of more than two genera

**DOI:** 10.3897/phytokeys.129.34988

**Published:** 2019-08-23

**Authors:** Alan J. Paton, Montfort Mwanyambo, Rafaël H.A. Govaerts, Kokkaraniyil Smitha, Somran Suddee, Peter B. Phillipson, Trevor C. Wilson, Paul I. Forster, Alastair Culham

**Affiliations:** 1 Science Directorate, Royal Botanic Gardens Kew, Richmond, Surrey,TW9 3AB, UK Royal Botanic Gardens Kew Richmond United Kingdom; 2 National Herbarium and Botanic Gardens of Malawi, Zomba, Malawi National Herbarium and Botanic Gardens of Malawi Zomba Malawi; 3 Department of Botany, University of Calicut, Kerala- 673 635, India University of Calicut Kerala India; 4 The Forest Herbarium (BKF), Department of National Parks, Wildlife and Plant Conservation, Chatuchack, Bangkok 10900,Thailand The Forest Herbarium Bangkok Thailand; 5 Missouri Botanical Garden, P.O. Box 299, St. Louis, MO 63166, U.S.A. and Institut de Systématique, Evolution et Biodiversité (UMR 7205 –CNRS MNHN UPMC EPHE), Muséum national d’Histoire naturelle, Sorbonne Universités, C.P. 39, rue Cuvier 57, 75231 Paris CEDEX 05, France Sorbonne Universités Paris France; 6 National Herbarium of New South Wales, Royal Botanic Gardens & Domain Trust, Mrs Macquaries Road, Sydney, NSW 2000, Australia and School of Life and Environmental Sciences, Science Road, University of Sydney, NSW 2006, Australia University of Sydney Sydney Australia; 7 Queensland Herbarium, Department of Environment & Science, Brisbane Botanic Gardens, Mount Coot-tha Road, Toowong, Qld 4066, Australia Queensland Herbarium Toowong Australia; 8 School of Biological Sciences, University of Reading, Whiteknights, Reading, UK University of Reading Reading United Kingdom

**Keywords:** *
Anisochilus
*, *
Equilabium
*, generic delimitation, nomenclature, Ocimeae, Plectranthinae, *
Pycnostachys
*, *
Solenostemon
*

## Abstract

A synopsis of the genera *Coleus* Lour, *Equilabium* A.J.Paton, Mwany. & Culham and *Plectranthus* L’Hér. (Lamiaceae, Tribe Ocimeae, Subtribe Plecranthinae) is presented. Generic delimitation follows a recently published molecular phylogeny which identified *Coleus* as the sister of the remaining genera of Subtribe Plectranthinae; *Plectranthus* as sister to *Tetradenia* Benth. and *Thorncroftia* N.E.Br., and a separate phylogenetically distinct genus *Equilabium* comprising species previously placed in *Plectranthus*. In this treatment, 294 species of *Coleus*, 42 of *Equilabium*, and 72 of *Plectranthus* are recognized. All but one of the combinations in *Equilabium* are new as only the genus and type species have been previously published. Two-hundred and twelve names are changed to combinations in *Coleus* from *Plectranthus*, *Pycnostachys* Hook. and *Anisochilus* Benth.

## Introduction

**Figure 1.**
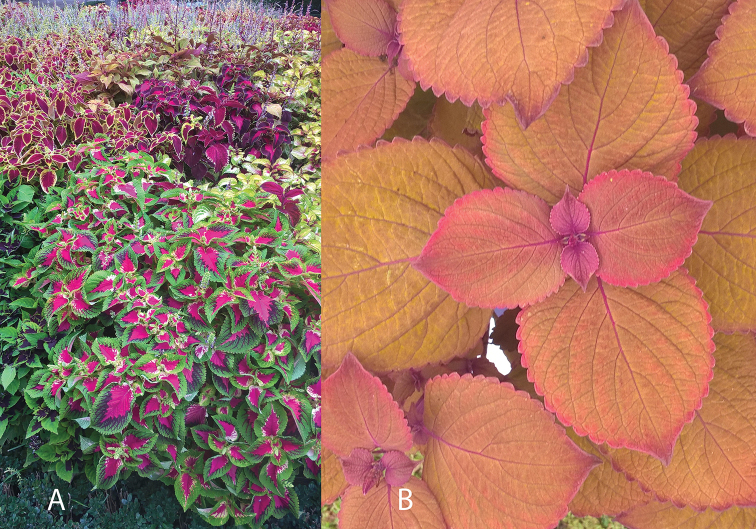
**A** Display of cultivated *Coleus scutellarioides* (L.) Benth. in the Real Jardin Botánico, Madrid. **B** Close up of the cultivar ‘Campfire’, winner of the Royal Horticultural Society’s peoples choice award at Wisley 2017 (Photo: A.Paton).

*Plectranthus* L’Hér. is a widely used horticultural and medicinal plant genus (van Jaarsveld 2007, Lukhoba et al. 2006, Rice et al. 2011). It belongs in tribe Ocimeae, subtribe Plectranthinae, an Old World tropical group of around 450 species. Current taxonomic treatments generally follow Harley et al. (2004) by including the formerly recognized *Coleus* Lour. and *Solenostemon* Thonn. within *Plectranthus*. However, within horticultural literature, *Solenostemon* is still frequently recognized (e.g. RHS (2017), Shepherd and Maybry (2016)). The commonly cultivated plant shown in Fig. 1 is thus variously known as *Plectranthus scutellarioides* (L.) R.Br or *Solenostemon scutellarioides* (L.) Codd., but “coleus” is frequently used as a common name for this species, increasing the confusion.

**Figure 2.**
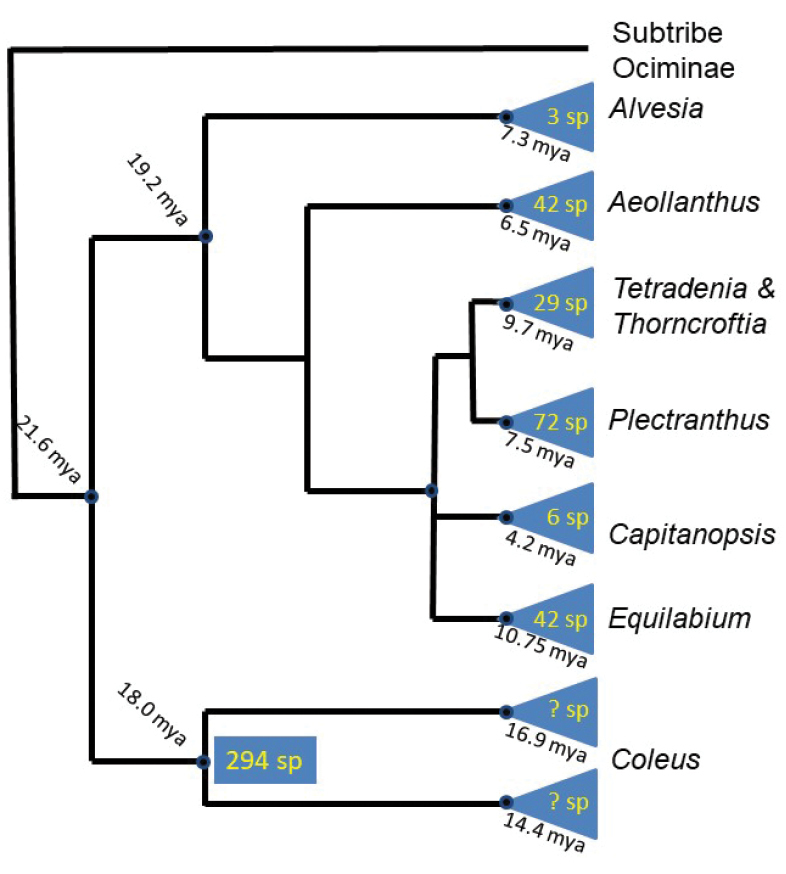
Summarised phylogeny of the Plectranthinae showing relationships of recognised genera following Paton et al. (2018): the numbers in triangles represent species numbers though the numbers of species in the two subclades of *Coleus* cannot be estimated (see discussion); dates of branching are given in black.

Recent phylogenetic work has shown that the circumscription of *Plectranthus* needs to be viewed in the context of related genera in Tribe Ocimeae, subtribe Plectranthinae. Paton et al. (2018) demonstrate that a clade containing the type of *Coleus* (*C. amboinicus* Lour. ≡ *P. amboinicus* (Lour.) Spreng.) is sister to the other genera of the Plectranthinae, and the type species of *Plectranthus*, *P. fruticosus* L’Hér., belongs in a clade which is sister to *Thorncroftia* N.E.Br and *Tetradenia* Benth. A further clade containing species that were previously placed in *Plectranthus* was recognized by Paton et al. (2018) as *Equilabium* Mwany., A.J.Paton & Culham (Fig. 2).

The aim of this paper is to provide the necessary nomenclatural changes consistent with the recognition of *Plectranthus*, *Coleus* and *Equilabium* as distinct genera following phylogenetic results provided by Paton et al. (2018).

## Taxonomic history

*Plectranthus* was described by L’Héritier in 1788 (L’Héritier 1788) and *Coleus* by Loureiro in 1790 (de Loureiro 1790). In previous global reviews of the Lamiaceae, e.g. Bentham (1832, 1848, 1876) and Briquet (1897), *Coleus* was treated as distinct from *Plectranthus* by having the 4 stamens fused together rather than free to their base. Morton (1962) recognized that the character of the fused stamens was more plastic than previously thought and merged *Coleus* into *Plectranthus*, as first suggested by Brown (1810). However, Morton also placed weight on calyx characteristics leading him to recognize several genera based on calyx morphology including *Solenostemon*, *Isodictyophorus* A.Chev, *Leocus* A.Chev., *Englerastrum* Briq. and *Neohyptis* J.K.Morton. The merging of *Coleus* into *Plectranthus* has been followed by most authors since Morton’s account, for example, Keng (1978), covering Malesian species, Blake (1971, Australia), Codd (1985, southern Africa), Hedge et al. (1998, Madagascar), Suddee et al. (2004, Indochina), Forster (2011, Australia) and Paton et al. (2009, 2013, eastern and southern tropical Africa). Furthermore, although Codd (1985) and Morton (2006) also maintained *Solenostemon*, Keng (1978), Hedge et al. (1998) and subsequent authors recognised *Solenostemon* as synonymous with *Plectranthus*.

Despite the agreed taxonomic status by the above authors, Cramer (1978) maintained that *Coleus* is a separate genus from *Plectranthus*, as did Wu and Huang (1977)and Li and Hedge (1994) for Chinese species. Cramer’s decisions were in part based upon Sri Lankan *Coleus* that have imbricate bracts (“floral leaves”) and a decurrent upper calyx lip in addition to fused stamens. Cramer also suggested *Plectranthus* could have a 3-lobed upper calyx lip, but that is misleading since he was considering species of *Isodon* (Schrad. ex Benth.) Spach as part of *Plectranthus*: *Isodon* is now recognized in a separate subtribe and is very distinct from the Plectranthinae (Ryding 1993, Harley et al. 2004, Zhong et al. 2010).

A common theme in the treatments listed above is that, since Briquet (1897), all have been based on regional accounts of relatively small numbers of species. Morton (1962) considered 28 species of the *Coleus*/*Plectranthus* complex and Cramer (1978), 11 species. Morton recognized the problems in this narrow regional approach noting, “*hence the genus Plectranthus must for the moment be a repository for Coleus or Plectranthus-like species which cannot satisfactorily be placed in other genera*” and Cramer considered his account to be “ *by no means conclusive, least of all authoritative”.* Paton in Harley et al. (2004) thus took a broader approach, considering characters used to delimit allied genera in the context of the Plectranthinae as a whole. In Paton’s treatment, *Coleus* and the small segregate genera recognized by Morton were merged into *Plectranthus*, making *Plectranthuss.l.* morphologically recognizable from other genera and an easily communicable concept. However, this was a conservative, morphologically based account and did not tackle how best to circumscribe taxa within the large *Plectranthuss.l.*

Paton et al. (2004) provided a preliminary test of the broad concept of *Plectranthus* by presenting a phylogeny of tribe Ocimeae, demonstrating that the Plectranthinae was a monophyletic group which could be generally diagnosed by having stamens adnate and contiguous at the base of the anterior corolla lip. The presence of fused stamens, the original defining character of *Coleus*, was shown to be homoplasious by mapping it onto this phylogeny. However, the study identified two main clades within the Plectranthinae: one containing the type of *Coleus* and members of *Solenostemon*, *Leocus* A.Chev., *Pycnostachys* Hook. and *Anisochilus* Benth.; and the other containing the type species of *Plectranthus* and members of *Alvesia* Welw., *Aeollanthus* C. Mart. ex Spreng, *Capitanopsis* S.Moore, *Tetradenia* and *Thorncroftia*.

**Figure 3.**
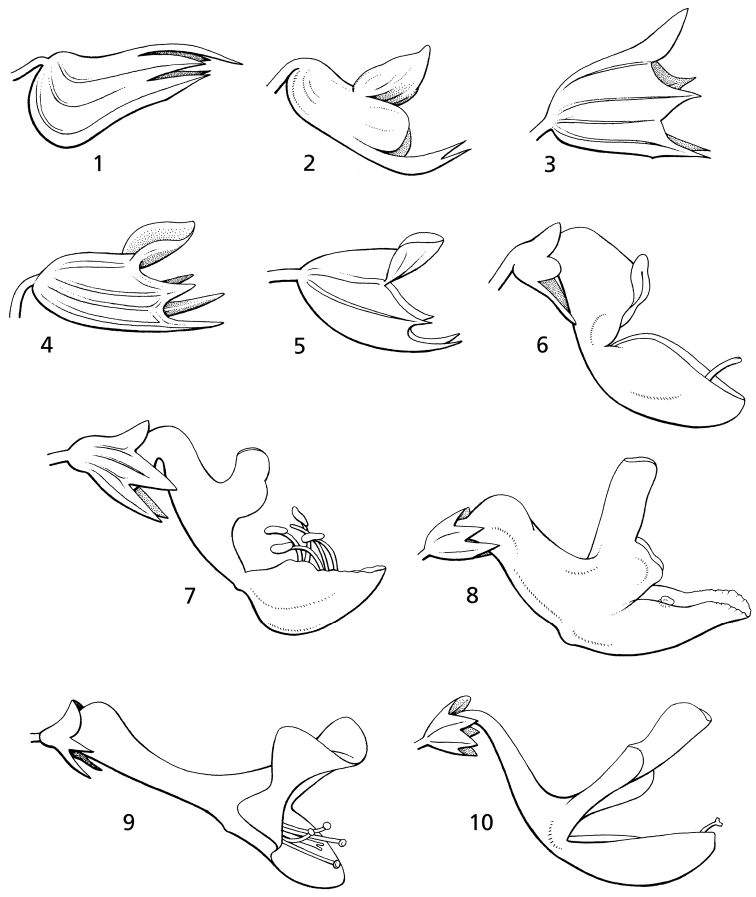
Calyx and corolla morphology. Calyces in *Coleus* with pedicel attached excentrically above midline of calyx: **1***C. betonicifolius***2***C. platyphyllus* showing the rounded lateral calyx lobe in some species of the formally recognized *Solenostemon*. Calyx in *Equilabium* with pedicel attached ± at centre of calyx base with the lateral lobe held midway between the anterior and posterior lobes: **3***E. megafolium***4***E. masukensis*. Calyces in *Plectranthus* with pedicel attached ± at centre of calyx base with the lateral lobe closer to the anterior than the posterior lobes: **5***P. alboviolaceus*. Corolla shape in *Coleus* showing unequal anterior and posterior corolla lips: **6***C. schizophyllus***7***C. hockii*. Corolla shape of *Equilabium* showing equal anterior and posterior corolla lips: **8***P. parvus***10***P. laxiflorus*. Corolla shape of *Plectranthus* showing equal anterior and posterior corolla lips and gibbous-based corolla: **9***P. strangulatus*;. Drawn by Emmanuel Papadopoulos. Reproduced from Flora of Tropical East Africa (Paton et al. 2009).

The phylogenetic study by Paton et al. (2018) increased the sampling of the Plectranthinae and added another plastid marker to increase tree resolution, and similarly found that *Plectranthuss. l.* was polyphyletic (see summary of analysis in Fig. 2). As a result of this work, Paton et al. (2018) suggested recognition of *Coleus* for the clade containing the *Coleus* type, *Plectranthuss.s.* for the clade sister to *Thorncroftia* and *Tetradenia*, and a new genus, *Equilabium*, for the clade formerly containing *Plectranthus* species mainly from tropical Africa, but with one in India. Paton et al. (2018) pointed out that these clades were morphologically diagnosable by characters that had been used to define informal morphological groups by Paton et al. (2009, 2013). These characters are the position of attachment of the pedicel to the calyx, the relative lengths of the corolla lobes and the shape of the corolla tube (Fig. 3). The phylogenetic results indicate that three genera recognized by Harley et al. (2004), (*Pycnostachys*, *Anisochilus* and *Leocus*) are nested within *Coleus*, and this close relationship is corroborated by species of intermediate morphology between *Coleus* and the former taxa *Pycnostachys* (Paton et al. 2009, 2013), *Anisochilus* (Suddee et al. 2014), and *Leocus* (Pollard and Paton 2009). Paton et al. (2018) also showed that *Solenostemon* is not monophyletic, and that recognition of *Solenostemon* would render *Coleus* paraphyletic by its exclusion, therefore we argue that *Solenostemon* should not be recognised.

## Methods

The phylogenetic study presented by Paton et al. (2018) was based on a representative sample of species of the Plectranthinae. Recent floristic accounts and the *World Checklist Of Selected Plant Families* were consulted (Govaerts et al. 2019, Paton et al. 2009, 2013, Suddee et al. 2004, Codd 1985, Hedge et al. 1998, Forster 1992, 1994, 2011 and references within). IPNI (www.ipni.org) was consulted to trace any recently described species of *Coleus* or *Plectranthus* and the protologues of these were examined. A survey of herbarium collections (AAU, BK, BKF, BM, BOL; BR, C, CALI, COI, EA, ETH, FT, G, HBG, K, L, LE, LMA, LISC, LMU, MEL, MH, MO, NHT, P, PRE, RNG, S, STU, TCD, UPS, W, WAG; Abbreviations following Thiers (2017)), was undertaken also using JSTOR Global Plants (https://plants.jstor.org/) to access type specimens not otherwise seen. Cited types have been seen unless otherwise stated. The attribution of species to the genera *Coleus*, *Plectranthus* and *Equilabium* was guided by the characters given in Paton et al. (2018) and in the key at the beginning of the taxonomic account below.

## Results and discussion

The changes in generic delimitation proposed by Paton et al. (2018) have a large impact on nomenclature. An alternative nomenclatural treatment would be to consider the monophyletic Plectranthinae to be components of a single genus, *Plectranthus*. Most recent taxonomic papers have included *Coleus* within *Plectranthus*. However, taking 2005 as a starting point, following publication of Harley et al. (2004) as the most comprehensive baseline taxonomy for the Lamiaceae, 15,700 papers cite *Coleus* in the title, but only 9320 cite *Plectranthus* (source Google Scholar). This suggests that *Coleus* is still a broadly used concept. *Tetradenia* with mainly dioecious, ± actinomorphic flowers is part of the sister group of the *Plectranthus* in the strict sense. Thus there are no easily observed morphological apomorphies that could be used to easily diagnose an enlarged *Plectranthus*; whereas the generic delimitation proposed in Paton et al. (2018), results in monophyletic genera which are relatively easy to diagnose morphologically.

In this treatment, 294 species of *Coleus*, 42 of *Equilabium*, and 72 *Plectranthus* are recognized. All the combinations in *Equilabium* are new as only the genus and type species *E. laxiflorum* have been previously published (Paton et al. 2018). 212 names need to be changed to combinations in *Coleus* from combinations in *Plectranthus*, *Pycnostachys* and *Anisochilus*. However, 82 of these combinations in *Coleus* already exist, reflecting previous confusion. No new names are necessary for species in *Plectranthus*.

Of the characters cited by Cramer (1978) as supporting the delimitation of *Coleus* and *Plectranthus*, the presence of a decurrent calyx tube is variable and found in *Coleus*, *Plectranthus* and *Equilabium*. Imbricate bracts do only occur in *Coleus*, but this character is only obvious in some species. Generally the species are very easy to attribute to *Coleus*, *Plectranthus* or *Equilabium* using the pedicel attachment, corolla lobe length and tube shape characters described in the key below. The presence of persistent bracts and slightly flattened nutlets also supports the division of *Coleus* (often cauducus bracts and slightly flattened nutlets), from *Plectranthus* and *Equilabium* (usually persistent bracts and ovoid nutlets), though those characters have more exceptions than those characters used in the key. Both *Equilabium* and *Plectranthus* have rarely more than 3-flowered cymes, whereas *Coleus* can have one to very many flowers per cyme. Although species can be misplaced in some instances due to atypical corolla structure, the asymetrical pedicel attachment at the base of the calyx gives the most clear indication of the species’ placement in *Coleus*. Generic identification can be harder with small-flowered *Coleus* species that show asymetrical pedicel attachment but have more or less equal corolla lips; however, the number of these cases is small. In *Equilabium* the upper lip can be slightly shorter than the lower lip in some cases but at the extreme it is about 0.8 of the length of the lower lip, whereas in *Coleus* the upper lip is usually much shorter. The southern African *Coleus zombensis* (Baker) A.J.Paton, *C. gibbosus* A.J.Paton (*Plectranthus rehmannii* Gürke) and *Coleus sessilifolius* (A.J.Paton) A.J.Paton can also provide confusion as the corolla is basally gibbous, similar to that of many species of *Plectranthus*; however, they have a *Coleus*-like calyx and the corolla tube is sigmoid rather than straight as in true *Plectranthus*.

We present the list of species in alphabetical order, rather than according to the previously proposed infrageneric classification by Codd (1975), which is unworkable. Although the smaller infrageneric groups he recognized in the infrageneric classification of *Plectranthuss.l.* of Southern Africa are natural groups of related Southern African species, Codd’s classification does not cover the extent of character variation and combinations seen outside the Flora of Southern Africa area, making it difficult to apply globally. As pointed out by Paton et al. (2018), groups previously thought to be easily delimited by morphological characters can be paraphyletic. For example, subgenus Calceolanthus Codd was delimited by Codd by having a dense ring of hairs in the calyx throat, but the phylogeny presented by Paton et al. (2018) suggests that the clade containing these species also contains species which lack this character. Larger infrageneric groupings recognized by Codd contain a mixture of lineages, for example, Codd’s section Plectranthus while containing mostly species placed in *Plectranthus* here, also includes *P. dolichopodus* Briq. (here placed in *Coleus*) and *P. laxiflorus* Benth. and *P. petiolaris* Benth. (here both are placed in *Equilabium*).

Further study is required to provide a more robust formal infrageneric classification of *Coleus* and *Plectranthus*, although within *Coleus* there seems to be a continuity of form making further morphological diagnoses of clades difficult. There are two well supported clades within *Coleus* (clades A and B *sensu*Paton et al. (2018)), but no clear morphological apomorphies to diagnose these clades have been identified. Further phylogenetic work is underway by the authors: fifty species are now described for Australia and a current phylogenetic analysis focusing on these species is being prepared from a comprehensively sampled data set (T.C. Wilson, unpubl.); and an analysis of species in India is also being prepared (K. Smitha, unpubl.).

### Taxonomic account

A key to the genera of the Plectranthinae is provided and followed by treaments of the three genera now containing species of *Plectranthus s.l. sensu*Harley et al. (2004): *Coleus*, *Equilabium* and *Plectranthus*. New combinations of *Capitanopsis* and the generic protologue of *Equilabium* can be found in Paton et al. (2018). No changes to other genera of the Plectranthinae are proposed. The generic descriptions are followed by a listing of the species in alphabetical order with details of synonymy, types and distribution. The account ends with a list of unplaced names, followed by a list of excluded names now placed in other genera. Readers should also consult the online treatment in the World Checklist of Selected Plant Families (Govaerts et al. 2019, www.kew.org/wcsp) as this is continually updated and includes TDWG level 3 distributions and Raunkiaer life form descriptors. A short description of the Plectranthinae and key to the genera recognized is provided below.

### Subtribe Plectranthinae Endlicher, Gen. Pl.: 609. 1838.

Herbs or shrubs. Inflorescence an indeterminate thyrse with cymes sessile or pedunculate, ebracteolate, rarely with minute persistent or deciduous bracteoles; bracts persistent or deciduous, rarely coloured. Calyx usually with a single-lobed posterior lip and a 4-lobed anterior lip, sometimes actinomorphic and regularly 5-lobed. Corolla strongly 2-lipped, rarely actinmorphic or subactinomorphic (*Tetradenia*), anterior lip concave to cucullate, more rarely flat. Stamens declinate, rarely spreading (*Tetradenia*), exserted, anterior and posterior attached adjacently at corolla throat (separated in *Tetradenia*). Disc usually 4-lobed, lobes alternating with nutlets. Nutlets with small inconspicuous areole.

### Key to the genera of the Plectranthinae

**Table:** 

1	Corolla actinomorphic, or almost so, stamens spreading not declinate	*** Tetradenia ***
–	Corolla strongly zygomorphic, stamens declinate	**2**
2	Lateral lobes of the corolla deflexed towards the anterior lobe	*** Thorncroftia ***
–	Lateral lobes of the corolla ascending towards the median lobes of the posterior lip or horizontal and held between the anterior lobe and median lobes of the posterior lip	**3**
3	Posterior lip of corolla truncate, lateral lobes usually horizontal, short, held between the anterior lobe and median lobes of the posterior lip	*** Capitanopsis ***
–	Posterior lip of corolla not truncate, diverging from anterior lip of the corolla, with lateral lobes usually ascending and close to median lobes of posterior lip	**4**
4	Calyx basally circumscissile; tube distally dorsiventrally flattened	*** Aeollanthus ***
–	Calyx very rarely circumscissile; tube not dorsiventrally flattened	**5**
5	Calyx 3-lobed (1 upper, 2 lower), expanded and membranous in fruit	*** Alvesia ***
–	Calyx 5-lobed (1 upper, 4 lower, or actinomorphic), rarely expanded and membranous in fruit	**6**
6	Calyx with pedicel attached asymmetrically to the base of the calyx tube, opposite the posterior lip; corolla with upper (posterior) lip shorter than lower (anterior), rarely subequally lobed (Fig. 3)	*** Coleus ***
–	Calyx with pedicel attached symmetrically to the base of the calyx tube, not opposite the upper lip, corolla lobes more or less equal in length (Fig. 3)	**7**
7	Calyx throat truncate with lateral lobes of the calyx held midway between the uppermost and lowermost lobes; corolla tube usually sigmoid, parallel-sided at base, dilating towards throat (Fig. 3)	*** Equilabium ***
–	Calyx throat oblique with lateral lobes of the calyx held closer to the lowermost pair of lobes than the upper lobe; corolla tube straight or curved downwards, usually gibbous, saccate or shortly spurred at base (Fig. 3)	*** Plectranthus ***

## Synopsis of genera and species of *Coleus*, *Equilabium* and *Plectranthus*

### Genus *Coleus*

#### Coleus

Taxon classificationPlantaeLamialesLamiaceae

Lour., Fl. Cochinch.: 372. 1790

14400339EBB05DDDB662E2861BE2D048

Figs 4 , 5

Pycnostachys Hook., Exot. Fl. 3: t. 202. 1826. synon. nov. Type: P. coerulea W.J.Hooker (here recognized under Coleus stenostachys (Baker) A.J.Paton, non Coleus coeruleus Gürke).
Solenostemon Thonn. in C.F.Schumacher, Beskr. Guin. Pl.: 271. 1827. Type: Solenostemon ocymoides Schumach. & Thonn (here recognized under Coleus monostachyus (P. Beauv.) A.J.Paton).
Anisochilus Wall. ex Benth., Edwards’s Bot. Reg. 15: t. 1300. 1830. synon. nov. Type: Anisochilus carnosus (L.f.) Wall. ex Benth. (here recognized under Coleus strobilifer (Roxb.) A.J.Paton, non Coleus carnosus Hassk.).
Mitsa Chapel. ex Benth. nom. inval, Labiat. Gen. Spec.: 52. 1832.
Echinostachys E.Mey., Comm. Pl. Afr. Austr.: 243. 1838. synon. nov. Type: Echinostachys reticulata E.Mey. (here recognized under Coleus kirkii (Baker) A.J.Paton, non Coleus reticulatus A.Chev.).
Saccostoma Wall. ex Voigt, Hort. Suburb. Calcutt.: 449. 1845. Type: S. urticifolius Voigt. (See unplaced names).
Majana Rumph. ex Kuntze, nom. superfl. Revis. Gen. Pl. 2: 523. 1891.
Englerastrum Briq., Bot. Jahrb. Syst. 19: 178. 1894. Type: E. schweinfurthii Briq. (here recognized under Coleus rhodesianum (N.E.Br.) A.J.Paton).
Neomuellera Briq., Bot. Jahrb. Syst. 19: 180. 1894. Type: N. welwitschii Briquet (here recognized under Coleus fredericii G.Taylor).
Capitanya Schweinf. ex Gürke, Bot. Jahrb. Syst. 21: 105. 1895. Type: Capitanya ostostegioides Gürke (here recognized as Coleus otostegioides (Gürke) A.J.Paton).
Burnatastrum Briq. in H.G.A.Engler & K.A.E.Prantl, Nat. Pflanzenfam. 4. 3A: 358. 1897. Type: Burnatastrum lanceolatum (Bojer ex Benth.) Briq. (here recognized as Coleus lanceolatus (Bojer ex Benth.) A.J.Paton).
Stiptanthus Briq. in H.G.A.Engler & K.A.E.Prantl, Nat. Pflanzenfam. 4. 3A: 352. 1897., synon. nov. Type: Stiptanthus polystachyus (Benth.) Briq. (here recognized as Coleus polystachys (Benth.) A.J.Paton).
Symphostemon Hiern, Cat. Afr. Pl. 1: 867. 1900. Type: S. insolitus (C. H. Wright) Hiern (here recognized as Coleus insolitus (C.H.Wright) Robyns & Lebrun).
Leocus A.Chev., J. Bot. (Morot) 22: 125. 1909. Type: L. lyratus Chevalier (here recognized as Coleus lyratus (A.Chev.) Roberty).
Isodictyophorus Briq., Mém. Soc. Bot. France 8: 285. 1917. Type: I. chevalieri Briquet (here recognized as Coleus reticulatus A.Chev.).
Briquetastrum Robyns & Lebrun, Ann. Soc. Sci. Bruxelles, Sér. B 49: 102. 1929. Type: B. africanum (Baker) W. Robyns et Lebrun (here recognized under Coleus engleri (Briq.) A.J.Paton, non C. africanum Benth.).
Holostylon Robyns & Lebrun, Ann. Soc. Sci. Bruxelles, Sér. B 49: 103. 1929. Type designated here: Holostylon gracilipedicellatum Robyns & Lebrun (here recognized as Coleus gracilipedicellatum (Robyns & Lebrun) A.J.Paton).
Ascocarydion G.Taylor, J. Bot. 69 (suppl. 2): 162. 1931. Type: Ascocarydion mirabile (Briq.) G.Taylor (here recognized as Coleus mirabilis Briquet).
Neohyptis J.K.Morton, J. Linn. Soc., Bot. 58: 258. 1962. Type: Neohyptis paniculata (Baker) J.K.Morton (here recognized under Coleus guerkei (Briq.) A.J.Paton, non Coleus paniculatus Benth.).
Rabdosiella Codd, Bothalia 15: 9. 1984. Type: R. calycina (Bentham) Codd (here recognized as Coleus calycinus (Benth.) A.J.Paton).
Coleus sect Solenostemonoides Briq. in H.G.A.Engler & K.A.E.Prantl, Nat. Pflanzenfam. 4(3A): 359. 1897. Type as below.
Calchas P.V.Heath, Calyx 5: 160. 1997. Type: Solenostemon latifolius (Hochst. ex Benth) J.K.Morton, designated by Codd (1975), here recognized under Coleus bojeri Benth.

##### Type species.

*C. amboinicus* Lour.

##### Description.

Annual or perennial herbs or shrubs, sometimes succulent, sometimes with a fleshy or tuberous rootstock. Leaves opposite, sometimes whorled. Inflorescence thyrsoid, strongly condensed to lax, with cymes sessile or pedunculate, sometimes with long cincinnate branches, 1– many-flowered; bracts subtending cymes usually not persistent beyond flowering, sometimes erect forming a coma at apex of flowering stem. Flowers sessile or pedicellate, sometimes spirally arranged on inflorescence axis. Calyx funnel-shaped to tubular, straight or curved, often ventrally gibbous, with pedicel attaching asymmetrically at calyx base opposite the posterior lip, two lipped to regularly 5-lobed, rarely circumscissile at base (*C. coeruleus*); throat glabrous, pubescent or bearded, open or closed by scales or converging lips; if regularly lobed the lobes triangular, lanceolate or spinescent; if two lipped, posterior lip 1-lobed, larger or similar size to other lobes, decurrent or not; anterior lip 4-lobed with lobes variously shaped; lateral lobes lanceolate, triangular to oblong or rounded; held midway between the posterior lobe and median lobes of the anterior lip; median lobes lanceolate to triangular, sometimes separated by a sinus or sometimes fused at base for a longer distance than the lateral and median lobes. Corolla two-lipped with posterior lip shorter than anterior lip, rarely subequal; tube sigmoid to almost straight, narrow and parallel-sided at base, expanding towards throat, very rarely gibbous, rarely annulate; posterior lip 4-lobed, erect or ascending; anterior lip horizontal cucullate to concave. Stamens 4; filaments fused together or free, held within the anterior lip or exserted. Style bifid with subulate lobes, rarely entire. Nutlets ovoid, often dorso-ventrally flattened.

**Figure 4.**
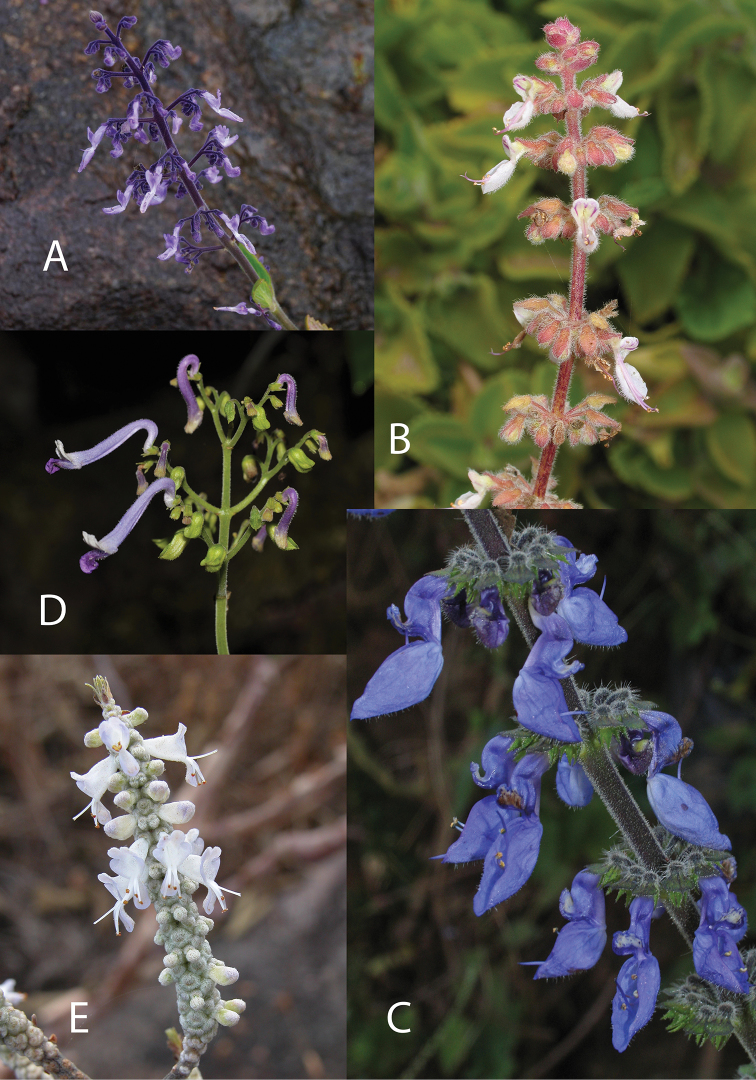
Species of *Coleus* 1. **A***Coleus anamudianus* (Photo: K. Smitha) **B***C. amboinicus* (Photo: Neil Crouch) **C***C. barbatus* (Photo: Neil Crouch) **D***C. bracteatus* (Photo: Witawat Keiwbang) **E***C. harmandii* (Photo: Thamarat Phutthai).

**Figure 5.**
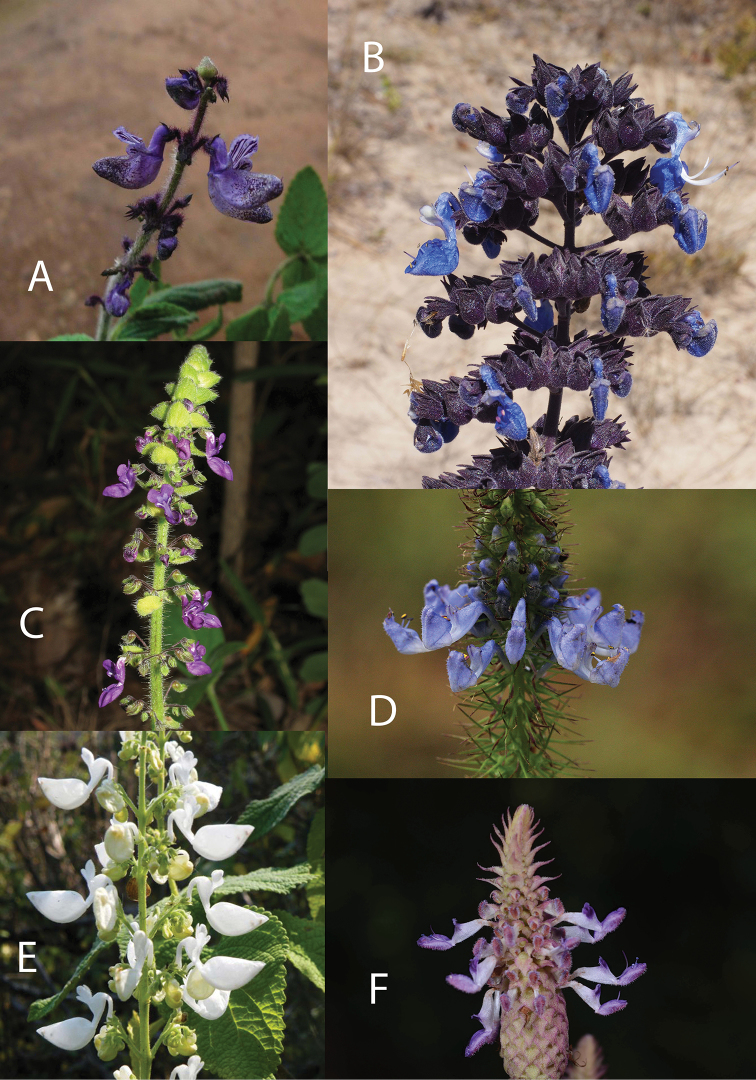
Species of Coleus 2. **A**Coleus maculosus subsp. lanatus (Photo: Vincent Droissart) **B***C. mirabilis* (Photo: David Goyder) **C***C. porphyranthus* (Photo: Neil Crouch) **D***C. orthodontus* (Photo: Bart Wursten) **E***C. tenuicaulis* (Photo: Ernst van Jaarsveld) **F***C. strobilifer* (Photo: Naiyana Tetsana).

294 species in the Old World tropics and subtropics; often cultivated elsewhere.

#### Coleus abyssinicus

Taxon classificationPlantaeLamialesLamiaceae

(Fresen.) A.J.Paton comb. nov.

9AEC70AA05F255F3A4AA30A3A74B7C82

urn:lsid:ipni.org:names:77200982-1

Pycnostachys abyssinica Fresen., Flora 21: 608. 1838. Type: Ethiopia, Sidamo, Rüppell s.n. (holotype: FR? Possibly lost, see Ryding 2006).

##### Distribution.

Ethiopia.

#### Coleus acariformis

Taxon classificationPlantaeLamialesLamiaceae

(P.I.Forst.) P.I.Forst. comb. nov.

A11A3E9217B05708BB7A025596FF5D52

urn:lsid:ipni.org:names:77200983-1

Plectranthus acariformis P.I.Forst., Austrobaileya 9: 279. 2014. Type: Australia, Queensland, Moreton District, Noosa National Park, Mt Emu, 5 Feb. 2014, P.I.Forster & G.Leiper PIF40678 (holotype: BRI; isotypes: K, MEL, NSW, US).

##### Distribution.

Australia: Queensland.

#### Coleus achankoviliensis

Taxon classificationPlantaeLamialesLamiaceae

Smitha & A.J. Paton nom. nov.

4242CECD833E595B97557996DADCBA4E

urn:lsid:ipni.org:names:77200984-1

Anisochilus petraeus J.Mathew & Yohannan, Taiwania 62(2): 144. 2017, non Coleus petraeus (Backer ex Adelb.) A.J.Paton. Type: India, Kollam district, 1.5 km from Achankovil town, Achankovil, J.Mathew 4527 (holotype TBGT; isotype MSSRF).

##### Distribution.

S. India.

#### Coleus actites

Taxon classificationPlantaeLamialesLamiaceae

(P.I.Forst.) P.I.Forst. comb. nov.

2BCF6342FF635FAF8C033388B51E1D38

urn:lsid:ipni.org:names:77200985-1

Plectranthus actites P.I.Forst., Austrobaileya 4: 163. 1994. Type: Australia, Queensland, Leichhardt District, Anvil Peak, Hodgson Range, NE of Clermont, 28 Aug. 1990, P.I.Forster PIF7255 (holotype: BRI).

##### Distribution.

Australia: Queensland.

#### Coleus adenanthus

Taxon classificationPlantaeLamialesLamiaceae

(Dalzell & A.Gibson) A.J.Paton comb. nov.

69E8D465BA125F75BC80A8D8986E7678

urn:lsid:ipni.org:names:77200986-1

Anisochilus adenanthus Dalzell & A.Gibson, Bombay Fl.: 206. 1861. Type: India, the Concan, near Dharwar, Bababoodan hills, Bombay Herbarium of the late N. A. Dalzell. Presented by Mrs Dalzell, April 1878, Dalzell s.n. (holotype: K).
Anisochilus verticillatus Hook.f., Fl. Brit. India 4: 629. 1885. Type: India, the Concan, near Dharwar, Bababoodan hills, Bombay Herbarium of the late N. A. Dalzell. Presented by Mrs Dalzell, April 1878, Dalzell s.n. (lectotype: K, designated by Suddee and Paton 2009).

##### Distribution.

S. India.

#### Coleus adenophorus

Taxon classificationPlantaeLamialesLamiaceae

(Gürke) A.J.Paton comb. nov.

2AC191EE7BB65D059391BDB5D9D9762C

urn:lsid:ipni.org:names:77200987-1

Plectranthus adenophorus Gürke, Bot. Jahrb. Syst. 30: 398. 1901.
Englerastrum adenophorum (Gürke) T.C.E.Fr., Notizbl. Bot. Gart. Berlin-Dahlem 9: 75. 1924. Type: Tanzania, Njombe District: Kipengere, Goetze 960 (holotype: B destroyed).

##### Distribution.

SW. Tanzania.

#### Coleus aegyptiacus

Taxon classificationPlantaeLamialesLamiaceae

(Forssk.) A.J.Paton comb. nov.

64C528D1CC695DC7A62ADED8568FEA39

urn:lsid:ipni.org:names:77200990-1

Ocimum aegyptiacum Forssk., Fl. Aegypt.-Arab.: cxv, 109. 1775.
Plectranthus aegyptiacus (Forssk.) C.Chr., Dansk Bot. Ark. 4(3): 21. 1922.
Plectranthus crassifolius Vahl, Symb. Bot. 1: 44. 1790., nom. illeg.
Germanea crassifolia Poir. in J.B.A.M.de Lamarck, Encycl., suppl. 2: 764. 1812., nom. superfl.
Coleus zatarhendi Benth., Labiat. Gen. Spec.: 50. 1832., nom. superfl.
Plectranthus zatarhendi E.A.Bruce, Bull. Misc. Inform. Kew 1935: 590. 1935., nom. superfl.
Ocimum zatarhendi Forssk., Fl. Aegypt.-Arab.: 109. 1775., nom. inval.
Majana zatarhendi (Forssk.) Kuntze, Revis. Gen. Pl. 2: 524. 1891, nom. inval. Type: Yemen, Beit el Fakih (Bayt al Faqih), Forsskål s.n. (type: not seen, believed destroyed); “Arabia”, Herb. Forsskål s.n. (neotype: KIEL; C, K (photos), designated by Ryding and Paton (2001)).
Coleus tenuiflorus Vatke, Linnaea 43: 92. 1881.
Plectranthus tenuiflorus (Vatke) Agnew, Upland Kenya Wild Fl.: 636. 1974. Type: Kenya, Kitui, Hildebrandt 2742 (lectotype: CORD, designated by Paton et al. (2009)).
Coleus camporum Gürke, Bot. Jahrb. Syst. 19: 214. 1894. Type: Tanzania, Pangani District: Pangani R. ford, Volkens 485 (holotype: B, destroyed; isotype: BM, G).
Coleus ghindanus Schweinf. ex Baker in D.Oliver & auct. suc. (eds.), Fl. Trop. Afr. 5: 435. 1900. Type. Eritrea, Ghinda valley, Schweinfurth & Riva 2149 (holotype: K; isotypes: BR, FT, P).

##### Distribution.

Eritrea to Tanzania, SW. Arabian Peninsula.

#### Coleus affinis

Taxon classificationPlantaeLamialesLamiaceae

(Gürke) A.J.Paton comb. nov.

5338F22FA1865A27B73B40B15B2436DB

urn:lsid:ipni.org:names:77200991-1

Pycnostachys affinis Gürke in H.G.A.Engler, Pflanzenw. Ost-Afrikas, C: 345. 1895.Type: Tanzania, Mwanza, Stuhlmann 4693 (holotype: B, destroyed).
Pycnostachys speciosa Gürke in H.G.A.Engler, Pflanzenw. Ost-Afrikas, C: 345. 1895., non Coleus speciosus Baker f. Type: Kenya/Tanzania, east shore of Lake Victoria, Fischer 499 (lectotype: B, destroyed, designated by Bruce 1940).
Pycnostachys dawei N.E.Br., Gard. Chron., ser. 3, 41: 18. 1907. Type: cultivated from seed sent from Uganda, Dawe s.n. 1906 (holotype: K).

##### Distribution.

Rwanda to E. Trop. Africa.

#### Coleus albicalyx

Taxon classificationPlantaeLamialesLamiaceae

(Suddee) Suddee comb. nov.

8DD2E1F132B05104997CFDB75DA408F9

urn:lsid:ipni.org:names:77200993-1

Plectranthus albicalyx Suddee, Kew Bull. 59: 401. 2004. Type: Thailand, Kanchanaburi, Salak Phrae W.S., Khao Tab Tao, 480 m, 29 Sept. 1998, Suddee et al. 868 (holotype: BKF; isotypes: K, TCD).

##### Distribution.

Thailand.

#### Coleus aliciae

Taxon classificationPlantaeLamialesLamiaceae

(Codd) A.J.Paton comb. nov.

633F827EFAA658FD815B1D407E12624F

urn:lsid:ipni.org:names:77200995-1

Plectranthus madagascariensis var. aliciae Codd, Bothalia 11: 404. 1975.
Plectranthus aliciae (Codd) van Jaarsv. & T.J.Edwards, Bothalia 27: 5. 1997. Type: South Africa, Transkei, Butterworth, Kentani, Pegler 909 (holotype: PRE).

##### Distribution.

South Africa: E. Cape Prov. to S. KwaZulu-Natal.

#### Coleus alloplectus

Taxon classificationPlantaeLamialesLamiaceae

(S.T.Blake) P.I.Forst. & T.C.Wilson comb. nov.

CA72711D3DAB58BFABB6D43BCF9CE1FB

urn:lsid:ipni.org:names:77200997-1

Plectranthus alloplectus S.T.Blake, Contr. Queensland Herb. 9: 29. 1971. Type: Australia, Queensland, Mt Greville, 21 Apr. 1962, S.T.Blake 21703 (holotype: BRI).

##### Distribution.

Australia: New South Wales, Queensland.

#### Coleus alpinus

Taxon classificationPlantaeLamialesLamiaceae

Vatke, Linnaea 37: 322. 1872

94B8D2A9E17C57AFA7D91905AEDB080F

Plectranthus alpinus (Vatke) Ryding, Bot. Jahrb. Syst. 121: 147. 1999. Type: Ethiopia, Edda Jesus near Dawra Tabor (Debra Tabor), 25 Sept. 1863, Schimper s.n. (lectotype: BM, designated by Ryding (1999); isolectotype JE).
Coleus assurgens Baker in D.Oliver & auct. suc. (eds.), Fl. Trop. Afr. 5: 428. 1900.
Plectranthus assurgens (Baker) J.K.Morton, J. Linn. Soc., Bot. 58: 267. 1962. Type: Ethiopia, Begemder, 1863- 8, Schimper s.n. (holotype: K, (K000431867)).
Coleus schoensis Gürke, Bot. Jahrb. Syst. 38: 170. 1906. Type: Ethiopia, Addis Abeba Highlands, Sept. 1900, Ellenbeck 1576 (holotype: B destroyed).
Coleus gracilentus S.Moore, J. Linn. Soc., Bot. 38: 273. 1908. Type: Uganda, E Ruwenzori, Wollaston s.n. (holotype: BM).
Coleus microtrichus Chiov., Nuovo Giorn. Bot. Ital., n.s., 36: 368. 1929. Type: Ethiopia, Arussi, Lake Zuai basin, Abruzzi 37 (holotype: TO; isotype: C).
Coleus lebrunii Robyns, Bull. Jard. Bot. État Bruxelles 17: 73. 1943. Type: DRC, Ruwenzori, Butagu valley, Robyns 4579 (holotype: BR; isotype: K).
Coleus wittei Robyns, Bull. Jard. Bot. État Bruxelles 17: 74. 1943. Type: DRC, Kamatembe, R. Bishkishaki, de Witte 1551 (holotype: BR).

##### Distribution.

Nigeria to Ethiopia and south to Malawi.

#### Coleus altanmouiensis

Taxon classificationPlantaeLamialesLamiaceae

(T.C.Wilson, P.I.Forst., & M.A.M.Renner) T.C.Wilson & P.I.Forst. comb. nov.

9A51C6E3315451788577987807681589

urn:lsid:ipni.org:names:77200999-1

Plectranthus altanmouiensis T.C.Wilson, P.I.Forst., & M.A.M.Renner, Telopea 21: 84. 2018. Type: Cape Melville National Park, Cape York, Cook District, Queensland, 14 Aug 2015, T.C. Wilson 610 and M.A.M. Renner (holotype: NSW 985152; isotype: BRI).

##### Distribution.

Australia: Queensland.

#### Coleus amboinicus

Taxon classificationPlantaeLamialesLamiaceae

Lour., Fl. Cochinch.: 372. 1790

9B7F1879D0D655A1B9FC63FF9377E089

Fig. 4B

Plectranthus amboinicus (Lour.) Spreng., Syst. Veg. 2: 690. 1825.
Majana amboinica (Lour.) Kuntze, Revis. Gen. Pl. 2: 524. 1891. Type: Rumphius plate in Herb. Amb. 5, t. 102/2. 1750 (lectotype: illustration, designated by Cramer (1978); epitype, designated by Suddee et al. (2004) and based on the choice of a representative specimen selected by Launert (1968): Thailand, Pai District: Mae Hong Son, 25 May 1921, Kerr s.n. (epitype: BM).
Ocimum vaalae Forssk., Fl. Aegypt.-Arab.: 111. 1775., nom. rej.
Coleus vaalae (Forssk.) Deflers, Rev. Egypt: 423. 1894.
Coleus aromaticus Benth. in N.Wallich, Pl. Asiat. Rar. 2: 16. 1830. Type: India, Patna, Buchanan-Hamilton Wall. Cat. 2730B (syntypes: K-W (K001116968, K001116969).
Coleus crassifolius Benth. in N.Wallich, Pl. Asiat. Rar. 2: 15. 1830. Type: India, Dindygul, Herb. Wight s.n. in Wall. Cat. 2731 (syntypes: BM, K (K000820149, K (K000820151); K-W (K001116970).
Coleus suganda Blanco, Fl. Filip.: 483. 1837.
Majana suganda (Blanco) Kuntze, Revis. Gen. Pl. 2: 524. 1891. Type: Philippines, Luzon, Maragondong, Cavite Province, Merrill: Species Blancoanae No. 129 (neotype, designated by Suddee *et al.* (2004): K). Blanco’s specimens have not been traced and are believed destroyed.
Coleus carnosus Hassk., Flora 25(2 Beibl.): 25. 1842.
Majana carnosa (Hassk.) Kuntze, Revis. Gen. Pl. 2: 524. 1891.
Coleus suborbicularis Zoll. & Moritzi, Syst. Verz.: 4. 1846., nom. illeg. Type: Indonesia, Zollinger 1950 (holotype: G-DC; isotypes: A, P, S).
Coleus amboinicus var. violaceus Gürke, Bot. Jahrb. Syst. 19: 210. 1894. Type: Tanzania, Moshi District: Lake Chala, Volkens 321 (syntypes: B destroyed, BM, K) & Pangani R. ford, Volkens 487 (syntypes: B, destroyed, BM, K).
Coleus subfrutectosus Summerh., Bull. Misc. Inform. Kew 1928: 392. 1928. Type: Seychelles, Mahé, Botanical Station, Dupont 204 (holotype: K).

##### Distribution.

Kenya to South Africa, Arabian Peninsula, India.

#### Coleus amicorum

Taxon classificationPlantaeLamialesLamiaceae

(S.T.Blake) P.I.Forst. & T.C.Wilson comb. nov.

C4477FB4D2D051DA94433283C66335F7

urn:lsid:ipni.org:names:77201000-1

Plectranthus amicorum S.T.Blake, Contr. Queensland Herb. 9: 26. 1971. Type: Australia, Queensland, Tinaroo Range, 21 Aug. 1963, S.T.Blake 22094 (holotype: BRI).

##### Distribution.

Australia: Queensland.

#### Coleus amiculatus

Taxon classificationPlantaeLamialesLamiaceae

(T.C.Wilson, P.I.Forst. & M.A.M.Renner) T.C.Wilson & P.I.Forst. comb. nov.

1280233D5A75588AA868AA4F21F0A44C

urn:lsid:ipni.org:names:77201001-1

Plectranthus amiculatus T.C.Wilson, P.I.Forst. & M.A.M.Renner, Telopea 21: 81. 2018. Type: Along Pajinka Road, Cook District, Queensland, 8 Aug 2015, T.C. Wilson 595 & M.A.M. Renner (holotype: NSW (NSW985146); isotype: BRI).

##### Distribution.

Australia: Queensland.

#### Coleus amoenus

Taxon classificationPlantaeLamialesLamiaceae

(P.I.Forst.) P.I.Forst. comb. nov.

BDE0CF7E84B05E3D800815F16A3443F5

urn:lsid:ipni.org:names:77201002-1

Plectranthus amoenus P.I.Forst., Austrobaileya 4: 656. 1997. Type: Australia, Queensland, Cook District, Baldy Mt., Atherton, Jun. 1991, P.I.Forster PIF8488 (holotype: BRI; isotypes: CNS, MEL).

##### Distribution.

Australia: Queensland.

#### Coleus anamudianus

Taxon classificationPlantaeLamialesLamiaceae

(Smitha & Sunojk.) Smitha comb. nov.

129CD01A824D5810A87FE38218EFDCE4

urn:lsid:ipni.org:names:77201003-1

Fig. 4A

Plectranthus anamudianus Smitha & Sunojk., Phytotaxa 284(1): 52. 2016. Type: India, Kerala, Idukki district, in valley, wet rocky surfaces, Rajamala, Smitha & Sunojkumar 135412 (holotype: CALI; isotype MH).

##### Distribution.

S. India.

#### Coleus angolensis

Taxon classificationPlantaeLamialesLamiaceae

(G.Taylor) A.J.Paton comb. nov.

61481BF0F4B85C41B0480E968422DAAC

urn:lsid:ipni.org:names:77201004-1

Pycnostachys angolensis G.Taylor, J. Bot. 69 (suppl. 2): 164. 1931. Type: Angola, Casima, Anha, Gossweiler 1734 (holotype: BM).

##### Distribution.

Angola.

#### Coleus angulatus

Taxon classificationPlantaeLamialesLamiaceae

(Hedge) A.J.Paton & Phillipson comb. nov.

EA63D4B6F7B1530C9F2AEE5E14784CFE

urn:lsid:ipni.org:names:77201005-1

Plectranthus angulatus Hedge, Fl. Madag. 175: 211. 1998. Type: Madagascar, Bekopaka, May 1964, Bosser 19616 (holotype: P; isotypes: E, MO, TAN).

##### Distribution.

Madagascar.

#### Coleus apoensis

Taxon classificationPlantaeLamialesLamiaceae

Elmer, Leafl. Philipp. Bot. 7: 2694. 1915

06FC86CC9CED5587B63A7E0C9208C19F

Plectranthus apoensis (Elmer) H.Keng, Gard. Bull. Singapore 24: 147. 1969. Type: Phillipines, Mindanao, Davao distr., Todaya, Mount Apo, Sept. 1909, A.D.E. Elmer 11601 (holotype PNH (not seen); isotypes: GH, K).

##### Distribution.

Philippines (Mindanao).

#### Coleus apreptus

Taxon classificationPlantaeLamialesLamiaceae

(S.T.Blake) P.I.Forst. & T.C.Wilson comb. nov.

C6356DCBB1465AD1970E366C039A5104

urn:lsid:ipni.org:names:77201006-1

Plectranthus apreptus S.T.Blake, Contr. Queensland Herb. 9: 47. 1971. Type: Australia, Queensland, Freshwater Creek, near Cairns, 17 May 1962, S.T.Blake 21730 (holotype: BRI).

##### Distribution.

Australia: Queensland.

#### Coleus apricus

Taxon classificationPlantaeLamialesLamiaceae

(P.I.Forst.) P.I.Forst. comb. nov.

AD3BDD541DEC5231A44A3EC861EBA00D

urn:lsid:ipni.org:names:77201007-1

Plectranthus apricus P.I.Forst., Austrobaileya 4: 165. 1994. Type: Australia, Queensland, Cook District. Turtle Rock Area, 22 Jan. 1991, P.I. Forster PIF12831 & A.R.Bean (holotype: BRI, isotypes: CNS, K, MEL).

##### Distribution.

Queensland.

#### Coleus arabicus

Taxon classificationPlantaeLamialesLamiaceae

Benth. in A.P. de Candolle, Prodr. 12: 79. 1848

3879FB0F480F51E6B4B3C1C667A62980

Majana arabica (Benth.) Kuntze, Revis. Gen. Pl. 2: 524. 1891. Type: Arabia, Botta s.n. (holotype: P).
Plectranthus asirensis J.R.I.Wood, Kew Bull. 37: 601. 1983. Type: Saudi Arabia, Taif, Botta s.n. (holotype: P; isotype K).
Teucrium mazziarii Heldr. ex Nyman, Consp. Fl. Eur.: 567. 1881, nom. inval.

##### Distribution.

Arabian Peninsula.

#### Coleus arenicola

Taxon classificationPlantaeLamialesLamiaceae

(P.I.Forst.) P.I.Forst. comb. nov.

9C6656551A725A9D9FC845F67121CCB2

urn:lsid:ipni.org:names:77201008-1

Plectranthus arenicola P.I.Forst., Muelleria 7: 375. 1991. Type: Australia, Queensland, Plant cultivated at St Lucia, Brisbane from material the same collection as P.I.Forster 5456 from Moreton Telegraph Station, near Kennedy Hill, 22 Oct. 1989, P.I.Forster PIF5835 (holotype: BRI; isotypes: CNS, K, MEL).

##### Distribution.

Australia: Queensland.

#### Coleus argentatus

Taxon classificationPlantaeLamialesLamiaceae

(S.T.Blake) P.I.Forst. & T.C.Wilson comb. nov.

48B8F571C0BF5A29AC4EEDD40C309AF9

urn:lsid:ipni.org:names:77201009-1

Plectranthus argentatus S.T.Blake, Contr. Queensland Herb. 9: 27. 1971. Type: Australia, Queensland, Mt Roberts, McPherson Range, 5 May 1955, S.T.Blake 19803 (holotype: BRI).

##### Distribution.

Australia: Queensland to NE. New South Wales. Widely cultivated (or hybrids from it).

#### Coleus argenteus

Taxon classificationPlantaeLamialesLamiaceae

(Gamble) A.J.Paton comb. nov.

C109F0C89FC154B29578E07A2ADE6287

urn:lsid:ipni.org:names:77201010-1

Anisochilus argenteus Gamble, Fl. Madras: 1127. 1924. Type: India, Madras, Pulney Hills, Kodaikanal Ghat new road, 29 Dec. 1898, Bourne 1441C (lectotype: K; isolectotype: K, designated by Suddee & Paton (2009).

##### Distribution.

S. India.

#### Coleus argentifolius

Taxon classificationPlantaeLamialesLamiaceae

(Ryding) A.J.Paton comb. nov.

099168CBFEAA5872B2D50D2F4B6FEC2D

urn:lsid:ipni.org:names:77201011-1

Plectranthus argentifolius Ryding, Kew Bull. 54: 122. 1999. Type: Solalia, Sanaag Region, Erigavo Escarpment, Popov 1218 (holotype: K; isotype: ETH).

##### Distribution.

N. Somalia, Yemen.

#### Coleus articulatus

Taxon classificationPlantaeLamialesLamiaceae

(I.M.Johnst.) A.J.Paton comb. nov.

4A0A3296CA17545A9FBD470010AA3552

urn:lsid:ipni.org:names:77201012-1

Symphostemon articulatus I.M.Johnst., Contr. Gray Herb., n.s., 73: 38. 1924. Type: Angola, east of Cuanza (Coanza) R., Curtis 309 (holotype: GH).
Plectranthus hockii De Wild., Repert. Spec. Nov. Regni Veg. 11: 542. 1913.
Plectranthus katangensis De Wild., Ann. Mus. Congo Belge, Bot., sér. 4, 2: 135. 1913., nom. superfl.
Holostylon katangense (De Wild.) Robyns & Lebrun, Ann. Soc. Sci. Bruxelles, Sér. B 49: 105. 1929., nom. superfl.
Ocimum hockii (De Wild.) Robyns & Lebrun, Rev. Zool. Bot. Africaines 16: 368. 1928., non Coleus hockii De Wild. Type: DRC, Upper Katanga, Dilemba, Hock s.n. (syntype: BR, K) & Plateau de Shinkwari (Manika), Hock s.n. (syntype: BR).

##### Distribution.

SW. Tanzania, DRC, Malawi, Zambia, Angola.

#### Coleus ater

Taxon classificationPlantaeLamialesLamiaceae

A.J.Paton nom. nov.

A02D111B37595FB38F6474D45F1A32F8

urn:lsid:ipni.org:names:77201014-1

Anisochilus wightii Hook.f., Fl. Brit. India 4: 628. 1885., non Coleus wightii Benth. Type: India, Deccan Peninsula, Anamallay Mts, July 1851, Herb. Wight 2132/1 (holotype: K; isotypes: C, L).

##### Distribution.

S. India.

#### Coleus auriglandulosus

Taxon classificationPlantaeLamialesLamiaceae

(A.J.Paton) A.J.Paton comb. nov.

F4AC65F6AC7F5D84BAB5E216E51999B1

urn:lsid:ipni.org:names:77201015-1

Plectranthus auriglandulosus A.J.Paton, Fl. Trop. E. Afr., Lamiac.: 307. 2009. Type: Kenya, Tana R. District: Tana River National Primate Reserve, near Makere, Luke et al. TPR 605 (holotype, K; isotype: EA).

##### Distribution.

SE. Kenya.

#### Coleus australis

Taxon classificationPlantaeLamialesLamiaceae

(R.Br.) A.J.Paton comb. nov.

9FAFC92A22F95381BA495DA75604993E

urn:lsid:ipni.org:names:77201016-1

Plectranthus australis R.Br., Prodr. Fl. Nov. Holl.: 506. 1810.
Moschosma australe (R.Br.) Benth., Labiat. Gen. Spec.: 708. 1835.
Moschosma brownii Heynh., Alph. Aufz. Gew.: 421. 1847.
Plectranthus parviflorus var. australis (R.Br.) Briq. in H.G.A.Engler & K.A.E.Prantl, Nat. Pflanzenfam. 4(3a): 357. 1895.
Germanea australis (R.Br.) Britten, Bot. Cook Voy. 3: 75. 1905. Type: New South Wales, Port Jackson, 17 May, 1802, R. Brown (lectotype: BM; isolectotypes: BRI, K, designated by Blake (1971)).
Plectranthus australis var. vulgaris Domin, Biblioth. Bot. 89: 564. 1928., nom. inval.
Plectranthus parviflorus Willd., Hort. Berol.: t. 65. 1806., non Coleus parviflorus Benth. Type: cultivated in Berlin, unknown origin, Wildenow Herbarium 11078 (holotype: B).
Plectranthus paniculatus Jacq., Fragm. Bot.: 62.tab 91. 1806., non Coleus paniculatus Benth. Type: Cultivated in Vienna. Illustration cited above.(lectotype: illustration designated here).
Plectranthus parviflorus Spreng. in L.V.F.Henckel von Donnersmarck, Adumbr. Pl. Hort. Hal.: 17. 1806., nom. illeg.
Germanea parviflora Poir. in J.B.A.M.de Lamarck, Encycl., suppl. 2: 764. 1811.
Majana parviflora (Poir.) Kuntze, Revis. Gen. Pl. 2: 524. 1891.
Plectranthus sieberi Benth., Labiat. Gen. Spec.: 710. 1835. Type: Australia, “In Nova Hollandia tropica Sieber” (holotype: K).
Plectranthus parviflorus var. elatior Benth. in A.P.de Candolle, Prodr. 12: 67. 1848. Type: Northern Territory, Port Essington, Armstrong (holotype: K).
Plectranthus parviflorus var. major F.M.Bailey, Queensland Agric. J. 28: 199. 1912., nom. nud.
Plectranthus parviflorus var. minor F.M.Bailey, Queensland Agric. J. 28: 199. 1912., nom. nud.
Plectranthus australis f. densiflora Domin, Biblioth. Bot. 89: 564. 1928. Type: Queensland, Tamborin Mtn., Domin s.n. (holotype: PR).

##### Distribution.

Australia, Lesser Sunda Is. to Pacific.

#### Coleus autranii

Taxon classificationPlantaeLamialesLamiaceae

Briq., Bull. Herb. Boissier 2: 129. 1894

465B3DE94EF95525A706FA1CB6DEFB41

Solenostemon autranii (Briq.) J.K.Morton, Novon 8: 266. 1998.
Plectranthus autranii (Briq.) Erhardt, Götz & Seybold, Grosse Zander 2: 1825. 2008.
Calchas autranii (Briq.) P.V.Heath, Calyx 5: 160. 1996. Type: Ethiopia, no precise locality, 1853, Schimper 693 (holotype: G; isotype: K).
Coleus silvaticus Gürke, Bot. Jahrb. Syst. 19: 219. 1894.
Solenostemon silvaticus (Gürke) Agnew, Upland Kenya Wild Fl.: 640. 1974. Types: Tanzania, Lushoto District: Usambara, Holst 3704 & 3708 (syntypes: B, destroyed); neotype: Tanzania, W Usambara Mts, Shagai Forest, near Sunga, Drummond & Hemsley 2593 (neotype: K, designated by Paton et al. (2009)).
Plectranthus tysonii Gürke, Bot. Jahrb. Syst. 26: 77. 1898. Type: South Africa, Umzimkulu, Clydesdale, Tyson 1295 (isotypes: K, SAM (numberered 1295 SAM herbarium number and cited by Gürke), PRE (PRE numbered Hb. Tyson 2769 and cross referenced to 1295 as cited in Codd (1985)).
Coleus odoratus Gürke, Bot. Jahrb. Syst. 38: 167. 1906. Type: Tanzania, W Usambara Mts, Sakare, Engler 939 (syntype: B, destroyed; photo K); & Engler 1006 (syntype: B destroyed).
Coleus latidens S.Moore, J. Linn. Soc., Bot. 38: 273. 1908.
Calchas latidens (S.Moore) P.V.Heath, Calyx 6: 51. 1999. Type: Uganda, E Ruwenzori, Wollaston s.n. (holotype: BM).
Coleus ostinii Chiov., Ann. Bot. (Rome) 9: 125. 1911. Type: Ethiopia, Dembia, Angareb to Gondar and Fenter, Chiovenda 1450 (syntype: FT); Cococc above Gondar, Chiovenda 1854 (syntype: FT).
Coleus darfurensis R.D.Good, J. Bot. 62: 138. 1924.
Solenostemon darfurensis (R.D.Good) B.Mathew, Kew Bull. 25: 258. 1971. Type: Sudan, Jebel Mara, Lynes 4c (holotype: BM; fragment K).
Coleus uliginosus T.C.E.Fr., Notizbl. Bot. Gart. Berlin-Dahlem 11: 29. 1930. Type: Kenya, Meru, R.E. & T.C.E. Fries 1762 (syntype: B destroyed; isosyntype: K).
Coleus ascendens Gilli, Ann. Naturhist. Mus. Wien 77: 32. 1973. Type: Tanzania, Morogoro District: Uluguru Mts, Chenzema (Tchenzema), Gilli 456 (holotype: W).

##### Distribution.

Ethiopia, Central Africa, East Africa to South Africa.

#### Coleus barbatus

Taxon classificationPlantaeLamialesLamiaceae

(Andrews) Benth. ex G.Don in J.C.Loudon, Hort. Brit.: 483. 1830

A0C4A89A257156DE835504673BC22D8C

Fig. 4C

Plectranthus barbatus Andrews, Bot. Repos. 10: t. 594. 1810. Type: Illustration of cultivated material in Bot. Rep. 9, t. 594. 1809., (lectotype: illustration cited above; Eritrea, Dekemehare, Colville 47 (epitype: K, designated by Ryding (1999)).
Coleus forskalaei auct., non Briq.

##### Distribution.

Eritrea to East and Central Africa, Arabian Peninsula, Indian Subcontinent to SC. China. Widely cultivated.

##### Notes.

The name *Coleus forskalaei* or *forskohlii* has been often used incorrectly for this species.

#### Coleus barbatus var. barbatus

Taxon classificationPlantaeLamialesLamiaceae

DEA7D2C4AD125CCC9290E4A500A15B82

Ocimum asperum Roth, Nov. Pl. Sp.: 268. 1821.
Plectranthus asper (Roth) Spreng., Syst. Veg. 2: 690. 1825.
Orthosiphon asperus (Roth) Benth. ex Sweet, Hort. Brit., ed. 2: 413. 1830. Type: India, Mysore, Nundidroog, Heyne s.n. in Wall. Cat. 2728C (holotype: B destroyed; isotype: K-W).
Plectranthus comosus Sims, Bot. Mag. 49: t. 2318. 1822. Type: illustration of plant originally from Nepal, (lectotype: illustration cited above).
Coleus huberi Regel syn. nov., Trudy Imp. S.-Peterburgsk. Bot. Sada 7: 543. 1881. Type: Russia, cultivated in St Petersburg from seed collected in Ethiopia, (type not found in LE).
Coleus penzigii Dammann ex Baker, Gard. Chron., ser. 3, 14: 616. 1893. Type: cultivated at Kew, received from Hort. Dammann, probably originating from material collected by Penzig in “the mountains of Abyssinia” (holotype: K).
Coleus vestitus Baker, Bull. Misc. Inform. Kew 1895: 224. 1895. Type: Somalia, Golis Range, E. Cole s.n. (syntype: K); Lort-Phillips (syntype: K).
Plectranthus pseudobarbatus J.K.Morton, Novon 8: 265. 1998. Type: Somalia, Togdheer Region, Golis Range, Cole s.n. (lectotype: K; designated by Ryding (1999)).
Coleus forskohlii var. adoensis Briq., Annuaire Conserv. Jard. Bot. Genève 2: 235. 1898. Type: Ethiopia, Adua, 1 Oct. 1837, Schimper 333 (holotype: not seen in G; isotypes: BR, K, MPU, WAG).
Coleus speciosus Baker f., J. Bot. 1899: 64. 1899. Type: Somalia, Woqooyi Galbeed/Togdheer Regions, “Wagga Mountains”, Lort-Phillips s.n. (holotype: BM; isotype K).
Coleus coerulescens Gürke, Bot. Jahrb. Syst. 38: 169. 1906.
Plectranthus coerulescens (Gürke) R.H.Willemse, Kew Bull. 40: 95. 1985. Type: Ethiopia, Harar, Ellenbeck 743 (holotype: B, destroyed; isotype: K).
Coleus adolfi-friderici Perkins in G.W.J.Mildbraed (ed.), Wiss. Erg. Deut. Zentr.-Afr. Exped., Bot. 2: 550. 1913. Type: Rwanda, Mohasi, Büschen to Buganza, Mildbraed 671 (holotype: B, destroyed).

##### Distribution.

Eritrea to N. Tanzania, Arabian Peninsula, Indian Subcontinent to SC. China.

#### Coleus barbatus var. grandis

Taxon classificationPlantaeLamialesLamiaceae

(L.H.Cramer) A.J.Paton comb. nov.

E878A6A3237858CEAE6A8360E5636DD8

urn:lsid:ipni.org:names:77201018-1

Coleus grandis L.H.Cramer, Kew Bull. 32: 556. 1978.
Plectranthus grandis (L.H.Cramer) R.H.Willemse, Blumea 25: 509. 1979.
Plectranthus barbatus var. grandis (L.H.Cramer) Lukhoba & A.J.Paton, Kew Bull. 58: 915. 2003. publ. 2004. Type: Sri Lanka, Sita Eleiya, Cramer 3869 (holotype: PDA; isotypes: K, US).
Coleus kilimandschari Gürke, Abh. Königl. Akad. Wiss. Berlin 1891: 359. 1892.
Plectranthus kilimandschari (Gürke) H.I.Maass in R.Mansfeld, Verz. Landwirtsch. Gärtn. Kulturpfl. 3: 1136. 1986. Types: Tanzania, Kilimanjaro, Marangu, Meyer 377 (syntype: B, destroyed) & forest nearby, 2400 m, Meyer 116 (syntype: B, destroyed). Marangu, Volkens 427 (neotype, designated by Paton et al. (2009): K; isoneotype: G).

##### Distribution.

NE. & E. Trop. Africa to DRC.

#### Coleus bariensis

Taxon classificationPlantaeLamialesLamiaceae

(Ryding) A.J.Paton comb. nov.

92399E0187595E0DB39288E1841E2E98

urn:lsid:ipni.org:names:77201019-1

Plectranthus bariensis Ryding, Kew Bull. 54: 123. 1999. Type: Somalia, Bari Region, Escarpment of Bunder Murrayha, Buraha Dhaxi, Thulin & Warfa 5286 (holotype: UPS).

##### Distribution.

N. Somalia.

#### Coleus batesii

Taxon classificationPlantaeLamialesLamiaceae

(Baker) A.J.Paton comb. nov.

BD309928DFFF5422B0CDBC3B24D3BDED

urn:lsid:ipni.org:names:77201020-1

Pycnostachys batesii Baker in D.Oliver & auct. suc. (eds.), Fl. Trop. Afr. 5: 386. 1900. Type: Cameroon, Efulen, Bates 372 (holotype: K.; isotype: BM).

##### Distribution.

Cameroon to SW. Uganda.

#### Coleus batianoffii

Taxon classificationPlantaeLamialesLamiaceae

(P.I.Forst.) P.I.Forst. comb. nov.

E9555CAC4FE85C80BD4CBF70E42211E5

urn:lsid:ipni.org:names:77201021-1

Plectranthus batianoffii P.I.Forst., Austrobaileya 7: 707. 2008. Type: Australia, Queensland, Cook District, Palfrey Island, 23 July 1990, G.N.Batianoff 12118 (holotype: BRI).

##### Distribution.

Australia: Queensland.

#### Coleus bellus

Taxon classificationPlantaeLamialesLamiaceae

(P.I.Forst.) P.I.Forst. comb. nov.

06A284A032415856B3E925F135E6EB65

urn:lsid:ipni.org:names:77201402-1

Plectranthus bellus P.I.Forst., Austrobaileya 8: 388. 2011. Type: Australia, Queensland, Cook District, Daintree National Park, Adeline Creek headwaters, 18 May 1999, P.I.Forster PIF24610 & R.Booth (holotype: BRI; isotype, MEL).

##### Distribution.

Australia: Queensland.

#### Coleus betonicifolius

Taxon classificationPlantaeLamialesLamiaceae

(Baker) A.J.Paton comb. nov.

07D7C747F48F5F44B0DC406999B4B9DC

urn:lsid:ipni.org:names:77201022-1

Plectranthus betonicifolius Baker, Bull. Misc. Inform. Kew 1895: 72. 1895. Types: Zambia, Fwambo, Carson 64 & 79 (syntypes: K).
Elsholtzia carsonii Baker in D.Oliver & auct. suc. (eds.), Fl. Trop. Afr. 5: 450. 1900. Type: Zambia, Fwambo, S of Lake Tanganyika, 1890, Carson s.n. (holotype: K).
Coleus baumii Gürke in O. Warburg (ed.), Kunene-Sambesi Exped.: 357. 1903. Type: Angola, Cuito, Baum 544 (holotype: B, destroyed ; isotypes: G, K, W).
Coleus kasomenensis De Wild., Repert. Spec. Nov. Regni Veg. 11: 514. 1913. Types: DRC, Upper Katanga, Kasomenia, Kassner 2555 (syntype: BR; isosyntype: K) & near Lubumbashi (Elizsabethville), Homblé 246 (syntype: BR) & Welgelegen, Bequaert 382 & 564 (syntypes: BR).

##### Distribution.

Tanzania, DRC, Zambia and Angola.

##### Notes.

The West African *C. lyratus* (A.Chev.) Roberty has been removed from synonymy and recognized here as a separate species, differing from Paton et al. (2013).

#### Coleus bifidus

Taxon classificationPlantaeLamialesLamiaceae

(A.J.Paton) A.J.Paton comb. nov.

180A6BB5B85C5744849E5D345B16CB86

urn:lsid:ipni.org:names:77201023-1

Plectranthus bifidus A.J.Paton, Fl. Zambes. 8(8): 235. 2013. Type: Zambia, Solwezi, Apr. 1960, Robinson 3520 (holotype: K; isotype: SRGH).

##### Distribution.

Zambia.

#### Coleus bishopianus

Taxon classificationPlantaeLamialesLamiaceae

(Gamble) Smitha & A.J.Paton comb. nov.

82583CD31FFB5BE694526C19B9C2F285

urn:lsid:ipni.org:names:77201025-1

Plectranthus bishopianus Gamble, Bull. Misc. Inform. Kew 265. 1924. Type: India. Tamil Nadu: Pulneys, shola forest at Pillar Rocks, 20 May 1901, A.G. Bourne and E.G. Bourne 1398 (lectotype: K, designated by Matthew (1993)).

##### Distribution.

S. India.

#### Coleus bipartitus

Taxon classificationPlantaeLamialesLamiaceae

(P.I.Forst.) P.I.Forst. comb. nov.

8B04245AEAC85415A52D20D1775947C6

urn:lsid:ipni.org:names:77201026-1

Plectranthus bipartitus P.I.Forst., Austrobaileya 9: 208. 2014. Type: Australia, Queensland, Cook District, Hann Tableland National Park, west of Mareeba, 8 April 2013, P.I.Forster PIF39595 (holotype: BRI; isotypes: CNS, K, MEL).

##### Distribution.

Australia: Queensland.

#### Coleus blakei

Taxon classificationPlantaeLamialesLamiaceae

(P.I.Forst.) P.I.Forst. comb. nov.

EF2A5A6B074153B38E3771B463906444

urn:lsid:ipni.org:names:77201027-1

Plectranthus blakei P.I.Forst., Muelleria 7: 417. 1992. Type: Australia, Queensland, Leichardt District, Mimosa Creek, Blackdown Tableland National Park, 16 May 1984, P.I.Forster PIF1782 (holotype: BRI; isotypes: K, MEL, NSW).

##### Distribution.

Australia: Queensland.

#### Coleus bojeri

Taxon classificationPlantaeLamialesLamiaceae

Benth., Labiat. Gen. Spec.: 52. 1832

6720DA33BF6B54D1A089DF1E92CE22BB

Solenostemon bojeri (Benth.) Guillaumet & A.Cornet, Adansonia, n.s., 15: 525. 1976.
Calchas bojeri (Benth.) P.V.Heath, Calyx 5: 160. 1997.
Plectranthus bojeri (Benth.) Hedge, Fl. Madag. 175: 218. 1998. Type: Madagascar, Emirna, Betani-mena, Bojer s.n. (lectotype: P, designated by Hedge et al. 1998).
Mitsa maculata Chapel. ex Benth., Labiat. Gen. Spec.: 52. 1832. nom. inval.
Plectranthus maculatus Bojer ex Benth., Labiat. Gen. Spec.: 52. 1832. nom. inval.
Coleus latifolius Hochst. ex Benth. in A.P.de Candolle, Prodr. 12: 74. 1848.
Majana latifolia (Hochst. ex Benth.) Kuntze, Revis. Gen. Pl. 2: 524. 1891.
Solenostemon latifolius (Hochst. ex Benth.) J.K.Morton, J. Linn. Soc., Bot. 58: 271. 1962. Types: Ethiopia, Gapdia, 17 Sept. 1838, Schimper 825 (syntypes: BM, E, K, G, GH, LG, STU, W) & Gafta, 18 Sept. 1838, Schimper 1228 (syntypes: BM, K, G, LG, W).
Coleus latifolius var. elatior Vatke, Linnaea 37: 322. 1872. Type: Ethiopia, Tigray, NNW of Axum, Bellitschen, 8 Sept. 1862, Schimper 378 (syntype: BM, JE).
Coleus micranthus Maxim., Trudy Imp. S.-Peterburgsk. Bot. Sada 3(1): 108. 1875. Type: seed from Ethiopia, 1872, Schimper (holotype: LE).
Plectranthus cymosus Baker, J. Linn. Soc., Bot. 21: 434. 1885.
Solenostemon cymosus (Baker) Guillaumet & A.Cornet, Adansonia, n.s., 15: 524. 1976., nom. illeg. Type: Central Madagascar, Baron 2250 (holotype: K; isotype: P).
Coleus bernieri Briq., Bull. Herb. Boissier 2: 128. 1894.
Solenostemon bernieri (Briq.) Guillaumet & A.Cornet, Adansonia, n.s., 15: 525. 1976.
Calchas bernieri (Briq.) P.V.Heath, Calyx 5: 160. 1997. Type: Madagascar, North of Madagascar, Bernier 147 (holotype: G).
Coleus gracilifolius Briq., Bull. Herb. Boissier 2: 127. 1894.
Solenostemon gracilifolius (Briq.) Guillaumet & A.Cornet, Adansonia, n.s., 15: 525. 1976. Type: Madagascar, Fito (Ambanivoulu), Goudot s.n. 1832 (holotype: G).
Coleus schweinfurthii Briq., Bot. Jahrb. Syst. 19: 181. 1894., nom. illeg., non C. schweinfurthii Vatke.
Coleus briquetii Baker in D.Oliver & auct. suc. (eds.), Fl. Trop. Afr. 5: 441. 1900., nom. superfl.
Calchas briquetii (Baker) P.V.Heath, Calyx 5: 160. 1997. nom. illeg.
Plectranthus bongensis Baker in D.Oliver & auct. suc. (eds.), Fl. Trop. Afr. 5: 410. 1900. Type: Sudan, Bongo, Jur (Gir), Schweinfurth 2490 (holotype: K; isotype: PRE).
Coleus dewevrei Briq., Bull. Soc. Roy. Bot. Belgique 37: 71. 1899.
Calchas dewevrei (Briq.) P.V.Heath, Calyx 6: 51. 1999. Type: DRC, Dewèvre 1092a (holotype: BR).
Coleus latifolius var. madiensis Baker in D.Oliver & auct. suc. (eds.), Fl. Trop. Afr. 5: 437. 1900. Type: Uganda, West Nile District: Madi, Grant in Speke & Grant s.n. (holotype: K).
Coleus heterotrichus Briq., Bull. Soc. Roy. Bot. Belgique 40: 40. 1901. Type: DRC, Bolobo, Demeuse 1155 (holotype: G; isotype: Z).
Coleus copiosiflorus Briq., Mém. Soc. Bot. France 8: 288. 1917.
Calchas copiosiflorus (Briq.) P.V.Heath, Calyx 5: 160. 1997. Types: Republic Central Africa, Haute Ombella, Kaga Do, 26 Oct. 1902, A. Chevalier 5967 (syntype: P); Mali, Koulikoro, 6–14 Oct. 1899, A. Chevalier s.n. (syntype, P).
Coleus phymatodes Briq., Mém. Soc. Bot. France 8: 289. 1917. Type: Mali, Koulikoro, 6–14 Oct 1899, A. Chevalier s.n. (holotype, P).
Coleus claessensii De Wild., Bol. Soc. Ibér. Ci. Nat. 19: 117. 1920.
Calchas claessensii (De Wild.) P.V.Heath, Calyx 5: 160. 1997. Types: DRC, Mobwassa, Claessens 741 (syntype: BR) & Ekuta on the Lua, Sapin s.n. (syntype: BR).
Coleus homblei De Wild., Contr. Fl. Katanga: 174. 1921. Types: DRC, Lubumbashi (Elisabethville), Homblé 210 (syntype: BR) & Tshisinka, Homblé 1259 (syntype: BR.).
Coleus ringoetii De Wild., Contr. Fl. Katanga: 174. 1921. Type: DRC, Shinsenda, Ringoet 546 (holotype: BR).
Coleus termetophilus De Wild., Contr. Fl. Katanga: 175. 1921. Type: DRC, Lubumbashi (Elisabethville), Homblé 220 (holotype: BR; isotype: Z).
Coleus platostomoides Robyns & Lebrun, Rev. Zool. Bot. Africaines 16: 362. 1928.
Solenostemon platostomoides (Robyns & Lebrun) Troupin, Bull. Jard. Bot. Natl. Belg. 55: 299. 1985.
Calchas platostomoides (Robyns & Lebrun) P.V.Heath, Calyx 6: 51. 1999. Type: DRC, Lake Kivu, Saké Bay, Kuteruzi, Robyns 2482 (holotype: BR; isotype: K).
Coleus quarrei Robyns & Lebrun, Rev. Zool. Bot. Africaines 16: 3. 1928. Type: DRC, Lubumbashi (Elisabethville), Kibembe Farm, Quarré 608 (holotype: BR).
Coleus collinus Lebrun & L.Touss., Bull. Jard. Bot. État Bruxelles 17: 81. 1943.
Solenostemon collinus (Lebrun & L.Touss.) Troupin, Bull. Jard. Bot. Natl. Belg. 55: 299. 1985.
Calchas collinus (Lebrun & L.Touss.) P.V.Heath, Calyx 5: 160. 1997. Type: Congo, Kivu, Rutshuru, Nov. 1937, Lebrun 8232 (holotype: BR).

##### Distribution.

Eastern, Central & Southern Africa, Madagascar.

#### Coleus botryosus

Taxon classificationPlantaeLamialesLamiaceae

(A.J.Paton) A.J.Paton comb. nov.

0ED09930E15C522FB5095E2705A2679D

urn:lsid:ipni.org:names:77201028-1

Plectranthus botryosus A.J.Paton, Fl. Trop. E. Afr., Lamiac.: 335. 2009. Type: Tanzania, Songea District: just E of R. Gembabili ± 3 km SW of Kitai, Milne-Redhead & Taylor 9735 (holotype:K, sheet 2; isotypes EA, K sheet 1).

##### Distribution.

S. Tanzania.

#### Coleus bourneae

Taxon classificationPlantaeLamialesLamiaceae

(Gamble) Smitha & A.J.Paton comb. nov.

3A95EF3A890957CA809594B273F760C2

urn:lsid:ipni.org:names:77201029-1

Plectranthus bourneae Gamble, Bull. Misc. Inform. Kew 1924: 264. 1924. Type: India. Tamil Nadu: Pulneys, Coelogyne rock, Poombarai road 16 May 1901, A.G. Bourne & E.G. Bourne 2028 (lectotype: K, designated by Smitha and Sunojkumar 2018).

##### Distribution.

S. India.

#### Coleus bracteatus

Taxon classificationPlantaeLamialesLamiaceae

Dunn, Notes Roy. Bot. Gard. Edinburgh 8: 158. 1913

372C6C047F805D8FB1B7002D2EF7A263

Fig. 4D

Plectranthus bracteatus (Dunn) Suddee, Kew Bull. 59: 399. 2004. Type: China, Yunnan, Puerh, 1350 m, Henry 13498 (lectotype: K, designated by Suddee et al. 2004; isolectotype: NY).
Plectranthus saphinensis Phuong, Bot. Zhurn. (Moscow & Leningrad) 67: 1131. 1982. Type: Vietnam, Sa Phin, Prov. Ha Tuyen, 15 Sept. 1977, Phuong 294B (holotype: HN; isotype: HN).

##### Distribution.

China: Yunnan to Indo-China.

#### Coleus brazzavillensis

Taxon classificationPlantaeLamialesLamiaceae

A.Chev., Veg. Ut. Afr. Trop. Franç. 1: 124. 1905

F704D6AF54785946AE429782510F1C65

Coleus brazzavillensis A.Chev., Veg. Ut. Afr. Trop. Franç. 1: 124. 1905. Type: Congo, Brazzaville, Mission du Chari-Lac Chad, Chevalier 11154 (holotype: P).

##### Distribution.

Republic of Congo (Brazzaville).

#### Coleus buchananii

Taxon classificationPlantaeLamialesLamiaceae

(Baker) Brenan, Mem. New York Bot. Gard. 9: 43. 1954

1C61C2F2F05152CFB4E004879409A3AC

Plectranthus buchananii Baker in D.Oliver & auct. suc. (eds.), Fl. Trop. Afr. 5: 402. 1900. Type: Malawi, Shire Highlands, Nakajumbu, Buchanan 365 (holotype: K).
Coleus scaposus C.H.Wright, Bull. Misc. Inform. Kew 1906: 167. 1906. Type: Malawi, Namasi, Cameron 60 (holotype: K).
Coleus hockii De Wild., Repert. Spec. Nov. Regni Veg. 11: 514. 1913. Type: DRC, Upper Katanga, valley of small Luembe, Hock s.n. (holotype: BR).

##### Distribution.

Tanzania to S. Trop. Africa.

#### Coleus burorum

Taxon classificationPlantaeLamialesLamiaceae

Chiov., Fl. Somala 1: 278. 1929

05337C56FC0C5830B41CB5F6EE851C7F

Plectranthus burorum (Chiov.) J.K.Morton, Novon 8: 265. 1998. Type: Somalia, Somalia Meridionale: Boscaglia di Bur Budulca, 16 Mar.1924, N.Puccioni & G. Stefanini 287 (323) (holotype: FT).
Coleus cicatricosus Hutch. & E.A.Bruce, Bull. Misc. Inform. Kew 1941: 180. 1941.
Plectranthus cicatricosus (Hutch. & E.A.Bruce) J.R.I.Wood, Handb. Yemen Fl.: 249. 1997. Type: Somalia, Buramo, 29 Jan. 1933, J.B.Gillett 4880 (holotype: K; isotype: FT).

##### Distribution.

Ethiopia to Somalia, Yemen.

#### Coleus caillei

Taxon classificationPlantaeLamialesLamiaceae

A.Chev. ex Hutch. & Dalziel, Fl. W. Trop. Afr. 2: 291. 1931

9E743C948B1658ED986CF651885063C1

Leocus caillei (A.Chev. ex Hutch. & Dalziel) J.K.Morton, J. Linn. Soc., Bot. 58: 270. 1962. Plectranthus caillei (A.Chev. ex Hutch. & Dalziel) B.J.Pollard, Kew Bull. 64: 260. 2009. Type: Guinea, Longuery, Caille in Herb Chevalier 14684 (holotype: P).

##### Distribution.

W. Trop. Africa to Cameroon.

#### Coleus calaminthoides

Taxon classificationPlantaeLamialesLamiaceae

(Baker) A.J.Paton comb. nov.

2655747311655931BC1ACF1D8C49BE2F

urn:lsid:ipni.org:names:77201030-1

Solenostemon calaminthoides Baker in D.Oliver & auct. suc. (eds.), Fl. Trop. Afr. 5: 421. 1900. Type: Gabon, Gabon River, Mann (holotype, K).

##### Distribution.

Cameroon, Gabon, DRC.

#### Coleus calcicola

Taxon classificationPlantaeLamialesLamiaceae

Murata, Acta Phytotax. Geobot. 24: 105. 1970

171C3785F94751A8B7866BAEECC24095

Plectranthus chiangdaoensis Suddee, Kew Bull. 59: 404. 2004., non Plectranthus calcicola Hand.-Mazz. Type: Thailand, Chiang Mai, Doi Chiang Dao, 1500 m, 15 Sept. 1967, Shimizu & Hutoh T-10153 (holotype: KYO; isotype: BKF, KYO, TI).

##### Distribution.

N. Thailand (Doi Chiang Dao).

#### Coleus caldericola

Taxon classificationPlantaeLamialesLamiaceae

(P.I.Forst.) P.I.Forst. comb. nov.

FA2AD3B2D94550C5A71EE4E0259C941A

urn:lsid:ipni.org:names:77201031-1

Plectranthus caldericola P.I.Forst., Austrobaileya 8: 392. 2011. Type: Australia, New South Wales, The Pinnacle, 13 km W of Mt. Warning, 6 Apr. 1994, P.I. Forster & G. Leiper PIF15093 (holotype: BRI; isotypes: MEL, NSW).

##### Distribution.

Australia: New South Wales.

#### Coleus calycinus

Taxon classificationPlantaeLamialesLamiaceae

(Benth.) A.J.Paton comb. nov.

39211FE122C652EDAF707C982DE5FC10

urn:lsid:ipni.org:names:77201032-1

Plectranthus calycinus Benth. in E.H.F.Meyer, Comm. Pl. Afr. Austr.: 230. 1838.
Rabdosia calycina (Benth.) Codd, Bothalia 11: 117. 1973.
Rabdosiella calycina (Benth.) Codd, Bothalia 15: 10. 1984.
Isodon calycinus (Benth.) H.W.Li, J. Arnold Arbor. 69: 293. 1988. Type: South Africa, Eastern Cape, between St Johns and Umsikaba rivers, 1837, Drège 3584 (lectotype: K designated by Codd (1975); isolectotypes: MO, P).
Plectranthus pyramidatus Gürke, Bull. Herb. Boissier 6: 552. 1898. Type: South Africa, Mpumalanga, Houtbosch, s.d., Rehmann 6179 (holotype: Z).
Plectranthus pachystachyus Briq., Bull. Herb. Boissier, sér. 2, 3: 1003. 1903.
Plectranthus calycinus var. pachystachyus (Briq.) T.Cooke in W.H.Harvey & auct. suc. (eds.), Fl. Cap. 5(1): 271. 1910. Type: South Africa, KwaZulu-Natal, Umkomaas, iii.1892, Medley Wood 4621 (holotype: G; isotype: K).

##### Distribution.

S. Mozambique to South Africa.

#### Coleus cambodianus

Taxon classificationPlantaeLamialesLamiaceae

(Murata) A.J.Paton comb. nov.

7100517EFCCE55ABBD49F5EA3E0342F4

urn:lsid:ipni.org:names:77201033-1

Anisochilus cambodianus Murata, Acta Phytotax. Geobot. 28: 26. 1977. Type: Cambodia, Kampot Province, Poporkvil, c. 1000 m alt, 4 Dec. 1964, Kira, Hozumi, Yoda & Kokawa 292 (holotype: KYO).

##### Distribution.

SE. Thailand to Cambodia.

#### Coleus caninus

Taxon classificationPlantaeLamialesLamiaceae

(Roth) Vatke, Linnaea 37: 318. 1872

55D51E895FC157F39EE833A49306C890

Plectranthus caninus Roth, Nov. Pl. Sp.: 279. 1821.
Majana canina (Roth) Kuntze, Revis. Gen. Pl. 2: 524. 1891. Type: India, Heyne s.n. in Wallich Cat. 2729C (holotype: B, destroyed; isotypes: BM, K(000820142), K-W (K001116966), P).

##### Distribution.

Eritrea to South Africa, India to Myanmar.

#### Coleus caninus subsp. caninus

Taxon classificationPlantaeLamialesLamiaceae

958378A0302C5130971E1B1C50E83921

Plectranthus caninus subsp. caninus , A.J.Paton, Fl. Trop. E. Afr., Lamiac.: 345. 2009. Type: as above.
Ocimum monadelphum R.Br. ex Roth, Nov. Pl. Sp.: 267. 1821.
Coleus heynei Benth., Labiat. Gen. Spec.: 50. 1832. Type: India, Heyne s.n. (holotype: B, destroyed; neotype,: India, Herb. Ham. in Wall. Cat. 2729B (neotype: K-W (K001116965), designated by Paton et al. 2009; isoneotype: K).
Coleus spicatus Benth. in N.Wallich, Pl. Asiat. Rar. 2: 15. 1830.
Majana spicata (Benth.) Kuntze, Revis. Gen. Pl. 2: 524. 1891. Type: India, Herb. Wight in Wall. Cat. 2729A (syntypes: BM, K (K000820139), K (K000820141), K-W(K001116964), G-DC).
Plectranthus monadelphus Roxb., Fl. Ind. ed. 1832, 3: 22. 1832. Type: Roxburgh’s illustration No. 1459 (lectotype: K, designated by Suddee et al. 2004).
Coleus spicatus var. rondinella Spreng., Gartenflora 45: 358. fig. 62. 1896. Type: cultivated, material from Eritrea, illustration cited above (lectotype: illustration designated by Paton et al. 2009).
Coleus pachyphyllus Gürke, Bot. Jahrb. Syst. 38: 168. 1906. Types: Ethiopia, Galla Highlands, Ginea, Ellenbeck 1951 (syntype: B, destroyed) & Korkora in Borana, Ellenbeck 2233 (syntype: B, destroyed); neotype: Somalia, Juba R. north of Gelib, Mooney 7672 (neotype: K, designated by Ryding (2000)).

##### Distribution.

Eritrea to N. Tanzania, India to Myanmar.

#### Coleus caninus subsp. flavovirens

Taxon classificationPlantaeLamialesLamiaceae

 (Gürke) A.J.Paton comb. nov.

6EB6C1FBF7A054119ED4E89C2F3734E1

urn:lsid:ipni.org:names:77201034-1

Coleus flavovirens Gürke in H.G.A.Engler, Pflanzenw. Ost-Afrikas, C: 347. 1895.
Plectranthus caninus subsp. flavovirens (Gürke) A.J.Paton, Fl. Trop. E. Afr., Lamiac.: 345. 2009. Type: Tanzania, Lake Chala (Dschallasee), 1893, Volkens 1771 (holotype: B destroyed; isotype: BR).
Coleus omahekensis Dinter, Repert. Spec. Nov. Regni Veg. Beih. 53: 123. 1928. Type: Namibia, Etemba and Otjikuara, Dinter 3265, two localities same number (syntypes: not seen.).

##### Distribution.

Ethiopia to South Africa.

#### Coleus capitatus

Taxon classificationPlantaeLamialesLamiaceae

A.J.Paton nom. nov.

A3BCA52620A65CADBDACB3FF7E66C57C

urn:lsid:ipni.org:names:77201035-1

Pycnostachys parvifolia Baker, Bull. Misc. Inform. Kew 1895: 72. 1895., non Coleus parviflorus Benth. Type: Zambia, Northern Province, Fwambo, Carson 103 (lectotype: K), designated by Bruce (1940).

##### Distribution.

W. & S. Tanzania to Zambia.

#### Coleus carnosifolius

Taxon classificationPlantaeLamialesLamiaceae

(Hemsl.) Dunn, Notes Roy. Bot. Gard. Edinburgh 8: 158. 1913

FD068B26C6F2543884C10261F88E70D4

Plectranthus carnosifolius Hemsl., J. Linn. Soc., Bot. 26: 270. 1890. Type: China, Guangdong Prov., Aug. 1887, C. Ford 107 (holotype: K).

##### Distribution.

SE. China.

#### Coleus cataractarum

Taxon classificationPlantaeLamialesLamiaceae

(B.J.Pollard) A.J.Paton comb. nov.

096AB5E0BA9751D090F7A459D5706E1F

urn:lsid:ipni.org:names:77201036-1

Plectranthus cataractarum B.J.Pollard, Kew Bull. 56: 976. 2001. Type: Mt Cameroon, 25 Nov. 1993, Cheek 5563 (holotype: K; isotypes MA, MO, SCA, WAG, YA).

##### Distribution.

Bioko, Cameroon.

#### Coleus caudatus

Taxon classificationPlantaeLamialesLamiaceae

(S.Moore) E.Downes & I.Darbysh. Blumea 62: 172. 2017

4A53C8CD57C55A09B85771BE3652AD43

Plectranthus caudatus S.Moore, J. Linn. Soc., Bot. 40: 176. 1911. Type: Zimbabwe, Chimanimani, 26 Nov. 1906, Swynnerton 2010 (holotype: BM; isotype: K).

##### Distribution.

Mozambique and Zimbabwe.

#### Coleus celsus

Taxon classificationPlantaeLamialesLamiaceae

A.J.Paton nom. nov.

A6E315F9B4D2554B9C721EC9A3A18129

urn:lsid:ipni.org:names:77201037-1

Solenostemon robustus Hiern, Cat. Afr. Pl. 1: 864. 1900., non Coleus robustus (Hook.f.) A.J.Paton.
Holostylon robustum (Hiern) G.Taylor, J. Bot. 69(suppl. 2): 161. 1931.
Plectranthus robustus (Hiern) A.J.Paton in Fl. Zambesiaca 8,8: 234. 2013. Type: Angola, Pungo Andongo, from Lombe to Condo, March 1857, Welwitsch 5538 (holotype: LISU; isotypes: BM, G).

##### Distribution.

Angola.

#### Coleus centraliafricanus

Taxon classificationPlantaeLamialesLamiaceae

A.J.Paton nom. nov.

4832340EA6655E74900C3F2D05D26E65

urn:lsid:ipni.org:names:77201038-1

Pycnostachys chevalieri Briq., Mém. Soc. Bot. France 8: 193. 1912., non Coleus chevalieri Briq. Type: Central African Republic, Dar Banda du Kaga Djé au Kaga Pongouru, A.Chevalier 6561 (holotype: P).

##### Distribution.

Central African Republic.

#### Coleus chevalieri

Taxon classificationPlantaeLamialesLamiaceae

Briq., Mém. Soc. Bot. France 8: 287. 1917

DBFD322069A856E78E69DEBABF1F63CC

Coleus chevalieri Briq., Mém. Soc. Bot. France 8: 287. 1917. Type: Central African Republic, Krebedjén (Fort Sibut), vallée de la moyenne Tomi, 8 Oct.1902, Chevalier 5662 (holotype: P).

##### Distribution.

Central African Republic.

#### Coleus ciliatus

Taxon classificationPlantaeLamialesLamiaceae

(Bramley) A.J.Paton comb. nov.

58CE1E4E8F935B5593888B58D9DE7383

urn:lsid:ipni.org:names:77201039-1

Pycnostachys ciliata Bramley, Kew Bull. 60: 587. 2005 publ. 2006. Type: Tanzania, Mbeya Dist., Songwe Valley, c. 2 km N of Mbeya–Tunduma road, fl. 25 Mar, 1988, Bidgood, Mwasumbi & Vollesen 693 (holotype: K; isotypes: C, DSM, NHT).

##### Distribution.

Tanzania to S. Trop. Africa.

#### Coleus circinnatus

Taxon classificationPlantaeLamialesLamiaceae

(Hedge) A.J.Paton & Phillipson comb. nov.

DE971F71FBE15E4A91AD3565270FC902

urn:lsid:ipni.org:names:77201041-1

Plectranthus circinnatus Hedge, Fl. Madag. 175: 228. 1998. Type: Madagascar, Prov. de Fianarantsoa, Massive d’Itremo, sur RN 35, 42 Km W. of Ambatofinandrahana, Clement, Phillipson & Rafamantanantsoa 2045 (holotype: E; isotypes MO, P).

##### Distribution.

Madagascar.

#### Coleus coeruleus

Taxon classificationPlantaeLamialesLamiaceae

Gürke, Bot. Jahrb. Syst. 19: 217. 1894

480C6863ABF7585AB9F77AE5E80DCE78

Calchas coeruleus (Gürke) P.V.Heath, Calyx 5: 160. 1997.
Plectranthus coeruleus (Gürke) Agnew, Upland Kenya Wild Fl.: 638. 1974. Type: Tanzania, Lushoto District: Kwa Mshusa, Holst 8895 (holotype: B, destroyed; isotypes: G, K, Z).

##### Distribution.

Kenya to N. Malawi.

#### Coleus comosus

Taxon classificationPlantaeLamialesLamiaceae

Hochst. ex Gürke, Bot. Jahrb. Syst. 19: 212. 1894

8F58D936CAE8524DABAE12638E0AD341

Plectranthus ornatus Codd, Bothalia 11: 393. 1975., non Plectranthus comosus Sims. Type: Ethiopia, Dschenausa, 1 June 1840, Schimper 1328 (holotype: B, destroyed; isotypes: BM, FI, G, P).

##### Distribution.

NE and E. Africa, naturalized in South Africa.

#### Coleus confertiflorus

Taxon classificationPlantaeLamialesLamiaceae

(A.J.Paton) A.J.Paton comb. nov.

142FF14BAF895192A6E094584FF3272F

urn:lsid:ipni.org:names:77201042-1

Plectranthus confertiflorus A.J.Paton, Fl. Trop. E. Afr., Lamiac.: 303. 2009. Type: Tanzania, Pare District: Mkomazi Game Reserve, Kisima Hill, Abdallah, Mboya & Vollesen 96/129 (holotype: K; isotypes: C, NHT, P).

##### Distribution.

N. Tanzania.

#### Coleus congensis

Taxon classificationPlantaeLamialesLamiaceae

(Gürke) A.J.Paton comb. nov.

F281CE61DB5056AA87A4F650517DB8C7

urn:lsid:ipni.org:names:77201043-1

Pycnostachys congensis Gürke, Bull. Herb. Boissier 4: 819. 1896. Types: DRC, Samba, Deschamps 28 (syntype: B); Lusambo (syntype: B).

##### Distribution.

DRC, Zambia.

#### Coleus congestus

Taxon classificationPlantaeLamialesLamiaceae

(R.Br.) A.J.Paton comb. nov.

A91F48E4D45C5789956E59247975B8B6

urn:lsid:ipni.org:names:77201044-1

Plectranthus congestus R.Br., Prodr. Fl. Nov. Holl.: 506. 1810. Type: Australia, Queensland, Endeavour River, 1770, Banks & Solander (holotype: BM).

##### Distribution.

N. Australia, Lesser Sunda Is., New Guinea.

#### Coleus conglomeratus

Taxon classificationPlantaeLamialesLamiaceae

(T.C.E.Fr.) Robyns & Lebrun, Ann. Soc. Sci. Bruxelles, Sér. B 49: 105. 1929

2AF18428B8555321B9D5F218CB76A0A5

Englerastrum conglomeratum T.C.E.Fr., Notizbl. Bot. Gart. Berlin-Dahlem 9: 72. 1924.
Plectranthus conglomeratus (T.C.E.Fr.) Hutch. & Dandy, Bull. Misc. Inform. Kew 1926:481. 1926. Type: Togo (Benin), Sokode, Kersting 93 (holotype: B, destroyed; fragment K).
Plectranthus harrisii J.K.Morton, J. Linn. Soc., Bot. 58: 267. 1962. Type: Ghana, between Toga Mt. and Maliato, Togo Plateau, 24 Dec. 1959, J.K.Morton A3830 (holotype: GC; isotypes: K, WAG). synon. nov.

##### Distribution.

Tropical West Africa.

#### Coleus crassus

Taxon classificationPlantaeLamialesLamiaceae

(N.E.Br.) Culham comb. nov.

588D33AAB9C356D08B1C5D7BBF8D0655

urn:lsid:ipni.org:names:77201045-1

Plectranthus crassus N.E.Br., Gard. Chron., ser. 3, 35: 21. 1904. Type: Plant cultivated at Kew in 1899 from material collected by Mahon 393 (holotype: K).

##### Distribution.

Malawi (Mt. Mulanje).

#### Coleus cremnus

Taxon classificationPlantaeLamialesLamiaceae

(B.J.Conn) A.J.Paton comb. nov.

49BA082BC63B518F9A2030F13BD71A6D

urn:lsid:ipni.org:names:77201046-1

Plectranthus cremnus B.J.Conn, Telopea 4: 643. 1992. Type: Australia, New South Wales, Sawtell, 20 Feb. 1990, B.J. Conn 3478 (holotype: NSW; isotype: MEL).

##### Distribution.

Australia, New South Wales.

#### Coleus cucullatus

Taxon classificationPlantaeLamialesLamiaceae

(A.J.Paton) A.J.Paton comb. nov.

0AB5EC2C9B3353BBA188E5AAAA24BB49

urn:lsid:ipni.org:names:77201047-1

Plectranthus cucullatus A.J.Paton – Fl. Zambes. 8(8): 261. 2013. Type: Mozambique, Ribáuè Dist., Ribáuè mountains, Oct. 1931, Gomes & Sousa 754 (holotype: K).

##### Distribution.

Mozambique.

#### Coleus cuneatus

Taxon classificationPlantaeLamialesLamiaceae

Baker f., J. Bot. 1899: 64. 1899

85A96E683BE151D7A1C97FB0F530C5D2

Plectranthus cuneatus (Baker f.) Ryding, Kew Bull. 54: 124. 1999. Type: Somalia, Woqooyi Galbeed/ Togdher Regions, Wagga Mt, Lort-Phillips s.n. (holotype: BM).

##### Distribution.

S. Djibouti to N. Somalia.

#### Coleus cyanophyllus

Taxon classificationPlantaeLamialesLamiaceae

(P.I.Forst.) P.I.Forst. comb. nov.

D64AFEB8C2C553FD97B40976CD9C48EE

urn:lsid:ipni.org:names:77201048-1

Plectranthus cyanophyllus P.I.Forst., Austrobaileya 4: 166. 1994. Type: Australia, Queensland, North Kennedy District. Mt Abbot, 50 km W of Bowen, 27 Oct. 1992, A.R.Bean 5188 (holotype: BRI; isotypes: CNS, K, MEL).

##### Distribution.

Australia: Queensland.

#### Coleus cylindraceus

Taxon classificationPlantaeLamialesLamiaceae

(Hochst. ex Benth.) A.J.Paton comb. nov.

4972B3B7929350BCB4A7E284BD43A776

urn:lsid:ipni.org:names:77201050-1

Plectranthus cylindraceus Hochst. ex Benth. in A.P.de Candolle, Prodr. 12: 60. 1848.
Germanea cylindracea (Hochst. ex Benth.) Hiern, Cat. Afr. Pl. 1: 861. 1900.
Burnatastrum cylindraceum (Hochst. ex Benth.) P.V.Heath, Calyx 4: 175. 2001. Type: Ethiopia, near Gapdia, 29 Nov.1838, Schimper 1113 (holotype: K; isotypes: BM, G, P,W).
Plectranthus montanus Benth. in N.Wallich, Pl. Asiat. Rar. 2: 17. 1830., non Coleus montanus Hochst. ex Ces. Type: India, Deccan Peninsula, exact locality unknown (“Peninsula India Orientalis”), Herb. Wight in Wall. Cat. 2747B (lectotype: K ((K000820120), designated by Suddee et al. (2004); isolectotype: K-W (K001117007).
Plectranthus marrubioides Hochst. ex Benth. in A.P.de Candolle, Prodr. 12: 60. 1848. Type: Ethiopia, near Jaja (Dschadscha), Jan. 1844, Schimper 1925 (holotype: K; isotypes: BM,G, K, P, W).
Geniosporum lasiostachyum Briq., Bot. Jahrb. Syst. 19: 164. 1894. Type: Angola, Huíla, Mar. 1879, Welwitsch 5489 (syntypes: K, BM, LISC).
Plectranthus fischeri Gürke, Bot. Jahrb. Syst. 19: 200. 1894. Types: Tanzania, Kilimanjaro (Kilimandcharo), Ugweno, Volkens 518 (syntype: B, destroyed); Masai Highlands (Massaihochland), Fischer ser.1 77 (syntype: B, destroyed; isosyntype: HBG) & Fischer ser.2 501 (syntype: B, destroyed).
Plectranthus moschosmoides Baker in D.Oliver & auct. suc. (eds.), Fl. Trop. Afr. 5: 414. 1900. Type: Angola, Huíla, Mar.1879, Welwitsch 5489 (holotype: BM; isotypes: K, LISC).
Geniosporum fissum S.Moore, J. Bot. 39: 263. 1901. Type: Kenya, Northern Frontier District: Dadaro, 1110 m, Delamere s.n. (holotype: BM).
Plectranthus densiflorus T.Cooke, Bull. Misc. Inform. Kew 1909: 378. 1909. Type: South Africa, KwaZulu-Natal, near the Mooi R., Medley-Wood 4475 (holotype: K; isotypes: GRA, NH, SAM).
Plectranthus villosus T.Cooke, Bull. Misc. Inform. Kew 1909: 378. 1909. Type: South Africa, KwaZulu-Natal, Entumeni, Medley-Wood 3955 (holotype: K; isotype NH).
Plectranthus glomeratus R.A.Dyer, Fl. Pl. South Africa 24: t. 946. 1944. nom. superfl. Based on the above.
Plectranthus spiciformis R.A.Dyer, Fl. Pl. South Africa 24: t. 946. 1944. Type: South Africa, Gauteng, Hammanskraal, Mogg 27138 (holotype: PRE).
Coleus subbaraoi Kumari & Malathi, Fl. Visakhapatnam Distr. 2: 68. 2008. Type: India, Andhra Pradesh, Visakhapatnam District, Kappakonda, Subba Rao 29637 (holotype: CAL; isotype: MH).

##### Distribution.

Trop. & South Africa, Arabian Peninsula, S. India

#### Coleus daviesii

Taxon classificationPlantaeLamialesLamiaceae

E.A.Bruce, Bull. Misc. Inform. Kew 1933: 477. 1933

B9746B8F1DA75C3DA78A8C0E97120CE6

Plectranthus daviesii (E.A.Bruce) B.Mathew, Kew Bull. 31: 174. 1976. Type: Tanzania, Rungwe, R.M. Davies R4 (holotype: K; isotype: SRGH).

##### Distribution.

SW. Tanzania to N. Malawi.

#### Coleus decimus

Taxon classificationPlantaeLamialesLamiaceae

(A.J.Paton) A.J.Paton comb. nov.

B506598FF6335B12847D055381077A5A

urn:lsid:ipni.org:names:77201051-1

Plectranthus decimus A.J.Paton, Fl. Trop. E. Afr., Lamiac.: 324. 2009. Type: Zambia, Mbala (Abercorn) District: Kawimbe, Richards 7288 (holotype: K).

##### Distribution.

W. Tanzania, Zambia to Angola.

#### Coleus decurrens

Taxon classificationPlantaeLamialesLamiaceae

Gürke, Bot. Jahrb. Syst. 19: 215. 1894

33E9FFC428FE524380A115BD22BABE7C

Plectranthus decurrens (Gürke) J.K.Morton, J. Linn. Soc., Bot. 58: 267. 1962. Type: Cameroon, Buea, Preuss 948 (holotype: B, destroyed; isotypes: COI, HBG).
Coleus elatus Baker in D.Oliver & auct. suc. (eds.), Fl. Trop. Afr. 5: 427. 1900. Types: Bioko (Fernando Po), Mann 584 (syntype: K); Gabon, Crystal Mts, Mann s.n. 1661 (synype: K).
Coleus toroensis S.Moore, J. Bot. 45: 95. 1907. Type: Uganda, Toro District: Dura (Durro) R., Bagshawe 1043 (holotype: BM).
Coleus variifolius De Wild., Bol. Soc. Ibér. Ci. Nat. 19: 124. 1920. Type: DRC, between Buta and Bima, Seret 96 (lectotype: BR, designated by Paton et al. 2009).

##### Distribution.

Nigeria to Uganda and Angola.

#### Coleus deflexifolius

Taxon classificationPlantaeLamialesLamiaceae

(Baker) A.J.Paton comb. nov.

B85CAB4059A85AD8B2FD0E2F9AB613DB

urn:lsid:ipni.org:names:77201052-1

Pycnostachys deflexifolia Baker in D.Oliver & auct. suc. (eds.), Fl. Trop. Afr. 5: 381. 1900. Type: Kenya, Naivasha District: near Lake Elmenteita, Scott Elliot 6756 (holoype: K).

##### Distribution.

E. Trop. Africa.

#### Coleus defoliatus

Taxon classificationPlantaeLamialesLamiaceae

(Hochst. ex Benth.) A.J.Paton comb. nov.

320AF1016F115895952D72C326A890AB

urn:lsid:ipni.org:names:77201053-1

Plectranthus defoliatus Hochst. ex Benth. in A.P.de Candolle, Prodr. 12: 60. 1848.
Isodictyophorus defoliatus (Hochst. ex Benth.) Agnew, Upland Kenya Wild Fl.: 640. 1974. Type: Ethiopia, Jomara (Dschomara), 26 Dec. 1839, Schimper 847 (holotype: K; isotypes BR, FI, G, MPU, TUB, P, W).
Coleus floribundus Baker in D.Oliver & auct. suc. (eds.), Fl. Trop. Afr. 5: 438. 1900. Type: Ethiopia, 1 Jan. 1853, Schimper 21 (holotype: K; isotype: BR, G).

##### Distribution.

Eritrea to S. Trop. Africa.

#### Coleus densus

Taxon classificationPlantaeLamialesLamiaceae

(N.E.Br.) A.J.Paton comb. nov.

7A7C9B98C00653639F74EEA8F02506D1

urn:lsid:ipni.org:names:77201054-1

Plectranthus densus N.E.Br., Bull. Misc. Inform. Kew 1894: 12. 1894. Type: Tanzania, N of Lake Malawi (Nyassa), Thomson s.n. (holotype: K).
Plectranthus primulinus Baker, Bull. Misc. Inform. Kew 1895: 292. 1895.
Coleus primulinus (Baker) A.Chev., Rev. Int. Bot. Appl. Agric. Trop. 33: 340. 1953. Type: Zambia, Mwero Plateau, 1894, Carson 36 (holotype: K).

##### Distribution.

SW. Tanzania to S. Trop. Africa.

#### Coleus descampsii

Taxon classificationPlantaeLamialesLamiaceae

(Briq.) A.J.Paton comb. nov.

B105996976755454A4E8A7A1B2F68656

urn:lsid:ipni.org:names:77201055-1

Pycnostachys descampsii Briq., Bull. Soc. Roy. Bot. Belgique 37: 63. 1899. Type: DRC, Katanga, Lufonzo (Lufogo) R., Mar. 1896, Descamps s.n. (holotype: BR).
Pycnostachys linifolia Gürke, Bot. Jahrb. Syst. 30: 397. 1901. Type: Tanzania, Njombe District: Ubena, near Liangira, Goetze 798 (holotype: B, destroyed; isotypes: BR, K, fragment).
Pycnostachys lavanduloides Perkins, Notizbl. Bot. Gart. Berlin-Dahlem 8: 68. 1921. Type: Tanzania, Iringa District, Uhehe, Udzungwa Mts (Utschungwe-Berge), 1600 m, Prince 28a (holotype: B, destroyed).
Pycnostachys pallide-caerulea Perkins, Notizbl. Bot. Gart. Berlin-Dahlem 8: 67. 1921. Type: Cameroon, Kumbo, Mt Bansso, Oct. 1909, Ledermann 5738 (holotype: B, destroyed, fragment K).

##### Distribution.

Cameroon to W. Tanzania.

#### Coleus dewildemanianus

Taxon classificationPlantaeLamialesLamiaceae

(Robyns & Lebrun) A.J.Paton comb. nov.

470CABD8BA515CC192EAA589717BF9BE

urn:lsid:ipni.org:names:77201057-1

Pycnostachys dewildemaniana Robyns & Lebrun, Rev. Zool. Bot. Africaines 16: 352. 1928. Type: DRC, Katanga, Munama, Quarré 1143 (holotype: BR; isotype: K).

##### Distribution.

Tanzania to S. Trop. Africa.

#### Coleus dichotomus

Taxon classificationPlantaeLamialesLamiaceae

(A.J.Paton) A.J.Paton comb. nov.

8B19A58B077959BA87D510E03113B1A4

urn:lsid:ipni.org:names:77201058-1

Plectranthus dichotomus A.J.Paton, Fl. Trop. E. Afr., Lamiac.: 317. 2009. Type: Tanzania, Kilosa District: Ukaguru, Mamiwa Forest Reserve, E of Ikwamba peak, Mabberley 1465 (holotype: K).

##### Distribution.

Tanzania (Ukaguru Mts.).

#### Coleus dinteri

Taxon classificationPlantaeLamialesLamiaceae

(Briq.) A.J.Paton comb. nov.

B4E95EB2E5005CC8BB06A538552B2E54

urn:lsid:ipni.org:names:77201059-1

Plectranthus dinteri Briq., Bull. Herb. Boissier, sér. 2, 3: 1070. 1903. Type: Namibia, Waterberg, Dinter 336 (holotype: Z).

##### Distribution.

Namibia, South Africa: Limpopo (W. Waterberg).

#### Coleus dissitiflorus

Taxon classificationPlantaeLamialesLamiaceae

Gürke, Bot. Jahrb. Syst. 19: 217. 1894

8D26032E0593504B9B3CF9AFF0DC62BE

Plectranthus dissitiflorus (Gürke) J.K.Morton, J. Linn. Soc., Bot. 58: 267. 1962. Type: Cameroon, between Buea and Mimbia, 9 Oct. 1891, Preuss 1055 (holotype: B, destroyed; isotype: COI).

##### Distribution.

WC. Trop. Africa.

#### Coleus divaricatus

Taxon classificationPlantaeLamialesLamiaceae

A.J.Paton nom. nov.

CECF71B11E535E7AABD1524ECC52FB90

urn:lsid:ipni.org:names:77201060-1

Anisochilus paniculatus Benth. in A.P.de Candolle, Prodr. 12: 82. 1848, non Coleus paniculatus Benth. Type: Sri Lanka, unknown locality, Walker 52 (holotype: K).

##### Distribution.

S. India.

#### Coleus diversus

Taxon classificationPlantaeLamialesLamiaceae

(S.T.Blake) P.I.Forst. & T.C.Wilson comb. nov.

A2CECD00A26B539184FE9DA43E61EFF9

urn:lsid:ipni.org:names:77201061-1

Plectranthus diversus S.T.Blake, Contr. Queensland Herb. 9: 20. 1971. Type: Australia, Queensland, Spring Creek near Bowen (cultivated at The Gap), Brisbane., Oct. 1963, S.T.Blake 22128 (holotype: BRI).

##### Distribution.

Australia: Queensland.

#### Coleus dolichopodus

Taxon classificationPlantaeLamialesLamiaceae

(Briq.) A.J.Paton comb. nov.

208B4B0C67DC592CBA4442A9BE09CC02

urn:lsid:ipni.org:names:77201062-1

Plectranthus dolichopodus Briq., Bull. Herb. Boissier, sér. 2, 3: 1069. 1903. Type: South Africa, KwaZulu-Natal, Karkloof, n.d., Rehmann 7383 (holotype: Z).

##### Distribution.

Malawi to South Africa.

#### Coleus dumicola

Taxon classificationPlantaeLamialesLamiaceae

(P.I.Forst.) P.I.Forst. comb. nov.

2E1E5F9C0A2E5BB59A06BDDA07AA1B71

urn:lsid:ipni.org:names:77201063-1

Plectranthus dumicola P.I.Forst., Austrobaileya 4: 168. 1994. Type: Australia, Queensland, Cook District, 31 km along road to Leo Creek Mine, McIlwraith Range, 8 June 1992, P.I.Forster PIF10268 & M.C.Tucker & G. Kenning (holotype: BRI; isotypes: CNS, K, MEL).

##### Distribution.

Australia: Queensland.

#### Coleus dysophylloides

Taxon classificationPlantaeLamialesLamiaceae

(Benth.) A.J.Paton comb. nov.

7FC25DFE906C582BA735FECB2B029B9E

urn:lsid:ipni.org:names:77201064-1

Anisochilus dysophylloides Benth. in N.Wallich, Pl. Asiat. Rar. 2: 19. 1830. Type: India, Madras, Nilghiri Hills, Herb. Wight in Wall. Cat. 2756 (lectotype: K ((K000674673)); isolectotypes: E, G-DC,K-W(K001117033), designated Suddee and Paton 2009).
Anisochilus sericeus Benth. in A.P.de Candolle, Prodr. 12: 82. 1848. Type: India, Deccan Peninsula, Courtallum, Herb. Wight 2515 (holotype: K: (K000674767)); isotype K (K000674768).
Anisochilus albidus Wight, Icon. Pl. Ind. Orient. 4: t. 1436. 1849. Type: Madras, Nilghiri Hills, About Coonoor and Kaitie, ex Herb. Wight, Wight Ic. t. 1436 (holotype: K (K000674776)).
Anisochilus purpureus Wight, Icon. Pl. Ind. Orient. 4: t. 1435. 1849.
Anisochilus dysophylloides var. purpureus (Wight) Gamble, Fl. Madras 2: 1128. 1924. Type: Madras, Nilghiri Hills, About Coonoor, Ex Herb. Wight Propr., Wight Ic. t. 1435 (holotype: K (K000674774)).

##### Distribution.

SW. India (Shervarayan Hills).

#### Coleus efoliatus

Taxon classificationPlantaeLamialesLamiaceae

De Wild., Contr. Fl. Katanga: 173. 1921

CDE32CD6DE0750C6AF6CD4C40C2556EA

Plectranthus efoliatus (De Wild.) A.J.Paton, Fl. Trop. E. Afr., Lamiac.: 289. 2009. Type: DRC, Welgelegen, Bequaert 486 (lectotype: BR, designated by Paton et al. (2009)).
Plectranthus leviculus N.E.Br., Bull. Misc. Inform. Kew 1921: 296. 1921. Type: DRC, Lubumbashi (Elisabethville), F.A. Rogers 26211 (holotype: K).
Englerastrum hjalmarii T.C.E.Fr., Notizbl. Bot. Gart. Berlin-Dahlem 9: 71. 1924.
Plectranthus hjalmarii (T.C.E.Fr.) Hutch. & Dandy, Bull. Misc. Inform. Kew 1926: 481. 1926.
Coleus hjalmarii (T.C.E.Fr.) Robyns & Lebrun, Ann. Soc. Sci. Bruxelles, Sér. B 49: 106. 1929. Type: Tanzania, Rungwe District: Kyimbila, Stoltz 926 (holotype: B, destroyed; isotypes: G, K, W).
Englerastrum kassneri T.C.E.Fr., Notizbl. Bot. Gart. Berlin-Dahlem 9: 70. 1924.
Plectranthus kassneri (T.C.E.Fr.) Hutch. & Dandy, Bull. Misc. Inform. Kew 1926: 481. 1926.
Coleus kassneri (T.C.E.Fr.) Robyns & Lebrun, Ann. Soc. Sci. Bruxelles, Sér. B 49: 106. 1929. Type: DRC, Mt Morumbe, Kassner 2951 (holotype: B, destroyed; isotypes: BM, K).

##### Distribution.

Rwanda to S. Trop. Africa.

#### Coleus elliotii

Taxon classificationPlantaeLamialesLamiaceae

(S.Moore) A.J.Paton comb. nov.

61AA789513C75193B6BB911AC5F4BAC5

urn:lsid:ipni.org:names:77201065-1

Pycnostachys elliotii S.Moore, J. Linn. Soc., Bot. 38: 275. 1908. Type: Uganda, Ruwenzori E, 2750 m, 10 Feb. 1906, Wollaston s.n. (holotype: BM).
Pycnostachys mildbraedii Perkins in G.W.J.Mildbraed (ed.), Wiss. Erg. Deut. Zentr.-Afr. Exped., Bot. 2: 548. 1913. Type: Uganda, Toro District/DRC, Ruwenzori, Butahu (Butagu) Valley, Friedrich 2537 (holotype: B, destroyed).
Pycnostachys cinerascens Robyns & Lebrun, Rev. Zool. Bot. Africaines 16: 352. 1928.
Pycnostachys bequaertii De Wild., Pl. Bequaert. 4: 393. 1928., nom. illeg., non Pycnostachys bequaertii De Wild. (1921). Type: DRC, Ruwenzori Lamia Valley, Bequaert 4287 (holotype: BR).
Pycnostachys butaguensis De Wild., Pl. Bequaert. 4: 389. 1928. Type: DRC, Ruwenzori, Butahu (Butagu) Valley, Bequaert 3715 (holotype: BR).

##### Distribution.

DRC to Uganda (Ruwenzori Mts.).

#### Coleus elongatus

Taxon classificationPlantaeLamialesLamiaceae

Trimen, J. Bot. 27: 165. 1889

0E8205F5EE6B5AA4BAA35D61E6FAAE80

Englerastrum elongatum (Trimen) Alston, Bull. Misc. Inform. Kew 1926: 298. 1926.
Plectranthus elongatus (Trimen) R.H.Willemse, Blumea 25: 510. 1979. Type: Sri Lanka, Ritgala, July 1887, Trimen s.n. (holotype: PDA; isotype: K).

##### Distribution.

Sri Lanka.

#### Coleus eminii

Taxon classificationPlantaeLamialesLamiaceae

(Gürke) A.J.Paton comb. nov.

8B7A91141F3B525A9B7A9D9249301998

urn:lsid:ipni.org:names:77201066-1

Pycnostachys eminii Gürke, Bot. Jahrb. Syst. 22: 145. 1895. Type: Tanzania, Kanessa, west of Lake Victoria, Kanessa, 14 Nov. 1890, Stuhlmann 943 (lectotype: B, destroyed, designated by Bruce (1940).
Pycnostachys ruwenzoriensis Baker in D.Oliver & auct. suc. (eds.), Fl. Trop. Afr. 5: 384. 1900. Type: Uganda, Ruwenzori, Scott Elliot 7621 (holotype: K).
Pycnostachys rotundatodentata De Wild., Pl. Bequaert. 4: 391. 1928. Type: DRC, Ruwenzori, Kisuki, Bequaert 4701 (lectotype: BR, designated by Bramley in Paton et al. (2009)).

##### Distribution.

Cameroon to Ethiopia and NW. Tanzania.

#### Coleus engleri

Taxon classificationPlantaeLamialesLamiaceae

(Briq.) A.J.Paton comb. nov.

26F6E93B029656428668DCF0EBB27EDD

urn:lsid:ipni.org:names:77201067-1

Anisochilus engleri Briq., Bot. Jahrb. Syst. 19: 190. 1894. Type: DRC, between Nyangwe and Kibundo, Pogge 1019 (lectotype: K, designated by Paton et al. (2009)).
Anisochilus africanus Baker, J. Linn. Soc., Bot. 30: 94. 1894., non Coleus africanus Benth.
Briquetastrum africanum (Baker) Robyns & Lebrun, Ann. Soc. Sci. Bruxelles, Sér. B 49: 102. 1929.
Coleus africanus (Baker) Roberty, Bull. Inst. Fondam. Afrique Noire, Sér. A, Sci. Nat. 16: 330. 1954. nom. illeg.
Leocus africanus (Baker) J.K.Morton, J. Linn. Soc., Bot. 58: 270. 1962.
Plectranthus africanus (Baker) A.J.Paton, Fl. Trop. E. Afr., Lamiac.: 308. 2009. Type: Sierra Leone, Freetown, Scott Elliot 5033 (holotype: K).

##### Distribution.

W. Trop. Africa to Uganda and NE. Angola.

##### Notes.

*Coleus africanus* Benth., Labiat. Gen. Spec.: 54. 1832., is superfluous being based on *Solenostemon ocymoides* Schumach. & Thonn. in C.F.Schumacher, Beskr. Guin. Pl.: 271 (1827).

#### Coleus erici-rosenii

Taxon classificationPlantaeLamialesLamiaceae

(R.E.Fr.) A.J.Paton comb. nov.

61DF0748851E5A3FB7BE2E398836BC3A

urn:lsid:ipni.org:names:77201068-1

Pycnostachys erici-rosenii R.E.Fr., Wiss. Erg. Schwed. Rhod.-Kongo Exped. 1: 281. 1916. Type: DRC, Ninagongo, 2000 m, R.E. Fries 1588 (holotype: UPS).
Pycnostachys albidoviolacea De Wild., Pl. Bequaert. 4: 400. 1928. Type: DRC, Kivu, Mukule, Bequaert 5889 (lectotype: BR, designated by Bramley in Paton et al. (2009)).
Pycnostachys robynsii De Wild., Pl. Bequaert. 4: 398. 1928. Type: DRC/Burundi, Busiga, Robyns 2356 (holotype: BR; isotype: K).

##### Distribution.

E. DRC to Uganda.

#### Coleus esculentus

Taxon classificationPlantaeLamialesLamiaceae

(N.E.Br.) G.Taylor, J. Bot. 69 (suppl. 2): 158. 1931

54E36E9A093D5EE08E793F6A484BAE6F

Plectranthus esculentus N.E.Br., Bull. Misc. Inform. Kew 1894: 12. 1894. Type: cultivated at K from material sent by J. Medley Wood from KwaZulu-Natal, 1893 (lectotype: K, designated by Codd (1975); isolectotype: BOL).
Plectranthus floribundus N.E.Br., Bull. Misc. Inform. Kew 1894: 12. 1894.
Englerastrum floribundum (N.E.Br.) T.C.E.Fr., Notizbl. Bot. Gart. Berlin-Dahlem 9: 73. 1924.
Coleus floribundus (N.E.Br.) Robyns & Lebrun, Rev. Zool. Bot. Africaines 16: 359. 1928. nom. illeg., non C. floribundus Baker. Type: South Africa, KwaZulu-Natal, Inanda, Medley Wood 646 (lectotype: K; isolectotype: PRE designated by Robyns & Lebrun (1928)).
Plectranthus floribundus var. longipes N.E.Br., Bull. Misc. Inform. Kew 1894: 13. 1894.
Coleus floribundus var. longipes (N.E.Br.) Robyns & Lebrun, Rev. Zool. Bot. Africaines 16: 360. 1928. Type: South Africa, valley of Umzingwani R., Baines s.n. (lectotype: K, designated by Robyns & Lebrun (1928)).
Coleus coppinii Heckel, Rev. Cultures Colon. 8: 166. 1900. Type and protologue not seen.
Coleus dazo A.Chev., Agric. Prat. Pays Chauds 4: 104. 1905. Type: tubers from Central African Republic, upper Oubangui, fragment at K sent from Oubangui, 1904 (syntype: K).
Coleus langouassiensis A.Chev., Veg. Ut. Afr. Trop. Franç. 1: 127. 1905. Type: not seen.

##### Distribution.

Trop. & South Africa.

#### Coleus eungellaensis

Taxon classificationPlantaeLamialesLamiaceae

P.I.Forst. & A.J.Paton nom. nov.

5779B0CE91555BC9B6B0E27545D49B54

urn:lsid:ipni.org:names:77201068-1

Plectranthus graniticola P.I.Forst., Austrobaileya 3: 732. 1992., non C. graniticola (A.Chev.) A.J.Paton. Type: Australia, Queensland, South Kennedy District, Clarke Range, Eungella National Park, 23 Apr. 1991, P.I.Forster PIF8056 & W.J.F. Mc Donald (holotype: BRI; isotypes: CNS, K, MEL).

##### Distribution.

Australia: Queensland.

#### Coleus excelsus

Taxon classificationPlantaeLamialesLamiaceae

(P.I.Forst.) P.I.Forst. comb. nov.

F229C1C981A25A0CB4946AE6C5039EC2

urn:lsid:ipni.org:names:77201070-1

Plectranthus excelsus P.I.Forst., Austrobaileya 4: 172. 1994. Type: Australia, Queensland, Cook District, Garroway Hill, 11 July 1993, P.I.Forster PIF13543 & M.C.Tucker (holotype: BRI, isotypes: A, AD, CBG, CNS, K, L, MEL).

##### Distribution.

Australia: Queensland.

#### Coleus exilis

Taxon classificationPlantaeLamialesLamiaceae

(A.J.Paton) A.J.Paton comb. nov.

0584ECAA793A554D87711286582FE26C

urn:lsid:ipni.org:names:77201071-1

Plectranthus exilis A.J.Paton, Fl. Trop. E. Afr., Lamiac.: 350. 2009. Type: Tanzania, Njombe District: Ruhudje, Lupembe, Schlieben 185 (holotype, K; isotypes: B, K, LISC, MO, PRE).

##### Distribution.

SW. Tanzania.

#### Coleus fasciculatus

Taxon classificationPlantaeLamialesLamiaceae

(P.I.Forst.) P.I.Forst. comb. nov.

1CFFB3D7258059C4968371B242E569DE

urn:lsid:ipni.org:names:77201072-1

Plectranthus fasciculatus P.I.Forst., Haseltonia 6: 14. 1998 publ. 1999. Type: Australia, Queensland, Cook District, Mt Windsor Tableland, State Forest 144, 24 July 1993, P.I.Forster PIF13709, G. Sankowsky & M.C. Tucker (holotype: BRI; isotype: CNS).

##### Distribution.

Australia: Queensland.

#### Coleus ferricola

Taxon classificationPlantaeLamialesLamiaceae

Phillipson, O. Hooper & A.J. Paton, Kew. Bull. 74, 24: 4. 2019

EDBEF0D8CB8F55B8922C2C847DFA9B17

Coleus ferricola Phillipson, O. Hooper & A.J. Paton, Kew. Bull. 74, 24: 4. 2019. Type: Guinea (Conakry), Nzérékoré, Lola, Guinée Forestière, Nimba Mountains, SMFG iron ore mine concession. East side of Pierre Richaud., Phillipson 6324 (holotype MO; isotypes BR, K, P, SERG, WAG).

#### Coleus foetidus

Taxon classificationPlantaeLamialesLamiaceae

(Benth.) A.J.Paton comb. nov.

97552498D3D45B15BF9045B04F15D45E

urn:lsid:ipni.org:names:77201073-1

Plectranthus foetidus Benth., Labiat. Gen. Spec.: 35. 1832.
Ocimum foetidum Banks ex Benth., Labiat. Gen. Spec.: 35. 1832. nom. nud. Type: Australia, Queensland, Endeavour River, 1770, Banks & Solander, (holotype: BM).

##### Distribuion.

Australia: Queensland.

#### Coleus foliatus

Taxon classificationPlantaeLamialesLamiaceae

(A.J.Paton) A.J.Paton comb. nov.

CB3171494ACD57189A62EA00435DA2FF

urn:lsid:ipni.org:names:77201074-1

Plectranthus foliatus A.J.Paton, Fl. Trop. E. Afr., Lamiac.: 284. 2009. Type: Tanzania, Sumbawanga District: Tatanda, Mbaa Hill, Bidgood et al. 3459 (holotype: K; isotypes: C, DSM, K, NHT).

##### Distribution.

SW. Tanzania to N. Zambia.

#### Coleus forsteri

Taxon classificationPlantaeLamialesLamiaceae

(Benth.) A.J.Paton comb. nov.

E7809D5F088F5AE69AB07091BF11E9A8

urn:lsid:ipni.org:names:77201075-1

Plectranthus forsteri Benth., Labiat. Gen. Spec.: 38. 1832. Type: Vanuatu, Tanna, J.R. & G. Forster (holotype, BM).

##### Distribution.

SW. Pacific.

#### Coleus fragrantissimus

Taxon classificationPlantaeLamialesLamiaceae

(P.I.Forst.) P.I.Forst. comb. nov.

249C6C344DBA57F8B85A2A83AAF778C8

urn:lsid:ipni.org:names:77201076-1

Plectranthus fragrantissimus P.I.Forst., Austrobaileya 8: 394. 2011. Type: Australia, Queensland, Port Curtis District, State Forest 316, Kroombit Tops, 16 Feb. 1995, P.I.Forster PIF16246 (holotype: BRI; MEL, NE, istotypes).

##### Distribution.

Australia: Queensland.

#### Coleus fredericii

Taxon classificationPlantaeLamialesLamiaceae

G.Taylor, J. Bot. 69 (Suppl. 2): 159. 1931

9A99019536FE5110B6F025FDD743880A

Neomuellera welwitschii Briq., Bot. Jahrb. Syst. 19: 180. 1894., non Coleus welwitschii Briq.
Plectranthus welwitschii (Briq.) Codd, Fl. Pl. Africa 42: t. 1646. 1972. Type: Angola, Pungo Andongo, April 1857, Welwitsch 5544 (isotypes: BM, C, K).

##### Distribution.

Angola.

#### Coleus fruticosus

Taxon classificationPlantaeLamialesLamiaceae

Wight ex Benth. in A.P.de Candolle, Prodr. 12: 78. 1848

C06F0E8DD3785B7BBEA4910F6227AD64

Plectranthus fruticosus (Wight ex Benth.) Hook.f., Fl. Brit. India 4: 623. 1885, nom. illeg.
Plectranthus deccanicus Briq., Annuaire Conserv. Jard. Bot. Genève 2: 234. 1898. Type: India, Pulney Hills, 1837, Wight 2514 (holotype: K).

##### Distribution.

SW. India

#### Coleus galeatus

Taxon classificationPlantaeLamialesLamiaceae

(Vahl) Benth., Labiat. Gen. Spec.: 56. 1832

02FFA3ACC72D5D12B682AABEE71CCD3F

Germanea galeata (Vahl) Poir. in J.B.A.M.de Lamarck, Encycl. 2: 763. 1788.
Majana galeata (Vahl) Kuntze, Revis. Gen. Pl. 2: 524. 1891.
Plectranthus galeatus Vahl, Symb. Bot. 1: 43. 1790. Type: Indonesia, Java, Vahl s.n. (holotype: C).
Ocimum plectranthus J.F.Gmel., Syst. Nat. ed. 13(bis): 917. 1792. Type: not seen.
Plectranthus bicolor Blume, Bijdr. Fl. Ned. Ind.: 837. 1826.
Coleus bicolor (Blume) Benth., Labiat. Gen. Spec.: 55. 1832.
Majana bicolor (Blume) Kuntze, Revis. Gen. Pl. 2: 524. 1891. Type: Indonesia, Java, Blume 2037. (lectotype: L, designated by Keng (1969)).
Plectranthus macrophyllus Blume, Bijdr. Fl. Ned. Ind.: 835. 1826.
Coleus macrophyllus (Blume) Benth., Labiat. Gen. Spec.: 55. 1832.
Majana macrophylla (Blume) Kuntze, Revis. Gen. Pl. 2: 524. 1891. Type: Indonesia, Java, Blume 1453 (syntype: L).
Plectranthus leschenaultii Benth., Labiat. Gen. Spec.: 34. 1832. Type: Java, Leschenault (holotype: P).
Coleus macrostachys Benth., Labiat. Gen. Spec.: 57. 1832. Type: Indonesia, Java, Commerson s.n. (lectotype: P, designated here).
Coleus macropus Miq., Fl. Ned. Ind. 2: 956. 1858. Type: Indonesia, ?Java, Horsfield s.n. (holotype: U; isotypes: BM, K).
Coleus puberulus Miq., Fl. Ned. Ind. 2: 955. 1858.
Majana puberula (Miq.) Kuntze, Revis. Gen. Pl. 2: 524. 1891. Type: Indonesia, Sumatra, Singalang, Teysmann s.n. (isotype: U).
Coleus remotiflorus Miq., Fl. Ned. Ind. 2: 954. 1858. Type: Indonesia, Java in Soerakerta, Horsfield Lab. 112 (holotype: U; isotypes: BM, K).
Coleus spectabilis Miq., Fl. Ned. Ind. 2: 955. 1858. Type: Indonesia, Java, in Blam.-bagan, Horsfield s.n. (holotype: U; isotypes: BM, K).

##### Distribution.

W. Malesia to Lesser Sunda Is. (Bali).

#### Coleus gamblei

Taxon classificationPlantaeLamialesLamiaceae

(Smitha & Sunojk.) Smitha comb. nov.

D8E0E09BA1A45F77A94BBE5324652092

urn:lsid:ipni.org:names:77201077-1

Plectranthus gamblei Smitha & Sunojk., Nordic J. Bot. 36 (8): 4. 2018. Type: India. Tamil Nadu, Coonoor, Aug 1883, Gamble 12263 (holotype: K).

##### Distribution.

S. India.

#### Coleus garckeanus

Taxon classificationPlantaeLamialesLamiaceae

Vatke, Linnaea 37: 323. 1872

710092C92DF35CCFAF16672B5772B2CA

Plectranthus garckeanus (Vatke) J.K.Morton, Novon 8: 265. 1998. Type: Ethiopia, Begember in valley of river Repp, 1 July 1863, Schimper 1193 (holotype: B, destroyed; isotypes: BM, E, JE, K).
Plectranthus salviiflorus Chiov., Malpighia 34: 516. 1937. Type: Ethiopia, Boyi near Kardobi and Abeba Kolla, Taschdjian 188 (syntype: FT); 124 (syntype: FT).

##### Distribution.

Ethiopia.

#### Coleus geminatus

Taxon classificationPlantaeLamialesLamiaceae

(P.I.Forst.) P.I.Forst. comb. nov.

EF1959025B775D3E964DEFED08E6F80B

urn:lsid:ipni.org:names:77200982-1

Plectranthus geminatus P.I.Forst., Austrobaileya 9: 285. 2014. Type: Australia, Queensland, Moreton District, Lamington National Park, Cainbable Falls, 27 Feb. 2014. P.I.Forster PIF40722 & G.Leiper (holotype: BRI; isotypes: K, MEL, NSW).

##### Distribution.

Australia: Queensland.

#### Coleus gibbosus

Taxon classificationPlantaeLamialesLamiaceae

A.J.Paton nom. nov.

11699B4C21205A1B9D16CC37121543E9

urn:lsid:ipni.org:names:77201079-1

Plectranthus rehmannii Gürke, Bull. Herb. Boissier 6: 553. 1898., non Coleus rehmanii Briq. Type: South Africa, Natal, Karkloof, Rehmann 7359 (holotype: Z; isotype: K).

##### Distribution.

South Africa: KwaZulu-Natal.

#### Coleus gigantifolius

Taxon classificationPlantaeLamialesLamiaceae

(Suddee) Suddee comb. nov.

439BC34F8615530593FC5A675683EA3C

urn:lsid:ipni.org:names:77201080-1

Plectranthus gigantifolius Suddee, Kew Bull. 59: 401 (2004). Type: Thailand, Kanchanaburi, Thung Yai Naresuarn Wildlife Sanctuary, Ban Saneh Pawng, Lai Wo Subdistr., 225 m, 7 Oct. 1993, Maxwell 93-1156 (holotype: CMU; isotypus: L).

##### Distribution.

SW. Thailand.

#### Coleus gillettii

Taxon classificationPlantaeLamialesLamiaceae

(J.K.Morton) A.J.Paton comb. nov.

D8B4F73840AD5BEB8FB2D946C6E11099

urn:lsid:ipni.org:names:77201081-1

Plectranthus gillettii J.K.Morton, Kew Bull. 53: 998. 1998. publ. 1999. Type: Kenya, Northern Frontier District: Moyale, Gillett 13721 (holotype: K; isotypes: EA, LMA).

##### Distribution.

S. Ethiopia, S. Somalia, N. Kenya.

#### Coleus glabriflorus

Taxon classificationPlantaeLamialesLamiaceae

(P.I.Forst.) P.I.Forst. comb. nov.

8EE40C1352355672ABF463F2682C897E

urn:lsid:ipni.org:names:77201082-1

Plectranthus glabriflorus P.I.Forst., Austrobaileya 4: 174. 1994. Type: Australia, Queensland, North Kennedy District, Herberton, 20 Jan. 1993, P.I.Forster PIF12814 & A.R.Bean (holotype: BRI; isotype: CNS).

##### Distribution.

Australia: Queensland.

#### Coleus globosus

Taxon classificationPlantaeLamialesLamiaceae

(Ryding) A.J.Paton comb. nov.

2999EEC71FDE5BE98C3B70A4ACE5F3D2

urn:lsid:ipni.org:names:77201083-1

Plectranthus globosus Ryding, Bull. Jard. Bot. Natl. Belg. 66: 101. 1997. Type: DRC, Shaba, Kundulungu Plateau, 20 Mar. 1971, Lisowski 23152 (holotype: POZG, photo C).

##### Distribution.

DRC.

#### Coleus goetzenii

Taxon classificationPlantaeLamialesLamiaceae

(Gürke) A.J.Paton comb. nov.

7BDADD094C8F5D0EA4379AB00F8F0AE5

urn:lsid:ipni.org:names:77201084-1

Pycnostachys goetzenii Gürke, Durch Afr., reimpr.: 8. 1896). Type: Rwanda, Virunga Mts, Goetzen 98 (holotype: B, destroyed).
Pycnostachys vulcanicola Lebrun & L.Touss., Bull. Jard. Bot. État Bruxelles 17: 71. 1943. Type: DRC, Virunga Range, Lebrun 5006 (holotype: BR; isotype: K).

##### Distribution.

EC. Trop. Africa (Virunga Mts.).

#### Coleus gossweileri

Taxon classificationPlantaeLamialesLamiaceae

A.J.Paton nom. nov.

33490339B59255FFB08DD672BE48A875

urn:lsid:ipni.org:names:77201085-1

Pycnostachys gracilis R.D.Good, J. Bot. 69 (suppl. 2): 164. 1931., non Coleus gracilis Gürke. Type: Angola, R. Cuartiri, Gossweiler 4035 (holotype: BM).

##### Distribution.

Angola.

#### Coleus gracillimus

Taxon classificationPlantaeLamialesLamiaceae

(T.C.E.Fr.) Robyns & Lebrun, Ann. Soc. Sci. Bruxelles, Sér. B 49: 106. 1929

AC0FC4CE129C5FA8929DC4A2F1B2FCCE

Englerastrum gracillimum T.C.E.Fr., Notizbl. Bot. Gart. Berlin-Dahlem 9: 69. 1924.
Plectranthus gracillimus (T.C.E.Fr.) Hutch. & Dandy, Bull. Misc. Inform. Kew 1926: 481. 1926. Type: DRC, Mt Corva, Deschamps s.n. (holotype: B, destroyed; isotypes: UPS, K, fragment).
Englerastrum schlechteri T.C.E.Fr., Notizbl. Bot. Gart. Berlin-Dahlem 9: 70. 1924.
Plectranthus schlechteri (T.C.E.Fr.) Hutch. & Dandy, Bull. Misc. Inform. Kew 1926: 481. 1926.
Coleus schlechteri (T.C.E.Fr.) Robyns & Lebrun, Ann. Soc. Sci. Bruxelles, Sér. B 49: 106. 1929. Types: Mozambique, Dondo near Beira, Schlechter 12240 (syntype: B, destroyed, isosyntype: PRE, K, fragment) & Tanzania, Rungwe District: Kyimbila, Mulinda, Stoltz 1440 (syntype: B, destroyed, isosyntypes: K, W).
Plectranthus micranthus Chiov., Boll. Soc. Bot. Ital. 1924: 44. 1924, nom. illeg., non Spreng.
Englerastrum diffusum Alston, Bull. Misc. Inform. Kew 1926: 298. 1926.
Plectranthus tenuis Hutch. & Dandy, Bull. Misc. Inform. Kew 1926: 481. 1926.
Coleus diffusus (Alston) Robyns & Lebrun, Ann. Soc. Sci. Bruxelles, Sér. B 49: 106. 1929. Type: Nigeria: Nupe, Barter s.n. (syntype: K); Nigeria, Muri Province, weed in arable land, Lamb 72 (syntype, K).

##### Distribution.

Widespread in Trop. Africa.

#### Coleus gracilipedicellatum

Taxon classificationPlantaeLamialesLamiaceae

(Robyns & Lebrun) A.J.Paton comb. nov.

7F92193B7D7A5194BB16E97B00058191

urn:lsid:ipni.org:names:77201086-1

Holostylon gracilipedicellatum Robyns & Lebrun, Ann. Soc. Sci. Bruxelles, Sér. B 49: 103. 1929. Type: DRC, Katanga, Pweto to Moba (Baudouinville), between Kayabala and Lungulungu, 29 Apr. 1926, Robyns 2196 (holotype: BR; isotypes: BM, K).
Plectranthus baumii Gürke in O. Warburg (ed.), Kunene-Sambesi Exped.: 356. 1903, non Coleus baumii Gürke.
Holostylon baumii (Gürke) G.Taylor, J. Bot. 69(suppl. 2): 161. 1931. Type: Angola, Kubango, Massaca, 19 Oct. 1899, Baum 283 (holotype: B, destroyed, isotypes: BM, E (as 238), K, W).

##### Distribution.

Southern DRC to Botswana.

#### Coleus gracilis

Taxon classificationPlantaeLamialesLamiaceae

Gürke, Bot. Jahrb. Syst. 38: 170. 1906

33C3E683198D538E844020BA52535672

Plectranthus puberulentus J.K.Morton, Novon 8: 265. 1998. Types: Ethiopia, Sagan, Riva 1564 (syntype: B, destroyed; isosyntype: FT), Harar, Ellenbeck 965 (syntype: B, destroyed).

##### Distribution.

Ethiopia to Tanzania.

#### Coleus graminifolius

Taxon classificationPlantaeLamialesLamiaceae

(Perkins) A.J.Paton comb. nov.

404C010CBFCE5437A7D350C5518A845E

urn:lsid:ipni.org:names:77201087-1

Pycnostachys graminifolia Perkins, Notizbl. Bot. Gart. Berlin-Dahlem 8: 66. 1921. Type: Tanzania, Dodoma District: Kilimatinde, Prittwitz 118 (holotype: B, destroyed; isotype: UPS, K, fragment).

##### Distribution.

Kenya.

#### Coleus grandicalyx

Taxon classificationPlantaeLamialesLamiaceae

E.A.Bruce, Hooker’s Icon. Pl. 34: t. 3391 1939

AE0F113F15085EC5B198D5D09FD0AC8D

Plectranthus grandicalyx (E.A.Bruce) J.K.Morton, Novon 8: 265. 1998. Type: Sudan, Imatong Mts, Lomul, A.S.Thomas 1935 (holotype: K).

##### Distribution.

S. Sudan to N. Uganda.

#### Coleus grandidentatus

Taxon classificationPlantaeLamialesLamiaceae

(Gürke) A.J.Paton comb. nov.

376BBE47C37451FAA3B56C01FD325163

urn:lsid:ipni.org:names:77201088-1

Plectranthus grandidentatus Gürke, Bull. Herb. Boissier 6: 554. 1898. Type: South Africa, Cape, East Griqualand, Emyembe Mt, April 1885, Tyson 1517 (lectotype: K, designated by Codd (1975)).

##### Distribution.

S. Zimbabwe to South Africa.

#### Coleus graniticola

Taxon classificationPlantaeLamialesLamiaceae

(A.Chev.) A.J.Paton comb. nov.

854BEE4A6DB2519FB28E4B92EB262F7A

urn:lsid:ipni.org:names:77201089-1

Solenostemon graniticola A.Chev., J. Bot. (Morot) 22: 121. 1909.
Solenostemon monostachyus var. graniticola (A.Chev.) Brenan, Kew Bull. 5: 231. 1950.
Plectranthus saxicola B.J.Pollard & A.J.Paton, Kew Bull. 61: 229. 2006. Type: Ivory Coast, Mt Nienokue, 20 km NE of Fort Binger, Chevalier 19469 (holotype: P; isotype: K).

##### Distribution.

W. & WC. Trop. Africa.

#### Coleus gratus

Taxon classificationPlantaeLamialesLamiaceae

(S.T.Blake) P.I.Forst. & T.C.Wilson comb. nov.

E5B4CEF2E41853A280B207A18282D171

urn:lsid:ipni.org:names:77201090-1

Plectranthus gratus S.T.Blake, Contr. Queensland Herb. 9: 49. 1971. Type: Australia, Queensland, Walsh’s Pyramid (Cultivated at The Gap), 10 Jan. 1960, S.T. Blake 21192 (holotype: BRI).

##### Distribution.

Australia: NE. Queensland.

#### Coleus graveolens

Taxon classificationPlantaeLamialesLamiaceae

(R.Br.) A.J.Paton comb. nov.

7B378354F4445F0C82192A5E8E6BC82D

urn:lsid:ipni.org:names:77201091-1

Plectranthus graveolens R.Br., Prodr. Fl. Nov. Holl.: 506. 1810.
Plectranthus parviflorus var. graveolens (R.Br.) Briq. in H.G.A.Engler & K.A.E.Prantl, Nat. Pflanzenfam. 4(3a): 357. 1895.
Plectranthus australis var. graveolens (R.Br.) Domin, Biblioth. Bot. 89: 565. 1928. Type: Australia, Queensland, Port Clinton, 22. Aug. 1802, R.Brown (holotype: BM).
Plectranthus australis f. eximius Domin, Biblioth. Bot. 89: 565. 1928. Type: not seen.

##### Distribution.

E. Australia, Lord Howe I.

#### Coleus guerkei

Taxon classificationPlantaeLamialesLamiaceae

(Briq.) A.J.Paton comb. nov.

07091383DD765B4CA75F84DC4D5F07DB

urn:lsid:ipni.org:names:77201092-1

Hyptis baumii Gürke in O. Warburg (ed.), Kunene-Sambesi Exped.: 354. 1903., non Coleus baumii Gürke.
Plectranthus guerkei Briq., Annuaire Conserv. Jard. Bot. Genève 7–8: 323. 1904.
Germanea guerkei Briq., Annuaire Conserv. Jard. Bot. Genève 7–8: 323. 1904., nom. altern. Type: Angola, Cuito (Kuito), Baum 789 (isotypes: K, W).
Geniosporum paniculatum Baker in D.Oliver & auct. suc. (eds.), Fl. Trop. Afr. 5: 351. 1900., non Coleus paniculatus Benth.
Neohyptis paniculata (Baker) J.K.Morton, J. Linn. Soc., Bot. 58: 259. 1962. Type: Angola, Pungo Andongo, near Catete, Welwitsch 5528 (syntypes: BM, K).
Geniosporum paniculatum var. debile Hiern, Cat. Afr. Pl. 1: 853. 1900. Type: Angola, Pungo Andongo, Casalala near Pedra Songue, Welwitsch 5527 (holotype: LISU; isotype: BM).
Hyptis quadrialata A.Chev., Explor. Bot. Afrique Occ. Franç. 1: 522. 1920., nom. nud.
Geniosporum borzianum Chiov. Ann. Bot. (Rome) 10: 402. 1912. Type: Ethiopia. Amhara-Dembià, Mt. Incedubà near Gondar, Chiovenda 2266 (holotype: FT), synon. nov.

##### Distribution.

Widespread in Trop. & South Africa.

#### Coleus gymnostomus

Taxon classificationPlantaeLamialesLamiaceae

Gürke, Bot. Jahrb. Syst. 19: 212. 1894

3203B26ABCC6500995F8418C8B49DF05

Plectranthus gymnostomus (Gürke) A.J.Paton, Fl. Trop. E. Afr., Lamiac.: 337. 2009. Type: East Africa, without precise locality, Fischer 333 (holotype: B, destroyed; isotype: K, photo.).

##### Distribution.

Tanzania.

#### Coleus habrophyllus

Taxon classificationPlantaeLamialesLamiaceae

(P.I.Forst.) P.I.Forst. comb. nov.

431DEBE8A5E85641B0857E8D44640C62

urn:lsid:ipni.org:names:77201093-1

Plectranthus habrophyllus P.I.Forst., Austrobaileya 4: 175. 1994. Type: Australia, Queensland, Moreton District, Ormeau, CSR land near water tower, 12 Nov. 1992, P.I.Forster PIF12391 & G.Leiper (holotype: BRI, isotypes: K, MEL).

##### Distribution.

Australia: Queensland.

#### Coleus hadiensis

Taxon classificationPlantaeLamialesLamiaceae

(Forssk.) A.J.Paton comb. nov.

CB1026D886145A4CB550FA4C13D58EF9

urn:lsid:ipni.org:names:77201104-1

Ocimum hadiense Forssk., Fl. Aegypt.-Arab.: 109. 1775.
Plectranthus forskalaei Vahl, Symb. Bot. 1: 44. 1790., nom. illeg.
Germanea forskalaei Poir. in J.B.A.M.de Lamarck, Encycl., suppl. 2: 764. 1812., nom. superfl.
Majana forskalaei (Poir.) Kuntze, Revis. Gen. Pl. 2: 524. 1891., nom. superfl.
Plectranthus hadiensis (Forssk.) Schweinf. ex Sprenger, Wiener Ill. Gart.-Zeitung 19: 2. 1894.
Coleus forskalaei Briq. in H.G.A.Engler & K.A.E.Prantl, Nat. Pflanzenfam. 4(3a): 359. 1897., nom. superfl. Type: Yemen, Hadiyah ((Hadi)) Mts, Forsskål s.n (C, holotype).
Plectranthus zeylanicus Benth., Labiat. Gen. Spec.: 36. 1832.
Coleus zeylanicus (Benth.) L.H.Cramer, Kew Bull. 32: 560. 1978. Type: Ceylon, Macrae s.n. (holotype: K).
Plectranthus tomentosus Benth. in E.H.F.Meyer, Comm. Pl. Afr. Austr.: 229. 1838. Plectranthus zatarhendi var. tomentosus (Benth.) Codd, Bothalia 11: 399. 1975.
Plectranthus hadiensis var. tomentosus (Benth.) Codd, Fl. S. Afr. 28: 153. 1985. Type: South Africa, Durban (Port Natal), Drège s.n. (holotype: K).
Coleus personatus Lem., Hort. Universel 6: 128. 1844. Type: not seen. Material at G under this name, labelled Hort Paris Aug. 1845, is P. hadiensis.
Plectranthus madagascariensis var. ramosior Benth. in A.P.de Candolle, Prodr. 12: 68. 1849.
Plectranthus ramosior (Benth.) van Jaarsv., Aloe 43: 46. 2006. Type: South Africa, Gauteng, Magaliesberg (Macalisberg), Burke s.n. (holotype: BM).
Coleus schweinfurthii Vatke, Linnaea 37: 323. 1872. Type: Ethiopia, Bellaka, Sept. 1854, Schimper s.n. (Type: destroyed B).
Plectranthus paucicrenatus Franch., Sert. Somal.: 56. 1882. Type: Somalia, Northern Somalia, Révoil *s.n.* (holotype: P).
Coleus rupestris Hochst. ex Briq., Bull. Herb. Boissier 2: 131. 1894., nom. nud.
Plectranthus cyaneus Gürke, Bot. Jahrb. Syst. 19: 208. 1894. Type: Tanzania, Lushoto District: Mashewa (Mascheua), Holst 8850 (syntype: B, destroyed; isosyntype: K) & Handei, near Kwa Mshusa, Holst 8984 (syntype: B, destroyed; isosyntype: JE, K, W).
Plectranthus woodii Gürke, Bot. Jahrb. Syst. 26: 76. 1898.
Plectranthus zatarhendi var. woodii (Gürke) Codd, Bothalia 11: 401. 1975.
Plectranthus hadiensis var. woodii (Gürke) Codd, Fl. S. Afr. 28: 154. 1985. Type: South Africa, KwaZulu-Natal, Ipolweni, Medley Wood s.n. (lectotype: GRA, designated by Codd (1975)).
Germanea horrida Hiern, Cat. Afr. Pl. 1: 863. 1900.
Plectranthus horridus (Hiern) Baker in D.Oliver & auct. suc. (eds.), Fl. Trop. Afr. 5: 416. 1900. Types: Angola, Pungo Andongo, R. Cuanza near Condo, Welwitsch 5537 (syntypes: BM, K, LISU.); Huila, Morro de Monino, Welwitsch 5613 (syntypes: LISU, K).
Plectranthus rupestris Vatke ex Baker in D.Oliver & auct. suc. (eds.), Fl. Trop. Afr. 5: 409. 1900.
Coleus rupestris Hochst. ex Baker in D.Oliver & auct. suc. (eds.), Fl. Trop. Afr. 5: 409. 1900., nom. inval. (based on Schimper 2172 as below) Type: Ethiopia, near Bellaka, Sept. 1854, Schimper 2172 (lectotype: K; isolectotypes: BM, W, designated by Paton et al. 2009).
Plectranthus draconis Briq., Bull. Herb. Boissier, sér. 2, 3: 1071. 1903. Type: South Africa, KwaZulu-Natal, Biggarsberg, Rehmann 7092 (holotype: Z).
Plectranthus hararensis Gürke, Bot. Jahrb. Syst. 36: 132. 1905. Type: Ethiopia, Harar, Bachufer, Ellenbeck 846 (holotype: B, destroyed; K, fragment).
Plectranthus erlangeri Gürke, Bot. Jahrb. Syst. 38: 166. 1906. Type: Ethiopia, Abuelkasim, Ellenbeck 1396 (holotype: B, destroyed but see fig. in protologue).
Plectranthus pachyphyllus Gürke ex T.Cooke in W.H.Harvey & auct. suc. (eds.), Fl. Cap. 5(1): 285. 1910. Type: South Africa, Natal, Inchanga, Rehmann 7878 (holotype: Z).
Plectranthus petrensis S.Moore, J. Linn. Soc., Bot. 40: 175. 1911. Type: Zimbabwe, Chimanimani, Swynnerton 2018 (holotype: BM; isotype: K).
Plectranthus fragrans Lebrun & L.Touss., Bull. Jard. Bot. État Bruxelles 17: 70. 1943. Type: DRC, Katanda, Lebrun 7618 (holotype: BR; isotype: K).

##### Distribution.

Widespread, Egypt to South Africa, Arabian Peninsula, Maldives, Sri Lanka.

##### Notes.

Heckenhauer et al. (2019) suggest the Sri Lankan *C. zeylanicus* may represent a separete species or subspecies but further work is required.

#### Coleus hallii

Taxon classificationPlantaeLamialesLamiaceae

(J.K.Morton) A.J.Paton comb.nov.

7DFA8723CBD65071AAF3428E4FC2765E

urn:lsid:ipni.org:names:77201105-1

Plectranthus hallii J.K.Morton, J. Linn. Soc., Bot. 58: 267. 1962. Type: Ghana, Gambaga Scarp, 14 Nov. 1959, J.K.Morton A3791, (holotype: GC; isotype: K).

##### Distribution.

Ghana.

#### Coleus harmandii

Taxon classificationPlantaeLamialesLamiaceae

(Doan ex Suddee & A.J.Paton) A.J.Paton comb. nov.

3FEF7B50FF6854B1B09D9DB861E7268C

urn:lsid:ipni.org:names:77201106-1

Fig. 4E

Anisochilus harmandii Doan ex Suddee & A.J.Paton, Kew Bull. 59: 384. 2004. Type: Cambodia, Mlu Prey, Jan. 1876, Harmand 326 (holotype: P; isotype: A).

##### Distribution.

Indo-China.

#### Coleus helferi

Taxon classificationPlantaeLamialesLamiaceae

(Hook.f.) A.J.Paton comb. nov.

30CE589FB87E5247B11A3B92CCA13D3C

urn:lsid:ipni.org:names:77201107-1

Plectranthus helferi Hook.f., Fl. Brit. India 4: 623. 1885. Type: Burma, Tenasserim, Helfer 4061 (holotype: K).
Plectranthus fulvescens Prain, J. Asiat. Soc. Bengal, Pt. 2, Nat. Hist. 66: 521. 1897. Type: Burma, Attran, Brandis 811 (holotype: CAL; isotype: K),
Coleus fulvescens Kurz, Mss in Herb. Calcutta, nom. inval., in protologue discussion above.

##### Distribution.

Myanmar to N. Thailand.

#### Coleus hereroensis

Taxon classificationPlantaeLamialesLamiaceae

(Engl.) A.J.Paton comb. nov.

0A03D34E51075D769F82C049F4F0D441

urn:lsid:ipni.org:names:77201108-1

Plectranthus hereroensis Engl., Bot. Jahrb. Syst. 10: 267. 1888.
Burnatastrum hereroensis (Engl.) P.V.Heath, Calyx 4: 175. 2001. Type: Namibia, Hereroland, Kaiser Wilhelmsberg near Okahandja, May 1886, Marloth 1350 (holotype: B, destroyed; isotypes: G, M, PRE, SAM).
Coleus aconitiflorus Welw. ex Hiern, Cat. Afr. Pl. 1: 866. 1900. Type: Angola, Huíla, Catumba, Mar.1879, Welwitsch 5495 (holotype: LISU; isotypes: G, K).
Plectranthus matabelensis Baker in D.Oliver & auct. suc. (eds.), Fl. Trop. Afr. 5: 417. 1900. Type: Zimbabwe, Matabeleland, Shashe R., 27.Mar.1875, Holub 1403–1406 (holotype: K).
Neomuellera damarensis S.Moore, J. Bot. 1901: 265. 1901. Type: Namibia, Damaraland, 1879, Een s.n. (holotype: BM).
Germanea myriantha Briq., Bull. Herb. Boissier, sér. 2, 3: 1001. 1903.
Plectranthus myrianthus Briq., Bull. Herb. Boissier, sér. 2, 3: 1001. 1903.
Coleus myrianthus (Briq.) Brenan, Mem. New York Bot. Gard. 9: 43. 1954. Type: South Africa, Witwatersrand, Apr.1895, Hutton 877 (holotype: Z: isotypes: GRA, K, PRE).
Coleus matopensis S.Moore, J. Bot. 45: 96. 1907. Type: Zimbabwe, Matobo Hills, Eyles 1024 (holotype: BM; isotype: GRA).
Coleus polyanthus S.Moore, J. Bot. 45: 96. 1907. Type: Zimbabwe, Matobo Hills, Nov. 1903, Eyles 1012 (holotype: BM).
Coleus gazensis S.Moore, J. Linn. Soc., Bot. 40: 178. 1911. Type: Zimbabwe, Chimanimani (Melsetter), 28 June1907, Swynnerton 1998 (holotype: BM).
Plectranthus otaviensis Dinter, Repert. Spec. Nov. Regni Veg. Beih. 53: 116. 1928. nom. inval. Based on Namibia, Otavi, Dinter 5699 (B, destroyed, PRE, SAM).
Plectranthus aurifer Dinter ex Launert, Mitt. Bot. Staatssamml. München 2: 312. 1957. Type: Namibia, Grootfontein, Nossib, 22 Apr.1934, Dinter 7367 (holotype: M, isotypes: BOL, K).

##### Distribution.

Southern tropical & South Africa.

#### Coleus hijazensis

Taxon classificationPlantaeLamialesLamiaceae

(Abdel Khaik) A.J.Paton comb. nov.

88D2EEAB5059561189D863D836E3FB01

urn:lsid:ipni.org:names:77201109-1

Plectranthus hijazensis Abdel Khalik, Turkish J. Bot. 40(5): 507. 2016. Type: Saudi Arabia, Al Baha Province, Saad Medhass, Abdel Khalik & Howladar s.n (holotype: UQU; isotypes: E, K, SHG).

##### Distribution.

SW. Arabian Peninsula

#### Coleus humulopsis

Taxon classificationPlantaeLamialesLamiaceae

(B.J.Pollard) A.J.Paton comb. nov.

281D85B75F2D5047926D46F6DC179F7B

urn:lsid:ipni.org:names:77201110-1

Leocus membranaceus J.K.Morton, J. Linn. Soc., Bot. 58: 270. 1962., non Coleus membranaceus Briq.
Plectranthus humulopsis B.J.Pollard, Kew Bull. 64: 260. 2009. Type: Ghana, Kintampo, Morton A2585 (holotype: GC; isotypes: FHI, IFAN, K, WAG).

##### Distribution.

W. Trop. Africa to Cameroon.

#### Coleus hymalis

Taxon classificationPlantaeLamialesLamiaceae

(J.R.I.Wood) A.J.Paton comb. nov.

914F5E6F05B8501980BA7DA6ABF23067

urn:lsid:ipni.org:names:77201111-1

Plectranthus hyemalis J.R.I.Wood, Kew Bull. 39: 133. 1984. Type: Yemen, between Musalla & Saqool, Ahkum, Hujariyah, 2 Feb. 1977, J.R.I. Wood 1532 (holotype: K; isotypes BM, E).

##### Distribution.

Yemen.

#### Coleus idukkianus

Taxon classificationPlantaeLamialesLamiaceae

(J.Mathew, Yohannan & B.J.Conn) Smitha comb. nov.

D369DECF8A3959D8A030CD58BDF3E6CA

urn:lsid:ipni.org:names:77201112-1

Plectranthus idukkianus J.Mathew, Yohannan & B.J.Conn, Telopea 20: 183. 2017. Type: India: Kerala: Idukki District, 10 km away from Kuttikkanam, Panchalimedu Hills, 14 Dec. 2015, J. Mathew 4821 (holotype: TBGT; isotype: MSSRF).

##### Distribution.

India, endemic to the Western Ghats.

#### Coleus igniarioides

Taxon classificationPlantaeLamialesLamiaceae

(Ryding) A.J.Paton comb. nov.

02D1F1DAFE2B5DB9B8DBB6350480C080

urn:lsid:ipni.org:names:77201113-1

Plectranthus igniarioides Ryding, Novon 15: 361. 2005. Type: Somalia, Sanaag, 43 km from Ceel Afweyn, on road to Ceerigaabo, M. Thulin 10768 (holotype: UPS; isotype: K).

##### Distribution.

Somalia.

#### Coleus igniarius

Taxon classificationPlantaeLamialesLamiaceae

Schweinf., Beitr. Fl. Aethiop.: 121. 1867

D453AD03D9C55572A1796C04655FD83F

Plectranthus igniarius (Schweinf.) Agnew, Upland Kenya Wild Fl.: 638. 1974. Types: Ethiopia, Dehli-Dikeno, 1854, Schimper 527 (holotype: B, destroyed; neotype: Ethiopia, Gondar Region, Dehli Dikeno, July 1853, Schimper 1623 (neotype: P, designated by Ryding (2001)).
Plectranthus malinvaldii Briq., Bull. Herb. Boissier 2: 125. 1894.
Coleus malinvaldii (Briq.) Briq., Annuaire Conserv. Jard. Bot. Genève 2: 240. 1898. Type: Ethiopia, Gondar Region, Dehli Dikeno, 22 Oct. 1854, Schimper 529 (lectotype: G-DC; isolectotype: G, FT, P; K, photo., designated by Ryding (2001)).
Coleus degasparisianus Buscal. & Muschl., Bot. Jahrb. Syst. 49: 487. 1913. Types: Mozambique, Mbusi (but in reality from Mahio in Haddes Valley, Eritrea), von Aosta (holotype: B, destroyed; neotype: Eritrea, Arrot Valley, south of Aidereso, Schweinfurth & Riva 1398 (neotype: FT; BR, G, P, S, isoneo. designated by Ryding (2001)).
Coleus guidottii Chiov., Atti Reale Accad. Italia, Mem. Cl. Sci. Fis. 11: 54. 1940. Type: Eritrea, Habab, Cub Cub near Nacfa, Guidotti 776 (holotype: FT).
Plectranthus igniarius subsp. grandicalyx Beentje, Kenya Trees, Shrubs & Lianas: 633. 1994, nom. inval. Based on: Kenya, Mt Kulal, Verdcourt 2269 (single collection at K or EA not specified).

##### Distribution.

NE. Trop. Africa to N. Kenya, Arabian. Peninsula.

#### Coleus ignotus

Taxon classificationPlantaeLamialesLamiaceae

(A.J.Paton) A.J.Paton comb. nov.

F16EF7422E5551A0B22DA5ED6A1BDDF7

urn:lsid:ipni.org:names:77201114-1

Plectranthus ignotus A.J.Paton, Fl. Trop. E. Afr., Lamiac.: 278. 2009. Type: Tanzania, Ufipa District: Mbisi (Mbizi) Mts, Bidgood, Mbago & Vollesen 2544 (holotype: K; isotypes: C, DSM, NHT).

##### Distribution.

SW. Tanzania.

#### Coleus inflatus

Taxon classificationPlantaeLamialesLamiaceae

Benth., Labiat. Gen. Spec.: 58. 1832

C14D1923EEE3559783C819B175165C62

Majana inflata (Benth.) Kuntze, Revis. Gen. Pl. 2: 524. 1891.
Plectranthus inflatus (Benth.) R.H.Willemse, Blumea 25: 509. 1979. Type: Ceylon, Macrae s.n., in Hb. Benth. (holotype: K (K000820110)).
Coleus benthamianus Arn., Nova Acta Phys.-Med. Acad. Caes. Leop.-Carol. Nat. Cur. 18: 354. 1836.
Majana benthamiana (Arn.) Kuntze, Revis. Gen. Pl. 2: 524. 1891. Type: Ceylon, Graham s.n. (type: not seen).

##### Distribution.

Sri Lanka.

#### Coleus inselbergii

Taxon classificationPlantaeLamialesLamiaceae

(B.J.Pollard & A.J.Paton) A.J.Paton comb. nov.

58AE5A6B8D3F5B33A26840F64E710ABA

urn:lsid:ipni.org:names:77201115-1

Plectranthus inselbergi B.J.Pollard & A.J.Paton, Kew Bull. 61: 225. 2006. Type: Equatorial Guinea, Dalle Rocheuse (inselberg), 3 km S of Asok, 13 Jan. 1999, LeJoly 99/282 (holotype: BRLU; isotype: K).

##### Distribution.

Equatorial Guinea to NW. Gabon.

#### Coleus insignis

Taxon classificationPlantaeLamialesLamiaceae

(Hook. f.) A.J.Paton comb. nov.

4A11BB56FAAB56DEAE489530D78D6C47

urn:lsid:ipni.org:names:77201116-1

Plectranthus insignis Hook.f., J. Proc. Linn. Soc., Bot. 7: 210. 1864. Type: Cameroons Upper Guinea Cameroon Mountain, 7000 ft., Mann 1257 (holotype: K).

##### Distribution.

Cameroon, Gabon, Central African Rep.

#### Coleus insolitus

Taxon classificationPlantaeLamialesLamiaceae

(C.H.Wright) Robyns & Lebrun, Ann. Soc. Sci. Bruxelles, Sér. B 49: 94. 1929

891946BC47495B868C4804504C943CD1

Symphostemon insolitus (C.H.Wright) Hiern, Cat. Afr. Pl. 1: 867. 1900.
Plectranthus insolitus C.H.Wright, J. Linn. Soc., Bot. 34: 275. 1900. Type: Angola, Welwitsch 5593 (holotype: BM).

##### Distribution.

Angola.

#### Coleus insularis

Taxon classificationPlantaeLamialesLamiaceae

(P.I.Forst.) P.I.Forst. comb. nov.

7F73D2314FFE58F18754B53FAA2244D0

urn:lsid:ipni.org:names:77201117-1

Plectranthus insularis P.I.Forst., Austrobaileya 8: 398. 2011. Type: Australia, Queensland, Darling Downs District, Mingimarny State Forest 131, Pine hill, 20 km S of Millmerran, 28 Apr. 1995, P.I.Forster PIF16468 & S.J.Figg (holotype: BRI; isotype: MEL).

##### Distribution.

Australia: Queensland.

#### Coleus intraterraneus

Taxon classificationPlantaeLamialesLamiaceae

(S.T.Blake) P.I.Forst. & T.C.Wilson comb. nov.

3B82D6CCFB575BCF9F28993BD84DC276

urn:lsid:ipni.org:names:77201118-1

Plectranthus intraterraneus S.T.Blake, Contr. Queensland Herb. 9: 33. 1971. Type: Australia, Northern Territory, Palm Valley W of Alice Springs (Cultivated at The Gap), 1958, S.T.Blake 20193 (holotype: BRI).

##### Distribution.

Australia: Queensland, Northern Territory, South Australia, Western Australia.

#### Coleus kanneliyensis

Taxon classificationPlantaeLamialesLamiaceae

L.H.Cramer & S. Balas., Kew Bull. 32: 558. 1978

DFB3730B8CEF5FB6AB9C990864D8DF85

Plectranthus kanneliyensis (L.H.Cramer & S. Balas.) R.H.Willemse, Blumea 25: 510. 1979. Type: Sri Lanka, Kanneliya, Cramer 4317 (holotype: PDA; isotypes: E, K, NVB, US).

##### Distribution.

Sri Lanka.

#### Coleus kanyakumariensis

Taxon classificationPlantaeLamialesLamiaceae

(Shinoj & Sunojk.) Smitha comb. nov.

4489B05666C0538EAA3A8CF2A579BDBF

urn:lsid:ipni.org:names:77201119-1

Anisochilus kanyakumariensis Shinoj & Sunojk. Phytotaxa 333(1): 100. 2018. Type: India, Kanyakumari district, Maruthwamala, K.Shinoj & P.Sunojkumar CU138400a (holotype: CALI; isotypes (CALI, MH).

##### Distribution.

S. India.

#### Coleus kapatensis

Taxon classificationPlantaeLamialesLamiaceae

R.E.Fr., Wiss. Erg. Schwed. Rhod.-Kongo Exped. 1: 283. 1916

2D706495C0B55F3E91ED51855EFDEA24

Plectranthus kapatensis (R.E.Fr.) J.K.Morton, Novon 8: 265. 1998. Type: Zambia, Bangweulu (Bangwelo), Kamindas on Kapata Peninsula, R.E.Fries 871 (isotype: K).

##### Distribution.

S. Tanzania to S. Trop. Africa.

#### Coleus kirkii

Taxon classificationPlantaeLamialesLamiaceae

(Baker) A.J.Paton comb. nov.

9B71E3D57D615A00926719E90CEFE222

urn:lsid:ipni.org:names:77201120-1

Pycnostachys kirkii Baker in D.Oliver & auct. suc. (eds.), Fl. Trop. Afr. 5: 381. 1900. Type: Malawi, Manganja Hills, Kirk s.n. (lectotype; K, designated by Bramley in Paton et al. (2009)).
Echinostachys reticulata E.Mey., Comm. Pl. Afr. Austr.: 243. 1838., non Coleus reticulatus A.Chev.
Pycnostachys reticulata (E.Mey.) Benth. in A.P.de Candolle, Prodr. 12: 83. 1848. Type: South Africa, KwaZulu-Natal, Umlazi Valley, near Durban, Drège s.n. (holotype: K; isotype: PRE).
Pycnostachys reticulata var. angustifolia Benth. in A.P.de Candolle, Prodr. 12: 83. 1848. Types: South Africa, KwaZulu-Natal, Durban, 1840, Krauss 329 (syntype: K); Gauteng, Magaliesburg, n.d., Burke s.n. (syntype: K).
Pycnostachys uliginosa Gürke, Bot. Jahrb. Syst. 30: 396. 1901. Type: Tanzania, Njombe District: Ubena, Luhigi stream, Goetze 806 (holotype: B, destroyed; K, fragment).
Pycnostachys holophylla Briq., Bull. Herb. Boissier, sér. 2, 3: 1000. 1903. Type: South Africa, Gauteng, Johannesburg, n.d., E.S.C.A. Herb. 347 (holotype: G).
Pycnostachys purpurascens Briq., Bull. Herb. Boissier, sér. 2, 3: 998. 1903. Type: South Africa, Witwatersrand, no locality, April.1895, Hutton 878 (holotype: Z).
Pycnostachys schlechteri Briq., Bull. Herb. Boissier, sér. 2, 3: 999. 1903. Type: South Africa, Eastern Cape, Mt Frere, 24 Jan.1895, Schlechter 6406 (holotype Z; isotypes: GRA, PRE).

##### Distribution.

Nigeria to Cameroon, Tanzania to South Africa.

#### Coleus kivuensis

Taxon classificationPlantaeLamialesLamiaceae

Lebrun & L.Touss., Bull. Jard. Bot. État Bruxelles 17: 72. 1943

6D0E0A6D54835FCCB2D3E95CDC93E42D

Plectranthus kivuensis (Lebrun & L.Touss.) R.H.Willemse, Kew Bull. 40: 96. 1985. Type: DRC, Kivu, Rutshuru, Lebrun 9031 (holotype BR; isotype: K).

##### Distribution.

Eritrea to N. Tanzania.

#### Coleus klossii

Taxon classificationPlantaeLamialesLamiaceae

(S.Moore) P.I.Forst. & T.C.Wilson comb. nov.

7504C1349C835A80A8DD3E53B16A10BE

urn:lsid:ipni.org:names:77201121-1

Plectranthus klossii S.Moore, Trans. Linn. Soc. London, Bot. 9: 137. 1916. Type: Indonesia, Papua, Utakwa River to Mt Carstensz, C. Boden-Kloss (holotype: BM (BM000950283); isotype: K).
Plectranthus klossii var. major S.Moore, Trans. Linn. Soc. London, Bot. 9: 137. 1916. Type: Indonesia, Papua, Utakwa River to Mt Carstenz, C. Boden-Kloss (holotype: BM (BM000950282)).

#### Coleus koualensis

Taxon classificationPlantaeLamialesLamiaceae

A.Chev. ex Hutch. & Dalziel, Fl. W. Trop. Afr. 2: 290. 1931

776D773C74575AB0BA5A627D308F0AE5

Solenostemon koualensis (A.Chev. ex Hutch. & Dalziel) J.K.Morton, J. Linn. Soc., Bot. 58: 270. 1962.
Plectranthus koualensis (A.Chev. ex Hutch. & Dalziel) B.J.Pollard & A.J.Paton, Kew Bull. 61: 228. 2006. Type: Ivory Coast, Upper Sassandra, Mt Dourou, Fleury in Chevalier 21715 (holotype: P).

##### Distribution.

Ivory Coast (Mt. Dourou).

#### Coleus koulikoroensis

Taxon classificationPlantaeLamialesLamiaceae

A.J.Paton nom. nov.

74B0C809881751AD9EBC214ED06BA9D2

urn:lsid:ipni.org:names:77201122-1

Solenostemon chevalieri Briq., Mém. Soc. Bot. France 8: 286. 1917., non Coleus chevalieri Briq.
Plectranthus chevalieri (Briq.) B.J.Pollard & A.J.Paton, Kew Bull. 61: 229. 2006. Type: Mali, Koulikoro, Chevalier 2779 (holotype: P).
Coleus nigericus A.Chev., Explor. Bot. Afrique Occ. Franç. 1: 519. 1920, nom. nud.

##### Distribution.

W. Trop. Africa.

#### Coleus kunstleri

Taxon classificationPlantaeLamialesLamiaceae

(Prain) A.J.Paton comb. nov.

61FFD9DF75CA55BC83D4C8459DC64513

urn:lsid:ipni.org:names:77201123-1

Plectranthus kunstleri Prain, J. Asiat. Soc. Bengal, Pt. 2, Nat. Hist. 66: 521. 1897. Type: Malaysia, Perak, Kwala Dipoug (Kuala Dipang), Kunstler, 8240 (holotype: CAL).

##### Distribution.

Peninsula Malaysia (Perak).

#### Coleus lactiflorus

Taxon classificationPlantaeLamialesLamiaceae

Vatke, Linnaea 43: 89. 1881

A5C1F299ED0D5F5B8990586DD4784552

Plectranthus lactiflorus (Vatke) Agnew, Upland Kenya Wild Fl.: 637. 1974. Type: Kenya, Teita District: Ndi Hill (N’Di), Hildebrandt 2522 (holotype: B, destroyed; neotype: Kenya, Teita District, Mbololo Forest, Taita Hills Expedition 1042 (neotype: K, isoneotype: EA, designated by Paton et al. (2009)).

##### Distribution.

Ethiopia to N. & NW. Tanzania.

#### Coleus laetus

Taxon classificationPlantaeLamialesLamiaceae

(P.I.Forst.) P.I.Forst. comb. nov.

AE0B8A9A1660515E99848975FFF4C489

urn:lsid:ipni.org:names:77201124-1

Plectranthus laetus P.I.Forst., Austrobaileya 9: 431. 2015. Type: Australia, Queensland, Cook District, Orchid Creek Station, 30 April 2014, P.I.Forster PIF41138 & S.L.Thompson (holotype: BRI; isotypes: CNS, MEL, NSW).

##### Distribution.

Australia: Queensland.

#### Coleus lageniocalyx

Taxon classificationPlantaeLamialesLamiaceae

Briq., Mém. Soc. Bot. France 8: 292. 1917

79AE9C2F796F5CF694F2770FDB523E4D

Coleus lageniocalyx Briq., Mém. Soc. Bot. France 8: 292. 1917. Type: Central African Republic, Bassinde Haute Ombella, Kaga Do, near Diouma, 26 Oct.1902, Chevalier 5923 (holotype: P).

##### Distribution.

Central African Republic

#### Coleus lanceolatus

Taxon classificationPlantaeLamialesLamiaceae

(Bojer ex Benth.) A.J.Paton & Phillipson comb. nov.

5BD843FB12B252D29F89E39C72152526

urn:lsid:ipni.org:names:77201125-1

Plectranthus lanceolatus Bojer ex Benth., Labiat. Gen. Spec.: 40. 1832.
Burnatastrum lanceolatum (Bojer ex Benth.) Briq. in H.G.A.Engler & K.A.E.Prantl, Nat. Pflanzenfam. 4(3a): 358. 1897. Type: Madagascar, Bojer s.n. (holotype: P; isotype: K).
Plectranthus lavanduloides Baker, J. Linn. Soc., Bot. 20: 230. 1883.
Burnatastrum lavanduloides (Baker) Briq. in H.G.A.Engler & K.A.E.Prantl, Nat. Pflanzenfam. 4(3a): 358. 1897. Type: Madagascar, Central Madagascar, Baron 978 (holotype: K).
Plectranthus burnatii Briq., Bull. Herb. Boissier 2: 124. 1894. Type: Madagascar, around Antananarive, Goudot s.n. (holotype: G; isotype: G).

##### Distribution.

Madagascar.

#### Coleus lancifolius

Taxon classificationPlantaeLamialesLamiaceae

(Bramley) A.J.Paton comb. nov.

4A37F371476B5E73832C771295243A37

urn:lsid:ipni.org:names:77201126-1

Pycnostachys lancifolia Bramley, Kew Bull. 60: 590. 2005 publ. 2006. Type: Tanzania, Kigoma District: Lubalisi village to Ntakatta, Bidgood, Leliyo & Vollesen 4621 (holotype: K; isotypes: C, DSM, NHT, P).

##### Distribution.

W. Tanzania.

#### Coleus lanuginosus

Taxon classificationPlantaeLamialesLamiaceae

Hochst. ex Benth. in A.P.de Candolle, Prodr. 12: 79. 1848

25A6CF4494D157E19A3B8A9695A06DBD

Majana lanuginosa (Hochst. ex Benth.) Kuntze, Revis. Gen. Pl. 2: 524. 1891.
Plectranthus lanuginosus (Hochst. ex Benth.) Agnew, Upland Kenya Wild Fl.: 638. 1974. Type: Ethiopia, Adua, 4 Oct. 1842, Schimper 1915 (lectotype: K; isolectotypes: BM, FT, KIEL, UPS, W; designated by Ryding (2000)).
Coleus albidus Vatke, Linnaea 37: 321. 1872. Type: Ethiopia, Mettaro, 1 Oct. 1862, Schimper 347 (holotype: B, destroyed; isotypes: JE, PRE, Z).
Coleus schimperi Vatke, Linnaea 37: 320. 1872.
Coleus barbatus var. schimperi (Vatke) Baker in D.Oliver & auct. suc. (eds.), Fl. Trop. Afr. 5: 430. 1900.
Plectranthus semayatensis Cufod., Bull. Jard. Bot. État Bruxelles 33(Suppl.): 838. 1963., non P. schimperi Vatke. Type: Ethiopia, Semajada (Semayata), 12 Oct. 1862, Schimper 618 (holotype: B, destroyed; isotypes: BM, G, JE, K).
Coleus montanus Hochst. ex Ces., Rendiconto Reale Accad. Sci. Fis. 12: 264–266. 1879. Type: Ethiopia, Schimper 2460 (holotype: RO, Erbario Cesati) synon. nov.
Coleus trichophorus Briq., Bull. Herb. Boissier 2: 130. 1894. Type: Ethiopia, Aman-eski, 5 Nov. 1854, Schimper 342 (holotype; G; isotype: P).
Coleus maculatus Gürke, Bot. Jahrb. Syst. 19: 210. 1894. Type: Tanzania, Moshi District: Marangu, Volkens 429 (holotype: B, destroyed; isotypes: BM, G).
Coleus maranguensis Gürke, Bot. Jahrb. Syst. 19: 216. 1894. Type: Tanzania, Moshi District: Marangu, Volkens 630 (holotype: B, destroyed; isotype: BM).
Coleus gomphophyllus Baker, Bull. Misc. Inform. Kew 1895: 225. 1895. Type: Somalia, Golis Range, Lort Phillips s.n. (holotype, K).
Coleus schweinfurthii Baker in D.Oliver & auct. suc. (eds.), Fl. Trop. Afr. 5: 432. 1900., nom. illeg., non C. schweinfurthii Vatke.
Coleus sodalium Baker in D.Oliver & auct. suc. (eds.), Fl. Trop. Afr. 5: 526. 1900. Type: Eritrea, Mogad (Magod) Valley, Schweinfurth & Riva 1810 (holotype: K; isotype: Z).
Coleus somalensis S.Moore, J. Bot. 1901: 265. 1901. Type: Somalia, Gan Liban, Donaldson-Smith s.n. (holotype: BM).
Coleus gallaensis Gürke, Bot. Jahrb. Syst. 38: 169. 1906. Type: Ethiopia, Belana, Ellenbeck 374 (syntype: B, destroyed); Gara Mulata, Ellenbeck 576 (syntype: B, destroyed; neotype: Ethiopia, Harerge Region, Gara Mulata, Gillett 5348 (neotype: K, designated by Ryding (2000)).
Coleus helenae Buscal. & Muschl., Bot. Jahrb. Syst. 49: 487. 1913. Type: Zambia, Lake Bangweulu (Banguelo-See), but in reality from Eritrea, Mai Gogol, near Segeneti, von Aosta 862 (holotype: B, destroyed; neotype: Eritrea, Sageneti, Schweinfurth & Riva 1184 (neotype: FT; isoneotype: BR, K, P; designated by Ryding (2001)).

##### Distribution.

Eritrea to N. Tanzania, SW. Arabian Peninsula. Naturalized in Zimbabwe.

#### Coleus lasianthus

Taxon classificationPlantaeLamialesLamiaceae

Gürke, Bot. Jahrb. Syst. 19: 212. 1894

6A4CF6922F2350408A81D5F944D3E10F

Plectranthus lasianthus (Gürke) Vollesen, Opera Bot. 59: 84. 1980. Types: Tanzania, Kondoa District: Irangi, Fischer 507 (holotype: B, destroyed; neotype selected here: Tanzania, Same District: Mkomazi Game Reserve, Ngurunga Dam, Abdallah, Mboya & Vollesen 96/109, (neotype: K; isoneotype: NHT, designated by Paton in Paton et al. (2009)).
Coleus decumbens Gürke, Bot. Jahrb. Syst. 19: 211. 1894. Types: Tanzania, Moshi District: Lake Chala (Dschallasee), Volkens 327 (syntype: B, destroyed; isosyntype; K) & Kenya, Kwale District: Duruma, Txamtei, Hildebrandt 2320 (syntype: B, destroyed).
Coleus tetensis Baker in D.Oliver & auct. suc. (eds.), Fl. Trop. Afr. 5: 431. 1900.
Plectranthus tetensis (Baker) Agnew, Upland Kenya Wild Fl.: 635. 1974. Type: Mozambique, Lower Zambesi, near Tete, Kirk s.n. (holotype: K).
Coleus menyharthii Briq., Bull. Herb. Boissier, sér. 2, 3: 1074. 1903. Type: Mozambique, Mefídze (Mfidzi) R., towards Katacha, Menyharth 1185 (isotype: Z).
Coleus vagatus E.A.Bruce, Bothalia 6: 227. 1951.
Plectranthus vagatus (E.A.Bruce) Codd in Ross, Fl. Natal: 305. 1971, nom. inval. Type: South Africa, Nelspruit District: Kruger National Park, 2.4 km E of Skukuza, Codd 5489 (holotype: PRE; isotype: K).
Plectranthus erubescens Hedge, Fl. Madag. 175: 216. 1998. Type: Madagascar, Beza Mahafaly Reserve, Phillipson 1833 (holotype: E; isotypes: K, MO, P).

##### Distribution.

Ethiopia to South Africa, Madagascar.

#### Coleus lateriticola

Taxon classificationPlantaeLamialesLamiaceae

(A.Chev.) Phillipson, O. Hooper & A.J. Paton, Kew. Bull. 74, 24: 9. 2019, non C. lateriticola A. Chev. Explor. Bot. Afrique Occ. Franc. 1: 519. 1920, nom. nud.

E1C074129E025731994ADDA93737ACF4

Solenostemon lateriticola A.Chev., J. Bot. (Morot) 22: 121. 1909.
Solenostemon monostachyus subsp. lateriticola (A.Chev.) J.K.Morton, J. Linn. Soc., Bot. 58: 271. 1962.
Plectranthus monostachyus subsp. lateriticola (A.Chev.) B.J.Pollard, Kew Bull. 56: 980. 2001. Type: Guinea, Fonta-Djalon, entre Irebeleya et Timbo, Chevalier 18300 (holotype: P, photo K).

##### Distribution.

Guinea, Ivory Coast, Sierra Leone.

#### Coleus laxus

Taxon classificationPlantaeLamialesLamiaceae

(T.C.Wilson & P.I.Forst) T.C.Wilson & P.I.Forst comb. nov.

8F19CDAAE4535415B648A80A9652C4CF

urn:lsid:ipni.org:names:77201128-1

Plectranthus laxus T.C.Wilson & P.I.Forst., Australian Systematic Botany 31: 440. 2018. Type: Queensland: Cook: Mount Cook walking track, before National Park, 7 April 2016, P.I. Forster PIF45043 & K.R. McDonald (holotype: BRI; isotype: NSW).

##### Distribution.

Australia: Queensland.

#### Coleus leiperi

Taxon classificationPlantaeLamialesLamiaceae

(P.I.Forst.) P.I.Forst. comb. nov.

328C05D97E6953559A1B1D061487F73F

urn:lsid:ipni.org:names:77201129-1

Plectranthus leiperi P.I.Forst., Austrobaileya 4: 177. 1994. Type: Australia, Queensland, Moreton District, Diana’s Bath area, D’Aguilar Range, 9 km E of Somerset Dam, 19 Jun. 1993, P.I.Forster PIF13348 (holotype: BRI, isotype: MEL).

##### Distribution.

Australia: Queensland.

#### Coleus linearifolius

Taxon classificationPlantaeLamialesLamiaceae

(J.K.Morton) A.J.Paton comb. nov.

4970BE69CD6B58EAA2739142666B39C5

urn:lsid:ipni.org:names:77201130-1

Plectranthus linearifolius (J.K.Morton) B.J.Pollard & A.J.Paton, Kew Bull. 61: 229. 2006.
Solenostemon linearifolius J.K.Morton, J. Linn. Soc., Bot. 58: 271. 1962. Type: Guinea, Fiendiou, Pobeguin 1920 (holotype: IFAN; isotype P).

##### Distribution.

Guinea.

#### Coleus livingstonei

Taxon classificationPlantaeLamialesLamiaceae

A.J.Paton nom. nov.

958FCD53B6D25F30B4F6B5426E5F2657

urn:lsid:ipni.org:names:77201131-1

Pycnostachys urticifolia Hook., Bot. Mag. 89: t. 5365. 1863.
Coleus urticifolius (Hook.) Roberty, Bull. Inst. Fondam. Afrique Noire, Sér. A, Sci. Nat. 16: 330. 1954, nom. illeg., non Coleus urticifolius Benth. Type: Malawi, ex Hort. Kew, seeds sent from Mt Zomba by Livingstone (holotype: K).
Pycnostachys pubescens Gürke in H.G.A.Engler, Pflanzenw. Ost-Afrikas, C: 345 (1895), non Coleus pubescens Merr.
Pycnostachys urticifolia var. pubescens (Gürke) Gürke, Bot. Jahrb. Syst. 22: 146 (1895). Types: Mozambique, Gorungosa, Carvalho s.n. (syntype: B, destroyed; isosyntype:, COI); Malawi, without precise locality, Buchanan s.n. (syntype: B, destroyed).

##### Distribution.

Tanzania to South Africa.

#### Coleus longipetiolatus

Taxon classificationPlantaeLamialesLamiaceae

Gürke, Bot. Jahrb. Syst. 19: 214. 1894

3C3F76A4C12A5697AD4D42D747DD34A1

Coleus longipetiolatus Gürke, Bot. Jahrb. Syst. 19: 214. 1894. Type: Tanzania, Usambara Mts, Kwa Mshusa, 15.Aug.1893, Holst 9076 (holotype: B destroyed; isotypes: COI, G, K, M, P, W).
Coleus leptophyllus Baker in D.Oliver & auct. suc. (eds.), Fl. Trop. Afr. 5: 427. 1900.
Plectranthus leptophyllus (Baker) A.J.Paton, Fl. Trop. E. Afr., Lamiac.: 284. 2009. Type: Kenya, Ribe (Ribi) to Galla country, Wakefield s.n. (holotype: K).
Coleus swynnertonii S.Moore, J. Linn. Soc., Bot. 40: 177. 1911.
Plectranthus chiridensis Codd, Fl. Pl. Africa 48: t. 1887. 1984. Type: Zimbabwe, Chirinda, Swynnerton 349 (holotype: BM; isotype K).

##### Distribution.

E. & S. Trop. Africa.

#### Coleus lyratus

Taxon classificationPlantaeLamialesLamiaceae

(A.Chev.) Roberty, Bull. Inst. Fondam. Afrique Noire, Sér. A, Sci. Nat. 16: 330. 1954

5A119CAA57A95D6FB58EF4CBD99A7729

Leocus lyratus A.Chev., J. Bot. (Morot) 22: 126. 1909. Type: Guinea, Fouta-Djalon, Mt Tinka, near Dalaba, Chevalier s.n. (holotype: P).

##### Distribution.

Sierra Leone, Guinea.

##### Notes.

Paton et al. (2013) considered this as a synonym of *P. betonicifolius*, but the west African *C. lyratus* is a larger plant with retrorse, rather than antrorse stem hairs.

#### Coleus macranthus

Taxon classificationPlantaeLamialesLamiaceae

Merr., Philipp. J. Sci. 1(suppl.): 234. 1906

351CFE2C23BC5E978C34183126313B5D

Plectranthus merrillii H.Keng, Fl. Males. 8: 391. 1978. Types: Phillippines, Luzon, Mt Data, Merrill 4502, 4483, 4505 (isosyntypes: K).

##### Distribution.

Philippines (N. Luzon).

#### Coleus maculosus

Taxon classificationPlantaeLamialesLamiaceae

(Lam.) A.J.Paton comb. nov.

FA000AE425CD58E0AABD549EE20E2611

urn:lsid:ipni.org:names:77201133-1

Galeopsis maculosa Lam., Encycl. 2: 601. 1788.
Germanea maculosa Lam., Encycl. 2: 691. 1788.
Plectranthus maculosus (Lam.) Salisb., Prodr. Stirp. Chap. Allerton: 88. 1796. Type: cultivated, from Africa (holotype: P-Lam).
Ocimum punctatum L.f., suppl. Pl.: 275. 1782, non Coleus punctatus Baker.
Plectranthus punctatus (L.f.) L’Hér., Stirp. Nov.: 87. 1788.
Germanea punctata (L.f.) Poir. in J.B.A.M.de Lamarck, Encycl. 2: 763. 1788.
Coleus praetermissus Bullock & Killick, Taxon 6: 239. 1957.
Plectranthus punctatus L’Hér. subsp. punctatus . Fl. Trop. E. Afr., Lamiac.: 350. 2009. Type: “Abyssinia”, cultivated from seed sent by Bruce at Hort. Kew, 1774, (neotype: BM; isoneotype; G; designated by Hedge et al. (1998)).

##### Distribution.

Widespread. Trop. Africa, Madagascar.

#### Coleus maculosus subsp. maculosus

Taxon classificationPlantaeLamialesLamiaceae

8425A5CDD44050BC8E1B56D132352BF9

Coleus glandulosus Hook.f., J. Proc. Linn. Soc., Bot. 7: 211. 1864.
Majana glandulosa (Hook.f.) Kuntze, Revis. Gen. Pl. 2: 524. 1891. Types: Cameroon, Cameroon Mt, Mann 1301 & 1988 (syntypes: K).
Coleus doba Hochst. ex Chiov., Ann. Bot. (Rome) 9: 126. 1911. Types: Ethiopia, Tigre, near Adua, 1852, Schimper 2040 (syntype: P; isosyntype: TUB); Semien, Dabarek, Chiovenda 969 (syntype: FT); Dembia, Gondar, Chiovenda 1500 (syntype: P).
Coleus serrulatus Robyns, Bull. Jard. Bot. État Bruxelles 17: 78. 1943.
Plectranthus serrulatus (Robyns) Troupin & Ayob., Bull. Jard. Bot. Natl. Belg. 55: 299. 1985. Type: DRC, R. Susa, de Witte 2214 (isosyntype: K).

##### Distribution.

Cameroon, to Eritrea and Tanzania, Madagascar.

#### Coleus maculosus subsp. edulis

Taxon classificationPlantaeLamialesLamiaceae

 (Vatke) A.J.Paton comb. nov.

F812ECA72D2859EBB4D4EA3ACBE99D63

urn:lsid:ipni.org:names:77201134-1

Coleus edulis Vatke, Linnaea 37: 319. 1872.
Plectranthus edulis (Vatke) Agnew, Upland Kenya Wild Fl.: 640. 1974.
Plectranthus punctatus subsp. edulis (Vatke) A.J.Paton, Fl. Trop. E. Afr., Lamiac.: 350. 2009. Type: Ethiopia, near Gaffat, 1863, Schimper 1212 (isotype: K, Z, mixed with C. barbatus).
Coleus tuberosus A.Rich., Tent. Fl. Abyss. 2: 185. 1850., nom. illeg., non C. tuberosus (Blume) Benth.
Majana richardiana Kuntze, Revis. Gen. Pl. 2: 524. 1891., nom. illeg., based on above.
Coleus palustris Vatke, Linnaea 37: 319. 1872. Type: Ethiopia, Daschan Meda, 15 Sept. 1863, Schimper 1186 (holotype: B, destroyed; isotypes: BM, JE, K, PRE, W, Z).
Coleus rivularis Vatke, Linnaea 37: 320. 1872. Type: Ethiopia, Gaffat, 25 Sept. 1863, Schimper 1172 (holotype: B, destroyed; isotypes: BM, JE, K, PRE, US, W, Z).
Coleus aquaticus Gürke, Bot. Jahrb. Syst. 19: 218. 1894. Types: Tanzania, Kilimanjaro, Marangu, Volkens 583 (syntype: B, destroyed); Mawenzi, Volkens 860 (syntype: B, destroyed; isosyntype: E).
Coleus clivicola S.Moore, J. Linn. Soc., Bot. 38: 274. 1908. Type: Uganda, E Ruwenzori, Wollaston s.n. (holotype: BM).
Coleus fimbriatus Lebrun & L.Touss., Bull. Jard. Bot. État Bruxelles 17: 79. 1943.
Plectranthus fimbriatus (Lebrun & L.Touss.) Troupin & Ayob., Bull. Jard. Bot. Natl. Belg. 55: 299. 1985. Type: DRC, Mt Mushumangabo, Lebrun 7163 (holotype: BR).

##### Distribution.

Ethiopia, Kenya, Uganda Tanzania, Burundi, Rwanda and DRC.

#### Coleus maculosus subsp. lanatus

Taxon classificationPlantaeLamialesLamiaceae

 (J.K.Morton) A.J.Paton comb. nov.

C24583B1586F57AEBFD093BA2407955D

urn:lsid:ipni.org:names:77201135-1

Fig. 5A

Plectranthus punctatus subsp. lanatus J.K.Morton, J. Linn. Soc., Bot. 58: 268. 1962. Type: Cameroon, Bamenda, near Bambui, Bamenda, Adams GC11322 (holotype: GC).

##### Distribution.

Cameroon.

##### Notes.

Paton et al. (2009, 2013) considered this as a synonym of subsp. edulis, but the prominent indumentum and disjunction warrant separate recognition.

#### Coleus madagascariensis

Taxon classificationPlantaeLamialesLamiaceae

(Pers.) A.Chev., Rev. Int. Bot. Appl. Agric. Trop. 33: 338. 1953

46D6BBCF9C7E593D8AAB9FAC18A7FDED

Ocimum madagascariense Pers., Syn. Pl. 2: 135. 1806.
Plectranthus madagascariensis (Pers.) Benth., Labiat. Gen. Spec.: 37. 1832. Type: Mauritius or Reunion, n.d., Commerson 266 (holotype: P-JU 5706).
Plectranthus hirtus Benth., Labiat. Gen. Spec.: 38. 1832. Type: South Africa, Cape, n.d., Masson s.n. (holotype: BM).
Plectranthus villosus Sieber ex Benth., Labiat. Gen. Spec.: 38. 1832. Type: Mauritus, Sieber, Fl. Maurit. Exsic. 152 (holotype: K; isotype: P).
Plectranthus mauritianus Bojer, Hortus Maurit.: 254. 1837., nom. inval.
Plectranthus pubescens Willd. ex Benth., Linnaea 11: 344. 1837., nom. inval.
Ocimum auricula Forssk. ex Benth. in A.P.de Candolle, Prodr. 12: 43, 68. 1848., nom. inval.

##### Distribution.

Mozambique to South Africa, Mascarenes.

#### Coleus malabaricus

Taxon classificationPlantaeLamialesLamiaceae

Benth. in N.Wallich, Pl. Asiat. Rar. 2: 16. 1830

AD94126416F85E06B8C4B299E29434D1

Plectranthus malabaricus (Benth.) R.H.Willemse, Blumea 25: 509. 1979. Type: India, Travancore, Wall. Num. List 2735A, in Hb. Madr. (holotype: K-W (K001116979)).
Coleus macraei Benth., Labiat. Gen. Spec.: 58. 1832. Type: Ceylon, Macrae s.n., (holotype: K(K000820112)).
Coleus ovatus Benth., Labiat. Gen. Spec.: 57. 1832. Type: India, in Coromandelia, Wright, s.n. (holotype: K (K000820121)).
Coleus leptostachys Benth. in A.P.de Candolle, Prodr. 12: 77. 1848.
Plectranthus malabaricus var. leptostachys (Benth.) R.H.Willemse, Blumea 25: 510. 1979. Type: Ceylon, Walker s.n., (holotype: K (K000820114)).
Coleus walkeri Benth. in A.P.de Candolle, Prodr. 12: 77. 1848. Type: Ceylon, Walker s.n., in Hb, Benth. (holotype: K (K000820113)).

##### Distribution.

S. India, Sri Lanka.

#### Coleus mannii

Taxon classificationPlantaeLamialesLamiaceae

Hook.f., J. Proc. Linn. Soc., Bot. 7: 211. 1864

BC2C6A350429562BBFB7D1196C422EFB

Majana mannii (Hook.f.) Kuntze, Revis. Gen. Pl. 2: 524. 1891.
Solenostemon mannii (Hook.f.) Baker in D.Oliver & auct. suc. (eds.), Fl. Trop. Afr. 5: 422. 1900.
Plectranthus occidentalis B.J.Pollard, Kew Bull. 60: 146. 2005. Type: Mt Cameroon, Mann 1967 (holotype: K).
Solenostemon cymosus Baker in D.Oliver & auct. suc. (eds.), Fl. Trop. Afr. 5: 422. 1900. Type: Cameroon, Mt. Cameroon, Mann 1251 (holotype: K).
Coleus giorgii De Wild., Bol. Soc. Ibér. Ci. Nat. 19: 120. 1920.
Solenostemon giorgii (De Wild.) Champl., Syst. Geogr. Pl. 69: 16. 1999. Type: DRC, Likimi, De Giorgi 1511 (BR: lectotype, designated by Champluvier & Dowsett-Lemaire (1999)). synon. nov.

##### Distribution.

W. Trop. Africa to DRC and Sudan.

#### Coleus marrubatus

Taxon classificationPlantaeLamialesLamiaceae

(J.K.Morton) A.J.Paton comb. nov.

C74111B00143578EB3D31943FDF9BD77

urn:lsid:ipni.org:names:77201136-1

Plectranthus marrubatus J.K.Morton, Kew Bull. 53: 997. 1998 publ. 1999. Type: Ethiopia, Mega, Gillett 14206 (holotype: K; isotype: FT).

##### Distribution.

S. Ethiopia to N. Kenya.

#### Coleus megacalyx

Taxon classificationPlantaeLamialesLamiaceae

(A.J.Paton) A.J.Paton comb. nov.

3F2F63242D1C5B7FAE5752C2EB80DC49

urn:lsid:ipni.org:names:77201137-1

Plectranthus megacalyx A.J.Paton, Fl. Trop. E. Afr., Lamiac.: 316. 2009. Type: Tanzania, Rungwe District: Ngozi, Geilinger 2884 (holotype: K).

##### Distribution.

SW. Tanzania to E. Zambia.

#### Coleus megadontus

Taxon classificationPlantaeLamialesLamiaceae

(P.I.Forst.) P.I.Forst. comb. nov.

038BD6CD3587541B91565E2855625A96

urn:lsid:ipni.org:names:77201138-1

Plectranthus megadontus P.I.Forst., Austrobaileya 4: 181. 1994. Type: Australia, Queensland, Cook District, Rocky River Scrub, eastern fall of McIlwraith Range, 16 June 1992, P.I.Forster PIF10637A, M.C.Tucker & G.Kenning (holotype: BRI; isotypes: CNS, K, L, MEL).

##### Distribution.

Australia: Queensland.

#### Coleus melleri

Taxon classificationPlantaeLamialesLamiaceae

(Baker) A.J.Paton & Phillipson comb. nov.

AF9DC9C2C6F054C085C30AC1CEEE072A

urn:lsid:ipni.org:names:77201145-1

Plectranthus melleri Baker, J. Bot. 20: 243. 1882. Type: Madagascar, between Toamasina (Tamatave) and Antananarivo, July–August 1862, Meller s.n. (holotype: K).
Plectranthus luteus Gürke, Bot. Jahrb. Syst. 28: 468. 1900.
Coleus luteus (Gürke) Staner, Bull. Agric. Congo Belge 25: 426. 1934. Type: Tanzania, Morogoro District: SE Uluguru Mts, Stuhlmann 8790 (holotype: B, destroyed).
Coleus entebbensis S.Moore, J. Bot. 44: 89. 1906. Type: Uganda, Mengo District: Entebbe, Bagshawe 701 (holotype: BM).

##### Distribution.

Liberia, Gabon, Uganda to S. Trop. Africa, Madagascar.

#### Coleus meyeri

Taxon classificationPlantaeLamialesLamiaceae

(Gürke) A.J.Paton comb. nov.

7F9AA388322752AA808E30D59E924BE8

urn:lsid:ipni.org:names:77201139-1

Pycnostachys meyeri Gürke, Abh. Königl. Akad. Wiss. Berlin 1891: 362. 1892. Type: Tanzania, Kilimanjaro, Rua stream, Meyer 279 (holotype: B, destroyed).
Pycnostachys volkensii Gürke in H.G.A.Engler, Pflanzenw. Ost-Afrikas, C: 344. 1895. Type: Tanzania, Kilimanjaro, Mawenzi, Volkens 823 (lectotype: K, designated by Bramley in Paton et al. (2009); isolectotype: BM).
Pycnostachys oblongifolia Baker in D.Oliver & auct. suc. (eds.), Fl. Trop. Afr. 5: 385. 1900. Type: Uganda, Toro District: Ruimi (Wimi) Valley, Scott Elliot 7883 (lectotype: K, designated by Bruce (1940)).
Pycnostachys bowalensis A.Chev., J. Bot. (Morot) 22: 126. 1909. Type: Guinea, Conakry, Fouta-Djalon, Kala, Diaguissa, Oct. 1907, A. Chevalier 18853 (holotype: P; isotype: K).
Pycnostachys longiacuminata Perkins, Notizbl. Bot. Gart. Berlin-Dahlem 8: 76. 1921. Type: Ethiopia, Sidamo, Lake Abassa, (?Lake Margharita), Dec. 1900, Ellenbeck 1728a (holotype: B, destroyed).
Pycnostachys longibracteata De Wild., Pl. Bequaert. 4: 388. 1928. Type: DRC, Ruwenzori, Lanuri Valley, Bequaert 4490 (holotype: BR; K fragment).
Pycnostachys ovoideoconica De Wild., Pl. Bequaert. 4: 396. 1928. Type: DRC, Mukule–Mokoto, Bequaert 6325 (holotype: BR; K, fragment).

##### Distribution.

W. Trop. Africa to Ethiopia and Tanzania.

#### Coleus minor

Taxon classificationPlantaeLamialesLamiaceae

(J.K.Morton) A.J.Paton comb. nov.

2C3E937AADB3541BA7F2DC6E102A95A0

urn:lsid:ipni.org:names:77201140-1

Solenostemon minor J.K.Morton, J. Linn. Soc., Bot. 58: 271. 1962. Type: Nigeria, West Kagoro Hills, Jos Plateau, Killick 15 (holotype: K).
Plectranthus decumbens Hook.f., J. Proc. Linn. Soc., Bot. 7: 210. 1864., non Coleus decumbens Gürke.
Solenostemon decumbens (Hook.f.) Baker in D.Oliver & auct. suc. (eds.), Fl. Trop. Afr. 5: 421. 1900.
Calchas decumbens (Hook.f.) P.V.Heath, Calyx 5: 160. 1997. Type: Cameroon, Mt Cameroon, Mann 2002 (holotype: K).

##### Distribution.

WC. Trop. Africa.

#### Coleus minutiflorus

Taxon classificationPlantaeLamialesLamiaceae

(Ryding) A.J.Paton comb. nov.

0C2FB604406E501CA49D75B20F75DB05

urn:lsid:ipni.org:names:77201141-1

Plectranthus minutiflorus Ryding, Nordic J. Bot. 20: 43. 2000. Type: Ethiopia, Sidamo, ± 35 km from the main road Yabelo–Mega, on road to Arero, Friis, Bidgood, L. & W. Malaku & Gashaw 8374 (holotype: C; isotypes: ETH, K).

##### Distribution.

Ethiopia to N. Kenya.

#### Coleus minutus

Taxon classificationPlantaeLamialesLamiaceae

(P.I.Forst.) P.I.Forst. comb. nov.

18223A189CC65C0F9CB76AEFF6FAC9F0

urn:lsid:ipni.org:names:77201142-1

Plectranthus minutus P.I.Forst., Austrobaileya 3: 734. 1992. Type: Australia, Queensland, Cook District, Mount Muligan, c 40 km NW of Dimbulah, c. 0.5 km SE of Dam on top of mountain, 17 April 1985, J.R.Clarkson 5902 (holotype: BRI; isotypes: CNS, MEL).

##### Distribution.

Australia: Queensland.

#### Coleus mirabilis

Taxon classificationPlantaeLamialesLamiaceae

Briq., Bot. Jahrb. Syst. 19: 183. 1894

60BAB9AC8F24557F81D9F0BEE60CA738

Fig. 5B

Coleus mirabilis var. hypisodontus Briq., Bot. Jahrb. Syst. 19: 184. 1894., nom. inval.
Ascocarydion mirabile (Briq.) G.Taylor, J. Bot. 69(suppl. 2): 162. 1931.
Burnatastrum mirabile (Briq.) P.V.Heath, Calyx 4: 175. 2001.
Plectranthus mirabilis (Briq.) Launert, Mitt. Bot. Staatssamml. München 7: 299. 1968. Type: Angola, Malanje (Malandsche), Mar. 1880, Mechow 489 (lectotype: Z isolectotype: W; designated by Codd (1975)).
Coleus mirabilis var. buchnerianus Briq., Bot. Jahrb. Syst. 19: 184. 1894. Types: Angola, Moma, N of Malanje (Malandsche), Buchner 81, 82, 83, 84 & 85 (syntypes: B, destroyed).
Coleus mirabilis var. mecjowianus Briq., Bot. Jahrb. Syst. 19: 184. 1894. Type: Angola, Lumsuneja stream, between Malanje (Malandsche) and Cuango (Quango) rivers, June1880, Mechow s.n. (isotype: Z).
Coleus mirabilis var. poggeanus Briq., Bot. Jahrb. Syst. 19: 184. 1894. Type: Congo, R.Lulua, 9°30'S, Pogge 350 (holotype: B, destroyed).
Coleus leucophyllus Baker, Bull. Misc. Inform. Kew 1895: 292. 1895. Type: Zambia, near Mwero, W of Lake Tanganyika, 1894, Carson 26 (holotype: K).

##### Distribution.

S. DRC to NE. Namibia.

#### Coleus mirus

Taxon classificationPlantaeLamialesLamiaceae

(S.T.Blake) P.I.Forst. & T.C.Wilson comb. nov.

90CA9B1F6A835041A5447A6886480D6F

urn:lsid:ipni.org:names:77201143-1

Plectranthus mirus S.T.Blake, Contr. Queensland Herb. 9: 22. 1971. Type: Australia, Queensland, White Cliff Point NW of Cairns, 21 May 1962, S.T.Blake 21791 (holotype: BRI).

##### Distribution.

Australia: Queensland.

#### Coleus mitis

Taxon classificationPlantaeLamialesLamiaceae

(R.A.Clement) A.J.Paton comb. nov.

866971CB54135D5187712EA321F0E187

urn:lsid:ipni.org:names:77201144-1

Anisochilus mitis R.A.Clement, Edinburgh J. Bot. 50: 35. 1993. Type: Bhutan, Khoma, open spaces, 1219 m, 21 July 1949, Ludlow, Sherriff & Hicks 20927 (holotype: BM).

##### Distribution.

E. Himalaya.

#### Coleus modestus

Taxon classificationPlantaeLamialesLamiaceae

(Baker) Robyns & Lebrun, Ann. Soc. Sci. Bruxelles, Sér. B 49: 106. 1929

921DB52C900C5A4EB562F84B44B407A1

Plectranthus modestus Baker, Bull. Misc. Inform. Kew 1895: 72. 1895.
Englerastrum modestum (Baker) T.C.E.Fr., Notizbl. Bot. Gart. Berlin-Dahlem 9: 76. 1924. Type: Zambia, Mbala District: Tanganyika Plateau, 1889, Carson s.n. (holotype: K).

##### Distribution.

SW. Tanzania to Zambia.

#### Coleus mollis

Taxon classificationPlantaeLamialesLamiaceae

Benth. in A.P.de Candolle, Prodr. 12: 77. 1848

211CCC4C4F5452D4A06F0CADFF1CA049

Plectranthus crameri R.H.Willemse, Blumea 25: 510. 1979. Type: Ceylon, Walker s.n., in Hb. Benth. (lectotype: K (K000820116), designated by Cramer (1978)).

##### Distribution.

Sri Lanka.

#### Coleus monostachyus

Taxon classificationPlantaeLamialesLamiaceae

(P.Beauv.) A.J.Paton comb. nov.

745CBD14B860517AA90EBDC219920D59

urn:lsid:ipni.org:names:77201146-1

Ocimum monostachyum P.Beauv., Fl. Oware 2: 60. 1818.
Plectranthus palisotii Benth., Labiat. Gen. Spec.: 39. 1832., nom. illeg.
Solenostemon monostachyus (P.Beauv.) Briq. in H.G.A.Engler & K.A.E.Prantl, Nat. Pflanzenfam. 4(3a): 359. 1897.
Solenostemon ocymoides var. monostachyus (P.Beauv.) Baker in D.Oliver & auct. suc. (eds.), Fl. Trop. Afr. 5: 421. 1900.
Plectranthus monostachyus (P.Beauv.) B.J.Pollard, Kew Bull. 56: 980. 2001. Type: Benin, Palisot de Beauvois s.n. (holotype: G).

#### Coleus monostachyus

Taxon classificationPlantaeLamialesLamiaceae

(P.Beauv.) A.J.Paton subsp. monostachyus

A2842C217AF15A2EAD8B73B80EDA21AC

Solenostemon ocymoides Schumach. & Thonn. in C.F.Schumacher, Beskr. Guin. Pl.: 271. 1827.
Coleus africanus Benth., Labiat. Gen. Spec.: 54. 1832., nom. illeg.
Solenostemon africanus Briq., Bot. Jahrb. Syst. 19: 181. 1894. Type: “Guinea”, Thonning 92 (lectotype: C, designated by Pollard & Paton (2001); Isert & Thonning herbarium, microf. 102: III. 7).
Solenostemon monostachyus var. amplifrons Briq., Bull. Herb. Boissier, sér. 2, 6: 826. 1906. Type: DRC, Bangala, 26 May 1888, Hens Ser. C 33 (holotype: Z).
Solenostemon godefroyae N.E.Br., Bot. Mag. 139: t. 8511. 1913. Type: illustration cited above (lectotype: illustration designated here).
Coleus godfroyae Godfroy-Lebeuf, Cat Pl. Nouv.: 2 1903. Type: not seen. Protologue of S. godefoyae suggests the name C. godfroy exists (with drawing), but this manuscript has not been seen.

##### Distribution.

W. Trop. Africa to S. Chad and Cabinda.

#### Coleus monostachyus

Taxon classificationPlantaeLamialesLamiaceae

(P.Beauv.) A.J.Paton subsp. marrubiifolius (Brenan) A.J.Paton comb. nov.

573CB6F3E3F15EFF97D8343A400B0849

urn:lsid:ipni.org:names:77201147-1

Solenostemon monostachyus var. marrubiifolius Brenan, Kew Bull. 5: 231. 1950.
Solenostemon monostachyus subsp. marrubiifolius (Brenan) J.K.Morton, J. Linn. Soc., Bot. 58: 272. 1962.
Plectranthus monostachyus subsp. marrubiifolius (Brenan) B.J.Pollard, Kew Bull. 56: 980. 2001. Type: Nigeria, Ondo Province, Carter’s Peak, Idanre Hills, Keay FHI 22585 (holotype: K).
Solenostemon gouanensis A.Chev., Explor. Bot. Afrique Occ. Franç. 1: 517. 1920., nom. nud.

##### Distribution.

W. & WC. Trop. Africa.

#### Coleus monostachyus

Taxon classificationPlantaeLamialesLamiaceae

(P.Beauv.) A.J.Paton subsp. perennis (J.K. Morton) A.J.Paton comb. nov.

EDD774B6B219534FB89E817061419B18

urn:lsid:ipni.org:names:77201148-1

Solenostemon monostachyus (P.Beauv.) Briq subsp. perennis J.K.Morton, J. Linn. Soc., Bot. 58: 272. 1962.
Plectranthus monostachyus (P.Beauv.) Briq subsp. perennis (J.K.Morton) B.J.Pollard, Kew Bull. 56: 981. 2001. Type: Ghana, Northern Province, Damongo Scarp, Morton A1242 (holotype: GC, not seen).

##### Distribution.

Sierra Leone, Ivory Coast, Ghana and Nigeria.

#### Coleus mutabilis

Taxon classificationPlantaeLamialesLamiaceae

(Codd) A.J.Paton comb. nov.

0C2D11AC139154ECB01F183EE93ACC9A

urn:lsid:ipni.org:names:77201149-1

Plectranthus mutabilis Codd, Bothalia 11: 404. 1975. Type: South Africa, Transvaal, Pietersburgh District, farm Bulbul, eastern end of Blouberg, Codd 7953 (holotype: PRE).

##### Distribution.

South Africa: Northern Prov.

#### Coleus myrianthellus

Taxon classificationPlantaeLamialesLamiaceae

Briq., Mém. Soc. Bot. France 8: 290. 1917

2B8FB18BBCC258FCA6241317665AA0E5

Coleus myrianthellus Briq., Mém. Soc. Bot. France 8: 290. 1917. Type: Central African Republic, Gribingui, bassin de la Moyenne Kododo, 29 Nov. 1902, Chevalier 6454 (holotype: P).

##### Distribution.

Central African Republic.

##### Notes.

Related to C. splendidus. A revision of this group is required.

#### Coleus namuliensis

Taxon classificationPlantaeLamialesLamiaceae

E.Downes & I.Darbysh., Blumea 62: 169. 2017

C220D1D74EAB535992E934978CB7195B

Coleus namuliensis E.Downes & I.Darbysh., Blumea 62: 169. 2017. Type: Mozambique, Zambezia Province, Mt Namuli, small peak 5 km west of highest peak, Harris 237 (holotype: K (K000962083); isotype K(K000962089), LMA).

##### Distribution

. Mozambique.

#### Coleus neochilus

Taxon classificationPlantaeLamialesLamiaceae

(Schltr.) Codd, Bothalia 7: 432. 1961

73DC9EA214325C039147129FD25C2F61

Plectranthus neochilus Schltr., J. Bot. 34: 394. 1896. Type: South Africa, Mpumalanga, Rimero Creek near Baberton, 1890, Galpin 968 (holotype: K; isotypes: BOL, GRA, NH).
Coleus schinzii Gürke, Bull. Herb. Boissier 6: 555. 1898. Type: Namibia, Ovamboland, Tsumeb, Schinz 56 (holotype: Z).
Coleus palliolatus S.Moore, J. Bot. 38: 464. 1900. Type: Zimbabwe, Bulawayo, Jan.1898, Rand 144 (holotype: BM).

##### Distribution.

S. Trop. & South Africa.

#### Coleus nepetifolius

Taxon classificationPlantaeLamialesLamiaceae

(Baker) A.J.Paton comb. nov.

613E0B752BBD582AAC93863A4E9F116B

urn:lsid:ipni.org:names:77201150-1

Pycnostachys nepetifolia Baker in D.Oliver & auct. suc. (eds.), Fl. Trop. Afr. 5: 383. 1900. Type: Kenya, Nandi/North Kavirondo District: Nandi Range, Scott Elliot 6471 (holotype: K; isotype: BM).

##### Distribution.

Kenya.

#### Coleus niamniamensis

Taxon classificationPlantaeLamialesLamiaceae

(Gürke) A.J.Paton comb. nov.

165F40FF8A9A5F03ADB887561BDA7A3D

urn:lsid:ipni.org:names:77201151-1

Pycnostachys niamniamensis Gürke, Bot. Jahrb. Syst. 22: 145. 1895. Type: Sudan, Niamniam, east of R. Huuh, Schweinfurth 3750a (holotype: B, destroyed; isotype: UPS, K, fragment).
Pycnostachys decussata Baker in D.Oliver & auct. suc. (eds.), Fl. Trop. Afr. 5: 382. 1900. Type: Kenya, ‘Kavirondo’, Scott Elliot 7155 (holotype: K).
Pycnostachys petherickii Baker in D.Oliver & auct. suc. (eds.), Fl. Trop. Afr. 5: 383. 1900. Type: Sudan, White Nile, Petherick s.n. (holotype: K).
Pycnostachys lindblomii T.C.E.Fr., Notizbl. Bot. Gart. Berlin-Dahlem 11: 24. 1930. Type: Kenya, North Kavirondo District: Elgon, Kitosh, Lindblom s.n. (holotype: S; K, fragment).

##### Distribution.

Sudan to Kenya.

#### Coleus nigericus

Taxon classificationPlantaeLamialesLamiaceae

(Alston) A.J.Paton comb. nov.

084225E8AF97531AA639D48B734630B0

urn:lsid:ipni.org:names:77201152-1

Englerastrum nigericum Alston, Bull. Misc. Inform. Kew 1926: 298. 1926. Type: Nigeria, Northern Nigeria, Katagum District, Dalziel 109 (holotype: K).
Plectranthus denudatus A.Chev. ex Hutch. & Dalziel, Fl. W. Trop. Afr. 2: 288. 1931.
Coleus denudatus (A.Chev. ex Hutch. & Dalziel) Robyns, Mem. Inst. Roy. Colon. Belge, Sect. Sci. Nat. (4°): 50. 1936. Type: Senegal, Diola (Casamance), Flory-Feygan, Chevalier 2815 (holotype: P).

##### Distribution.

W. Trop. Africa.

##### Notes.

Non *C. nigericus* A.Chev., nom. nud.

#### Coleus nitidus

Taxon classificationPlantaeLamialesLamiaceae

(P.I.Forst.) P.I.Forst. comb. nov.

769DF8D0DB07503CB8F5C0F6BCD1CF67

urn:lsid:ipni.org:names:77201153-1

Plectranthus nitidus P.I.Forst., Austrobaileya 3: 736. 1992. Type: Australia, New South Wales, cultivated at Byron Bay, from plant collected at Terania Creek, Nightcap Range, P. Hardwick s.n. (holotype: BRI; isotypes: CNS, K, L, MEL, NSW).

##### Distribution.

E. Australia.

#### Coleus niveus

Taxon classificationPlantaeLamialesLamiaceae

(Hiern) A.J.Paton comb. nov.

9CDF0B6134B95023B81AB795012D2D21

urn:lsid:ipni.org:names:77201154-1

Solenostemon niveus Hiern, Cat. Afr. Pl. 1: 864. 1900 (August). Type: Angola, Bumbo, Sierra de Xella, Welwitsch 5619 (holotype: LISU; isotype: BM).
Coleus orbicularis Baker in D.Oliver & auct. suc. (eds.), Fl. Trop. Afr. 5: 437. 1900. (December). Type: Angola, Benguella Prov., Welwitsch 5619, (holotype: K).

##### Distribution.

Angola.

#### Coleus nyikensis

Taxon classificationPlantaeLamialesLamiaceae

Baker in D.Oliver & auct. suc. (eds.), Fl. Trop. Afr. 5: 439. 1900

8A8084F5BF975E638D75BF627E1F921A

Plectranthus malawiensis B.Mathew, Kew Bull. 31: 174. 1976. Type: Malawi, Nyika Plateau, July 1896, Whyte s.n. (holotype: K).

##### Distribution.

SE. Kenya to N. Zambia.

#### Coleus omissus

Taxon classificationPlantaeLamialesLamiaceae

(P.I.Forst) P.I.Forst. comb. nov.

882A9F36D6D854D2A4D296F546A1FB0C

urn:lsid:ipni.org:names:77201155-1

Plectranthus omissus P.I.Forst., Austrobaileya 3: 731. 1992. Type: Australia, Queensland, Wide Bay District, Mudlow Gap, Beauty Spot 11, Timber reserve 26, 8 km N of Kilkivan, 21 Feb. 1989, P.I. Forster PIF4977 (holotype: BRI; isotypes: CANB, K, MO).

##### Distribution.

Australia: Queensland.

#### Coleus orthodontus

Taxon classificationPlantaeLamialesLamiaceae

(Gürke) A.J.Paton comb. nov.

FD831479F1425B89B8A29B4F200643BF

urn:lsid:ipni.org:names:77201156-1

Fig. 5D

Pycnostachys orthodonta Gürke in H.G.A.Engler, Pflanzenw. Ost-Afrikas, C: 345. 1895. Type: Mozambique, Manica, near Catandica (Gouveia), 1884, Carvalho s.n. (holotype: B, destroyed; isotypes: COI, K frag.).
Pycnostachys cyanea Gürke in H.G.A.Engler, Pflanzenw. Ost-Afrikas, C: 345. 1895. Type: Tanzania, Mpwapwa, Tubugwe, Stuhlmann 213 (lectotype: B, destroyed; designated by Bramley in Paton et al. (2009)).
Pycnostachys leptophylla Baker, Bull. Misc. Inform. Kew 1898: 161. 1898. Type: Malawi, Shire Highlands, 1891, Buchanan 873 (lectotype: K; designated by Bramley in Paton et al. (2009)).
Pycnostachys hanningtonii Baker in D.Oliver & auct. suc. (eds.), Fl. Trop. Afr. 5: 384. 1900. Type: Tanzania, Dodoma Dist., Ugogo, n.d., Hannington s.n. (holotype: K).
Pycnostachys bussei Gürke, Bot. Jahrb. Syst. 36: 131. 1905. Type: Tanzania, Lindi Dist., near Nguya, n.d., Busse 2834 (holotype: B, destroyed; isotypes: BR, K frag.).

##### Distribution.

Tanzania to S. Trop. Africa.

#### Coleus otostegioides

Taxon classificationPlantaeLamialesLamiaceae

(Gürke) A.J.Paton comb. nov.

38BDAF7F870B595D996D24C2D0ADB186

urn:lsid:ipni.org:names:77201157-1

Capitanya otostegioides Gürke, Bot. Jahrb. Syst. 21: 106. 1895.
Plectranthus otostegioides (Gürke) Ryding, Kew Bull. 54: 126. 1999. Types: Eritrea, upper Lebka valley, Heuglin s.n. (syntype: B, destroyed) & Müller-Prosko-Capitany s.n. (syntype: B, destroyed) & Tanzania, Kilimanjaro, Volkens 492 (syntype: B, destroyed); neotype: Tanzania, Arusha District: Ngaserai Plain, Richards & Arasululu 26472 (neotype: K, designated by Ryding (1999)).

##### Distribution.

NE. Trop. Africa to N. Tanzania.

#### Coleus pallidus

Taxon classificationPlantaeLamialesLamiaceae

(Wall.) A.J.Paton comb. nov.

4D7DA40F660D5F87974BC9D9120D2E61

urn:lsid:ipni.org:names:77201158-1

Anisochilus pallidus Wall., Pl. Asiat. Rar. 2: 18. 1830. Type: Burma, Taong Dong, 26 Nov. 1826, Wallich Cat. 2754, (lectotype: K (K000674759); isolectotypes: G-DC, K-W (K001117031), designated by Suddee and Paton (2009)).

##### Distribution.

Nepal to SC. China and Indo-China.

#### Coleus paniculatus

Taxon classificationPlantaeLamialesLamiaceae

Benth. in N.Wallich, Pl. Asiat. Rar. 2: 16. 1830

83E0C0F7F07E5E25841EB3389DA34F55

Majana paniculata (Benth.) Kuntze, Revis. Gen. Pl. 2: 524. 1891.
Solenostemon paniculatus (Benth.) Guillaumet & A.Cornet, Adansonia, n.s., 15: 525. 1976. Type: India, Dindygul, Herb. Wight in Wall. Cat. 2734 (syntypes: K (K000820126); K-W(K001116978).
Coleus glabratus Benth., Labiat. Gen. Spec.: 58. 1832.
Plectranthus glabratus (Benth.) Alston in H.Trimen, Handb. Fl. Ceylon 6(suppl.): 236. 1931. Type: India, Madras, Wight s.n. ((holotype: K (K000820127)).
Coleus wightii Benth., Labiat. Gen. Spec.: 58. 1832. Type: India, Nilgiris, Wight s.n. (holotype: K (K000820125)).
Plectranthus coleoides Benth. in A.P.de Candolle, Prodr. 12: 64. 1848. Type: India, Nilgiris, Perrottet s.n. (syntypes: 2 shts. G-DC; isosyntype: K (K000820123), P (P00720715).
Plectranthus bernardii Doan in H.Lecomte, Fl. Indo-Chine 4: 949. 1936 nom. inval. no Latin descr.

##### Distribution.

S. India to S. Indo-China.

#### Coleus parishii

Taxon classificationPlantaeLamialesLamiaceae

(Hook.f.) A.J.Paton comb. nov.

C987EA16264F5219B0372FEA6A0595D2

urn:lsid:ipni.org:names:77201159-1

Plectranthus parishii Hook.f., Fl. Brit. India 4: 622. 1885. Type: Burma, Tenasserim, Moulmein, Parish 444 (holotype: K).
Calamintha esquirolii H.Lév., Repert. Spec. Nov. Regni Veg. 8: 450. 1910.
Coleus esquirolii (H.Lév.) Dunn, Notes Roy. Bot. Gard. Edinburgh 8: 158. 1913. Type: China, Kweichow, Shin- gny-hien, chemin pierreux de Sy-Koua-Tong, Nov. 1906, Esquirol 1058 (holotype: E).
Coleus mucosus Hayata, J. Coll. Sci. Imp. Univ. Tokyo 30(1): 225. 1911. Types: Kodensho, Oct. 1906, Kawakami & Mori 1755 (TI: syntype); Kashito, Aug. 1905, Nakahara 302 (TI: syntype); Banchoryo, Oct. 1905, Nakahara 582, Oct. 1905 (TI: syntype); Kodensho, Oct. 1905, Nagasawa 764 (TI: syntype)

##### Distribution.

Myanmar to SC. China and Taiwan.

##### Notes.

See Suddee et al. (2004) for discussion of *C. mucosus*.

#### Coleus parvicalyx

Taxon classificationPlantaeLamialesLamiaceae

(A.J.Paton) A.J.Paton comb.nov.

654403345AC451A79106F2F673D554F9

urn:lsid:ipni.org:names:77201160-1

Plectranthus parvicalyx A.J.Paton, Fl. Trop. E. Afr., Lamiac.: 316. 2009. Type: Tanzania, Rungwe District: Kiejo Volcano, Hepper, Field & Mhoro 5389 (holotype: K).

##### Distribution.

SW. Tanzania.

#### Coleus penicillatus

Taxon classificationPlantaeLamialesLamiaceae

(A.J.Paton) A.J.Paton comb. nov.

309EED11F8CA595D9DEFF1464F9CA9C5

urn:lsid:ipni.org:names:77201161-1

Plectranthus penicillatus A.J.Paton, Fl. Trop. E. Afr., Lamiac.: 325. 2009. Type: Zambia, Mbuzi–Kaluza, Greenway & Trapnell 5627 (holotype: K).

##### Distribution.

S. Tanzania to E. Zambia.

#### Coleus pentheri

Taxon classificationPlantaeLamialesLamiaceae

Gürke, Ann. K. K. Naturhist. Hofmus. 20: 48. 1905

CAFDFA700761557F83C18D557F2CD874

Plectranthus pentheri (Gürke) van Jaarsv. & T.J.Edwards, Bothalia 27: 5. 1997. Type: South Africa, Mpumalanga, Rimero Creek near Baberton, 1890, Galpin 968 (holotype: K; isotypes: BOL, GRA, NH).

##### Distribution.

SW. Zimbabwe to South Africa.

#### Coleus perrieri

Taxon classificationPlantaeLamialesLamiaceae

(Hedge) A.J.Paton & Phillipson comb. nov.

EB1F1536B9E15629A8BB5DB500E70364

urn:lsid:ipni.org:names:77201162-1

Plectranthus perrieri Hedge, Fl. Madag. 175: 199. 1998. Type: Madagascar, Ampandrandava, Seyrig 637 (holotype: P).

##### Distribution.

Madagascar.

#### Coleus persoonii

Taxon classificationPlantaeLamialesLamiaceae

Benth., Labiat. Gen. Spec.: 55. 1832

C1D7573655165365B03267CCEF60E0BD

Ocimum paniculatum Pers., Syn. Pl. 2: 135. 1806, non Coleus paniculatus Benth.
Solenostemon paniculatus (Pers.) Guillaumet & Cornet, Adansonia n.s., 15: 525. 1976
Plectranthus persoonii (Benth.) Hedge, Fl. Madag. 175: 222. 1998. Type: Madagascar, Commerson *s.n.* (holotype: P).
Coleus goudotii Briq., Bull. Herb. Boissier 2: 126. 1894.
Solenostemon goudotii (Briq.) Guillaumet & A.Cornet, Adansonia, n.s., 15: 525. 1976. Type: Madagascar, Goudot s.n. (G, holotype).

##### Distribution.

Madagascar.

#### Coleus petiolatissimus

Taxon classificationPlantaeLamialesLamiaceae

Briq., Mém. Soc. Bot. France 8: 291. 1917

37784DC39BC854AC8E63993C1D842142

Coleus petiolatissimus Briq., Mém. Soc. Bot. France 8: 291. 1917. Type: Central African Republic (Bangui), 16 Aug. 1902, Chevalier 5214 bis. (holotype: P).

##### Distribution.

Central African Republic.

##### Notes.

Related to *P. bojeri*. Complex in need of revision. 

#### Coleus petraeus

Taxon classificationPlantaeLamialesLamiaceae

(Backer ex Adelb.) A.J.Paton comb. nov.

46AFA218EC5654B58610F3849FAA9305

urn:lsid:ipni.org:names:77201163-1

Plectranthus petraeus Backer ex Adelb., Reinwardtia 3: 152. 1954. Type: Java, Idjin Plateau, Sempolm, Backer 36387 (holotype: L).

##### Distribution.

E. Java.

#### Coleus petricola

Taxon classificationPlantaeLamialesLamiaceae

(J.Mathew & B.J.Conn) A.J.Paton comb. nov.

249D7D2F54A550A1B05818CADAED43CA

urn:lsid:ipni.org:names:77201164-1

Plectranthus petricola J.Mathew & B.J.Conn, Telopea 20: 179. 2017. Type: India: Kerala: Idukki District, 10 km away from Udumbanchola, Chemmannar Hills, 20 Feb 2010, J. Mathew 4417 (holotype: TBGT; isotype: MSSRF).

##### Distribution.

India, endemic to Western Ghats.

#### Coleus phulangkaensis

Taxon classificationPlantaeLamialesLamiaceae

(Suddee, Suphuntee & Saengrit) Suddee comb. nov.

385942F0C5265C21A5F391A81ECEC515

urn:lsid:ipni.org:names:77201165-1

Plectranthus phulangkaensis Suddee, Suphuntee & Saengrit, Thai Forest Bull., Bot. 42: 6. 2014 (Dec 2014) Type: Thailand. Nakhon Phanom, Ban Phaeng District, Phu Langka National Park, 9 Oct. 2013, Suddee, Puudjaa, Rueangruea, Kiewbang, Hemrat & Pansamrong 4588 (holotype: BKF; isotypes: BKF).

##### Distribution.

Thailand.

#### Coleus plantagineus

Taxon classificationPlantaeLamialesLamiaceae

(Hook.f.) A.J.Paton comb. nov.

8675B0F033865921B928AB0193DDCC63

urn:lsid:ipni.org:names:77201166-1

Anisochilus plantagineus Hook.f., Fl. Brit. India 4: 628. 1885. Type: India, Deccan Peninsula, Mysore, Bababoodan Hills, Dalzell s.n. (lectotype: K(K000674770); isolectotype K(K000674771), designated by Suddee & Paton (2009).

##### Distribution.

SW. India.

#### Coleus platyphyllus

Taxon classificationPlantaeLamialesLamiaceae

(A.J.Paton) A.J.Paton comb. nov.

536ECA77945C5EA587F771753CE0189F

urn:lsid:ipni.org:names:77201167-1

Plectranthus platyphyllus A.J.Paton, Fl. Trop. E. Afr., Lamiac.: 331. 2009. Type: Tanzania, Iringa District: Mafinga (Sao Hill) to Madibira road, Richards 17354 (holotype: K; isotype: EA).

##### Distribution.

SW. Tanzania.

#### Coleus pobeguinii

Taxon classificationPlantaeLamialesLamiaceae

Hutch. & Dalziel, Fl. W. Trop. Afr. 2: 291. 1931

99B20744A9955CEEBA70A198C51B49A0

Leocus pobeguinii (Hutch. & Dalziel) J.K.Morton, J. Linn. Soc., Bot. 58: 270. 1962.
Plectranthus pobeguinii (Hutch. & Dalziel) B.J.Pollard, Kew Bull. 64: 260. 2009. Type: Guinea (Conakry), Kouroussa, Pobéguin 556 (P, lectotype, designated by Pollard and Paton (2009)).

##### Distribution.

W. Trop. Africa.

#### Coleus polystachys

Taxon classificationPlantaeLamialesLamiaceae

(Benth.) A.J.Paton comb. nov.

1A2BFD0526605AEE9EFD7DF93FB3A233

urn:lsid:ipni.org:names:77201168-1

Anisochilus polystachys Benth. in N.Wallich, Pl. Asiat. Rar. 2: 19. 1830.
Stiptanthus polystachyus (Benth.) Briq. in H.G.A.Engler & K.A.E.Prantl, Nat. Pflanzenfam. 4(3a): 352. 1897. Type: Nepal, Hetoundah (Hetauda), 14 Dec. 1820, Wall. Cat. 2755 (lectotype: K (K000674760); isolectotypes: BM, E, G-DC, K (K000674761), K-W(K001117032), designated Suddee & Paton (2009)).

##### Distribution.

Nepal to Bangladesh.

#### Coleus porcatus

Taxon classificationPlantaeLamialesLamiaceae

(van Jaarsv. & P.J.D.Winter) A.J.Paton comb. nov.

74A21B1A193F5CEB818D5E9869603E3F

urn:lsid:ipni.org:names:77201169-1

Plectranthus porcatus van Jaarsv. & P.J.D.Winter, Bothalia 35: 170. 2005. Type: South Africa, Sekukuniland, N. Leolo Range, Farm de Kamp, P.J.D. Winter 6725 (holotype: PRE; isotype: NBG).

##### Distribution.

South Africa: Limpopo (Leolo Mts.).

#### Coleus porphyranthus

Taxon classificationPlantaeLamialesLamiaceae

(T.J.Edwards & N.R.Crouch) A.J.Paton comb. nov.

1FD5B1637A8958D08A1AAEAE1EDAC29D

urn:lsid:ipni.org:names:77201170-1

Fig. 5C

Plectranthus porphyranthus T.J.Edwards & N.R.Crouch, Kew Bull. 55: 459. 2000. Type: Zimbabwe, Masvingo Dist., Richmond Farm, Harare road, 4 km from Masvingo, Dec. 1997, Crouch 800 (holotype: NU; isotypes: E, K, PRE, SRGH).

##### Distribution.

S. Zimbabwe.

#### Coleus prittwitzii

Taxon classificationPlantaeLamialesLamiaceae

(Perkins) A.J.Paton comb. nov.

7736BD6436AB5CE7A81B3B01A8F55A57

urn:lsid:ipni.org:names:77201171-1

Pycnostachys prittwitzii Perkins, Notizbl. Bot. Gart. Berlin-Dahlem 8: 68. 1921. Type: Tanzania, Iringa District: Ndembera flood plain, near Gominyi, Prittwitz & Gaffron 28 (holotype: B, destroyed; K, fragment).

##### Distribution.

SW. Tanzania to N. Zambia.

#### Coleus prostratus

Taxon classificationPlantaeLamialesLamiaceae

(Gürke) A.J.Paton comb. nov.

2C7B73AF8B1F50C78C99EB79C76CEDFC

urn:lsid:ipni.org:names:77201172-1

Plectranthus prostratus Gürke, Bot. Jahrb. Syst. 19: 206. 1894. Types: Tanzania, Pangani, Volkens 484 (syntype: B, destroyed; isosyntype: G) & North Mara District: Ukira, Fischer 497 (syntype: B, destroyed).
Plectranthus quadridentatus Schweinf. ex Baker in D.Oliver & auct. suc. (eds.), Fl. Trop. Afr. 5: 409. 1900. Type: Eritrea, Mount Alam Kale, NW of Aidereso, Schweinfurth & Riva 2086 (holotype: K).
Plectranthus margeritae Buscal. & Muschl., Bot. Jahrb. Syst. 49: 485. 1913. Type: Neumann Camp (Kenya, but in reality based on material from E or NE Africa and Yemen), von Aosta 1688 (holotype: B, destroyed); neotype: Eritrea, Nefasit, base of Mt Bizen, Ryding et al. 2095 (neotype: C; isoneotypes: ASMU, ETH, UPS, designated by Ryding (2001)).
Plectranthus ugandensis Lye, Norweg. J. Bot. 20: 57. 1973, nom. illeg., non P. ugandensis S.Moore.

##### Distribution.

Yemen, Eritrea to Tanzania.

#### Coleus psammophilus

Taxon classificationPlantaeLamialesLamiaceae

(Codd) A.J.Paton comb. nov.

6860B521635E59898D92E46AD0FBF19A

urn:lsid:ipni.org:names:77201173-1

Plectranthus psammophilus Codd, Bothalia 11: 405. 1975. Type: South Africa, KwaZulu-Natal, Makatini Flats, Jan.1964, Strey 5779 (holotype: PRE; isotypes: BR, K, M, NH).

##### Distribution.

Mozambique to N. KwaZulu-Natal.

#### Coleus pseudospeciosus

Taxon classificationPlantaeLamialesLamiaceae

(Buscal. & Muschl.) A.J.Paton comb. nov.

0042D4643D8F57B99382B0387ACE977D

urn:lsid:ipni.org:names:77201174-1

Pycnostachys pseudospeciosa Buscal. & Muschl., Bot. Jahrb. Syst. 49: 486. 1913. Type: Zambia, Lake Bangweulu, n.d, D’Aosta 1002 (holotype: B, destroyed; lectotype: K, designated by Ryding (2001)).
Pycnostachys ballotoides Perkins, Notizbl. Bot. Gart. Berlin-Dahlem 8: 72. 1921. Type: Congo, Katanga, Mt Senga, Kassner 2930a (holotype: B, destroyed).
Pycnostachys mausaensis Gürke, Notes Fl. Katanga 7: 47. 1921. Type: Congo, Katanga, Mt Senga, 31.May 1908, Kassner 2920a (holotype: BR; isotype: K).

##### Distribution.

S. DRC to N. Zambia.

#### Coleus pulchellus

Taxon classificationPlantaeLamialesLamiaceae

(P.I.Forst.) P.I.Forst. comb. nov.

632E1D9275CC5B9596B11668367122DB

urn:lsid:ipni.org:names:77201175-1

Plectranthus pulchellus P.I.Forst., Austrobaileya 4: 182. 1994. Type: Australia, Queensland, Cook District, Wasp Gully, Glennie Tableland, 5 Jul. 1991, M.C.Tucker (holotype: BRI (AQ509442); isotype: CNS).

##### Distribution.

Australia: Queensland.

#### Coleus recurvata

Taxon classificationPlantaeLamialesLamiaceae

(Ryding) A.J.Paton comb. nov.

404D2ED423055256B0AC15A252078488

urn:lsid:ipni.org:names:77201176-1

Pycnostachys recurvata Ryding, Novon 9: 101. 1999. Type: Ethiopia, 60km W of Ambo along Lekemti (Nekemte) road, W.J.de Wilde & de Wilde-Duyfjes 8754 (holotype: C; isotypes: BR, ETH, K, UPS, WAG).

##### Distribution.

Ethiopia.

#### Coleus repens

Taxon classificationPlantaeLamialesLamiaceae

Gürke, Bot. Jahrb. Syst. 19: 213. 1894

EEF94661CEA75915B03B9DFBEA736214

Solenostemon repens (Gürke) J.K.Morton, J. Linn. Soc., Bot. 58: 272. 1962.
Plectranthus epilithicus B.J.Pollard, Kew Bull. 60: 145. 2005. Type: Kamerun, im Urwald westlich von Buea, Preuss 949 (holotype: B, destroyed); (neotype: Cameroon, Mt Kupe, Nyasoso, nature trail above the Government High School, Pollard 83 (neotype: K; isoneotypes KUPE, WAG YA, designated by Pollard (2005)).
Coleus carnosus A.Chev., J. Bot. (Morot) 22: 125. 1909., nom. illeg.
Coleus carnosus var. lamiifolius A.Chev., Explor. Bot. Afrique Occ. Franç. 1: 518. 1920. nom. illeg.

##### Distribution.

W. & WC. Trop. Africa.

#### Coleus reticulatus

Taxon classificationPlantaeLamialesLamiaceae

A.Chev., Mém. Soc. Bot. France 8: 200. 1912

B0BA64E970E25BAB90819DBD93CB90C3

Isodictyophorus reticulatus (A.Chev.) J.K.Morton, J. Linn. Soc., Bot. 58: 272. 1962.
Plectranthus reticulatus (A.Chev.) B.J.Pollard, Kew Bull. 67: 49. 2012. Type: Guinée Francaise, Cercle de Faranah (Faranna), dans la brousse très marécageuse et au bord des petits marigots, entre Kaba et Tonfili, 11 Jan.1909, Chevalier 20393 (holotype: P; isotype: K).
Isodictyophorus chevalieri Briq., Mém. Soc. Bot. France 8: 285. 1917. Type: Mali, Sedwhi toTimbuktu, 11–12 Feb. 1900, Chevalier s.n. (holotype: P).
Coleus casamancicus A.Chev. ex Hutch. & Dalziel, Fl. W. Trop. Afr. 2: 291. 1931. Type: Senegal, Casamance from Farancounda to Tambanaba, Sedhiou, 12 Feb. 1900, Chevalier 2777b (holotype: P).

##### Distribution.

W. Trop. Africa.

#### Coleus rhodesianum

Taxon classificationPlantaeLamialesLamiaceae

(N.E.Br.) A.J.Paton comb. nov.

DD75E338E5755E8E9F519E7E158C1D8F

urn:lsid:ipni.org:names:77201177-1

Englerastrum rhodesianum N.E.Br., Bull. Misc. Inform. Kew 1922: 31. 1922. Types: Zambia, Mumbwa, Macauly 637 (syntype: K); Livingstone, Rogers 7205 (syntype: not seen).
Englerastrum schweinfurthii Briq., Bot. Jahrb. Syst. 19: 178. 1894, non Coleus schweinfurthii Vatke.
Coleus englerastrum Roberty, Bull. Inst. Fondam. Afrique Noire, Sér. A, Sci. Nat. 16: 330. 1954. Type: Sudan, headwaters of Ghasal, Addai, Schweinfurth 2532 (isotypes: K, WU).
Englerastrum djalonense A.Chev., J. Bot. (Morot) 22: 126. 1909, non Coleus djalonensis A.Chev.
Plectranthus djalonensis (A.Chev.) A.J.Paton, Fl. Trop. E. Afr., Lamiac.: 286. 2009. Type: Guinea, between Timbo and Kouria, Sept. 1907, Chevalier s.n. (holotype: P; isotype: K).
Plectranthus piliferus Chiov., Boll. Soc. Bot. Ital. 1924: 44. 1924. Type: Angola, Mossamedes, Huambo, Mazzocchi-Alemanni (holotype: FT).
Englerastrum hutchinsonianum Alston, Bull. Misc. Inform. Kew 1926: 298. 1926. Type: Nigeria, Lokoja, Mt Patti, Dalziel 105 (holotype: K).
Englerastrum schlechteri Alston, Bull. Misc. Inform. Kew 1926: 297. 1926., nom. illeg., non. T.C.E.Fr.

##### Distribution.

Trop. Africa to Caprivi Strip.

#### Coleus robustus

Taxon classificationPlantaeLamialesLamiaceae

(Hook.f.) A.J.Paton comb. nov.

C438E6E172C75142B98288AF2C4A4059

urn:lsid:ipni.org:names:77201178-1

Anisochilus robustus Hook.f., Fl. Brit. India 4: 629. 1885. Type: India, Deccan Peninsula, Courtallum, Herb. Wight 625 (holotype: K (K000674766); isotype: E).
Anisochilus henryi K.Ravik. & V.Lakshm., Rheedea 9: 72. 1999. Type: India, Tamil Nadu, Theni Distr., near Varaiyaatumottai peak, Venniar Estate, Pachakumatchi hills, 1800 m, 28 May 1989, Lakshmanan & Ravikumar 91078 (holotype: CAL; isotypes: K, MH).

##### Distribution.

S. India.

#### Coleus rotundifolius

Taxon classificationPlantaeLamialesLamiaceae

(Poir.) A.Chev. & Perrot, Veg. Ut. Afr. Trop. Franç. 1: 101. 1905

C1DDEE0493485C4EA4BE13BF7D7F7480

Germanea rotundifolia Poir. in J.B.A.M.de Lamarck, Encycl. 2: 763. 1788.
Plectranthus rotundifolius (Poir.) Spreng., Syst. Veg. 2: 690. 1825.
Solenostemon rotundifolius (Poir.) J.K.Morton, J. Linn. Soc., Bot. 58: 272. 1962. Type: Mauritius (Ile de France), Commerson s.n. (holotype: P; isotype: FI).
Nepeta madagascariensis Lam., Encycl. 1: 712. 1785, non Coleus madagascariensis (Benth.) A.Chev. Type: illustration in Rheede, Hortus Mal. 11: 49 t.25. 1692.(lectotype: illustration designated here).
Plectranthus ternatus Sims, Bot. Mag. 51: t. 2460. 1824.
Coleus ternatus (Sims) A.Chev., Veg. Ut. Afr. Trop. Franç. 1: 109. 1905. Type: specimen of cultivated plant illustrated in Bot. Mag. t. 2460 (lectotype: illustration designated here).
Plectranthus tuberosus Blume, Bijdr. Fl. Ned. Ind.: 838. 1826.
Coleus tuberosus (Blume) Benth., Labiat. Gen. Spec.: 59. 1832.
Majana tuberosa (Blume) Kuntze, Revis. Gen. Pl. 2: 524. 1891. Type: Indonesia, Java, Blume s.n. (holotype: L: isotypes: NY, P).
Coleus rugosus Benth. in N.Wallich, Pl. Asiat. Rar. 2: 15. 1830. Type: India, Madras, Palamcotta, Herb. Madras, with ‘Dracocephalum rugosum’ mss., Wall. Cat. 2760 (holotype: K (no barcode.); isotype: K-W(K001117037).
Coleus parviflorus Benth. in A.P.de Candolle, Prodr. 12: 72. 1848.
Calchas parviflorus (Benth.) P.V.Heath, Calyx 6: 51. 1999. Type: South India, Quilon and Panpanassum, Wight 2512 (holotype: K(K000674660); isotype: E).
Coleus dysentericus Baker, Bull. Misc. Inform. Kew 1894: 10. 1894. Type: Niger, Zomba region, Barter 846 (holotype: K).
Coleus salagensis Gürke, Bot. Jahrb. Syst. 19: 220. 1894. Type: Benin, between Paratau and station Bismarkburg, Kling 199, Buttner 94, 690 (syntypes: not seen).
Plectranthus coppinii Heckel, Rev. Cultures Colon. 8: 164. 1900. Type and protologue not seen.
Coleus rehmannii Briq., Bull. Herb. Boissier, sér. 2, 3: 1075. 1903. Type: South Africa, Transvaal, Houtbosch, Rehmann 6156 (holotype: Z).
Coleus pallidiflorus A.Chev., J. Bot. (Morot) 22: 124. 1909. Type: Guinea, Conakry, Fouta Djalon, Dalaba, Nov. 1907, Chevalier s.n. (holotype: P). Locality of Chevalier 18234 in P and annotated as type, does not match protologue locality.
Coleus rotundifolius var. nigra A.Chev., Explor. Bot. Afrique Occ. Franç. 1: 520. 1920. Type: Ivory Coast, Haute sassandra, Pays Dyolas, village de Man, 13 May 1909, Chevalier 21532 (holotype: P).

##### Distribution.

Tropical Asia, cultivated elsewhere.

#### Coleus ruandensis

Taxon classificationPlantaeLamialesLamiaceae

(De Wild.) A.J.Paton comb. nov.

42FCB74BBB115E3EB75DE01A807419AD

urn:lsid:ipni.org:names:77201179-1

Pycnostachys ruandensis De Wild., Pl. Bequaert. 4: 401. 1928. Type: Rwanda, between Kirinda and Lubengera, Robyns 2449 (holotype: BR).
Pycnostachys clinodon Mildbr., Notizbl. Bot. Gart. Berlin-Dahlem 11: 405. 1932. Type: Tanzania, Njombe District: Lupembe, Schlieben 1053 (holotype: B, destroyed; isotypes: BM, BR, K, fragment).

##### Distribution.

Uganda, Burundi, Rwanda, DRC and Malawi.

#### Coleus rutenbergianus

Taxon classificationPlantaeLamialesLamiaceae

(Vatke) A.J.Paton & Phillipson comb. nov.

7378B3F125235356A80C65B7C5BC3F2C

urn:lsid:ipni.org:names:77201180-1

Plectranthus rutenbergianus Vatke, Abh. Naturwiss. Vereins Bremen 9: 135. 1885.
Solenostemon rutenbergianus (Vatke) Guillaumet & A.Cornet, Adansonia, n.s., 15: 524. 1976. Type: Madagascar, Nahe des Itasi Sees, 19 Dec. 1877, Rütenberg s.n. (holotype: B, destroyed).

##### Distribution.

Madagascar.

#### Coleus sahyadricus

Taxon classificationPlantaeLamialesLamiaceae

(Smitha & Sunojk.) Smitha comb. nov.

76495AE96C2D5B39BC66558171CF2DBB

urn:lsid:ipni.org:names:77201181-1

Plectranthus sahyadricus Smitha & Sunojk., Phytotaxa 345 (2): 166. 2018. Type: India. Kerala: Idukki district, Munnar, Lockhart Gap view point, 21 Sept. 2015, Smitha & Sunojkumar 135456 (holotype: CALI).

##### Distribution.

S. India.

#### Coleus sallyae

Taxon classificationPlantaeLamialesLamiaceae

(A.J.Paton) A.J.Paton comb. nov.

E645952803BB5756B89D3A793B25D78F

urn:lsid:ipni.org:names:77201182-1

Plectranthus sallyae A.J.Paton, Fl. Trop. E. Afr., Lamiac.: 275. 2009. Type: Tanzania, Ufipa District: Nkansi, 15 km on Kipili–Namanyere road, Bidgood, Mbago & Vollesen 2661 (holotype: K(K000431951); isotypes: K (K000431952), BR, C, DSM, EA, NHT, UPS).

##### Distribution.

SW. Tanzania.

#### Coleus sanguineus

Taxon classificationPlantaeLamialesLamiaceae

(Britten) A.J.Paton comb. nov.

ECB32B811A395F9383FC7415F3C13A6B

urn:lsid:ipni.org:names:77201183-1

Plectranthus sanguineus Britten, Trans. Linn. Soc. London, Bot. 4: 36. 1894. Type: Malawi, Mulanje, Dec.1891, Whyte 46 (holotype: BM; isotypes: G, K).

##### Distribution.

S. Trop. Africa.

#### Coleus saxorum

Taxon classificationPlantaeLamialesLamiaceae

(J.Mathew, Yohannan & B.J.Conn) Smitha comb. nov.

10B63389BA06539E8D96D911DA3445A2

urn:lsid:ipni.org:names:77201184-1

Plectranthus saxorum J.Mathew, Yohannan & B.J.Conn, Telopea 20: 187. 2017. Type: India, Kerala, Kozhikode District, Vellarimala, 10 km away from Muthappanpuzha, 30 Jan. 2015, J.Mathew 4911 (holotype: TBGT; isotype, MSSRF).

##### Distribution.

India, endemic to Western Ghats.

#### Coleus scaber

Taxon classificationPlantaeLamialesLamiaceae

(Benth.) A.J.Paton comb. nov.

EE8FFEB91EFB5C6E9F45A767E06D2970

urn:lsid:ipni.org:names:77201185-1

Anisochilus scaber Benth. in A.P.de Candolle, Prodr. 12: 81. 1848. Type: India, Deccan Peninsula, Courtallum, Herb. Wight 2520 (holotype: K (K000674680); isotypes E, K (K000674681).

##### Distribution.

S. India.

#### Coleus scandens

Taxon classificationPlantaeLamialesLamiaceae

Gürke, Abh. Königl. Akad. Wiss. Berlin 1894: 51. 1894

5C800E84FBF55277B4B36E2186AE72A9

Englerastrum scandens (Gürke) Alston, Bull. Misc. Inform. Kew 1926: 299. 1926.
Plectranthus scandens (Gürke) R.H.Willemse, Blumea 25: 510. 1979. Types: Tanzania, Lushoto District: Msinga to Kwa Mshusa, Holst 9119 (syntype: B, destroyed; isosyntype: K) & 9120a (syntype: B, destroyed; isosyntypes: G, JE, K, W).

##### Distribution.

Kenya to N. Tanzania.

#### Coleus scebeli

Taxon classificationPlantaeLamialesLamiaceae

Chiov., Fl. Somala 1: 2. 1929

A5A2C1D2498B5431B4160756513B7188

Plectranthus scebeli (Chiov.) Ryding, Kew Bull. 54: 125. 1999. Type: Somalia, Shabeellaha Dhexe Rguion, between Ganale and Audegle, Puccioni & Stafanini 48 (holotype: FT).

##### Distribution.

S. Somalia.

#### Coleus schizophyllus

Taxon classificationPlantaeLamialesLamiaceae

(Baker) A.J.Paton comb. nov.

96CB99B4F30A5B7086A96E4936FBFF88

urn:lsid:ipni.org:names:77201186-1

Plectranthus schizophyllus Baker in D.Oliver & auct. suc. (eds.), Fl. Trop. Afr. 5: 414. 1900.
Calchas schizophyllus (Baker) P.V.Heath, Calyx 6: 51. 1999. Type: Malawi, between Khondowe and Karonga, Whyte 376 (holotype: K).

##### Distribution.

SW. Tanzania to N. Malawi.

#### Coleus schliebenii

Taxon classificationPlantaeLamialesLamiaceae

(Mildr.) A.J.Paton comb. nov.

C3D39DAEB79B595F88AC6348BCD48278

urn:lsid:ipni.org:names:77201187-1

Pycnostachys schliebenii Mildbr., Notizbl. Bot. Gart. Berlin-Dahlem 11: 405. 1932. Type: Tanzania, Njombe District: Lupembe, Schlieben 713 (holotype: B, destroyed: isotypes: BR, Z; K, fragment).

##### Distribution.

Tanzania to Zambia.

#### Coleus scruposus

Taxon classificationPlantaeLamialesLamiaceae

A.J.Paton nom. nov.

B1F52070805B56418AC1CB9700751EDF

urn:lsid:ipni.org:names:77201188-1

Pycnostachys kassneri De Wild., Contr. Fl. Katanga: 172 1921., non Coleus kassneri Robyns & Lebrun. Type: DRC, Katanga, West Kundelungu, Kassner 2794 (holotype: BR; isotypes: BM, K).
Pycnostachys carigensis Gürke, Ann. Mus. Congo Belge, Bot., sér. 4, 2: 135. 1913., nom. nud.

##### Distribution.

Tanzania to Zambia.

#### Coleus scutellarioides

Taxon classificationPlantaeLamialesLamiaceae

(L.) Benth. in N.Wallich, Pl. Asiat. Rar. 2: 16. 1830

14B2B4EE11B954E0B716C471EFCD2253

Ocimum scutellarioides L., Sp. Pl. ed. 2: 833. 1763.
Plectranthus scutellarioides (L.) R.Br., Prodr. Fl. Nov. Holl.: 506. 1810.
Plectranthus aromaticus Roxb., Hort. Bengal.: 45. 1814.
Majana scutellariodes (L.) Kuntze, Revis. Gen. Pl. 2: 524. 1891.
Solenostemon scutellarioides (L.) Codd, Bothalia 11: 439. 1975.
Calchas scutellarioides (L.) P.V.Heath, Calyx 6: 51. 1999. Type: Majana (alba et rubra) Rumphius, Herb. Amboin. 5: 291, t. 101. 1747, (lectotype: illustration designated by Merrill (Interpr. Rumph. Herb. Amboin.: 460 1917).
Polypodium ovatum Burm. f., Fl. Ind.: 233. 1768. Type: Java, Unknown collector (holotype: G).
Germanea nudiflora Poir. in J.B.A.M.de Lamarck, Encycl. 2: 763. 1788.
Plectranthus nudiflorus (Poir.) Willd., Sp. Pl. 3: 168. 1800. Type: Amboina, Willdenow Herbarium 11079.1 & 11079.2 (syntypes: B-W).
Ocimum peltatum Schweigg. ex Schrank, Denkschr. Königl.-Baier. Bot. Ges. Regensburg 2: 55. 1822. Type: Cultivated from seed collected by Martius in Brazil (type: not seen).
Plectranthus ingratus Blume, Bijdr. Fl. Ned. Ind.: 836. 1826.
Coleus ingratus (Blume) Benth., Labiat. Gen. Spec.: 53. 1832.
Coleus scutellarioides var. ingratus (Blume) Miq., Fl. Ned. Ind. 2: 950. 1858. Type: Indonesia, Java, G. Parang, Blume 992 (lectotype: L, designated by Keng (1969)).
Plectranthus laciniatus Blume, Bijdr. Fl. Ned. Ind.: 838. 1826.
Coleus laciniatus (Blume) Benth., Labiat. Gen. Spec.: 56. 1832.
Coleus scutellarioides var. laciniatus (Blume) Miq., Fl. Ned. Ind. 2: 950. 1858. Type: Indonesia, Java, Blume s.n. (lectotype: L, designated by Keng (1969)).
Plectranthus scutellarioides Blume, Bijdr. Fl. Ned. Ind.: 837. 1826., nom. illeg.
Coleus blumei Benth., Labiat. Gen. Spec.: 56. 1832.
Coleus scutellarioides var. blumei (Benth.) Miq., Fl. Ned. Ind. 2: 950. 1858.
Majana scutellarioides var. blumei (Benth.) Kuntze, Revis. Gen. Pl. 2: 524. 1891.
Solenostemon blumei (Benth.) M.Gómez, Bol. Secr. Agric. Comerc. Trab., Cuba 22: 127. 1914.
Plectranthus blumei (Benth.) Launert, Mitt. Bot. Staatssamml. München 7: 301. 1968. Type: Indonesia, Java, Blume s.n. (lectotype: L, designated by Suddee et al. (2004)).
Coleus atropurpureus Benth. in N.Wallich, Pl. Asiat. Rar. 2: 16. 1830.
Majana scutellarioides var. atropurpureus (Benth.) Kuntze, Revis. Gen. Pl. 2: 524. 1891.
Calchas atropurpureus (Benth.) P.V.Heath, Calyx 5: 160. 1997. Type: Singapore, Wall. Cat. 2733A (syntypes: K (no barcode), K-W (K001116976)).
Coleus atropurpureus var. ramosus Benth. in N.Wallich, Pl. Asiat. Rar. 2: 16. 1830. Type: Malaysia, Penang, Wall. Cat. 2733B (syntypes: G-DC(microfiche), K-W (K001116976).
Coleus acuminatus Benth., Linnaea 6: 81. 1831.
Majana acuminata (Benth.) Kuntze, Revis. Gen. Pl. 2: 524. 1891.
Calchas acuminatus (Benth.) P.V.Heath, Calyx 5: 160. 1996. Type: Philippines, Island of Samar, 1841, Cuming 1683 (neotype: K (K000897809), neotype designated by Suddee et al. 2004; isoneotypes: BM, K (K000897810), P, TCD).
Coleus atropurpureus var. densiflorus Benth., Labiat. Gen. Spec.: 54. 1832. Type: Philippines, Manila, 1830, Chamisso s.n. (syntype: K (K000897808).
Coleus grandifolius Benth., Labiat. Gen. Spec.: 53. 1832.
Majana grandifolia (Benth.) Kuntze, Revis. Gen. Pl. 2: 524. 1891.
Coleus scutellarioides var. grandifolius (Benth.) Keng, Gard. Bull. Singapore 24: 58. 1969. Type: Timor, Guichenot s.n. (syntype P; isosyntypes: K, L, G-DC).
Coleus multiflorus Benth., Labiat. Gen. Spec.: 55. 1832.
Majana multiflora (Benth.) Kuntze, Revis. Gen. Pl. 2: 524. 1891. Type: Philippines, Manila, Perrottet s.n. (holotype: P).
Coleus secundiflorus Benth., Labiat. Gen. Spec.: 55. 1832.
Majana secundiflora (Benth.) Kuntze, Revis. Gen. Pl. 2: 524. 1891. Type: Timor, Guichenot s.n. (holotype: P).
Coleus grandifolius Blanco, Fl. Filip.: 482. 1837., nom. illeg.
Coleus blancoi Benth. in A.P.de Candolle, Prodr. 12: 79. 1848.
Majana blancoi (Benth.) Kuntze, Revis. Gen. Pl. 2: 524. 1891. Type: Phillippines, Blanco, s.n. (type: not seen).
Coleus pumilus Blanco, Fl. Filip.: 482. 1837.
Majana pumila (Blanco) Kuntze, Revis. Gen. Pl. 2: 524. 1891. Type: Philippines, Barrio of Pineda, Pasig, Rizal Province, Luzon, Oct. 1914, Merrill: Species Blancoanae No. 190 (neotype: K, designated by Suddee et al. (2004)).
Coleus atropurpureus var. javanicus Benth. in A.P.de Candolle, Prodr. 12: 74. 1848. Type: Indonesia, Java, Zollinger 138 (syntypes: G-DC, K).
Coleus blumei var. pectinatus C.Morren, Belgique Hort. 6: 99. t 18. 1856. Type: Cultivated in Liege, illustration cited above (lectotype: illustration designated here).
Coleus scutellarioides var. celebicus Miq., Fl. Ned. Ind. 2: 950. 1858. Type: Indonesia, Celebes, Reinwardt s.n. (type: not seen).
Coleus scutellarioides var. gracilis Miq., Fl. Ned. Ind. 2: 950. 1858. Type: Indonesia, Ceram, Bangka, Miquel (type: not seen).
Perilla nankinensis Wender., Linnaea 29: 726. 1859, nom. illeg., non P. nankinensis (Lour.) Decne.
Coleus verschaffeltii Lem., Ill. Hort. 8: t. 293. 1861. Type: based on cultivated plants from Java,illustration cited above (lectotype: illustration, designated by Suddee et al. 2004).
Coleus marmoratus W.Bull, Proc. Roy. Hort. Soc. London 4: 133. 1864.
Coleus verschaffeltii var. marmoratus (W.Bull) André, Pl. Feuill. Ornem.: 144. 1866. Type: not seen.
Coleus gibsonii Verl., Veitch & Sons in Gard. Chron. 1866: 432 (12 May 1866). Rev. Hort. (Paris) 37: 279. 1866 (July 1866). Type: Cultivated in London, sent by John Veitch from New Caledonia. (type: not seen).
Coleus veitchii Dombrain, Fl. Mag. (London) 6: t. 345. 1867. Type: Cultivated in London, sent by John Veitch from New Caledonia, illustration cited above (lectotype: illustration, designated here).
Coleus batemannii T.Moore, Gard. Chron. 1868: 377. 1868. Type: not seen.
Coleus bausei T.Moore, Gard. Chron. 1868: 377. 1868. Type: not seen.
Coleus berkeleyi T.Moore, Gard. Chron. 1868: 376. 1868. Type: not seen.
Coleus clarkii T.Moore, Gard. Chron. 1868: 377. 1868. Type: not seen.
Coleus dixii T.Moore, Gard. Chron. 1868: 376. 1868. Type: not seen.
Coleus marshallii T.Moore, Gard. Chron. 1868: 376. 1868.
Coleus blumei var. marshallii (T.Moore) Rothsch., Pl. Feuill. Col., 2: 74. t. 37. 1869. Type: Illustration cited above (lectotype: illustration, designated here).
Coleus murrayi T.Moore, Gard. Chron. 1868: 377. 1868.
Coleus blumei var. murrayi (T.Moore) Rothsch., Pl. Feuill. Col., 2: 74 t. 37. 1869. Type: Illustration cited above (lectotype: illustration, designated here).
Coleus reveesii T.Moore, Gard. Chron. 1868: 377. 1868. Type: not seen.
Coleus ruckeri T.Moore, Gard. Chron. 1868: 376. 1868. Type: not seen.
Coleus saundersii T.Moore, Gard. Chron. 1868: 376. 1868. Type: not seen.
Coleus scottii T.Moore, Gard. Chron. 1868: 377. 1868. Type: not seen.
Coleus wilsonii T.Moore, Gard. Chron. 1868: 377. 1868. Type: not seen.
Coleus telfordii McPhail ex H.Laurentius, Nursery Cat. (H.Laurentius) 40: 3. 1868.
Coleus blumei var. telfordii (McPhail ex H.Laurentius) Rothsch., Pl. Feuill. Col., 2: 74 t. 37. 1869. Type: Illustration cited above (lectotype: illustration, designated here).
Coleus scutellarioides var. angustifolia Benth., Fl. Austral. 5: 80. 1870.
Calchas scutellarioides var. angustifolia (Benth.) P.V.Heath, Calyx 6: 51. 1999. Type: Australia, Table Hill, Victoria River & Macadam Range, 1855, F. Mueller s.n. (syntype: K); Port Essington, Armstrong 244 (syntype: K).
Coleus scutellarioides var. laxa Benth., Fl. Austral. 5: 80. 1870. Types: Australia, Roe River, York Sound, NW coast, A.Cunningham (syntype: K); Roebuck Bay NW coast, Martin s.n. (syntype: not seen); Arnhems land, Mackinley, R.Brown (syntype: MEL).
Coleus scutellarioides var. limnophila Benth., Fl. Austral. 5: 80. 1870.
Calchas scutellarioides var. limnophila (Benth.) P.V.Heath, Calyx 6: 51. 1999. Type: Australia, Nicholson & MacArthur Rivers, F. Mueller (syntypes: K).
Coleus tryonii auct., Fl. Mag. (London), n.s., 1: t. 34. 1872. Type: cultivated in UK, Leicester, garden of Captain Tryon (lectotype: illustration designated here).
Coleus verschaffeltii var. splendens auct., Garden (London 1871–1927) 5: 54. 1874. Type: not seen.
Coleus multicolor J.H.Veitch ex Kellock, Gard. Chron., n.s., 7: 571. 1877. Type: not seen.
Coleus hendersonii Regel, Gartenflora 28: 244. 1879. Type: not seen.
Coleus × eureka auct., Gard. Chron., ser. 3, 5: 808. 1889. nom. nud.
Coleus gaudichaudii Briq., Annuaire Conserv. Jard. Bot. Genève 2: 237. 1898. Type: Philippines, fle de Luzon pres de Manille, Dec. 1836, Gaudichaud s.n. (holotype: G-DEL).
Coleus igolotorum Briq., Annuaire Conserv. Jard. Bot. Genève 2: 236. 1898. Type: Philippines, fle de Luzon, dans le pays des Igolotes, Callery 48 (holotype: G-DEL; isotype: P).
Coleus savannicola K.Schum. in K.M.Schumann & C.A.G.Lauterbach, Fl. Schutzgeb. Südsee: 529. 1900. Type: not seen.
Coleus formosanus Hayata, J. Coll. Sci. Imp. Univ. Tokyo 22: 320. 1906. Type: Kotosho, 21 Nov. 1899, Miyake s.n. (holotype: TI).
Coleus macranthus var. crispipilus Merr., Philipp. J. Sci. 1(suppl.): 235. 1906.
Coleus crispipilus (Merr.) Merr., Philipp. J. Sci., C 5: 382. 1910.
Calchas crispipilus (Merr.) P.V.Heath, Calyx 5: 160. 1996.
Coleus scutellarioides var. crispipilus (Merr.) H.Keng, Gard. Bull. Singapore 24: 56. 1969.
Calchas scutellarioides var. crispipilus (Merr.) P.V.Heath, Calyx 6: 51. 1999. Type: Philippines, Suyoc to Panai, Province of Benguet, Luzon, 2200 m, Nov. 1905, Merrill 4780 (holotype: PNH; isotypes: BM, K, NY).
Coleus pubescens Merr., Philipp. J. Sci., C 3: 432. 1909. Type: Philippines, Babuyan Island, 17 June 1907, Eugenio Zenix, Bureau of Science 3892 (holotype: PNH; isotype: BM).
Coleus zschokkei Merr., Philipp. J. Sci., C 5: 382. 1910. Type: Philippines, Mt Pulog, Prov. of Bengnet, Luzon, May 1909, Merrill 6529 (holotype: PNH; isotypes: K, NY, P).
Coleus integrifolius Elmer, Leafl. Philipp. Bot. 7: 2696. 1915.
Coleus scutellarioides var. integrifolius (Elmer) Keng, Gard. Bull. Singapore 24: 58. 1969. Type: Philippines, Mindanao, Cabadbaran (Mt Urdaneta), Prov. of Agusan, Island of Mindanao, August 1912, Elmer 13627 (holotype: PNH; isotypes: A, BM, K, L, NY, P).
Coleus gibbsiae S.Moore in L.S.Gibbs, Fl. Arfak Mts.: 178. 1917.
Coleus scutellarioides var. gibbsiae (S.Moore) Keng, Gard. Bull. Singapore 24: 58. 1969. Type: Indonesia, New Guinea, Arfak Mts, 2100 m, Gibbs 5909 (holotype: BM; isotypes: K, L).
Coleus rehneltianus A.Berger, Bot. Jahrb. Syst. 54 (5, Beibl. 120): 197. 1917. Type: not seen.
Coleus hybridus Cobeau, Arch. Bot. (Forlì) 4: 35. 1928. Type: not seen.

##### Distribution.

Trop. & Subtrop. Asia to N. Australia. Widely cultivated as an ornamental.

#### Coleus seretii

Taxon classificationPlantaeLamialesLamiaceae

De Wild., Bol. Soc. Ibér. Ci. Nat. 19: 122. 1920

4D40E3C7F0795D6FADD56FB1E0F10A4F

Calchas seretii (De Wild.) P.V.Heath, Calyx 6: 51. 1999.
Plectranthus seretii (De Wild.) Vollesen, Opera Bot. 59: 84. 1980. Type: DRC, Buta to Bima (Bali), Seret 68 (syntype: BR) & Suronga Forest, Seret 420 (wrongly cited as 120 in protologue) (syntype: BR; isosyntype: K).

##### Distribution.

Ethiopia, DRC, Tanzania, NW. Madagascar.

#### Coleus sessilifolius

Taxon classificationPlantaeLamialesLamiaceae

(A.J.Paton) A.J.Paton comb. nov.

6BC35D9710C6580EB7A5A98CE86D6A59

urn:lsid:ipni.org:names:77201190-1

Plectranthus sessilifolius A.J. Paton, Fl. Zambes. 8(8): 281. 2013. Type: Zimbabwe, Mutare Dist., Himalaya Mts, 5 Mar.1954, Wild 4505 (holotype: K; isotypes: LISC, MO, SRGH).

##### Distribution.

Zimbabwe, Mozambique.

#### Coleus shirensis

Taxon classificationPlantaeLamialesLamiaceae

Gürke, Bot. Jahrb. Syst. 19: 216. 1894

90C0FCF670C058DCB40ADA22EAD39426

Solenostemon shirensis (Gürke) Codd, Bothalia 11: 440. 1975.
Calchas shirensis (Gürke) P.V.Heath, Calyx 6: 51. 1999.
Plectranthus shirensis (Gürke) A.J.Paton, SABONET Rep. Ser. 31: 189. 2005. Type: Malawi, 1891, Buchanan 376 (lectotype: K; isolectotype: E, designated by Mathew (1976)).
Coleus punctatus Baker, Bull. Misc. Inform. Kew 1895: 291. 1895. Type: Zambia, Mweru Plateau, W of Lake Tanganyika, 1894, Carson 25 (holotype: K).
Solenostemon zambesiacus Baker in D.Oliver & auct. suc. (eds.), Fl. Trop. Afr. 5: 421. 1900. Types: Malawi, Blantyre, 1887, Last s.n. (syntype: K); ?Mozambique, “Zambesia”, between Shibisa and Tshinmuzo, Nov.1859, Kirk s.n. (syntype: K).

##### Distribution.

SW. & S. Tanzania to S. Trop. Africa.

#### Coleus shoolamudianus

Taxon classificationPlantaeLamialesLamiaceae

(Sunil & Naveen Kum.) Smitha & A.J. Paton comb. nov.

0A55301B80C05BCAB6DADF8807C5D15A

urn:lsid:ipni.org:names:77201191-1

Anisochilus shoolamudianus Sunil & Naveen Kum. Webbia 70(2): 217. 2015. Type: India, Ernakulam District, Edamalayar Forest Range, Shoolamudi, Sunil & Naveen Kumar 6918 (holotype: MH; isotype CALI).

#### Coleus sigmoideus

Taxon classificationPlantaeLamialesLamiaceae

(A.J.Paton) A.J.Paton comb. nov.

377EB9F4B94D51668924FBD7F2BBB624

urn:lsid:ipni.org:names:77201192-1

Plectranthus sigmoideus A.J.Paton, Fl. Trop. E. Afr., Lamiac.: 333. 2009. Type: Zambia, track opposite turning to Mbala (Abercorn) Club, Richards 4353 (holotype: K).

##### Distribution.

SW. Tanzania to Zambia.

#### Coleus socotranus

Taxon classificationPlantaeLamialesLamiaceae

(A.R.Sm.) A.J.Paton comb. nov.

5BA72BB59DCC5EF09274DF75DF63504D

urn:lsid:ipni.org:names:77201193-1

Plectranthus socotranus A.R.Sm., Hooker’s Icon. Pl. 37: t. 3692. 1971. Type: Socotra, Adhoh de Melhoh, Hagghiher Mts, 22 Apr. 1967, Smith & Lavranos 499 (holotype: K; isotype FI).

##### Distribution.

Socotra.

#### Coleus sparsiflorus

Taxon classificationPlantaeLamialesLamiaceae

Elmer, Leafl. Philipp. Bot. 7: 2699 1915

B057F35FE03C5712A90E177938C3C5F6

Plectranthus sparsiflorus (Elmer) H.Keng, Fl. Males. 8: 391. 1978. Type: Philippines, Mindanao, Mt. Apo, Sept. 1912, A.D.E.Elmer 11646 (isotypes: G, L).

##### Distribution.

Philippines.

#### Coleus magnificus

Taxon classificationPlantaeLamialesLamiaceae

P.I.Forst. & A.J.Paton nom. nov.

FFADA3A679B6554C963C125FF5CAF5CC

urn:lsid:ipni.org:names:77201303-1

Plectranthus spectabilis S.T.Blake, Contr. Queensland Herb. 9: 50. 1971, non Coleus spectabilis Miq. Type: Australia, Queensland, Macalister Range near Hartley Creek C NW of Cairns (Cultivated at The Gap), Apr. 1959, S.T.Blake 20535 (holotype: BRI).

##### Distribution.

Australia: Queensland.

#### Coleus sphaerocephalus

Taxon classificationPlantaeLamialesLamiaceae

(Baker) A.J.Paton comb. nov.

B72ACBC3885C5E04900E5655624EB3A4

urn:lsid:ipni.org:names:77201194-1

Pycnostachys sphaerocephala Baker, Bull. Misc. Inform. Kew 1898: 162. 1898. Type: Malawi, Nyika Plateau, 1800–2100 m, Whyte 139 (holotype: K).
Pycnostachys whytei Baker in D.Oliver & auct. suc. (eds.), Fl. Trop. Afr. 5: 383. 1900. Type: Malawi, Chitipa (Fort Hill), Whyte s.n. (holotype: K).
Pycnostachys perkinsii E.A.Bruce, Bull. Misc. Inform. Kew 1939: 587. 1940.
Pycnostachys kaessneri Perkins, Notizbl. Bot. Gart. Berlin-Dahlem 8: 73. 1921., nom. illeg. Type: DRC, Katanga, Muhila (Mugila) Mts, western slope, Kassner 2991a (holotype: B, destroyed; isotypes: BR, K).

##### Distribution.

Tanzania to Zambia.

#### Coleus splendens

Taxon classificationPlantaeLamialesLamiaceae

(P.I.Forst.) P.I.Forst. comb. nov.

0B0DD26941745327BBA08DA5B6747517

urn:lsid:ipni.org:names:77201302-1

Plectranthus splendens P.I.Forst., Austrobaileya 9(2): 211. 2014. Type: Australia, Queensland, Cook District, Hann Tableland National Park, 8 April 2013, P.I.Forster PIF39587 (holotype: BRI; isotypes: CNS, K, MEL).

##### Distribution.

Australia: Queensland.

#### Coleus splendidus

Taxon classificationPlantaeLamialesLamiaceae

A.Chev., J. Bot. (Morot) 22: 122. 1909

1186C275898D54939D4400C1D7AD04D4

Coleus splendidus A.Chev., J. Bot. (Morot) 22: 122. 1909. Type: Guinea, Timbo, 16 September 1907, A. Chevalier s.n. (holotype: P).
Coleus djalonensis A.Chev., J. Bot. (Morot) 22: 123. 1909. Type: Guinea, between Kouria and Bouroukountou, 12 Sept. 1907, A. Chevalier s.n. (holotype: P).
Coleus peulhorum var. violacea A.Chev., J. Bot. (Morot) 22: 124. 1909. Type: Guinea: Fouta Djallon, entre Diaguissa et Boulwel, Chevalier 18606bis (holotype: P; isotype: P).

##### Distribution.

Guinea.

#### Coleus stachyoides

Taxon classificationPlantaeLamialesLamiaceae

(Oliv.) E.A.Bruce, Bull. Misc. Inform. Kew 1934: 306. 1934

EB866CACEF0E50598A37449E22D0E3D3

Plectranthus stachyoides Oliv., Trans. Linn. Soc. London 29: 136. 1875. Type: Uganda, West Nile District: Madi, Grant 732 (holotype: K).
Plectranthus cylindrostachys Robyns & Lebrun, Rev. Zool. Bot. Africaines 16: 356. 1928. Type: Burundi, Irubura, Akanguru valley, Robyns 2403 (holotype: BR; isotype: K).

##### Distribution.

Central African Rep. to E. Trop. Africa.

#### Coleus steenisii

Taxon classificationPlantaeLamialesLamiaceae

(H.Keng) A.J.Paton comb. nov.

A71407183F835BEAACB03DC78D69B68C

urn:lsid:ipni.org:names:77201304-1

Plectranthus steenisii H.Keng, Gard. Bull. Singapore 24: 145. 1969. Type: Java, res. Pasoeroean, G. Ardjoeno, rd bet. Sawahan Bakal and Sawaham Ardjoeno, 5 June 1935, van Steenis 7111 (holotype: L).

##### Distribution.

E. Java.

#### Coleus stenostachys

Taxon classificationPlantaeLamialesLamiaceae

(Baker) A.J.Paton & Phillipson comb. nov.

B511EF53132E5233A12766A7A75DF1DF

urn:lsid:ipni.org:names:77201305-1

Pycnostachys stenostachys Baker in D.Oliver & auct. suc. (eds.), Fl. Trop. Afr. 5: 380. 1900. Type: Uganda, Bunyoro District: sides of Nile, 1862, Grant in Speke & Grant s.n. (holotype: K).
Pycnostachys coerulea Hook., Exot. Fl. 3: t. 202. 1826, non Coleus coeruleus Gürke. Type: cultivated from seeds sent from Madagascar, collected by Bojer & Helsinger (holotype: K; isotype: M).
Pycnostachys micrantha Gürke in H.G.A.Engler, Pflanzenw. Ost-Afrikas, C: 345. 1895, non Coleus micranthus Maxim. Types: Tanzania, Bukoba District: Karagwe, Stuhlmann 1720 & Mpororo, Stuhlmann 2056 & Bukoba, Stuhlmann 3276, 3718 & 3953 (syntypes: B, destroyed).
Pycnostachys brevipetiolata De Wild., Pl. Bequaert. 4: 394. 1928. Type: DRC, Kivu, Rutshuru R., Kaisafu, Bequaert 5972 (holotype: BR).

##### Distribution.

Ethiopia to South Africa, Madagascar.

#### Coleus strictipes

Taxon classificationPlantaeLamialesLamiaceae

(G.Taylor) A.J.Paton comb. nov.

A263CCA35F23507A972BC69242401565

urn:lsid:ipni.org:names:77201306-1

Plectranthus strictipes (G.Taylor) A.J.Paton, Fl. Zambes. 8(8): 234. 2013.
Holostylon strictipes G.Taylor in J. Bot. 69, suppl.2: 161. 1931. Types: Angola, Cahaingalamush near Mulanje, 1903, Gossweiler 1038 (syntype: BM, K, P); Gola Luige, Malanje, Nov. 1922, Gossweiler 8848 (syntype: BM, K).

##### Distribution.

Angola.

#### Coleus strobilifer

Taxon classificationPlantaeLamialesLamiaceae

(Roxb.) A.J.Paton comb. nov.

272D5F557CAA5B1E88D134E89AEFE1FD

urn:lsid:ipni.org:names:77201307-1

Fig. 5E

Plectranthus strobilifer Roxb., Hort. Bengal.: 45. 1814. Type: Katu-Kurka, Illustration in Rheede, Hort. Malab. 10: 179., t.90. 1690, (lectotype: illustration, designated by Suddee and Paton 2009).
Lavandula carnosa L.f., suppl. Pl.: 273. 1782 non Coleus carnosus Hassk.
Plectranthus carnosus (L.f.) Sm. in A.Rees, Cycl. 27: n.° 7. 1814.
Anisochilus carnosus (L.f.) ex Benth., Edwards’s Bot. Reg. 15: t. 1300. 1830. Type: India, Koenig s.n., Linnean Herbarium 727.7 (lectotype LINN).
Plectranthus dubius Spreng., Syst. Veg. 2: 691. 1825., nom. illeg.
Anisochilus carnosus var. purpurascens Benth. in N.Wallich, Pl. Asiat. Rar. 2: 18. 1830. Type: Burma, inm onte Taong Dong Ava, 24 Nov. 1826, Wall. Cat. 2753A (lectotype: K (K000674685); isolectotypes G-DC, K-W (K001117024); designated by Suddee & Paton (2009)).
Anisochilus glaber Schrad., Index Seminum (GOET) 1833: 1. 1833.
Anisochilus carnosus var. glaber (Schrad.) Benth., Labiat. Gen. Spec.: 711. 1835. Type: India, W Himalaya, to 2400 m, Edgworth 14 (neotype: K(K000674684), designated by Suddee et al. (2004)).
Anisochilus carnosus var. villosior Benth. in A.P.de Candolle, Prodr. 12: 81. 1848. Type: Deccan Peninsula. Herb. Wight 2516 (K (K000674688), lectotype designated Suddee and Paton 2009).
Anisochilus carnosus var. viridis Benth. in A.P.de Candolle, Prodr. 12: 81. 1848. Type: India, Deccan Peninsula, Herb. Wight 2521, (holotype: K (K000674686).
Anisochilus crassus Benth. in A.P.de Candolle, Prodr. 12: 81. 1848. Type: Deccan Peninsula, Herb. Wight. 2517 (lectotype: K (K000674682); isolectotype: E; designated Suddee and Paton 2009).
Anisochilus eriocephalus Benth. in A.P.de Candolle, Prodr. 12: 81. 1848.
Anisochilus carnosus var. eriocephalus (Benth.) T.Cooke, Fl. Bombay 2: 450. 1908. Type: Deccan Peninsula, Bellary, Herb. Wight 2518 (lectotype: K (K000674676); isolectotypes: E, K (K000674678, K000674677), designated Suddee & Paton (2009)).
Anisochilus decussatus Dalzell & A.Gibson, Bombay Fl.: 206. 1861. Type: India, Concan, on the Highest Ghauts opposite Bombay, Dalzell s.n. (holotype: K).
Anisochilus rupestris Wight ex Hook.f., Fl. Brit. India 4: 627. 1885. Type: India Deccan Peninsula, Herb. Wight 2521, (holotype: K (K000674686).

##### Distribution.

Indian Subcontinent to Indo-China.

#### Coleus stuhlmannii

Taxon classificationPlantaeLamialesLamiaceae

(Gürke) A.J.Paton comb. nov.

A0B533D857285625B122B7AD4AF5A32F

urn:lsid:ipni.org:names:77201308-1

Pycnostachys stuhlmannii Gürke in H.G.A.Engler, Pflanzenw. Ost-Afrikas, C: 345. 1895. Type: Tanzania, Bukoba District: Karagwe, Ngaramo, Stuhlmann 1630 (holotype: B, destroyed; K, fragment).
Pycnostachys remotifolia Baker, Bull. Misc. Inform. Kew 1898: 161. 1898. Type: Malawi, Chitipa (Fort Hill), July 1896, Whyte s.n. (holotype: K).
Pycnostachys bequaertii De Wild., Contr. Fl. Katanga: 171. 1921. Type: DRC, Katanga, Shinsenda, Bequaert 425 (lectotype: BR; designated by Bramley in Paton et al. (2009)).
Pycnostachys longifolia De Wild., Contr. Fl. Katanga: 172. 1921. Type: DRC, Katanga, Welgelegen, Bequaert 562 (holotype: BR; isotype: K, fragment).

##### Distribution.

Kenya to S. Trop. Africa.

#### Coleus suaveolens

Taxon classificationPlantaeLamialesLamiaceae

(S.T.Blake) P.I.Forst. & T.C.Wilson comb. nov.

76B5C28DF61F5377A90B6A51215CBD41

urn:lsid:ipni.org:names:77201309-1

Plectranthus suaveolens S.T.Blake, Contr. Queensland Herb. 9: 30. 1971. Type: Australia, Queensland, Cultivated at The Gap, Brisbane. Grown from transplants collected at Queen Mary’s Falls, by J.A. Gresty, Jan. 1959, S.T.Blake 20506 (holotype: BRI).

##### Distribution.

E. Australia.

#### Coleus subspicatus

Taxon classificationPlantaeLamialesLamiaceae

(Hochst.) Walp., Repert. Bot. Syst. 6: 658. 1847

072BC123B9CE57228BA8CDB3E0991DDC

Plectranthus subspicatus Hochst., Flora 28: 67. 1845. Type: South Africa, Cape, Uitenhage, Krauss 1112 (syntypes: TUB (TUB008736, TUB008735).
Plectranthus spicatus E.Mey., Comm. Pl. Afr. Austr.: 230. 1838.
Burnatastrum spicatum (E.Mey.) Briq. in H.G.A.Engler & K.A.E.Prantl, Nat. Pflanzenfam. 4(3a): 358. 1897, non Coleus spicatus Benth. Type: South Africa, Cape, Bathurst Division, Glenfilling, 1836, Drège 4731b (lectotype: K; isolectotypes: MO, P, S; designated by Codd (1975)).

##### Distribution.

S. Mozambique to South Africa.

#### Coleus succulentus

Taxon classificationPlantaeLamialesLamiaceae

Pax, Bot. Jahrb. Syst. 39: 646. 1907

6F423DC3EB2850C8B1B8943A026BB8EF

Coleus succulentus Pax, Bot. Jahrb. Syst. 39: 646. 1907. Types. Ethiopia, Shoa, Schankora, Rosen s.n. (holotype: WRSL, destroyed); neotype: Ethiopia, Zuquala Mt, Ryding & Puff 1657 (neotype: UPS; isoneotype: ETH ; designated by Ryding (2000)).
Plectranthus pseudomarrubioides R.H.Willemse, Kew Bull. 40: 93. 1985. Type: Ethiopia, Debre Libanos, de Wilde 8656 (holotype: WAG).

##### Distribution.

Ethiopia to N. Tanzania, Arabian Peninsula.

#### Coleus suffruticosus

Taxon classificationPlantaeLamialesLamiaceae

(Wight) A.J.Paton comb. nov.

5F88994DDCF85F759667346DD7C26DEC

urn:lsid:ipni.org:names:77201310-1

Anisochilus suffruticosus Wight, Icon. Pl. Ind. Orient. 4 (1): t. 1437. 1849. Type: India, Deccan Peninsula, Western Ghats, Nilghiri Hills, western slopes at Sisparah, Ex Herb. Wight Propr, Wight 1437 (holotype: K).

##### Distribution.

India.

#### Coleus sylvestris

Taxon classificationPlantaeLamialesLamiaceae

(Gürke) A.J.Paton & Phillipson comb. nov.

1BFE2CDB139F5BF8B5F5F08402264ACD

urn:lsid:ipni.org:names:77201311-1

Plectranthus sylvestris Gürke, Bot. Jahrb. Syst. 19: 205. 1894. Type: Tanzania, Kilimanjaro, Rifinika Hill on Mawenzi, 14 Sept. 1893, Volkens 965 (holotype: B, destroyed; isotype: BM, G).
Coleus ruwenzoriensis Baker in D.Oliver & auct. suc. (eds.), Fl. Trop. Afr. 5: 427. 1900. Types: Uganda, Ruwenzori, Scott Elliot 7556 & 7798 (syntypes: BM).
Plectranthus chiradzulensis Baker in D.Oliver & auct. suc. (eds.), Fl. Trop. Afr. 5: 412. 1900. Types: Malawi, Mt Chiradzulu summit, Meller s.n. (syntype: K (K000430788); Mt Mulanje, Whyte s.n. (syntype: K (K000430787).
Coleus ferrugineus Robyns, Bull. Jard. Bot. État Bruxelles 17: 77. 1943.
Plectranthus ferrugineus (Robyns) Troupin & Ayob. in G.M.D.Troupin, Fl. Rwanda 3: 336. 1985., nom. inval. Type: DRC, Karisimbi, Lebrun 5005 (holotype: BR; isotype: K).
Coleus subulatus Robyns, Bull. Jard. Bot. État Bruxelles 17: 76. 1943. Type: DRC, Kivu, Tschamugussa, de Witte 1854 (holotype: BR).
Plectranthus bosseri Hedge, Fl. Madag. 175: 214. 1998. Type: Madagascar, Bosser 17953 (holotype: P).

##### Distribution.

Trop. Africa, Madagascar.

#### Coleus tenuicaulis

Taxon classificationPlantaeLamialesLamiaceae

Hook.f., J. Proc. Linn. Soc., Bot. 7: 211. 1864

1285716E274F592195D345AD3A6E2B38

Fig. 5E

Majana tenuicaulis (Hook.f.) Kuntze, Revis. Gen. Pl. 2: 524. 1891.
Plectranthus tenuicaulis (Hook.f.) J.K.Morton, J. Linn. Soc., Bot. 58: 268. 1962. Type: Cameroon, Mt Cameroon, Dec.1862, Mann 1939 (holotype: K).
Coleus membranaceus Briq., Bot. Jahrb. Syst. 19: 182. 1894. Type: Angola, Cissacala (Sacalla?) in Cuango (Quango) region, Jan.1881, Mechow 554 (isotype: W).
Coleus montanus Gürke, Bot. Jahrb. Syst. 19: 218. 1894., nom. illeg., non Coleus montanus Hochst. ex Ces.
Plectranthus herbaceus Briq., Bot. Jahrb. Syst. 19: 179. 1894.
Germanea herbacea (Briq.) Hiern, Cat. Afr. Pl. 1: 861. 1900.
Coleus herbaceus (Briq.) G.Taylor, J. Bot. 69 (suppl. 2): 158. 1931. Type: Angola, Huíla, Lopolo, Mar. 1879, Welwitsch 5506 (syntypes: BM, G, K).
Plectranthus minimus Gürke, Bot. Jahrb. Syst. 19: 205. 1894. Type: Cameroon, Buea, Preuss 1019 (holotype: B, destroyed).
Coleus cunenensis Baker in D.Oliver & auct. suc. (eds.), Fl. Trop. Afr. 5: 443. 1900. Type: Angola, near Rio Cunene, Sept. 1883, Johnston s.n. (holotype: K).
Germanea andongensis Hiern, Cat. Afr. Pl. 1: 862. 1900.
Plectranthus andongensis (Hiern) K.Schum., Just’s Bot. Jahresber. 28(1): 486. 1902.
Coleus andongensis (Hiern) G.Taylor, J. Bot. 69 (suppl. 2): 158. 1931. Type: Angola, Pungo Andongo, 1856–57, Welwitsch 5543 (holotype: LISU, isotype: BM).
Germanea concinna Hiern, Cat. Afr. Pl. 1: 861. 1900.
Plectranthus concinnus (Hiern) K.Schum., Just’s Bot. Jahresber. 28(1): 486. 1902. Type: Angola, Pungo Andongo, Mata de Pungo, Apr. 1857, Welwitsch 5533 (holotype: LISU; isotype: K).
Germanea concinna var. albiflora Hiern, Cat. Afr. Pl. 1: 862. 1900. Type: Angola, Pungo Andongo, between Luxilo and Cazela, May 1857, Welwitsch 5586 (holotype: LISU; isotypes: BM, G).
Germanea concinna var. caerulea Hiern, Cat. Afr. Pl. 1: 862. 1900. Type: Angola, Pungo Andongo, Nov. 1856, Welwitsch 5521 (holotype: LISU; isotype: BM).
Coleus peulhorum A.Chev., J. Bot. (Morot) 22: 123. 1909.
Plectranthus peulhorum (A.Chev.) J.K.Morton, J. Linn. Soc., Bot. 58: 268. 1962. Type: Guinea, Fouta Djalon, 13 Sept. 1907, Chevalier s.n. (holotype: P).
Coleus drymophilus G.Taylor, J. Bot. 69 (Suppl. 2): 160. 1931. Type: Angola, Cassuango, Cuiriri, n.d., Gossweiler 4056 (holotype: BM; isotype: K).

##### Distribution.

W. Trop. Africa to Cameroon, SW. Tanzania to S. Trop. Africa.

#### Coleus tetradenifolius

Taxon classificationPlantaeLamialesLamiaceae

(A.J.Paton) A.J.Paton comb. nov.

10BE129CED205F47A330B12D8D441253

urn:lsid:ipni.org:names:77201312-1

Plectranthus tetradenifolius A.J.Paton, Fl. Trop. E. Afr., Lamiac.: 304. 2009. Type: Uganda, Karamoja District: Mt Moroto, Tweedie 2665 (holotype: K).

##### Distribution.

Cameroon, S. Sudan to E. Trop. Africa.

#### Coleus tetragonus

Taxon classificationPlantaeLamialesLamiaceae

(Gürke) Robyns & Lebrun, Ann. Soc. Sci. Bruxelles, Sér. B 49: 106. 1929

58E659BF3E5C5624931C582A11334C4D

Plectranthus tetragonus Gürke, Bot. Jahrb. Syst. 19: 209. 1894.
Englerastrum tetragonum (Gürke) T.C.E.Fr., Notizbl. Bot. Gart. Berlin-Dahlem 9: 73. 1924. Type: Tanzania, Lushoto District: Usambara Mts, Mashewa (Mascheua), Holst 3573 (holotype: B, destroyed; isotype: K, W, Z).
Plectranthus melanocarpus Gürke, Bot. Jahrb. Syst. 19: 210. 1894.
Englerastrum melanocarpum (Gürke) T.C.E.Fr., Notizbl. Bot. Gart. Berlin-Dahlem 9: 71. 1924.
Coleus melanocarpus (Gürke) Robyns & Lebrun, Ann. Soc. Sci. Bruxelles, Sér. B 49: 106. 1929. Type: Tanzania, Masai steppe, Fischer 511 (holotype: B, destroyed; isotype: K, fragment).
Plectranthus biflorus Baker in D.Oliver & auct. suc. (eds.), Fl. Trop. Afr. 5: 402. 1900.
Englerastrum biflorum (Baker) T.C.E.Fr., Notizbl. Bot. Gart. Berlin-Dahlem 9: 73. 1924. Type: Malawi, between Khondowe and Karonga, Whyte s.n. (holotype: K).
Plectranthus dekindtianus De Wild., Ann. Mus. Congo Belge, Bot., sér. 4, 2: 135. 1913. Type: DRC, Bugege, Hock s.n. (holotype: BR).

##### Distribution.

Kenya to South Africa.

#### Coleus thalassoscopicus

Taxon classificationPlantaeLamialesLamiaceae

(P.I.Forst.) P.I.Forst. comb. nov.

B5403512B4155FA7B9CFCBAD2BB41F8E

urn:lsid:ipni.org:names:77201313-1

Plectranthus thalassoscopicus P.I.Forst., Austrobaileya 4: 653. 1997. Type: Australia, Queensland, Cook District, North Bell Peak, Malbon Thompson Range, 12 Nov. 1995, P.I. Forster PIF18048, R.L. Jago & R. Jensen (holotype: BRI; isotypes: CNS, K, MEL).

##### Distribution.

Australia: Queensland (Cook).

#### Coleus thyrsoideus

Taxon classificationPlantaeLamialesLamiaceae

Baker, Bot. Mag. 125: t. 7672 1899

AF3F0A1B10AF5D4685DEAD10D3D2CF22

Plectranthus thyrsoideus (Baker) B.Mathew, Kew Bull. 31: 174. 1976. Type: Plant cultivated at Kew 3 Jan.1899; seed from herbarium specimen from N of Lake Malawi collected by Whyte (holotype: K).

##### Distribution.

S. Trop. Africa.

#### Coleus togoensis

Taxon classificationPlantaeLamialesLamiaceae

(Perkins) A.J.Paton comb. nov.

A3979C7F1E7E574584D74876EADE1CEC

urn:lsid:ipni.org:names:77201314-1

Pycnostachys togoensis Perkins, Notizbl. Bot. Gart. Berlin-Dahlem 8: 69. 1921. Type: Benin (Togo), between Kete and the Volta, Jan. 1895, Baumann 404 (B, holotype, destroyed; K, fragment).
Pycnostachys schweinfurthii Briq., Bot. Jahrb. Syst. 19: 191. 1894, non Coleus schweinfurthii Vatke. Type: Sudan, Equatoria, Dokuto (Dukuttu), Schweinfurth 2770 (holotype: B, destroyed; isotype: K).

##### Distribution.

W. Trop. Africa to Tanzania.

#### Coleus tomentifolius

Taxon classificationPlantaeLamialesLamiaceae

(Suddee) Suddee comb. nov.

F0696565C5FF52D7A6EBFB30BE8C313F

urn:lsid:ipni.org:names:77201315-1

Plectranthus tomentifolius Suddee, Kew Bull. 59: 393. 2004. Type: Thailand, Takua Thung, Nov. 1896, Curtis s.n. (holotype: SING).

##### Distribution.

Peninsula Thailand.

#### Coleus torrenticola

Taxon classificationPlantaeLamialesLamiaceae

(P.I.Forst.) P.I.Forst. comb. nov.

07BE50F80EFB586E80614B09C36D8935

urn:lsid:ipni.org:names:77201316-1

Plectranthus torrenticola P.I.Forst., Austrobaileya 3: 729. 1992. Type: Australia, Moreton District, Pooles Dam, Cooloolabin, State Forest Drive, 20 Feb. 1991, P.I. Forster PIF7797 & P.R. Sharpe (holotype: BRI; isotypes: CANB, K, MEL, NSW).

##### Distribution.

Australia: Queensland.

#### Coleus triangularis

Taxon classificationPlantaeLamialesLamiaceae

(A.J.Paton) A.J.Paton comb. nov.

DDE3A327533A5614B2BC0B96A185B09C

urn:lsid:ipni.org:names:77201317-1

Plectranthus triangularis A.J.Paton, Fl. Trop. E. Afr., Lamiac.: 313. 2009. Type: Kenya, Taita District: Ngangao Forest, Luke et al. 5497 (holotype: K; isotype: EA).

##### Distribution.

SE. Kenya to E. Tanzania.

#### Coleus trullatus

Taxon classificationPlantaeLamialesLamiaceae

(A.J.Paton) A.J.Paton comb. nov.

9F9147F91CA35923A4BF21D918A374EE

urn:lsid:ipni.org:names:77201318-1

Plectranthus trullatus A.J.Paton, Fl. Trop. E. Afr., Lamiac.: 315. 2009. Type: Tanzania, Iringa District: Udzungwa Mts, Sanje path above loggers’ camp, Bridson 641 (holotype: K).

##### Distribution.

Tanzania.

#### Coleus umbrosus

Taxon classificationPlantaeLamialesLamiaceae

Vatke, Linnaea 43: 91. 1881

EF77B007B5C55CF5B4D34E4E23068093

Pycnostachys umbrosa (Vatke) Perkins, Notizbl. Bot. Gart. Berlin-Dahlem 8: 77. 1921. Type: Kenya, Teita District: Ndara, Hildebrandt 2424 (holotype: B, destroyed).
Coleus tricholobus Gürke, Abh. Königl. Akad. Wiss. Berlin 1894: 62. 1894. Type: Tanzania, Lushoto District: W Usambara Mts, Mlalo, Holst 455 (holotype: B, destroyed; K, fragment).

##### Distribution.

Kenya to N. Tanzania.

#### Coleus unguentarius

Taxon classificationPlantaeLamialesLamiaceae

(Codd) A.J.Paton comb. nov.

B4DDD00694D75810A0DA47D3AA4BB4FE

urn:lsid:ipni.org:names:77201319-1

Plectranthus unguentarius Codd, Bothalia 11: 387. 1975. Type: Namibia, Kaokoveld, 17 km S. of Kaoko Otavi on road to Sesfontein, 21 April 1957, De Winter & Leistner 5595 (holotype: PRE).

##### Distribution.

Namibia.

#### Coleus urticifolius

Taxon classificationPlantaeLamialesLamiaceae

Benth. in A.P.de Candolle, Prodr. 12: 78. 1848

051C320AA02A5C01B93FF5BCBDD1F64A

Plectranthus urticifolius (Benth.) Hook.f., Fl. Brit. India 4: 622. 1885., nom. illeg.
Plectranthus beddomei Raizada, Indian Forester 84: 503. 1958. Type: India, Courtallum, R.Wight 622 (holotype: K (K000820175); isotype: K(K000820174)).

##### Distribution.

S. India.

#### Coleus velutinus

Taxon classificationPlantaeLamialesLamiaceae

(Trimen) A.J.Paton comb. nov.

BCABFB600AA75429B5162E5574086D89

urn:lsid:ipni.org:names:77201320-1

Anisochilus velutinus Trimen, Handb. Fl. Ceylon 3: 377. 1895. Type: Sri Lanka, Gunner’s Quoin, in the Batticaloa Distr, CP 3573 (holotype: PDA; isotypes: BM, K).
Anisochilus suffruticosus sensu Thwaites, Enum. Pl. Zeyl.: 238. 1860., non Anisochilus suffruticosus Wight; non Coleus suffrutescens (Wight) A.J.Paton.

##### Distribution.

Sri Lanka.

#### Coleus venteri

Taxon classificationPlantaeLamialesLamiaceae

(van Jaarsv. & L.Hankey) A.J.Paton comb. nov.

6290E4BF872457A0BBC26DE2498A10FD

urn:lsid:ipni.org:names:77201321-1

Plectranthus venteri van Jaarsv. & L.Hankey, Aloe 34: 40. 1997. Type: South Africa, Northern Prov., Tzaneen, Geeneinde, Schoonoord, Sekekuneland, Venter 13626 (holotype: PRE).

##### Distribution.

S. Zimbabwe to Limpopo (Leolo Mts.).

#### Coleus ventosus

Taxon classificationPlantaeLamialesLamiaceae

(P.I.Forst.) P.I.Forst. comb. nov.

E312E82D99FF575C985F32B6CB30D6E0

urn:lsid:ipni.org:names:77201322-1

Plectranthus ventosus P.I.Forst., Austrobaileya 9: 435. 2015. Type: Australia, Queensland, Cook District; ex situ cultivation from Melville Peak, Cape Melville National Park, 10 April 2015, H.B.Hines & C.J.Hoskin CM4 (holotype: BRI).

##### Distribution.

Australia: Queensland.

#### Coleus venustus

Taxon classificationPlantaeLamialesLamiaceae

(P.I.Forst.) P.I.Forst. comb. nov.

B91E751F1D9F5B168195B09DC8355B96

urn:lsid:ipni.org:names:77201323-1

Plectranthus venustus P.I.Forst., Austrobaileya 8: 401. 2011. Type: Queensland, Cook District, Kennedy Hill Gorge, 21 June 1989, P.I.Forster PIF5388 & M.C.Tucker (holotype: BRI; isotypes: CNS, MEL).

##### Distribution.

Australia: Queensland.

#### Coleus verticillatus

Taxon classificationPlantaeLamialesLamiaceae

(Baker) A.J.Paton comb. nov.

AC5129ADA2D65CE4884FDEACCE6C5C86

urn:lsid:ipni.org:names:77201324-1

Pycnostachys verticillata Baker, Bull. Misc. Inform. Kew 1895: 71. 1895. Type: Zambia, Northern Province, Fwambo, Carson 38 (holotype: K).

##### Distribution.

W. Tanzania to Zambia.

#### Coleus vettiveroides

Taxon classificationPlantaeLamialesLamiaceae

K.C.Jacob, J. Bombay Nat. Hist. Soc. 42: 320. 1941

648DE2AB4C705DEB8104DA942019FB73

Plectranthus vettiveroides (K.C.Jacob) N.P.Singh & B.D.Sharma, J. Bombay Nat. Hist. Soc. 79: 712. 1982. publ. 1983. Type: India, Coimbatore, Madras Herbarium No. 85676. (holotype: MH).
Coleus osmirrhizon Elliot, Fl. Andhrica: 105. 1859. nom. inval.

##### Distribution.

S. India.

#### Coleus veyretiae

Taxon classificationPlantaeLamialesLamiaceae

(Guillaumet & A.Cornet) A.J.Paton & Phillipson comb. nov.

E0C40A1E42F25782BE8B8CF4B54F4BA3

urn:lsid:ipni.org:names:77201325-1

Solenostemon veyretiae Guillaumet & A.Cornet, Adansonia, n.s., 15: 526. 1976.
Plectranthus veyretiae (Guillaumet & A.Cornet) Hedge, Fl. Madag. 175: 226. 1998. Type: Madagascar, Mahajanga, Sofia, Veyret 1154 (holotype: P; isotypes: K, TAN).

##### Distribution.

Madagascar.

#### Coleus villosus

Taxon classificationPlantaeLamialesLamiaceae

(Forssk.) A.J.Paton comb. nov.

F83D5F8E70235CA99B9BF538890D59E9

urn:lsid:ipni.org:names:77201326-1

Ocimum villosum Forssk., Fl. Aegypt.-Arab.: cxv, 369. 1775.
Ocimum β zatarhendi Forssk. Fl. Aegypt.-Arab.: 110. 1775.
Plectranthus villosus (Forssk.) C, Chr.,, Dansk Bot. Arkiv. 4, 21. 1922, nom. illeg., non P. villosus Sieber ex Benth.
Plectranthus arabicus E.A.Bruce, Bull. Misc. Inform. Kew 1935: 324. 1935., non Coleus arabicus Benth. Type: Yemen, between Dorebat and Taaes (Taiz), Forsskal (holotype C, destroyed). Neotype: Saudi Arabia, Wolledje, Gebel Melhan Schweinfurth 690 (neotype: K; isoneotype: BM, P; designated by Wood (1983)).

##### Distribution.

SW. Arabian Peninsula.

#### Coleus wallamanensis

Taxon classificationPlantaeLamialesLamiaceae

(T.C.Wilson & P.I.Forst.) T.C.Wilson & P.I.Forst. comb. nov.

9879B6AE966A5D06BF1C551B4CB454FC

urn:lsid:ipni.org:names:77201328-1

Plectranthus wallamanensis T.C.Wilson & P.I.Forst. Australian Systematic Botany, 31: 443. 2018. Type: Australia, Queensland:,North Kennedy District, Girringun National Park, rock fall at the base of Wallaman Falls, 9 May 2014, T.C.Wilson 521 & M.A.M.Renner (holotype: NSW 852570; isotypes: BRI, CANB).

##### Distribution.

Australia: Queensland.

#### Coleus welwitschii

Taxon classificationPlantaeLamialesLamiaceae

Briq., Bot. Jahrb. Syst. 19: 185. 1895

F747977754985EB7A80BB4B5B8D9683F

Coleus welwitschii Briq., Bot. Jahrb. Syst. 19: 185. 1895. Type. Angola, Welwitsch 5589 (isosyntypes: BM, K) & Angola, Pungo Andongo, Mechow 75 (not seen).
Coleus marquesii Briq., Annuaire Conserv. Jard. Bot. Genève 2: 239. 1898. Type: Angola, Cuango and Cuillo, Marques 191 (holotype: G).
Coleus dupuisii Briq., Bull. Soc. Roy. Bot. Belgique 37: 70. 1899.
Plectranthus dupuisii (Briq.) A.J.Paton, Fl. Trop. E. Afr., Lamiac.: 329. 2009. Type: DRC, Kasai, Mayumbe, Dupuis s.n. (holotype: BR).
Coleus eetveldeanus Briq., Bull. Soc. Roy. Bot. Belgique 37: 73. 1899. Type: Congo, Lake Tanganyika, Mtowa, Deschamps s.n. (holotype: BR).
Coleus betonicoides Baker ex Hiern, Cat. Afr. Pl. 1: 866. 1900.
Calchas betonicoides (Baker ex Hiern) P.V.Heath, Calyx 5: 160. 1997. Type: Angola, Huila, Lopollo, Ferrão da Sola, Welwitsch 5612 (holotype: LISU; isotypes: BM, G, K).
Plectranthus microphyllus Baker in D.Oliver & auct. suc. (eds.), Fl. Trop. Afr. 5: 410. 1900., nom. illeg.
Plectranthus porpeodon Baker in D.Oliver & auct. suc. (eds.), Fl. Trop. Afr. 5: 525. 1900.
Solenostemon porpeodon (Baker) J.K.Morton, Novon 8: 266. 1998. Type: Tanzania, Tanga District: Magila, Kirk s.n. (holotype: K).
Coleus newtonii Briq., Bull. Herb. Boissier, sér. 2, 6: 826. 1906. Type: Angola, Mossamedes, Huila, Newton 105 (holotype: Z).
Coleus delpierrei De Wild., Bol. Soc. Ibér. Ci. Nat. 19: 119. 1920. Types: DRC, Vankerkhovenville, Delpierre s.n. (syntype: BR) & Niangara, Delpierre s.n. (syntype: BR).
Coleus laurentii De Wild., Bol. Soc. Ibér. Ci. Nat. 19: 121. 1920. Type: DRC, Gombe, E. & M. Laurent s.n. (holotype: BR).
Coleus thyrsiflorus Lebrun & L.Touss. in J.-P.A.Lebrun, Expl. Parc Nat. Kagera 1: 120. 1948.
Solenostemon thyrsiflorus (Lebrun & L.Touss.) Vollesen, Opera Bot. 59: 85. 1980. Type: DRC, Kagera, Nyakayaga, Lebrun 9447 (holotype: BR; isotype: K).

##### Distribution.

Ethiopia to S. Trop. Africa.

#### Coleus xanthanthus

Taxon classificationPlantaeLamialesLamiaceae

C.Y.Wu & Y.C.Huang, Acta Phytotax. Sin. 10: 241. 1965

1EEC6C5E1E955A4C8E1D850A00530C68

Coleus xanthanthus C.Y.Wu & Y.C.Huang, Acta Phytotax. Sin. 10: 241. 1965. Type: China, Yunnan, Mon-lun, 15 Sept. 1959, S.K.Pei (holotype: HK).

##### Distribution.

China: Yunnan.

#### Coleus xerophilus

Taxon classificationPlantaeLamialesLamiaceae

(Codd) A.J.Paton comb. nov.

D68970B37A78550FB00E0D6E8E7ECB4B

urn:lsid:ipni.org:names:77201329-1

Plectranthus xerophilus Codd, Bothalia 11: 282. 1974. Type: South Africa, Transvaal, Lydenburg, district, near Marone, Codd & Dyer 7729 (holotype: PRE).

##### Distribution.

South Afica: Northern Prov.

#### Coleus xylopodus

Taxon classificationPlantaeLamialesLamiaceae

(Lukhoba & A.J.Paton) A.J.Paton comb. nov.

B8AD155863D75B509AE9F1CE9EF761DD

urn:lsid:ipni.org:names:77201330-1

Plectranthus xylopodus Lukhoba & A.J.Paton, Kew Bull. 55: 960. 2000. Type: Kenya, Trans-Nzoia District: S Cherangani, Symes 293 (holotype: EA; isotype: K).
Ocimum cinereum R.Br. in H.Salt, Voy. Abyss.: 64. 1814., nom. nud.

##### Distribution.

S. Ethiopia to Uganda.

#### Coleus yemenensis

Taxon classificationPlantaeLamialesLamiaceae

A.J.Paton nom. nov.

97F42180348553CF9D5F47C8E4D1B24B

urn:lsid:ipni.org:names:77201331-1

Plectranthus ovatus Benth., Labiat. Gen. Spec.: 709 1835., non Coleus ovatus Benth.
Ocimum gratissimum Forssk., Fl. Aegypt.-Arab.: 110. 1775., nom. illeg. Type: Yemen, July 1763, Forsskal 350 (holotype: C).

##### Distribution.

Arabian Peninsula.

#### Coleus zombensis

Taxon classificationPlantaeLamialesLamiaceae

(Baker) Mwany. comb. nov.

6D246A427E1D574FAA093B8C87C5D451

urn:lsid:ipni.org:names:77201332-1

Plectranthus zombensis Baker in D.Oliver & auct. suc. (eds.), Fl. Trop. Afr. 5: 402. 1900. Type: Malawi, summit of Mt Zomba, 1881, Buchanan 395 (holotype: K).
Plectranthus lastii Baker in D.Oliver & auct. suc. (eds.), Fl. Trop. Afr. 5: 403. 1900. Type: Malawi, near Blantyre, Jan. 1887, Last s.n. (holotype: K).

##### Distribution.

S. Malawi.

### Genus *Equilabium*

#### Equilabium

Taxon classificationPlantaeLamialesLamiaceae

Mwany., A.J. Paton & Culham, Bot. J. Linn. Soc. 188(4): 367. 2018

8C5FF8F2207654C2B8BFE28B2052CE1D

Figs 6 , 7

##### Type species.

*Equilabium laxiflorum* (Benth.) Mwany., A.J.Paton & Culham.

##### Description.

Annual or perennial herbs or soft wooded shrubs, rarely woody shrubs, sometimes with a persistent woody or fleshy rootstock. Leaves opposite. Inflorescence thrysoid, usually lax; cymes sessile or pedunculate, 1–3(–5, very rarely –7); bracts subtending cymes persistent. Flowers pedicelate. Calyx funnel-shaped, straight with pedicel attaching symmetrically at the calyx base, not opposite posterior lip, two-lipped; throat glabrous within, open; posterior lip 1-lobed, usually broader than anterior lobes, shortly decurrent on tube or not; anterior lip 4-lobed; lobes triangular or lancolate; lateral lobes broader or equal to median lobes and held midway between the posterior lobe and the median lobes of the anterior lip; median lobes triangular to lanceolate. Corolla two-lipped with lips equal in length; tube sigmoid, sometimes strongly so, narrow and parallel-sided at base and expanding towards throat; posterior lip 4-lobed erect or ascending; anterior lobe horizontal, cucullate, sometimes frilled at apex. Stamens 4; filaments not fused together, held within the anterior lip. Style bifid with lobes subulate. Nutlets ovoid.

**Figure 6.**
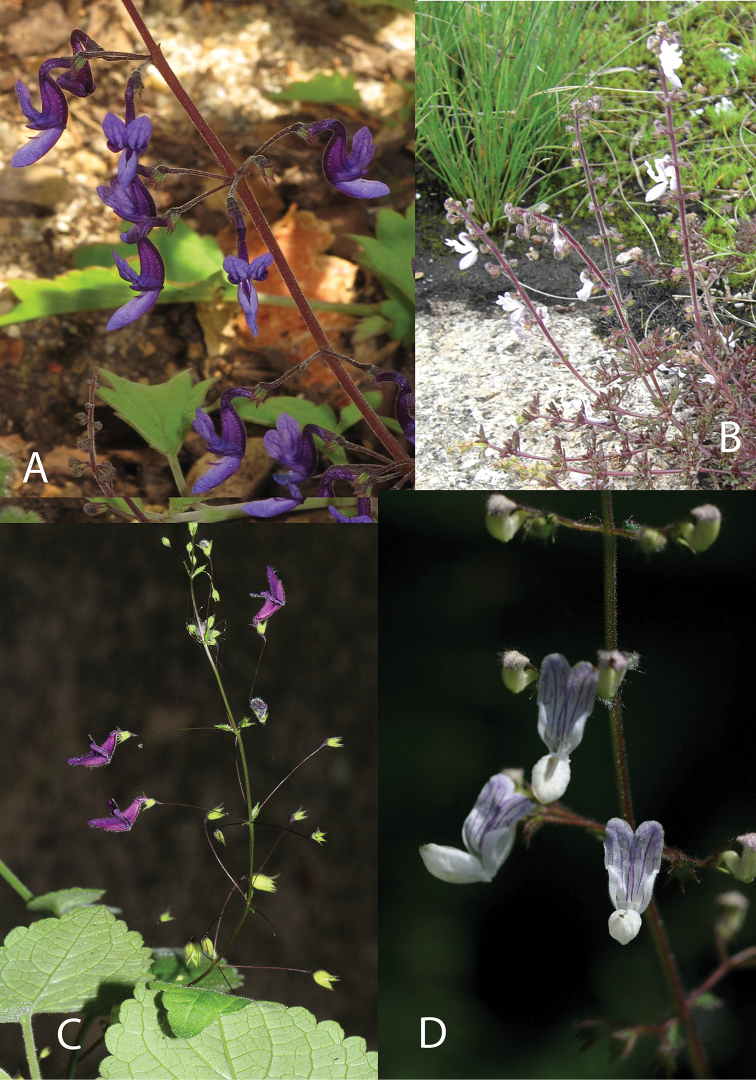
Species of *Equilabium* 1. **A***Equilabium dolomiticum* (Photo: Neil Crouch) **B***E. dissectum* var. dissectum (Photo: Montfort Mwanyambo) **C***E. gracilum* (Photo: Bart Wursten) **D***E. laxiflorum* (Photo: Bart Wursten).

**Figure 7.**
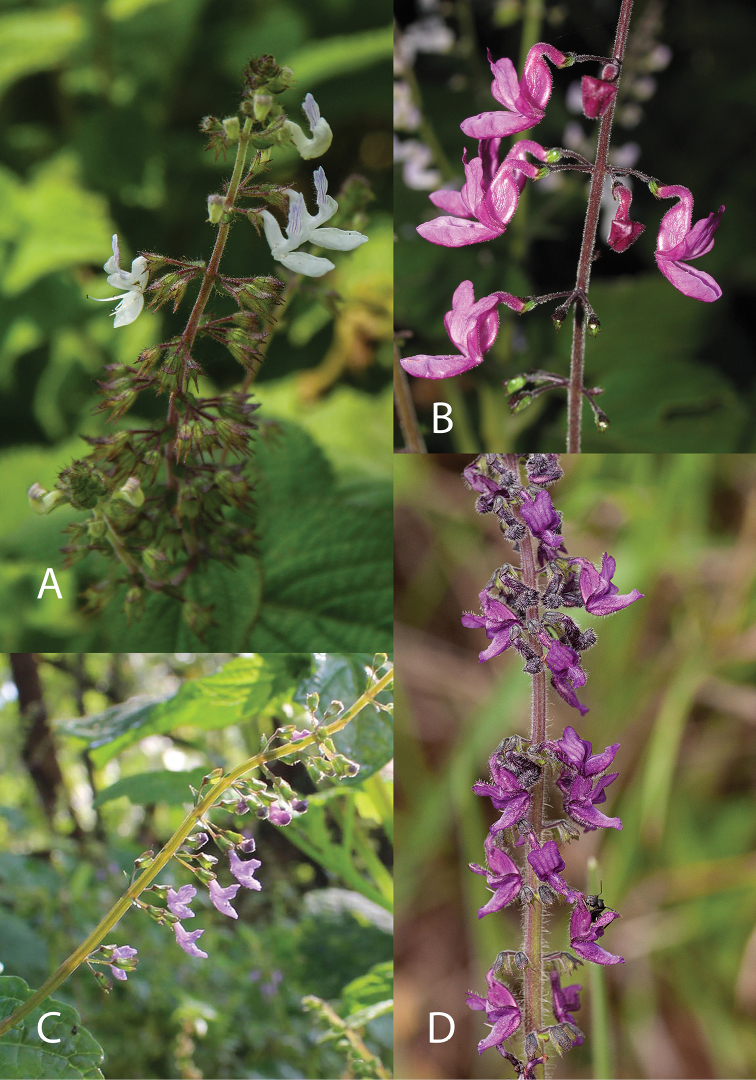
Species of *Equilabium* 2. **A***Equilabium laxiflorum* (Photo: Bart Wursten) **B***E. petiolare* (Photo: Neil Crouch) **C***E. subincisum* (Photo: K. Smitha) **D***E. stenophyllum* (Photo: Bart Wursten).

42 species mainly in Tropical Africa but including two in India.

#### Equilabium acaule

Taxon classificationPlantaeLamialesLamiaceae

(Brummitt & Seyani) Mwany., Culham & A.J.Paton comb. nov.

DBEFD0556EEC554496DD7906D37AB609

urn:lsid:ipni.org:names:77201333-1

Plectranthus acaulis Brummitt & Seyani, Kew Bull. 42: 695. 1987. Type: Malawi, Nyika Plateau, 8. Dec. 1965, Banda 760 (holotype: K; isotype: MAL).

##### Distribution.

Malawi to Zambia (Nyika Plateau).

#### Equilabium agnewii

Taxon classificationPlantaeLamialesLamiaceae

(Lukhoba & A.J.Paton) Mwany. & A.J.Paton comb. nov.

711E1CB515EE5F11BF9DBD2C91073DC0

urn:lsid:ipni.org:names:77201334-1

Plectranthus agnewii Lukhoba & A.J.Paton, Kew Bull. 55: 957. 2000. Type: Kenya, Nakuru National Park, W of Lake, Gillett 20847 (holotype: EA; isotype: K).

##### Distribution.

E. Tropical Africa.

#### Equilabium annuum

Taxon classificationPlantaeLamialesLamiaceae

(A.J.Paton) Mwany. & A.J.Paton comb. nov.

B17C544FD85E55CBB4CF9E643DE16EB1

urn:lsid:ipni.org:names:77201335-1

Plectranthus annuus A.J.Paton, Fl. Trop. E. Afr., Lamiac.: 270. 2009. Type: Tanzania, Masasi District: Ndanda Mission, Bidgood, Abdallah & Vollesen 2077 (holotype: K (sheet 2); isotypes: BR, C, DSM, EA, K (sheet 1), NHT).

##### Distribution.

S. Tanzania to Mozambique.

#### Equilabium caespitosum

Taxon classificationPlantaeLamialesLamiaceae

(Lukhoba & A.J.Paton) Mwany. & A.J.Paton comb. nov.

FCECDABED747591683C62F135B5556D1

urn:lsid:ipni.org:names:77201336-1

Plectranthus caespitosus Lukhoba & A.J.Paton, Kew Bull. 58: 910. 2003, publ. 2004. Type: Kenya, E Mt Elgon, Irwin 286 (holotype: EA; isotype: K).

##### Distribution.

Kenya to N. Tanzania.

#### Equilabium candelabriforme

Taxon classificationPlantaeLamialesLamiaceae

(Launert) Mwany. & A.J.Paton comb. nov.

6E4C9769421D5D12B182A90B7CB9173C

urn:lsid:ipni.org:names:77201337-1

Plectranthus candelabriformis Launert, Mitt. Bot. Staatssamml. München 7: 300. 1968. Type: Namibia, 16 km E of Runtu, 7 Mar. 1958, Merxmüller & Giess 1912 (holotype: M).

##### Distribution.

Tanzania to N. Namibia.

#### Equilabium cinereum

Taxon classificationPlantaeLamialesLamiaceae

(A.J.Paton) Mwany. & A.J.Paton comb. nov.

62AF822C3642523092FC576092051359

urn:lsid:ipni.org:names:77201338-1

Plectranthus cinereus A.J.Paton, Fl. Trop. E. Afr., Lamiac.: 247. 2009. Type: Tanzania, Mbulu District: Lake Manyara National Park, Ndala R., Greenway & Kanuri 11151 (holotype: K; isotype: EA).

##### Distribution.

Kenya to Tanzania.

#### Equilabium dissectum

Taxon classificationPlantaeLamialesLamiaceae

(Brenan) Mwany. & A.J.Paton comb. nov.

D7F6EFB0A06153D8A6C6FA07DD5F2375

urn:lsid:ipni.org:names:77201339-1

Plectranthus dissectus Brenan, Mem. New York Bot. Gard. 9: 42. 1954. Type: Malawi, Zomba Plateau, 31 May 1946, Brass 16124 (holotype: K; isotypes: MO, PRE).

##### Distribution.

S. Malawi.

#### Equilabium dissectum var. dissectum

Taxon classificationPlantaeLamialesLamiaceae

01ECF23866825ABAA37246622976C5BF

Fig. 6B

Plectranthus dissectus Brenan var. dissectus A.J.Paton, Fl. Zambes. 8(8): 224. 2013.

#### Equilabium dissectum

Taxon classificationPlantaeLamialesLamiaceae

var. chambense (A.J.Paton) Mwany. & A.J. Paton comb. nov.

024134DB7F2F53F09BBF00EA097FF789

urn:lsid:ipni.org:names:77201340-1

Plectranthus dissectus Brenan var. chambensis A.J.Paton, Fl. Zambes. 8(8): 224. 2013. Type: Malawi, Mulanje, Chambe basin, 4 May1989, Goyder, Pope & Radcliffe-Smith 3205 (holotype: K).

##### Distribution.

S. Malawi.

#### Equilabium dolomiticum

Taxon classificationPlantaeLamialesLamiaceae

(Codd) Mwany. & A.J.Paton comb. nov.

9739644D0B795AD09898B116CF07BD78

urn:lsid:ipni.org:names:77201341-1

Fig. 6A

Plectranthus dolomiticus Codd, Bothalia 15: 142. 1984. Type: South Africa, Transvaal, Farm Ostend, 7 km NW of Penge Mine, 10 Dec. 1982, Van Jaarsveld 7052 (holotype, PRE).

##### Distribution.

Zimbabwe to Limpopo.

#### Equilabium equisetiforme

Taxon classificationPlantaeLamialesLamiaceae

(E.A.Bruce) Mwany. & A.J.Paton comb. nov.

06B2D414014253FD80DC35C51A96AD4F

urn:lsid:ipni.org:names:77201342-1

Coleus equisetiformis E.A. Bruce in Bull. Misc. Inform., Kew 1935: 285. 1935.
Plectranthus equisetiformis (E.A. Bruce) Launert in Mitt. Bot. Staatssamml. München 7: 299. 1968. Type: Tanzania, Mpwapwa, Kiboriani Mts, 19 Apr. 1932, Burtt 3892 (holotype: K).

##### Distribution.

Tanzania to N. Zambia.

#### Equilabium flaccidum

Taxon classificationPlantaeLamialesLamiaceae

(Vatke) Mwany. & A.J.Paton comb. nov.

F3D6F235C07A5022A211C89BEE1DDAC5

urn:lsid:ipni.org:names:77201343-1

Coleus flaccidus Vatke in Linnaea 43: 9. 1881.
Plectranthus flaccidus (Vatke) Gürke, Bot. Jahrb. Syst. 19: 206. 1894. Type: Tanzania, Bagamoyo District: Ruvu [Kingani] R., Hildebrandt 1264 (holotype: B, destroyed; isotype: K).
Plectranthus fragilis Baker in Fl. Trop. Afr. 5: 407. 1900. Type: Zanzibar, Kirk s.n. (holotype: K).

##### Distribution.

S. Somalia to Mozambique, Comoros.

#### Equilabium glandulosum

Taxon classificationPlantaeLamialesLamiaceae

(Hook. f.) Mwany. & A.J.Paton comb. nov.

A703241C3C485415A4CE37BB3D68C4B7

urn:lsid:ipni.org:names:77201344-1

Plectranthus glandulosus Hook.f., J. Proc. Linn. Soc., Bot. 6: 17. 1862. Type: Bioko [Fernando Po], Clarence Peak, Mann 318 (holotype: K).
Plectranthus hylophilus Gürke, Bot. Jahrb. Syst. 19: 283. 1894. Type: Cameroon, Mann’s Spring, Preuss 815 (holotype B, destroyed; isotype: K).
Plectranthus laxiflorus var. genuinus Briq., Bot. Jahrb. Syst. 19: 180. 1894., nom. inval.
Germanea laxiflora var. genuina (Briq.) Hiern, Cat. Afr. Pl. 1: 861. 1900., nom. inval. Based on: Angola, Pungo Andongo, Welwitsch 5545 (BM, COI, K, PRE).
Plectranthus phryxotrichus Briq., Bull. Soc. Roy. Bot. Belgique 37: 66. 1899. Type: Congo-Kinshasa, Dewèvre 862 a (holotype: BR).
Plectranthus urticoides Baker in D.Oliver & auct. suc. (eds.), Fl. Trop. Afr. 5: 412. 1900. Type: Angola, Pungo Andongo, Welwitsch 5545 (holotype: LISU; isotype: K).
Plectranthus almamii A.Chev., J. Bot. (Morot) 22: 120. 1909. Type: Guinea, Conakry, Diagussa, around Diagussa and Dalaba, Oct. 1907, A.Chevalier 18609 (holotype: P; isotype: P).

##### Distribution.

Widespread in Trop. Africa.

#### Equilabium goetzei

Taxon classificationPlantaeLamialesLamiaceae

(Gürke) Mwany. & A.J.Paton comb. nov.

F75E54B95476502EBD582F0E162ADA91

urn:lsid:ipni.org:names:77201345-1

Plectranthus goetzei Gürke, Bot. Jahrb. Syst. 28: 469. 1900. Type: Tanzania, Iringa Dist., Rungemba, Mar. 1899, Goetze 724 (holotype: B, destroyed; isotype: BM).
Plectranthus salubenii Brummitt & Seyani, Kew Bull. 42: 690. 1987. Type: Malawi, Katumbi Escarpment, 41.5 km N of Rumphi, 26 Dec. 1970, Pawek 4155 (holotype: K).

##### Distribution.

SW. Tanzania to Zambia.

#### Equilabium gracile

Taxon classificationPlantaeLamialesLamiaceae

(Suess.) Mwany. & A.J.Paton comb. nov.

71157292C0E05E7E8EA668EA79AAC2BF

urn:lsid:ipni.org:names:77201346-1

Fig. 6E

Plectranthus gracilis Suess., Trans. Rhodesia Sci. Assoc. 43: 52. 1951. Type: Zimbabwe, Marondera (Marandellas), 26 Feb. 1943, Dehn 748 (holotype: M).
Plectranthus zernyi Gilli, Ann. Naturhist. Mus. Wien 77: 39. 1973. Type: Tanzania, Matengo Highlands, WSW from Songea, Ugano, 2 Mar. 1936, Zerny 483 (holotype: W; isotype: K).
Plectranthus seyanii A.J.Paton & Brummitt, Kew Bull. 46: 523. 1991. Type: Malawi, Shire Valley, 16 km W of Zomba, 12 Mar. 1977, Brummitt & Patel 14821 (holotype: K; isotypes: MAL, SRGH, WAG).

##### Distribution.

S. Tanzania to S. Trop. Africa.

#### Equilabium intrusum

Taxon classificationPlantaeLamialesLamiaceae

(Briq.) Mwany. & A.J.Paton comb. nov.

2C07F7D6CE5E56F28D206E137B98AD2D

urn:lsid:ipni.org:names:77201347-1

Plectranthus intrusus Briq., Bull. Herb. Boissier, sér. 2, 1: 834. 1901. Type: DRC, N’Dembo, ann. 1898, J.Gillet s.n. (holotype: BR).

##### Distribution.

DRC.

#### Equilabium janthinothryx

Taxon classificationPlantaeLamialesLamiaceae

(Lebrun & L. Touss) Mwany. & A.J.Paton comb. nov.

72B8E98639D65ABBB0F64932FEB0BC18

urn:lsid:ipni.org:names:77201348-1

Plectranthus janthinothryx Lebrun & L.Touss., Bull. Jard. Bot. État Bruxelles 17: 69. 1943. Type: DRC, Rwindi, Lebrun 7930 (holotype: BR, isotype: K).

##### Distribution.

DRC and Uganda.

#### Equilabium jebel-marrae

Taxon classificationPlantaeLamialesLamiaceae

(Wickens & B.Mathew) A.J.Paton comb. nov.

C3DD58E53F365EAD88019E33E239E5C2

urn:lsid:ipni.org:names:77201349-1

Plectranthus jebel-marrae Wickens & B.Mathew, Kew Bull. 25: 255. 1971. Type: Sudan, Darfur Province: Kulme, Sept. 1921. Lynes 589 (holotype: BM).

##### Distribution.

Sudan.

#### Equilabium kamerunense

Taxon classificationPlantaeLamialesLamiaceae

(Gürke) Mwany. & A.J.Paton comb. nov.

C140C58CE4195CB28EDB5C39BD95B8DB

urn:lsid:ipni.org:names:77201350-1

Plectranthus kamerunensis Gürke, Bot. Jahrb. Syst. 19: 202. 1894. Type: Cameroon, West Buea, Preuss 1039 (holotype: B, destroyed).
Plectranthus mannii Baker in D.Oliver & auct. suc. (eds.), Fl. Trop. Afr. 5: 408. 1900. Type: Cameroon, Mt Cameroon, Mann 1947 (holotype: K).
Plectranthus keniensis S.Moore, J. Bot. 1901: 264. 1901. Type: Kenya, Mt Kenya, Mackinder s.n. (holotype: BM).
Plectranthus sangerawensis Gürke, Bot. Jahrb. Syst. 36: 134. 1905. Types: Tanzania, E Usambara Mts, Sangerawe, Mongu, Engler 872 & Amani, Warnecke in Herb. Amani 481 (syntypes: B, destroyed).
Plectranthus aberdaricus T.C.E.Fr., Notizbl. Bot. Gart. Berlin-Dahlem 11: 27. 1930. Type: Kenya, Aberdares, R.E. & T.C.E. Fries 2784 (isotype: BM).
Plectranthus sangerawensis var. glabrior T.C.E.Fr., Notizbl. Bot. Gart. Berlin-Dahlem 11: 25. 1930. Types: Kenya, W Mt Kenya, R.E. & T.C.E. Fries 514, 518, 843 & Aberdares, R.E. & T.C.E. Fries 2443a (syntypes: UPS; isosyntype of 518: K).
Plectranthus vicinus T.C.E.Fr., Notizbl. Bot. Gart. Berlin-Dahlem 11: 27. 1930. Type: Kenya, Meru, R.E. & T.C.E. Fries 1580 (isotypes: K, MO).

##### Distribution.

Nigeria to Cameroon, E. Trop. Africa.

#### Equilabium laxiflorum

Taxon classificationPlantaeLamialesLamiaceae

(Benth.) Mwany. & A.J.Paton, Bot. J. Linn. Soc. 188(4): 367. 2018

54923CB65CF15BE69A019D3FC57CB97E

Figs 6D , 7A

Plectranthus laxiflorus Benth. in E.H.F.Meyer, Comm. Pl. Afr. Austr.: 228. 1838.
Germanea laxiflora (Benth.) Hiern, Cat. Afr. Pl. 1: 861. 1900.
Coleus laxiflorus (Benth.) Roberty, Bull. Inst. Fondam. Afrique Noire, Sér. A, Sci. Nat. 16: 331. 1954. Type: South Africa, KwaZulu-Natal, between Umzimkulu and Umkomaas Rivers, Drège 3586 (lectotype: K; designated by Codd (1975)).
Plectranthus albus Gürke, Bot. Jahrb. Syst. 19: 202. 1894. Type: Tanzania, Moshi District: Marangu, Volkens 744 (holotype: B, destroyed, isotype: BM).
Plectranthus violaceus Gürke, Bot. Jahrb. Syst. 19: 201. 1894. Type: Tanzania, Lushoto District: Usambara Mts, near Lutindi, Holst 3317 (holotype: B, destroyed; isotypes: K, W).
Plectranthus johnstonii Baker in D.Oliver & auct. suc. (eds.), Fl. Trop. Afr. 5: 411. 1900. Type: Tanzania, Kilimanjaro, H.H. Johnston 69 (holotype: K).
Plectranthus kondowensis Baker in D.Oliver & auct. suc. (eds.), Fl. Trop. Afr. 5: 417. 1900. Type: Malawi, between Khondowe and Karonga, 1896, Whyte s.n. (holotype: K).
Plectranthus triflorus Baker in D.Oliver & auct. suc. (eds.), Fl. Trop. Afr. 5: 417. 1900. Type: Tanzania, Kilimanjaro, Thomson s.n. (holotype: K).
Plectranthus neumannii Gürke, Bot. Jahrb. Syst. 36: 131. 1905. Types: Ethiopia, Gofa, Neumann 168 & 179 (syntype: B, destroyed); neotype: Ethiopia, Gamo Gofa, Busa Forest, near Gidole. Ryding et al. 1630 (neotype: UPS; isoneotype: ETH designated by Ryding (2000)).
Plectranthus lilacinus Gürke, Bot. Jahrb. Syst. 41: 315. 1908. Types: Tanzania, Kilimanjaro, Engler 1797 (syntype: B, destroyed) & Moshi, Uhlig 137 (isosyntype: EA).
Coleus keniensis Standl., Smithsonian Misc. Collect. 68(5): 14. 1917. Type: Kenya, W of Mt Kenya, Mearns 1334 (holotype: US; isotype: BM).
Plectranthus fraternus T.C.E.Fr., Notizbl. Bot. Gart. Berlin-Dahlem 11: 26. 1930. Type: Kenya W Mt Kenya, between Cole’s Mill and Forest Station, R.E. & T.C.E. Fries 911 (lectotype UPS; isolectotype K, MO; designated by Paton et al. (2009)).

##### Distribution.

Ethiopia to South Africa.

#### Equilabium longipes

Taxon classificationPlantaeLamialesLamiaceae

(Baker) Mwany. & A.J.Paton comb. nov.

7BA213878CC85B499CA7F0C43DE07A02

urn:lsid:ipni.org:names:77201351-1

Plectranthus longipes Baker in D.Oliver & auct. suc. (eds.), Fl. Trop. Afr. 5: 406. 1900. Type: Eritrea, Ghinda, Schweinfurth 178 (holotype: K, isotype: G).
Plectranthus amaniensis Gürke, Bot. Jahrb. Syst. 36: 134. 1905. Types: Tanzania, Lushoto District: E Usambara Mts, between Amani & Kwambugo, Engler 783 & same vicinity, Warnecke in Herb. Amani 413a (syntypes: B, destroyed).
Plectranthus ellenbeckii Gürke, Bot. Jahrb. Syst. 36: 132. 1905. Types: Ethiopia, Gallaland, Belana, Ellenbeck 334 (holotype: B, destroyed); neotype: Ethiopia, Harerge Region, Awalla Valley, Jansen 1952 (neotype: WAG, designated by Ryding (2000)).
Plectranthus panganensis Gürke, Bot. Jahrb. Syst. 41: 314. 1908. Type: Kenya, Kwale District: Samburu, Kassner 483 (lectotype: BM, designated by Paton et al. (2009)).
Plectranthus emanueli Buscal. & Muschl., Bot. Jahrb. Syst. 49: 484. 1913. Types: Kenya, Guaso Nyiro valley, but probably from elsewhere, von Aosta 1558 (holotype: B, destroyed); neotype: Eritrea, Nefasit, Mt Bizen, Ryding et al. 2034 (neotype: UPS; isoneotype: ASMU, ETH, designated by Ryding (2001)).
Plectranthus buraensis S.Moore, J. Bot. 54: 290. 1916. Type: Kenya, Teita District: Bura, A. Buchanan s.n. (holotype: BM).

##### Distribution.

Eritrea to Rwanda and Tanzania.

#### Equilabium mafiense

Taxon classificationPlantaeLamialesLamiaceae

(A.J.Paton) Mwany. & A.J.Paton comb. nov.

7EE497E22892528A888DAA6EF5E2DCB7

urn:lsid:ipni.org:names:77201352-1

Plectranthus mafiensis A.J.Paton, Fl. Trop. E. Afr., Lamiac.: 267. 2009. Type: Tanzania, Mafia I., Kikutani, Greenway 5218 (holotype: EA; isotype: K).

##### Distribution.

Tanzania (Mafia I.).

#### Equlabium masukense

Taxon classificationPlantaeLamialesLamiaceae

(Baker) Mwany. & A.J.Paton comb. nov.

61265E75F03C5B41835E9BFF5DE29846

urn:lsid:ipni.org:names:77201353-1

Plectranthus masukensis Baker in D.Oliver & auct. suc. (eds.), Fl. Trop. Afr. 5: 405. 1900. Type: Malawi, Masuku (Masuka) Plateau, Whyte 299 (holotype: K).

##### Distribution.

Kenya to N. Zambia.

#### Equilabium masukense

Taxon classificationPlantaeLamialesLamiaceae

(Baker) Mwany. & A.J.Paton var. masukense.

994A102254425A6FA4188A5A2BAC63E3

Orthosiphon nyasicus Baker in D.Oliver & auct. suc. (eds.), Fl. Trop. Afr. 5: 373. 1900.
Plectranthus nyasicus (Baker) M.R.Ashby, J. Bot. 76: 47. 1938. Type: Malawi, “north Nyassa”, 1896, Whyte s.n. (holotype: K).
Plectranthus brevipes Baker in D.Oliver & auct. suc. (eds.), Fl. Trop. Afr. 5: 406. 1900. Type: Malawi, Nyika Plateau, Whyte s.n. (lectotype: K (K000430762), designated by Paton et al. (2009)).

##### Distriution.

Kenya to N. Zambia.

#### Equilabium masukense

Taxon classificationPlantaeLamialesLamiaceae

(Baker) Mwany. & A.J.Paton var. dissectum (A.J.Paton) A.J.Paton comb. nov.

0BF42AA7235C5C8082C70DC80EF549F8

urn:lsid:ipni.org:names:77201354-1

Plectranthus masukensis var. dissectus A.J.Paton, Fl. Trop. E. Afr., Lamiac.: 264. 2009. Type: Tanzania, Ufipa District: Sumbawanga, Tatanda Mission, Bidgood, Mbago & Vollesen 2383 (holotype: K; isotype: DSM).

##### Distribution.

W. Tanzania.

#### Equilabium megafolium

Taxon classificationPlantaeLamialesLamiaceae

(A.J.Paton) Mwany. & A.J.Paton comb. nov.

9DF04E568C2857D39FCCDCE23DAFD1B1

urn:lsid:ipni.org:names:77201355-1

Plectranthus megafolius A.J.Paton, Fl. Zambes. 8(8): 217. 2013.
Plectranthus macrophyllus A.J.Paton, nom. illegit. in F.T.E.A., Lamiaceae: 262. 2009, non Blume, Bijdr. Fl. Ned. Ind. 14: 835, 1826. Type: Tanzania, Pare Dist., Mkomazi Game Reserve, 6 km on Kisiwani–Mnazi road, Abdallah & Vollesen 95/181 (holotype: K; isotypes: C, NHT, P).

#### Equilabium molle

Taxon classificationPlantaeLamialesLamiaceae

(Aiton) Mwany. & A.J.Paton comb. nov.

9DF371E39DCE5B3284D8FC33A536FF72

urn:lsid:ipni.org:names:77201356-1

Ocimum molle Aiton, Hort. Kew. 2: 322. 1789.
Plectranthus mollis (Aiton) Spreng., Syst. Veg. 2: 690. 1825.
Plectranthus secundus Roxb., Fl. Ind. ed. 1832, 3: 20. 1832. Nom. superfl. based on Ocimum molle Aiton. Type: Cultivated, native of the East Indies. Introduced by Sir Joseph Banks in 1781 (holotype: BM).
Ocimum maypurense Roth, Nov. Pl. Sp.: 271. 1821.
Plectranthus maypurensis (Roth) Spreng., Syst. Veg. 2: 691. 1825. Type: India, Heyne s.n. in Wall. Cat. 2736C (holotype B, destroyed; isotypes: K(K000820180), K-W (K001116983)).
Plectranthus incanus Link, Enum. Hort. Berol. Alt. 2: 120. 1822. Type: Cultivated, (B, destroyed).
Plectranthus divaricatus Weinm., Syll. Pl. Nov. 1: 68. 1824. Type: Cultivated (not seen).
Ocimum cordifolium Hamilton in D.Don Prodr. Fl. Nepal.: 116. 1825. nom inval.
Plectranthus cordifolius Buch.-Ham. ex D.Don, Prodr. Fl. Nepal.: 116. 1825. Type: Nepal, Wall. Cat. 2736A (holotype: not at BM; neotype: K (K001116984), isoneotype: K (K000820186); K-W (K001116982) designated by Suddee et al. (2004)).

##### Distribution.

Indian Subcontinent to N. Myanmar.

#### Equilabium orbiculare

Taxon classificationPlantaeLamialesLamiaceae

(Gürke) Mwany. & A.J.Paton comb. nov.

C81B943509F352D0B2CCFB050559B1D5

urn:lsid:ipni.org:names:77201357-1

Plectranthus orbicularis Gürke, Bot. Jahrb. Syst. 19: 203. 1894. Types: Tanzania, Usambara Mts, Upanga swamp, Holst 4159 (syntype: B, destroyed) & Zanzibar, Stuhlmann 826 (syntype: B, destroyed; isosyntype: HBG).
Plectranthus sphaerophyllus Baker in D.Oliver & auct. suc. (eds.), Fl. Trop. Afr. 5: 410. 1900. Types: Tanzania, Uzaramo District: Dar es Salaam, Kirk s.n. (syntype: K) & Zanzibar, Kirk s.n. (syntype: K).

##### Distribution.

E. Tanzania (incl. Zanzibar, Pemba).

#### Equilabium parvum

Taxon classificationPlantaeLamialesLamiaceae

(Oliv.) Mwany. & A.J.Paton comb. nov.

53C4FB3DD1B95FBBA4C671C55866031B

urn:lsid:ipni.org:names:77201358-1

Plectranthus parvus Oliv., Trans. Linn. Soc. London, Bot. 2: 347. 1887. Type: Tanzania, Kilimanjaro, H.H. Johnston s.n. (holotype: K).

##### Distribution.

Uganda to N. Zambia.

#### Equilabium pauciflorum

Taxon classificationPlantaeLamialesLamiaceae

(Baker) Mwany. & A.J.Paton comb. nov.

D6AA0B7F25CA56C58DB89D04C38923A9

urn:lsid:ipni.org:names:77201359-1

Plectranthus pauciflorus Baker in D.Oliver & auct. suc. (eds.), Fl. Trop. Afr. 5: 405. 1900. Type: Tanzania, Shinyanga District: Msalala [Misilala], Hannington s.n. (holotype: K).
Plectranthus ugandensis S.Moore, J. Linn. Soc., Bot. 37: 200. 1903. Type: Uganda, Kigezi District: Rukiga [Ruchigga], Bagshawe 532 (holotype: BM).
Plectranthus auriculatus Robyns & Lebrun, Rev. Zool. Bot. Africaines 16: 353. 1928. Type: DRC, Kivu, Saké, Katouzi, Robyns 2486 (holotype: BR, isotype: K).

##### Distribution.

DRC, East Africa, Zambia.

#### Equilabium petiolare

Taxon classificationPlantaeLamialesLamiaceae

(Benth.) Mwany. & A.J.Paton comb. nov.

6D0182B438575D419A2D9E96B7745E59

urn:lsid:ipni.org:names:77201360-1

Fig. 7B

Plectranthus petiolaris Benth. in E.H.F.Meyer, Comm. Pl. Afr. Austr.: 228. 1838. Type: South Africa, Eastern Cape, between Umtata and Umzimvubu Rivers, 1836, Drège 4773b (K lectotype), lectotypified by Codd (1975).
Plectranthus kuntzei Gürke in C.E.O.Kuntze, Revis. Gen. Pl. 3(2): 260. 1898. Type: South Africa, KwaZulu-Natal, Clairmont, 18 Oct. 1913, Kuntze s.n. (holotype: NY; isotype: K).

##### Distribution.

S. Mozambique to South Africa.

#### Equilabium pinetorum

Taxon classificationPlantaeLamialesLamiaceae

(A.J.Paton) Mwany. & A.J.Paton comb. nov.

124D4EC046BE586DB88E1E160CB65CA4

urn:lsid:ipni.org:names:77201361-1

Plectranthus pinetorum A.J.Paton, Fl. Zambes. 8(8): 224. 2013. Type: Malawi, Mulanje, Sombani basin, 15 Feb. 1992, Goyder & Paton 3660 (holotype: K).

##### Distribution.

Malawi and E. Zimbabwe.

#### Equilabium pulcherissimum

Taxon classificationPlantaeLamialesLamiaceae

(A.J.Paton) Mwany. & A.J.Paton comb. nov.

DB68459AD03655CFB1AAD5793BA44E92

urn:lsid:ipni.org:names:77201362-1

Plectranthus pulcherissimus A.J.Paton, Fl. Zambes. 8(8): 214. 2013. Type: Zambia, Mwinilunga Dist., Kalene Hill, 5 km N of bridge over Zambezi R. on Kalene Hill–Jimbe Bridge road, 2 Mar.1995, Harder, Zimba, Luwiika & Nawa 2854 (holotype: K; isotype: MO).

##### Distribution.

DRC and Zambia.

#### Equilabium pubescens

Taxon classificationPlantaeLamialesLamiaceae

(Baker) Mwany. & A.J.Paton comb. nov.

5149930812B15D4C9A2C6426F1F94458

urn:lsid:ipni.org:names:77201363-1

Plectranthus pubescens Baker in D.Oliver & auct. suc. (eds.), Fl. Trop. Afr. 5: 416. 1900. Type: Malawi, Mt Malosa, Nov. 1896, Whyte s.n. (holotype: K).
Plectranthus manganjensis Baker in D.Oliver & auct. suc. (eds.), Fl. Trop. Afr. 5: 406. 1900. Types: Malawi, Manganja Highlands, iv.1859, Kirk (syntype: K); Malawi, Mt Zomba, Dec. 1896, Whyte (syntype: K).
Plectranthus nyikensis Baker in D.Oliver & auct. suc. (eds.), Fl. Trop. Afr. 5: 416. 1900. Type: Malawi, Nyika Plateau, n.d., Whyte 162 (holotype: K).

##### Distribution.

SW. Tanzania to Mozambique.

#### Equilabium radiatum

Taxon classificationPlantaeLamialesLamiaceae

(A.J.Paton) Mwany. & A.J.Paton comb. nov.

81D643A89B0C53FCA0A031C2BCED61D9

urn:lsid:ipni.org:names:77201364-1

Plectranthus radiatus A.J.Paton, Fl. Trop. E. Afr., Lamiac.: 277. 2009. Type: Tanzania, Tunduru District: 13 km NW of Tunduru, Milne-Redhead & Taylor 7842 (holotype: K).

##### Distribution.

S. Tanzania.

#### Equilabium rungwense

Taxon classificationPlantaeLamialesLamiaceae

(A.J.Paton) Mwany. & A.J.Paton comb. nov.

7A75AC6C99DD59BF85FDCA71375FB5E9

urn:lsid:ipni.org:names:77201365-1

Plectranthus rungwensis A.J.Paton, Fl. Trop. E. Afr., Lamiac.: 252. 2009. Type: Tanzania, Rungwe District: NW slope of Mt Rungwe on N slope of Mwashitu Hill, above Sinihi stream, Mwasumbi 16222 (holotype: K; isotypes: DSM, MO).

##### Distribution.

Tanzania (Mt. Rungwe).

#### Equilabium scopulicola

Taxon classificationPlantaeLamialesLamiaceae

(A.J.Paton) Mwany. & A.J.Paton comb. nov.

4D8921D21E9157C5BF6F8A5E3C5F6FFC

urn:lsid:ipni.org:names:77201366-1

Plectranthus scopulicola A.J.Paton, Fl. Trop. E. Afr., Lamiac.: 259. 2009. Type: Tanzania, Lushoto District: World’s View, 1.5 km W of Gologolo, Drummond & Hemsley 2846 (holotype: K (K000431939); isotypes: BR, EA, K).

##### Distribution.

Tanzania (W. Usambara Mts.).

#### Equilabium selukwense

Taxon classificationPlantaeLamialesLamiaceae

(N.E.Br.) Mwany. & A.J.Paton comb. nov.

BDE4D3B48263581990487C15E9D17A92

urn:lsid:ipni.org:names:77201367-1

Plectranthus selukwensis N.E.Br., Bull. Misc. Inform. Kew 1906: 167. 1906. Type: Zimbabwe, Shurugwi (Selukwe), Nov. 1899, Cecil 123 (holotype: K).

##### Distribution.

Zambia to Zimbabwe.

#### Equilabium spananthum

Taxon classificationPlantaeLamialesLamiaceae

(A.J.Paton, Friis & Sebsebe) A.J.Paton comb. nov.

D4FEC0C2BCB25907AD1801521DD26F5E

urn:lsid:ipni.org:names:77201368-1

Plectranthus spananthus A.J.Paton, Friis & Sebsebe, Webbia 73(2): 216. 2018. Type: Ethiopia, Somali Regional State, Afder Zone, El Kere Woreda (in Bale floristic province of the Flora of Ethiopia and Eritrea), on road from Imi to El Kere, 10 km N of El Kere, I. Friis et al. 15539 (holotype: ETH; isotypes: C, K).

##### Distribution.

Ethiopia.

#### Equilabium stenophyllum

Taxon classificationPlantaeLamialesLamiaceae

(Baker) Mwany. & A.J.Paton comb. nov.

4552F6258D4E5A258D88423A09F10A08

urn:lsid:ipni.org:names:77201369-1

Fig. 7D

Plectranthus stenophyllus Baker in D.Oliver & auct. suc. (eds.), Fl. Trop. Afr. 5: 402. 1900. Type: Malawi, Shire Highlands, near Blantyre, 1887, Last s.n. (holotype: K).

##### Distribution.

S. Tanzania to S. Trop. Africa.

#### Equilabium stenosiphon

Taxon classificationPlantaeLamialesLamiaceae

(Baker) Mwany. & A.J.Paton comb. nov.

4B3F6768EE0A5B2BA73945E72B95889F

urn:lsid:ipni.org:names:77201370-1

Plectranthus stenosiphon Baker in D.Oliver & auct. suc. (eds.), Fl. Trop. Afr. 5: 415. 1900. Type: Malawi, R. Shire, 1863, Kirk s.n. (lectotype: K designated by Paton et al. (2013)).
Plectranthus albocaeruleus N.E.Br., Bull. Misc. Inform. Kew 1901: 130. 1901. Type: Malawi, Zomba, plant cultivated at Kew raised from seed sent June1898, Mahon s.n. (holotype: K).

##### Distribution.

S. Malawi to C. Mozambique and Zimbabwe.

#### Equilabium stoltzii

Taxon classificationPlantaeLamialesLamiaceae

(Gilli) Mwany. & A.J.Paton comb. nov.

CBEE084E29A45FCA839C5418F41650A2

urn:lsid:ipni.org:names:77201371-1

Plectranthus stolzii Gilli, Ann. Naturhist. Mus. Wien 77: 40. 1973. Type: Tanzania, Rungwe Dist., Kyimbila, 6 Oct. 1911, Stolz 914 (holotype: W; isotypes: B, C, G, K, LD, M, STU, Z).

##### Distribution.

SW. Tanzania to N. Malawi.

#### Equilabium subincisum

Taxon classificationPlantaeLamialesLamiaceae

(Benth.) Mwany., Smitha & A.J.Paton comb. nov.

5761B1226F4158AA8522D813BC0BA200

urn:lsid:ipni.org:names:77201372-1

Fig. 7C

Plectranthus subincisus Benth. in N.Wallich, Pl. Asiat. Rar. 2: 16. 1830. Type: India, Tamil Nadu: Courtallum, 1829, Wallich, Cat. No. 2737 (holotype: K (K000820179); isotype: K-W (K001116985)).

##### Distribution.

S. India, Sri Lanka. Occurrence in Sri Lanka is questionable and it is likely to be extinct there (Cramer 1981).

#### Equilabium vesiculare

Taxon classificationPlantaeLamialesLamiaceae

(A.J.Paton) Mwany. & A.J.Paton comb. nov.

6ED98D9990CB5794A9D290B562F1B904

urn:lsid:ipni.org:names:77201373-1

Plectranthus vesicularis A.J.Paton, Fl. Trop. E. Afr., Lamiac.: 272. 2009. Type: Tanzania, Kilwa Dist., Kilunda, 6.5 km (4 mile), 24 Apr. 1971, Ludanga in MRC 1301 (holotype: K; isotype: EA).

##### Distribution.

Tanzania to N. Mozambique.

#### Equilabium viphyense

Taxon classificationPlantaeLamialesLamiaceae

(Brummitt & Seyani) Mwany., Culham & A.J.Paton comb. nov.

A1C1B94C1D9E5FE6AC0503741AC320A6

urn:lsid:ipni.org:names:77201374-1

Plectranthus viphyensis Brummitt & Seyani, Kew Bull. 42: 692. 1987. Type: Malawi, Mzimba Dist., Viphya, 11 Jan. 1970, Pawek 3338 (holotype: K).

##### Distribution.

Tanzania to S. Trop. Africa.

#### Equilabium viphyense subsp. viphyense

Taxon classificationPlantaeLamialesLamiaceae

227AF21629CF55F985D7676706B08504

##### Distribution.

Tanzania to S. Trop. Africa.

#### Equilabium viphyense subsp. zebrarum

Taxon classificationPlantaeLamialesLamiaceae

 (Brummitt & Seyani) Mwany. & A.J.Paton comb. nov.

4685FE2AB0BC5BF38F445A419C331D6B

urn:lsid:ipni.org:names:77201375-1

Plectranthus zebrarum Brummitt & Seyani, Kew Bull. 42: 694. 1987.
Plectranthus viphyensis subsp. zebrarum (Brummitt & Seyani) A.J.Paton, Fl. Trop. E. Afr., Lamiac.: 276. 2009. Type: Malawi, Nyika Plateau, Chelinda, below No.1 dam, 27 Feb.1982, Brummitt, Polhill & Banda 16140 (holotype: K).

##### Distribution.

E. Zambia to N. Malawi.

#### Equilabium wollastonii

Taxon classificationPlantaeLamialesLamiaceae

(S.Moore) Mwany. & A.J. Paton comb. nov.

5B90F2FD180351A4ACD87516501BCE91

urn:lsid:ipni.org:names:77201378-1

Plectranthus wollastonii S.Moore, J. Linn. Soc., Bot. 38: 272. 1908. Type: Uganda, E Ruwenzori, Wollaston s.n. (holotype: BM).

##### Distribution.

EC. Trop. Africa to S. Kenya.

### Genus *Plectranthus*

#### Plectranthus

Taxon classificationPlantaeLamialesLamiaceae

L’Hér., Stirp. Nov.: 84, verso 1788 nom. cons.

DC50FA9D169359FE96C017901EA3915C

Figs 8 , 9

Germanea Lam., Encycl. 2: 690. 1788. Lectotype species: G. urticifolia Lam. (here placed under Plectranthus fruticosus L’Hér.) designated by Heath (2001).

##### Lectotype species.

*Plectranthus fruticosus* L’ Hér. (typ. cons.).

##### Description.

Perennial, rarely annual herbs or soft-wooded shrubs, sometimes succulent; sometimes with a tuberous base. Leaves opposite. Inflorescence thrysoid, usually lax; cymes sessile, 1–3-flowered; bracts subtending cymes persistent. Flowers pedicelate. Calyx funnel-shaped straight, with pedicel attaching symmetrically at calyx base, not opposite posterior lip, two-lipped; throat glabrous within, open; posterior lip 1-lobed, broader than lobes of anterior lip, shortly decurrent on tube or not; anterior lip 4-lobed; lobes lanceolate; lateral lobes broader at base than medial lobes and held closer to the median lobes than the posterior lip; median lobes usually fused for a greater distance than the lateral and median lobe. Corolla two-lipped with lips equal in length; tube straight or gently curved downwards, often gibbous, saccate or almost spurred a base, more rarely dilating gradually towards throat; posterior lip 4-lobed erect, often becoming reflexed; anterior lip cucullate or concave straight or becoming reflexed. Stamens 4, very rarely only 2 fertile (*P. zuluensis*); filaments not fused together (anterior stamen and adjacent posterior stamen sometimes fused in *P. alboviolaceus*), held within the anterior lip or becoming free from it and sometimes coiling downwards away from style after anthesis. Style bifid with lobes subulate. Nutlets ovoid.

**Figure 8.**
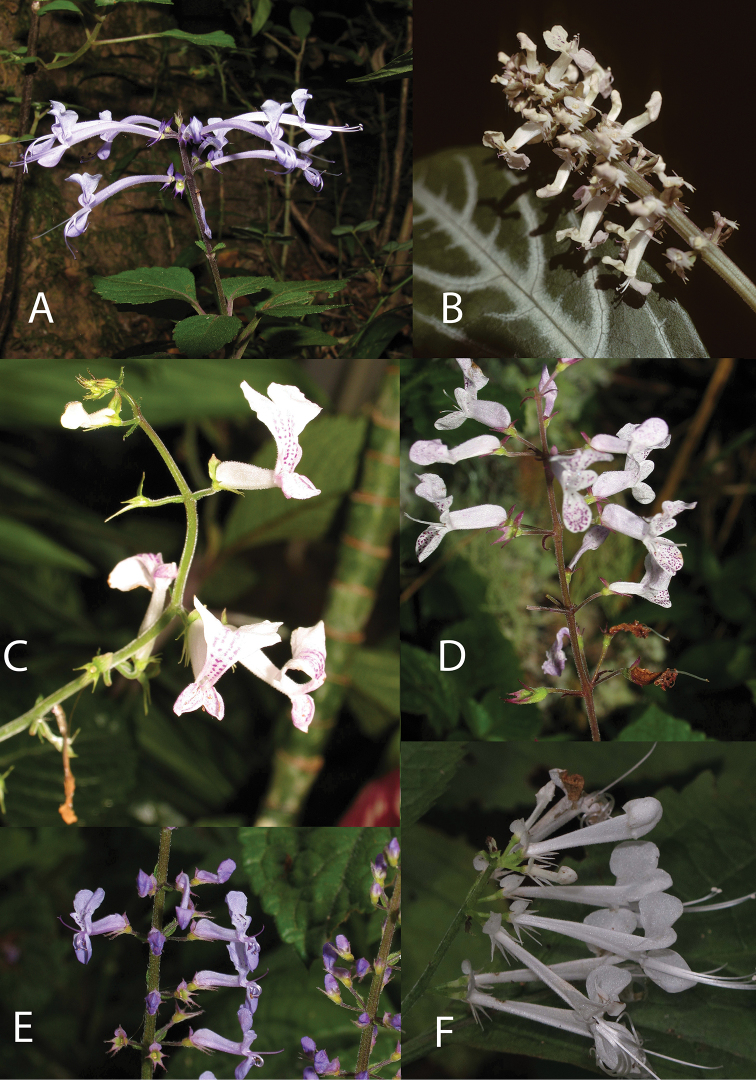
Species of *Plectranthus* 1. **A***Plectranthus ambiguus* (Photo: Neil Crouch) **B***P. brevicaulis* (Photo: Jean Bosser) **C***P. brevimentus* (Photo: Neil Crouch) **D**P. ciliatus (Photo: Neil Crouch) **E***P. fruticosus* (Photo: Neil Crouch) **F***P. ecklonii* (Photo: Neil Crouch).

**Figure 9.**
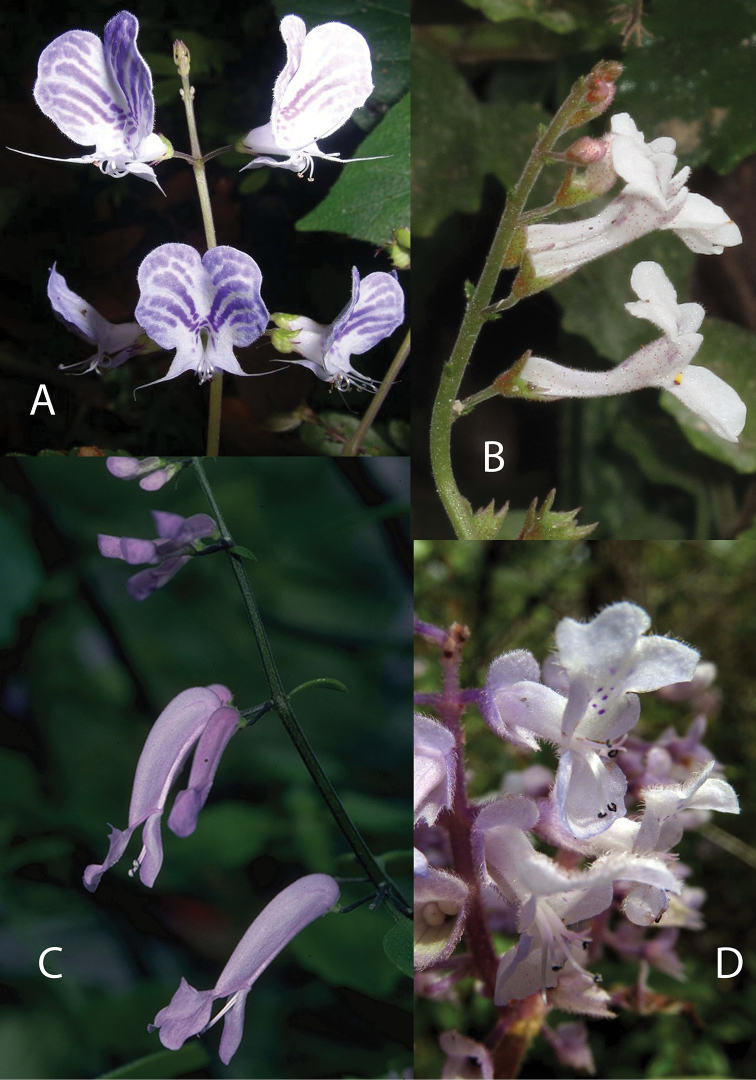
Species of *Plectranthus* 2. **A***Plectranthus papilionaceus* (Photo: Patrick Ranirison) **B**P. purpuratus subsp. montanus (Photo: Neil Crouch) **C**P. saccatus var. longitubus (Photo RBG Kew) **D***P. vestitus* (Photo: Patrice Antilahimena).

72 species in Southern and Tropical Africa and Madagascar and including one in Sri Lanka.

#### Plectranthus alboviolaceus

Taxon classificationPlantaeLamialesLamiaceae

Gürke, Bot. Jahrb. Syst. 30: 397. 1901

90DF24CCA8025E028E74FB311E9A5233

Plectranthus alboviolaceus Gürke, Bot. Jahrb. Syst. 30: 397. 1901. Type: Tanzania, Rungwe District: Kalengalenga Mts, Goetze 1140 (isotype: E).
Plectranthus rhomboideus Gürke, Bot. Jahrb. Syst. 36: 135. 1905. Type: Tanzania, Kilimanjaro, Engler 1775 (holotype: B, destroyed).

##### Distribution.

Ethiopia to S. Trop. Africa.

#### Plectranthus ambiguus

Taxon classificationPlantaeLamialesLamiaceae

(Bolus) Codd, Bothalia 8: 159. 1964

C79E57EDEC3A5512890A8D5CCD9F0C4D

Fig. 8A

Orthosiphon ambiguus Bolus, J. Linn. Soc., Bot. 18: 394. 1881. Type: South Africa, Eastern Cape, near Grahamstown, MacOwen (holotype: BOL; isotype: SAM).
Plectranthus coloratus E.Mey., Comm. Pl. Afr. Austr.: 228. 1838., nom. illeg., non. P. coloratus D.Don.
Plectranthus dregei Codd, Fl. Pl. Africa 32: t. 1244. 1957. Type: South Africa, between Umgazana and Umzimvubu rivers, 19 May 1832, Drège a (syntypes: BM, HBG, K (K000430874), MO, P(P00450719, P00450717), S); between Umizimkulu and Umkomass Rivers, 1 May 1832, Drège b (syntype: P(00450718).

##### Distribution.

South Africa: E. Cape Prov. to N. KwaZulu-Natal.

#### Plectranthus amplexicaulis

Taxon classificationPlantaeLamialesLamiaceae

Hedge, Fl. Madag. 175: 203. 1998

8F409B028D2451E6A82881F73F0F6416

Plectranthus amplexicaulis Hedge, Fl. Madag. 175: 203. 1998. Type: Madagascar, Andrambovato, à l’Est de Fianarantsoa, H. Humbert & R.P.R. Capuron 28517 (holotype: P).

##### Distribution.

Madagascar.

#### Plectranthus antongilicus

Taxon classificationPlantaeLamialesLamiaceae

Hedge, Fl. Madag. 175: 160. 1998

D721D77392865430A269FEE22F4E9998

Plectranthus antongilicus Hedge, Fl. Madag. 175: 160. 1998. Type: Madagascar, around Rantabe, baie d’Antongil, Perrier de la Bâthie 10475 (holotype: P; isotypes: MO, TAN).

##### Distribution.

Madagascar.

#### Plectranthus asymmetricus

Taxon classificationPlantaeLamialesLamiaceae

A.J.Paton, Fl. Zambes. 8(8): 207. 2013

E6ED26F06E495D3B84C2A711F06EB6B5

Plectranthus asymmetricus A.J.Paton,, Fl. Zambes. 8(8): 207. 2013. Type: Zambia, Mbala Dist., Chinakila, Jembele Forest, 13 Jan.1965, Richards 19510 (holotype: K).

##### Distribution.

Zambia.

#### Plectranthus atroviolaceus

Taxon classificationPlantaeLamialesLamiaceae

Hedge, Fl. Madag. 175: 156. 1998

B0C0907ED63E56DCB9CB22901E2AC4B1

Plectranthus atroviolaceus Hedge, Fl. Madag. 175: 156. 1998. Type: Madagascar, Haute vallée de la Rienana (bassin du Matitanana). 2e voyage. Humbert 3464 (holotype: P; isotype: E).

##### Distribution.

Madagascar.

#### Plectranthus betamponus

Taxon classificationPlantaeLamialesLamiaceae

Hedge, Fl. Madag. 175: 175. 1998

3A46E4C489EA50F4BE8A1EC3F61C4AFB

Plectranthus betamponus Hedge, Fl. Madag. 175: 175. 1998. Type: Madagascar, Reserve Integrale #1 Betampona, approximately 3 km N of Fotsimavo, Schatz, Goldblatt, Rakotozafy & Randrianasolo 2682 (holotype: MO; isotype: P).

##### Distribution.

Madagascar.

#### Plectranthus bracteolatus

Taxon classificationPlantaeLamialesLamiaceae

A.J.Paton, Fl. Trop. E. Afr., Lamiac.: 245. 2009

55890083BE2851839E9F6E69986CE951

Plectranthus bracteolatus A.J.Paton, Fl. Trop. E. Afr., Lamiac.: 245. 2009. Type: Tanzania, Morogoro District: Uluguru Mts, Schlieben 4215 (holotype: K; isotypes: K, MO, PRE, SRGH).

##### Distribution.

Tanzania.

##### Notes.

More work is needed to be confident about the generic placement of this species. A preliminary investigation suggests a possible relationship to *Isodon* or a clade basal to the Ocimeae and Plectranthinae.

#### Plectranthus brevicaulis

Taxon classificationPlantaeLamialesLamiaceae

(Baker) Hedge, Fl. Madag. 175: 154. 1998

B2A6C9D41B9D5941B4E516371EDAE9BF

Fig. 8B

Orthosiphon brevicaulis Baker, J. Linn. Soc., Bot. 21: 433. 1885. Type: Madagascar, central, Baron 2656 (holotype: K; isotype: P).

##### Distribution.

Madagascar.

#### Plectranthus brevimentus

Taxon classificationPlantaeLamialesLamiaceae

T.J.Edwards, Bothalia 35: 149. 2005

AD608C3D5CB45BDB95CF310B0CB47359

Fig. 8C

Plectranthus brevimentus T.J.Edwards, Bothalia 35: 149. 2005. Type: South Africa, Eastern Cape; Lupatana River Gorge; ca 1km from sea, T.J.Edwards, D.G.A. Styles, N.Crouch, N.D.U.Bellstedt, C.J.Potgieter 3210 (holotype: NU).

##### Distribution.

South Africa: Eastern Cape Prov.

#### Plectranthus canescens

Taxon classificationPlantaeLamialesLamiaceae

Benth ., Labiat. Gen. Spec.: 33. 1832

EE1E951BCA7757FCBE9E31623E004102

Plectranthus canescens Benth., Labiat. Gen. Spec.: 33. 1832. Type: Madagascar, Bojer s.n. (lectotype: K, designated by Hedge et al. (1998); isolectotype: P).

##### Distribution.

Madagascar.

#### Plectranthus capuronii

Taxon classificationPlantaeLamialesLamiaceae

Hedge, Fl. Madag. 175: 164. 1998

3BE845847FB3515D8EF6E7E0803EE699

Plectranthus capuronii Hedge, Fl. Madag. 175: 164. 1998. Type: Madagascar, montagnes au nord de Mangindrano jusqu’aux sommets d’Ambohimirahavavy, Humbert & Capuron 25183 (holotype: P; isotypes: B, BR, K, MA, MO, NY, TAN, WAG).

##### Distribution.

Madagascar.

#### Plectranthus chimanimanensis

Taxon classificationPlantaeLamialesLamiaceae

S.Moore, J. Linn. Soc., Bot. 40: 174. 1911

F36FF1DB2C7F5F668D6D1027DEB39E0A

Plectranthus chimanimanensis S.Moore, J. Linn. Soc., Bot. 40: 174. 1911. Type: Mozambique, Gazaland, 26 Sept. 1906, Swynnerton 2019 (holotype: BM; isotype: K).

##### Distribution.

E. Zimbabwe to W. Mozambique.

#### Plectranthus ciliatus

Taxon classificationPlantaeLamialesLamiaceae

E.Mey., Comm. Pl. Afr. Austr.: 227. 1838

A95ECB15D0715D92B45150C066D485AB

Fig. 8D

Plectranthus ciliatus E.Mey., Comm. Pl. Afr. Austr.: 227. 1838. Type: South Africa, Natal, Omsamvubo [Umzimvubu] Drège 4777 (lectotype: K (K000430860), designated by Codd (1975)).
Plectranthus natalensis Gürke, Bull. Herb. Boissier 6: 552. 1898. Type: South Africa, Natal, Camperdown, Rehmann 7701 (holotype: Z).

##### Distribution.

South Africa and Swaziland.

#### Plectranthus clementiae

Taxon classificationPlantaeLamialesLamiaceae

Hedge, Fl. Madag. 175: 168. 1998

4220B892D6985DE888173A13BBB27E6D

Plectranthus clementiae Hedge, Fl. Madag. 175: 168. 1998. Type: Madagascar:, prov. de Tuléar, près de la réserve intégrale d’Andohahela, parcelle 1, 33km sur la route vers Ranomafana, à partir de la RN 13, Clement, Phillipson & Rafamantanantsoa 2097 (holotype: E; isotypes: MO, P).

##### Distribution.

Madagascar.

#### Plectranthus cordatus

Taxon classificationPlantaeLamialesLamiaceae

A.J.Paton & Phillipson nom. nov.

7CE40C2AD9C85E3FB8B9E33CD71484A9

urn:lsid:ipni.org:names:77201380-1

Plectranthus cardiophyllus Hedge, Fl. Madagasc. 175: 172. 1998, nom. illeg., non. P. cardiophyllus Hemsl. Type: Madagascar, Mt Vatovavy, bassin du Mananjary, Perrier de la Bâthie 10441 (holotype: P).

##### Distribution.

Madagascar.

#### Plectranthus decaryi

Taxon classificationPlantaeLamialesLamiaceae

Hedge, Fl. Madag. 175: 182. 1998

C2D592DC3E6C5CC5B8C3B8D9F55DB80C

Plectranthus decaryi Hedge, Fl. Madag. 175: 182. 1998. Type: Madagascar, Mt Maromizaha, près d’Analamazaotra, Perrier de la Bâthie 16027 (holotype: P).

##### Distribution.

Madagascar.

#### Plectranthus delicatissimus

Taxon classificationPlantaeLamialesLamiaceae

Hedge, Fl. Madag. 175: 197. 1998

59FF5F48B0125E8FB6A4E83D201F5C2B

Plectranthus delicatissimus Hedge, Fl. Madag. 175: 197. 1998. Type: Madagascar, prov. d’Antsiranana, réserve spéciale 5, Manongarivo, sud-est d’Ankaramy et à 1–3 km au sud d’Ambalafary, Schatz 3204 (holotype: MO; isotype: E).

##### Distribution.

Madagascar.

#### Plectranthus ecklonii

Taxon classificationPlantaeLamialesLamiaceae

Benth. in A.P.de Candolle, Prodr. 12: 64. 1848

B7C5AE235E845828A850ACDC40FF6EFE

Fig. 8F

Plectranthus ecklonii Benth. in A.P.de Candolle, Prodr. 12: 64. 1848. Type: South Africa, Cape, slopes of Katberg, Ecklon s.n. (holotype: K).

##### Distribution.

South Africa.

#### Plectranthus elegans

Taxon classificationPlantaeLamialesLamiaceae

Britten, Trans. Linn. Soc. London, Bot. 4: 36. 1894

DC7F2DF9BE1056B09DF0808466F4CA1A

Plectranthus elegans Britten, Trans. Linn. Soc. London, Bot. 4: 36. 1894. Type: Malawi, Mulanje, 1891, Whyte s.n. (holotype: BM).
Plectranthus mahonii (Baker) N.E.Br. ex Hook.f., Bot. Mag. 128: t. 7818. 1902.
Coleus mahonii Baker in D.Oliver & auct. suc. (eds.), Fl. Trop. Afr. 5: 434. 1900. Type: Cultivated plant, raised from seed sent from Malawi by Mahon 19 Dec.1899 (holotype: K).

##### Distribution.

S. Malawi.

#### Plectranthus elegantulus

Taxon classificationPlantaeLamialesLamiaceae

Briq., Bull. Herb. Boissier, sér. 2, 3: 1005 1903

3E00E09BF993518AABCB79D2D10BF06D

Germanea elegantula Briq., Bull. Herb. Boissier, sér. 2, 3: 1005. 1903. Type: South Africa, Natal, Karkloof, Rehmann 7368 (holotype: Z).

##### Distribution.

South Africa: KwaZulu-Natal.

#### Plectranthus ellipticus

Taxon classificationPlantaeLamialesLamiaceae

Hedge, Fl. Madag. 175: 186. 1998

8004E0874F9C533BB7C95CA3F173AF93

Plectranthus ellipticus Hedge, Fl. Madag. 175: 186. 1998. Type: Madagascar, haute vallée de la Rienana, Hubert 3638 (holotype: P; isotype: E).

##### Distribution.

Madagascar.

#### Plectranthus emirnensis

Taxon classificationPlantaeLamialesLamiaceae

(Baker) Hedge, Fl. Madag. 175: 187. 1998

6E28066F49F9567B80A126E88FF5AB67

Orthosiphon emirnensis Baker, J. Linn. Soc., Bot. 21: 433. 1885. Type: Madagascar, centre, Baron 1056 (lectotype: K, designated by Hedge et al. (1998)).
Orthosiphon hildebrandtii Vatke, Abh. Naturwiss. Vereins Bremen 9: 134. 1885. nom. superfl. based on Baron 1506.
Ocimum hildebrandtii (Vatke) Briq. in H.G.A.Engler & K.A.E.Prantl, Nat. Pflanzenfam. 4(3a): 372. 1897., nom. illeg.
Ocimum siphonanthum Briq., Bull. Herb. Boissier 2: 121. 1894. Type: Madagascar, Betsileo, near Ankafina, Hildebrandt 3947 (holotype: G, not seen; isotype: P).

##### Distribution.

Madagascar.

#### Plectranthus ernstii

Taxon classificationPlantaeLamialesLamiaceae

Codd, Fl. Pl. Africa 47: t. 1855. 1982

4C6C98C3C449549786A168637E9471FB

Plectranthus ernstii Codd, Fl. Pl. Africa 47: t. 1855. 1982. Type: South Africa, Kwa Zulu Natal, Oribi Gorge, Fairacres Farm, van Jaarsveld 2196; Cultivated in Pretoria sub Garden 24529 in PRE 57984 (holotype: PRE; isotype: NBG).

##### Distribution.

South Africa: E. Cape Prov. to S. KwaZulu-Natal.

#### Plectranthus forsythii

Taxon classificationPlantaeLamialesLamiaceae

Hedge, Fl. Madag. 175: 177. 1998

E92677DABD305E89862BB9F6D00FD4A0

Plectranthus forsythii Hedge, Fl. Madag. 175: 177. 1998. Type: Madagascar, entre Itandraka et Ambohimitombo, Forsyth Major 727 (holotype: K, isotype: BM).

##### Distribution.

Madagascar.

#### Plectranthus fruticosus

Taxon classificationPlantaeLamialesLamiaceae

L’Hér., Stirp. Nov.: 85. 1788

5CE226FDF1E65551809D8EAF6E4E7037

Fig. 8E

Plectranthus fruticosus L’Hér., Stirp. Nov.: 85. 1788. Type: cultivated, illustration t.41 of L’Hér. Stirp. Nov. fasc. 4. 1788 (lectotype: Illustration, designated by Codd (1975)).
Germanea urticifolia Lam., Encycl. 2: 691. 1788.
Plectranthus urticifolius (Lam.) Salisb., Prodr. Stirp. Chap. Allerton: 88. 1796. Type: Cultivated, illustration, Tabl. Encycl. 3: 514. 1819 (lectotype: Illustration, designated by Codd (1975)).
Plectranthus galpinii Schltr., J. Bot. 1896: 393. 1896. Type: South Africa, Transvaal, Barberton, Rimers Creek, Galpin 939 (holotype: B, destroyed; isotype: PRE).
Plectranthus arthropodus Briq., Bull. Herb. Boissier, sér. 2, 3: 1073. 1903. Type: South Africa, Transvaal, Houtbosch, Rehman 6151 (holotype: Z).
Plectranthus charianthus Briq., Bull. Herb. Boissier, sér. 2, 6: 824. 1906. Type: South Africa, Transvaal, Houtbosch, Rehman 6157 (holotype: Z).
Plectranthus peglerae T.Cooke, Bull. Misc. Inform. Kew 1909: 378. 1909. Type: South Africa, Eastern Cape, Kentani, Pegler 377 (holotype: K; isotypes: GRA, PRE).
Plectranthus behrii Compton, J. S. African Bot. 11: 122. 1945. Type: South Africa, Pondoland, Lusikisiki, Behr, sub NBG 1252/31 (holotype: SAM; isotype: PRE).

##### Distribution.

S. Mozambique to South Africa.

#### Plectranthus gardneri

Taxon classificationPlantaeLamialesLamiaceae

Thwaites, Enum. Pl. Zeyl.: 237. 1860

8744230AA3735097BFBDC2020EB999D7

Plectranthus subincisus Benth. var. gardneri (Thwaites) Hook. f. in Flora of British India 4: 622. 1885. Type: Sri Lanka, Newera Ellis, Gardner in Thwaites CP16 (holotype: PDA; isotype: K).

##### Distribution.

Sri Lanka.

#### Plectranthus gibbosus

Taxon classificationPlantaeLamialesLamiaceae

Hedge, Fl. Madag. 175: 200. 1998

3BDA0BCB85765915B8C02D02DC893E67

Plectranthus gibbosus Hedge, Fl. Madag. 175: 200. 1998. Type: Madagascar, Fenerive, Decary 3922 (holotype: P(P00541407); isotype: P(P00541408)).

##### Distribution.

Madagascar.

#### Plectranthus grallatus

Taxon classificationPlantaeLamialesLamiaceae

Briq., Bull. Herb. Boissier, sér. 2, 3: 1004. 1903

E077B642F1B7507088B3D12513BB6944

Germanea grallata Briq. *loc. cit* as alternative name). Type: South Africa, Natal, Mt Frere, Jan. 1895, Schlechter 6415 (holotype: Z; isotype: GRA).
Plectranthus transvaalensis Briq., Bull. Herb. Boissier, sér. 2, 3: 1005. 1903.
Germanea transvaalensis Briq., *loc. cit* as alternative name. Type: South Africa, Transvaal, Houtbosch, Rehmann 6154 (holotype: Z).
Plectranthus krookii Gürke, Ann. K. K. Naturhist. Hofmus. 20: 48. 1905. Type: South Africa, Griqualand East, R. Umzinklawa, Krook in Herb Penther 1698 (holotype: W).
Plectranthus praetervisus Briq., Bull. Herb. Boissier, sér. 2, 6: 825. 1906. Type: South Africa, Natal, Drakensberg, Mountain Prospect, Rehmann 6965 (holotype: Z).
Plectranthus cooperi T.Cooke, Bull. Misc. Inform. Kew 1909: 377. 1909. Type: South Africa: Orange River Colony, Cooper, 2982 (lectotype: K, designated here).

##### Distribution.

South Africa, Lesotho.

#### Plectranthus grandibracteatus

Taxon classificationPlantaeLamialesLamiaceae

Hedge, Fl. Madag. 175: 152. 1998

60359C3234275274BBF18113A3C6BF7B

Plectranthus grandibracteatus Hedge, Fl. Madag. 175: 152. 1998. Type: Madagascar, vallée de l’Androranga, affluent de la Bemarivo aux env. Antongondriha, mont Anjenabe, Mubolt & Cuperon 24144 (holotype: P).

##### Distribution.

Madagascar.

#### Plectranthus guruensis

Taxon classificationPlantaeLamialesLamiaceae

A.J.Paton, Fl. Zambes. 8(8): 205. 2013

D52BB1DCBF75510D934BC29346BEA99A

Plectranthus guruensis A.J.Paton, Fl. Zambes. 8(8): 205. 2013. Type: Mozambique, Gurué Dist., Rio Malema, Marope, 22 km from Gurué, 1 Aug. 1979, de Koning 7530 (holotype: LMU; isotype: LISC).

##### Distribution.

Mozambique.

#### Plectranthus hexaphyllus

Taxon classificationPlantaeLamialesLamiaceae

Baker, J. Linn. Soc., Bot. 20: 231. 1883

4A80CFB1121D5D90A98B27EDE05F2AEA

Plectranthus hexaphyllus Baker, J. Linn. Soc., Bot. 20: 231. 1883. Type: Madagascar, central, Baron 1799 (holotype: K; isotype: P).

##### Distribution.

Madagascar.

#### Plectranthus hilliardiae

Taxon classificationPlantaeLamialesLamiaceae

Codd, Bothalia 11: 282. 1974

FCDC7BB161E959E28AA62D083E9E8491

Plectranthus hilliardiae Codd, Bothalia 11: 282. 1974. Type: South Africa, Natal, near Umtamvuna River, Hilliard & Burtt 6767 (holotype: PRE; isotype: NU).

##### Distribution.

South Africa: E. Cape Prov. to KwaZulu-Natal.

#### Plectranthus hilliardiae subsp. hilliardiae

Taxon classificationPlantaeLamialesLamiaceae

.

B7387B2005985832A93956A282EB625A

##### Distribution.

South Africa: KwaZulu-Natal.

#### Plectranthus hilliardiae subsp. australis

Taxon classificationPlantaeLamialesLamiaceae

 van Jaarsv. & A.E.van Wyk, Bothalia 31: 44. 2001

AD31483CE57F57958CD5D0F86B542CE2

Plectranthus hilliardiae subsp. australis van Jaarsv. & A.E.van Wyk, Bothalia 31: 44. 2001. Type: South Africa, Eastern cape, Mbotyi forest, van Jarsveld, Bellsredt, Dekker, van Wyk & Adams 16345 (holotype: NBG).

##### Distribution.

South Africa: E. Cape Prov.

#### Plectranthus hirsutus

Taxon classificationPlantaeLamialesLamiaceae

Hedge, Fl. Madag. 175: 158. 1998

781F5654845A5D76A5DAF6ED216B0B17

Plectranthus hirsutus Hedge, Fl. Madag. 175: 158. 1998. Type: Madagascar, Mont Kalabenono, Perrier de la Bâthie 10397 (holotype: P).

##### Distribution.

Madagascar.

#### Plectranthus hoslundioides

Taxon classificationPlantaeLamialesLamiaceae

Scott-Elliot, J. Linn. Soc., Bot. 29: 44. 1891

A3DDFF22D3C552D9B3AF53067EEA4BB6

Plectranthus hoslundioides Scott-Elliot, J. Linn. Soc., Bot. 29: 44. 1891. Type: Madagascar, near Fort Dauphin, Scott- Elliot 2645 (holotype: K; isotype: P).

##### Distribution.

Madagascar.

#### Plectranthus humbertii

Taxon classificationPlantaeLamialesLamiaceae

Hedge, Fl. Madag. 175: 194. 1998

5A5E859D6BD35FB2BC5D0643A135C849

Plectranthus humbertii Hedge, Fl. Madag. 175: 194. 1998. Type: Madagascar, partie occidentale du massif du Marojejy, de la vallée de l’Ambatoharanana au bassin supérieur de l’Antsahaberoka, Humbert & Saboureau 31311 (holotype: P; isotype: P).

##### Distribution.

Madagascar.

#### Plectranthus incrassatus

Taxon classificationPlantaeLamialesLamiaceae

Hedge, Edinburgh J. Bot. 60: 246. 2003

363A992C8C1D5DD399B648456C76E33A

Plectranthus incrassatus Hedge, Edinburgh J. Bot. 60: 246. 2003.Type: Madagascar: massif de l’Anjanaharibe, à l’ouest d’Andapa, basin de la Lokoho, Humbert, Capuron & Cours 24774 (holotype: P; isotype: K).
Plectranthus pachyphyllus Hedge, Fl. Madag. 175: 176. 1998., nom. illeg., non. P. pachyphyllus Gürke.

##### Distribution.

Madagascar.

#### Plectranthus laurifolius

Taxon classificationPlantaeLamialesLamiaceae

Hedge, Fl. Madag. 175: 170. 1998

9F24D977866D51F986D9CABA663F8278

Plectranthus laurifolius Hedge, Fl. Madag. 175: 170. 1998. Type: Madagascar, vallée de la Lokoho, mont Ambodilaitra, au nord d’Andranomiforitra, Humbert 23277 (holotype: P; isotype: MO).

##### Distribution.

Madagascar.

#### Plectranthus linearis

Taxon classificationPlantaeLamialesLamiaceae

Hedge, Fl. Madag. 175: 191. 1998

690F09F9994C53869C28B1F5FAA28396

Plectranthus linearis Hedge, Fl. Madag. 175: 191. 1998. Type: Madagascar, pentes occidentales du massif du Marojejy, bassin de la Lokoho, à l’est d’Ambalamanasy, Humbert & Capuron 22069 (holotype: P; isotypes: MO, P).

##### Distribution.

Madagascar.

#### Plectranthus longiflorus

Taxon classificationPlantaeLamialesLamiaceae

Benth., Labiat. Gen. Spec.: 33. 1832

13B72BA4DB2B5CF5938E07926F38108E

Plectranthus longiflorus Benth., Labiat. Gen. Spec.: 33. 1832.Type: Madagascar, forêt de Beforona, Bojer s.n. (holotype: K; isotype: P).

##### Distribution.

Madagascar.

#### Plectranthus longipetiolatus

Taxon classificationPlantaeLamialesLamiaceae

Hedge, Fl. Madag. 175: 147. 1998

68D41BCE5EF257EEA1A0C8DA1824BAE5

Plectranthus longipetiolatus Hedge, Fl. Madag. 175: 147. 1998. Type: Madagascar, massif de Beampingaratra, du col de Bevava au sommet de Bekoho, Humbert 6432 (holotype: P; isotypes: B, E, K, MO, WAG).

##### Distribution.

Madagascar.

#### Plectranthus lucidus

Taxon classificationPlantaeLamialesLamiaceae

(Benth.) van Jaarsv. & T.J.Edwards, Bothalia 27: 5. 1997

B4F06025D2C35A8B86DA0294BE2A84E8

Plectranthus strigosus var. lucidus Benth. in A.P.de Candolle, Prodr. 12: 68. 1848. Type: Eastern Cape, Bathurst, Burchell 3924, (holotype: K).

##### Distribution.

South Africa: S. Cape Prov.

#### Plectranthus macilentus

Taxon classificationPlantaeLamialesLamiaceae

Hedge, Fl. Madag. 175: 170. 1998

A7E828C4CB2B5A1EB6422FFD06EAA741

Plectranthus macilentus Hedge, Fl. Madag. 175: 170. 1998. Type: Madagascar, collines et plateaux de l’Ankarana du Nord, (Province de Diégo-Suarez), W de la grande route Mt Ambatopiraka, Antsiranana Humbert & Cours 32655 (holotype: P).

##### Distribution.

Madagascar.

#### Plectranthus malvinus

Taxon classificationPlantaeLamialesLamiaceae

van Jaarsv. & T.J.Edwards, Bothalia 27: 1. 1997

74123B51B33351BFA0E4CD49D1CC22D7

Plectranthus malvinus van Jaarsv. & T.J.Edwards, Bothalia 27: 1. 1997. Type: Eastern Cape, 3129 (Port St Johns), Mount Sullivan, (-DA), E. van Jaarsveld & Bingham 10522 (holotype: NBG).

##### Distribution.

South Africa: E. Cape Prov.

#### Plectranthus mandalensis

Taxon classificationPlantaeLamialesLamiaceae

Baker in D.Oliver & auct. suc. (eds.), Fl. Trop. Afr. 5: 405. 1900

74F4AA4B99BC56C5A773408614F102A0

Plectranthus mandalensis Baker in D.Oliver & auct. suc. (eds.), Fl. Trop. Afr. 5: 405. 1900. Type: Malawi, Mandala, cultivated at Kew, Feb. 1893, Moir s.n. (holotype: K).

##### Distribution.

Malawi (Mt. Mulanje), Mozambique (Mt. Namuli).

#### Plectranthus membranaceus

Taxon classificationPlantaeLamialesLamiaceae

(Scott-Elliot) Hedge, Fl. Madag. 175: 145. 1998

2434E96D519056F7AA1FB8FB7F75C5DD

Plectranthus canescens var. membranaceus Scott-Elliot, J. Linn. Soc., Bot. 29: 49. 1891. Type: Madagascar, between Angalampena and the sea, Scott-Elliot 3072 (holotype: K; isotype: P).

##### Distribution.

Madagascar.

#### Plectranthus mocquerysii

Taxon classificationPlantaeLamialesLamiaceae

Briq., Annuaire Conserv. Jard. Bot. Genève 2: 233. 1898

B639645114B659CDB66AEA6D9DC5FD38

Plectranthus mocquerysii Briq., Annuaire Conserv. Jard. Bot. Genève 2: 233. 1898. Type: Madagascar, Maroa, forest inland from bay d’ Antongil, Mocquerys 239 (holotype: G).

##### Distribution.

Madagascar.

#### Plectranthus mzimvubuensis

Taxon classificationPlantaeLamialesLamiaceae

van Jaarsv., Bothalia 34: 30. 2004

6344B3F5B6CE5FA6925EBCEB62E4D91B

Plectranthus mzimvubuensis van Jaarsv., Bothalia 34: 30. 2004. Type: South Africa, Eastern Cape, near Ludonga village, Mzimvubu River, Ecca group shale cliffs, Van Jaarsveld, Xaba, Harower & Zwide 92 (holotype: PRE).

##### Distribution.

South Africa: SE. Cape Prov.

#### Plectranthus oblanceolatus

Taxon classificationPlantaeLamialesLamiaceae

Hedge, Fl. Madag. 175: 161. 1998

C66448B8D32E5D4EB41F776C94A3C529

Plectranthus oblanceolatus Hedge, Fl. Madag. 175: 161. 1998. Type: Madagascar, partie occidentale du Massif du Marojejy, de la vallée de l’Ambatoharanana au bassin supérieur de l’Antsahaberoka, Humbert & Saboureau 31614 (holotype: P; isotype: P).

##### Distribution.

Madagascar.

#### Plectranthus oertendahlii

Taxon classificationPlantaeLamialesLamiaceae

T.C.E.Fr., Acta Horti Gothob. 1: 253. 1924

77F5939DC9125CAC8D27BEA28824A30C

Plectranthus oertendahlii T.C.E.Fr., Acta Horti Gothob. 1: 253. 1924. Type: cultivated from material collected near Port Shepstone, Uppsala, (holotype, UPS).

##### Distribution.

South Africa: S. KwaZulu-Natal.

#### Plectranthus ombrophilus

Taxon classificationPlantaeLamialesLamiaceae

Hedge, Fl. Madag. 175: 166. 1998

76F7CAC2B4C05747B4AEFDBD904F6692

Plectranthus ombrophilus Hedge, Fl. Madag. 175: 166. 1998. Type: Madagascar, haute vallée de la Manampanihy, col de Saindro, Humbert 14005 (holotype: P; isotypes: B, E, G, MO, PRE).

##### Distribution.

Madagascar.

#### Plectranthus oribiensis

Taxon classificationPlantaeLamialesLamiaceae

Codd, Fl. Pl. Africa 46: t. 1809. 1980

186933722A5957FE85687577C346B07D

Plectranthus oribiensis Codd, Fl. Pl. Africa 46: t. 1809. 1980. Type: South Africa, Port Shepstone, Oribi Gorge, van Jaarsveld 2198, cultivated in Pretoria sub Garden 24530 (holotype: PRE).

##### Distribution.

South Africa: KwaZulu-Natal.

#### Plectranthus papilionaceus

Taxon classificationPlantaeLamialesLamiaceae

Ranir. & Phillipson, Candollea 62: 158. 2007

2E02B06139B3526FA1CD82685DE4D729

Fig. 9A

Plectranthus papilionaceus Ranir. & Phillipson, Candollea 62: 158. 2007. Type: Madagascar, Province de Diego-Suarez/Antsiranana: sous-préfecture de Vohémar, commune rurale de Daraina, forêt de Binara, P.Ranirison & L.Nusbaumer PR 993 (holotype: G; isotypes: K, MO, P, TEF).

##### Distribution.

Madagascar.

#### Plectranthus pichompae

Taxon classificationPlantaeLamialesLamiaceae

Hedge, Fl. Madag. 175: 163. 1998

0138C0FC95EE5CFF918686E0762C0811

Plectranthus pichompae Hedge, Fl. Madag. 175: 163. 1998. Type: Madagascar, partie occidentale du massif du Marojejy, de la vallée de l’Ambatoharanana au bassin supérieur de l’Antsahaberoka, Humbert & Saboureau 31546 (holotype: P; isotype P).

##### Distribution.

Madagascar.

#### Plectranthus praetermissus

Taxon classificationPlantaeLamialesLamiaceae

Codd, Fl. Pl. Africa 45: t. 1791 1979

16D6C004841C5B7A9831658786522F6A

Plectranthus praetermissus Codd, Fl. Pl. Africa 45: t. 1791. 1979. Type: South Africa, Port St Johns, Stutterrhiem sub PRE 57330 (holotype: PRE).

##### Distribution.

South Africa: SE. Cape Prov.

#### Plectranthus purpuratus

Taxon classificationPlantaeLamialesLamiaceae

Harv., Thes. Cap. 1: 53. 1860

ECBB4BDCC61D5C55826DAC75D6750686

Plectranthus purpuratus Harv., Thes. Cap. 1: 53. 1860. Type: cultivated, ex Hort., Kew, from seed sent from Port Natal [KwaZulu-Natal: Durban], R. Vause s.n. (holotype: K).

##### Distribution.

South Africa.

#### Plectranthus purpuratus subsp. purpuratus

Taxon classificationPlantaeLamialesLamiaceae

55E492A4B2BD5088A5E08263C10CDD35

##### Distribution.

South Africa: KwaZulu-Natal.

#### Plectranthus purpuratus subsp. montanus

Taxon classificationPlantaeLamialesLamiaceae

 van Jaarsv. & T.J.Edwards, Bothalia 27: 4. 1997

9C9542D8B5AC5F62AB4BB59CA4930494

Fig. 9B

Plectranthus purpuratus subsp. montanus van Jaarsv. & T.J.Edwards, Bothalia 27: 4. 1997. Type: Swaziland, 2631 (Mbabane): Mbabane, Compton 32207 (holotype: NBG).

##### Distribution.

South Africa: S. Mpumalanga to Swaziland.

#### Plectranthus purpuratus subsp. tongaensis

Taxon classificationPlantaeLamialesLamiaceae

 van Jaarsv. & T.J.Edwards, Bothalia 27: 2. 1997

FEA1C7B83B7D58F499E65EA5D6C8EAAD

Plectranthus purpuratus subsp. tongaensis van Jaarsv. & T.J.Edwards, Bothalia 27: 2. 1997. Type: South Africa, KwaZulu-Natal, 2732 (Ubombo): Kosi Bay, (-BB), Van Jaarsveld 12206 (holotype: NBG).

##### Distribution.

South Africa: KwaZulu-Natal.

#### Plectranthus reflexus

Taxon classificationPlantaeLamialesLamiaceae

van Jaarsv. & T.J.Edwards, Fl. Pl. Africa 51: t. 2034 1991

191E3FBD51E752F5800927BB1FAD64D2

Plectranthus reflexus van Jaarsv. & T.J.Edwards, Fl. Pl. Africa 51: t. 2034 1991. Type: South Africa, Port St Johns, western embankment of Bulowe River, van Jaarsveld, Campher & Dekker 10156 (holotype, NBG).

##### Distribution.

South Africa: SE. Cape Prov.

#### Plectranthus rosulatus

Taxon classificationPlantaeLamialesLamiaceae

Hedge, Adansonia, sér. 3, 27: 321. 2005

EE80C631A24856D4A3EE7DAFDBC1DBF2

Plectranthus rosulatus Hedge, Adansonia, sér. 3, 27: 321. 2005. Type: Cultivated plants under glass, Royal Botanic Garden Edinburgh; specimen from Munich Botanic Garden, originally grown from seed collected in NW Madagascar, c. 50 km west of Sambava, in moss cushions in rain forest, by Mangelsdorff HC22 (holotype: E).

##### Distribution.

NW. Madagascar.

#### Plectranthus rubropunctatus

Taxon classificationPlantaeLamialesLamiaceae

Codd, Bothalia 11: 420. 1975

D4522FE29468538A90B8D8E583EB4AA0

Plectranthus rubropunctatus Codd, Bothalia 11: 420. 1975. Type: South Africa, Barberton, Nelshootge Forest Station, Strey 4081 (holotype: PRE).

##### Distribution.

South Africa: Limpopo to Swaziland.

#### Plectranthus rubroviolaceus

Taxon classificationPlantaeLamialesLamiaceae

Hedge, Fl. Madag. 175: 151. 1998

2267087C577854DE8BB0360125854A0A

Plectranthus rubroviolaceus Hedge, Fl. Madag. 175: 151. 1998. Type: Madagascar, contreforts occidentaux du massif du Marojejy, près du col de Boanyanala, limite des bassins de la Lokoho et de l’Androranga, Humbert 23127 (holotype: P; isotypes: BR, G, MO, NY, S, TAN).

##### Distribution.

Madagascar.

#### Plectranthus saccatus

Taxon classificationPlantaeLamialesLamiaceae

Benth. in E.H.F.Meyer, Comm. Pl. Afr. Austr.: 227. 1838

0D35B95CD4D75C12964435023D50AFF7

Plectranthus saccatus Benth. in E.H.F.Meyer, Comm. Pl. Afr. Austr.: 227. 1838. Type: South Africa, Cape, Omsamwubo [Umzimvubu river, near Port St Johns], Drège 4771 (lectotype: K (K000430872), designated by Codd 1975; isolectotypes: G, P, S).

##### Distribution.

South Africa: SE. Cape Prov. to KwaZulu-Natal.

#### Plectranthus saccatus subsp. saccatus

Taxon classificationPlantaeLamialesLamiaceae

E51B98050C205F1D903EB43C3DDECF30

##### Distribution.

South Africa: SE. Cape Prov. to KwaZulu-Natal.

#### Plectranthus saccatus var. saccatus

Taxon classificationPlantaeLamialesLamiaceae

A811943D252A543AAD87D015D0B9903E

##### Distribution.

SE. Cape Prov. to KwaZulu-Natal.

#### Plectranthus saccatus var. longitubus

Taxon classificationPlantaeLamialesLamiaceae

AE8B205C16CA531E8C92F7A19FBBAFCB

Fig. 9C

Plectranthus saccatus var. longitubus Codd, Bothalia 11: 428. 1975. Type: South Africa, Natal, Ingwavuma District, 2732 (Ubombo), Gwalaweni forest (-AC), Edwards 2930 (holotype: PRE).

##### Distribution.

South Africa: KwaZulu-Natal.

#### Plectranthus saccatus subsp. pondoensis

Taxon classificationPlantaeLamialesLamiaceae

 van Jaarsv. & T.J.Edwards, Bothalia 27: 4. 1997

9B6D5874C470538FBD489B26215517E4

Plectranthus saccatus subsp. pondoensis van Jaarsv. & T.J.Edwards, Bothalia 27: 4. 1997. Type: South Africa KwaZulu-Natal, 3030 (Port Shepstone): Oribi Gorge, (-CA), E. van Jaarsveld 2201 (holotype: PRE).

##### Distribution.

South Africa: SE. Cape Prov. to S. KwaZulu-Natal.

#### Plectranthus scaposus

Taxon classificationPlantaeLamialesLamiaceae

Hedge, Fl. Madag. 175: 157. 1998

F8E34777919555C4919489ECD792053B

Plectranthus scaposus Hedge, Fl. Madag. 175: 157. 1998. Type: Madagascar, Antsiranana, rés. nat. du Marojejy, pentes au nord d’Ambatosoratra, Miller 4232 (holotype: P; isotype: MO).

##### Distribution.

Madagascar.

#### Plectranthus secundiflorus

Taxon classificationPlantaeLamialesLamiaceae

(Baker) Hedge, Fl. Madag. 175: 180. 1998

B2175DB4E9CC5477AC41BA30054327C9

Orthosiphon secundiflorus Baker, J. Linn. Soc., Bot. 21: 433. 1885. Type: Madagascar, central, Baron 1226 (holotype: K).

##### Distribution.

Madagascar.

#### Plectranthus strangulatus

Taxon classificationPlantaeLamialesLamiaceae

A.J.Paton, Fl. Trop. E. Afr., Lamiac.: 246. 2009

1601029D97B4558CBAE1FF79455F4869

Plectranthus strangulatus A.J.Paton, Fl. Trop. E. Afr., Lamiac.: 246. 2009. Type: Tanzania, Morogoro District: Uluguru Mts, Kitundu, E.M. Bruce 957 (holotype: K (K000431930); isotypes: BR, K (K000431929)).

##### Distribution.

Tanzania (Uluguru Mts.).

#### Plectranthus strigosus

Taxon classificationPlantaeLamialesLamiaceae

Benth. ex E.Mey., Comm. Pl. Afr. Austr.: 229. 1838

409819377B235A12B956B271A2D3EDC4

Plectranthus strigosus Benth. ex E.Mey., Comm. Pl. Afr. Austr.: 229. 1838. Type: South Africa, Cape, Olifantshoek Forest (Alexandria), 1836, Eklon s.n. (lectotype: K (K000430853), designated by Codd 1975).
Plectranthus parviflorus Gürke in C.E.O.Kuntze, Revis. Gen. Pl. 3(2): 261. 1898., nom. illeg., non P. parviflorus Willd.
Plectranthus kuntzeanus Domin, Biblioth. Bot. 89: 564. 1928. Type: South Africa, East London, Kuntze s.n. (lectotype: NY, designated by Codd 1975).

##### Distribution.

South Africa: S. Cape Prov.

#### Plectranthus stylesii

Taxon classificationPlantaeLamialesLamiaceae

T.J.Edwards, Bothalia 35: 149. 2005

316BE5369FBE54E7AC4BA86B45EDE606

Plectranthus stylesii T.J.Edwards, Bothalia 35: 149. 2005. Type: South Africa, Port St Johns, Msikaba River Gorge, c. 4km from the mouth, Edwards 3259 (holotype: NU; isotypes: E, GRA, K, PRE).

##### Distribution.

South Africa: Eastern Cape Prov.

#### Plectranthus swynnertonii

Taxon classificationPlantaeLamialesLamiaceae

S.Moore, J. Linn. Soc., Bot. 40: 176. 1911

097220843D3058A697BE79DEE617D46D

Plectranthus swynnertonii S.Moore, J. Linn. Soc., Bot. 40: 176. 1911. Type: Zimbabwe, Chirinda forest, Swynnerton 337 (holotype: K).

##### Distribution.

S. Trop. Africa to South Africa, Mpumalanga.

#### Plectranthus trilobus

Taxon classificationPlantaeLamialesLamiaceae

Hedge, Fl. Madag. 175: 205. 1998

B386CECC7A385A50BDCE2272A6C31296

Plectranthus trilobus Hedge, Fl. Madag. 175: 205. 1998. Type: Madagascar, dans le haut bassin de l’Andranomaizina, affluent de l’Imonty, au-dessous du col d’Etsilotsilo, Capuron SF 22442 (holotype: P).

##### Distribution.

Madagascar.

#### Plectranthus verticillatus

Taxon classificationPlantaeLamialesLamiaceae

(L.f.) Druce, Rep. Bot. Soc. Exch. Club Brit. Isles 4: 640. 1916 (publ. 1917)

B5D1AB15F38E5ED8B0ADCDE77017F840

Ocimum verticillatum L.f., suppl. Pl.: 276. 1782.
Plectranthus thunbergii Benth., Labiat. Gen. Spec.: 37. 1832., nom. illeg. Type: South Africa, Cape, Thunbergs.n. (holotype: LINN 749.4).
Ocimum racemosum Thunb., Prodr. Pl. Cap.: 96. 1800. Type: South Africa, Cape, Houteniquas, Thunberg s.n. (holotype: UPS; isotype: SBT).
Plectranthus nummularius Briq., Bull. Herb. Boissier, sér. 2, 3: 1072. 1903. Type: South Africa, KwaZulu-Natal, Camperdown, n.d., Rehmann 7702 (holotype: Z).

##### Distribution.

S. Mozambique to South Africa.

#### Plectranthus vestitus

Taxon classificationPlantaeLamialesLamiaceae

Benth., Labiat. Gen. Spec.: 32. 1832

A5FF108F6640526F8B0F01CB527F7066

Fig. 9D

Plectranthus vestitus Benth., Labiat. Gen. Spec.: 32. 1832. Type: Madagascar, Prov. de Bezanozano, Bojer s.n. (holotype: K).

##### Distribution.

Madagascar.

#### Plectranthus vinaceus

Taxon classificationPlantaeLamialesLamiaceae

Hedge, Fl. Madag. 175: 148. 1998

39E59707D1F45D10826E398496924F0B

Plectranthus vinaceus Hedge, Fl. Madag. 175: 148. 1998. Type: Madagascar, sommet oriental du massif du Marojejy, ál’ouest de la haute Manantenina, affluent de la Lokoho, Humbert 22737 (holotype: P).

##### Distribution.

Madagascar.

#### Plectranthus zuluensis

Taxon classificationPlantaeLamialesLamiaceae

T.Cooke, Bull. Misc. Inform. Kew 1909: 379. 1909

E6F01BD1B72058148AA969D3EF039F77

Plectranthus zuluensis T.Cooke, Bull. Misc. Inform. Kew 1909: 379. 1909. Type: South Africa, Natal, Gerrard 1675 (holotype: K).

##### Distribution.

South Africa: SE. Cape Prov. to Swaziland

### Unplaced names

*Anisochilus siamensis* Ridl., J. Straits Branch Roy. Asiat. Soc. 59: 159. 1911. Type: Thailand, Bangsaphan Distr., Prachuap Khirikhan Province, Keith s.n. (not seen- see also Suddee & Paton (2009)).

*Coleus anamallayensis* Bedd., Madras J. Lit. Sci., ser. 2, 22: 73. 1861. Type: not seen, not cited in protologue.

*Coleus aulihanensis* Schweinf. & Volkens, Liste Pl. Somalis: 211. 1897. Type: Ethiopia, Aulihan, Dec. 1895 to Jan 1996, Schweinfurth & Volkens s.n. (not seen). Protologue suggests similarity to *C. igniarius*.

*Coleus kisanfuensis* De Wild., Contr. Fl. Katanga: 174. 1921. Types: Democratic Republic of Congo, Elizabethville, Hock s.n. (syntype: not seen); Kiasanfu, Hock s.n. (syntype: not seen).

*Coleus luengerensis* Gürke, Bot. Jahrb. Syst. 38: 167. 1906. Type: Tanzania, Usambara, Tanga, x. 1895, Heinsen 121 (syntype: B, destroyed); Sept. 1902, Engler 890 (syntype: B, destroyed).

*Coleus mechowianus* Briq., Bot. Jahrb. Syst. 19: 183. 1894. Type: Angola, Pungo Andongo, Mechow 123 (B, holotype destroyed). Protologue suggests similarity to *C. tenuicaulis*.

*Coleus nervosus* Briq., Bot. Jahrb. Syst. 19: 185. 1894. Type: DRC; South Central Kasongo; near the Lomami River, Pogge 1034 (B, holotype, destroyed).

*Coleus parensis* Gürke, Bot. Jahrb. Syst. 41: 317. 1908. Types: Tanzania, Pare District: foot of Pare Mts, Sengina–Simba, Engler 1620 (syntype: B, destroyed) & Uluguru Mts, Meyer 188 (syntype: B, destroyed).

*Coleus petrophilus* Gürke, Bot. Jahrb. Syst. 41: 319. 1908. Type: Tanzania, Lushoto District: W Usambara Mts, Mbalu, Engler 1488 (holotype: B, destroyed)

*Coleus petersianus* Vatke, Linnaea 40: 180. 1876. Type: Mozambique, Rios de Sena, *Peters* s.n. (holotype: B, destroyed). The protologue suggests that this is either *C. bojeri* or *C. welwitschii*.

*Coleus poggeanus* Briq., Bot. Jahrb. Syst. 19: 182. 1894. Type: DRC, Mussumba des Muata Jamwo, Jan. 1876, Pogge 364 (type: B, destroyed).

*Coleus preussii* Gürke, Bot. Jahrb. Syst. 19: 219. 1894. Type: Cameroon, Buea, Preuss 691 (B, destroyed, isotype); Dusen 309 (B, destroyed).

*Coleus saxicola* Gürke, Bot. Jahrb. Syst. 41: 316. 1908. Type: Tanzania, Lushoto District: W Usambara Mts, Kwai, Eick 27 (syntype: B, destroyed) & Engler 2250 (syntype: B, destroyed).

*Coleus subscandens* Gürke, Bot. Jahrb. Syst. 41: 319. 1908. Type: Tanzania, Lushoto District: W Usambara Mts, Mlalo, Engler 1394 (holotype: B, destroyed).

*Coleus ulugurensis* Gürke, Bot. Jahrb. Syst. 28: 470. 1900. Type: Tanzania, Morogoro District: NE Uluguru Mts, Kitope, Stuhlmann 9060 (holotype: B, destroyed).

*Coleus viridis* Briq., Bot. Jahrb. Syst. 19: 181. 1894. Type: DRC, Mussumba, Pogge 365, (B, holotype, destroyed).

*Coleus wugensis* Gürke, Bot. Jahrb. Syst. 41: 318. 1908. Type: Tanzania, Lushoto District: W Usambara Mts, Wuga area, Engler 1165 (holotype: B, destroyed)

*Plectranthus crenatus* Gürke, Bot. Jahrb. Syst. 19: 208. 1894. Type: Tanzania, Lushoto District: Usambara Mts, Maschewa, Holst 8730 (holotype: B, destroyed).

*Plectranthus eminii* Gürke, Bot. Jahrb. Syst. 19: 207. 1894. Type: Tanzania, Bukoba, Stuhlmann 3882 (holotype: B, destroyed)

*Plectranthus holstii* Gürke, Bot. Jahrb. Syst. 19: 204. 1894. Type: Tanzania, Lushoto District: W Usambara Mts, Mlalo, Holst 248 (holotype: B, destroyed). Perhaps close to *E. orbiculare* (Gürke) Mwany. & A.J.Paton.

*Plectranthus lycopifolius* A.Rich., Tent. Fl. Abyss. 2: 181. 1850. Type Ethiopia, Tigre, near Adoa, Quartin-Dillon (holotype: P, not seen, no Richard specimen in P which matches protologue.).

*Plectranthus mbaluensis* Gürke, Bot. Jahrb. Syst. 36: 136. 1905. Type: Tanzania, W Usambara Mts, Mbalu, Engler 1479 (holotype: B, destroyed). Note. Similar to *E. kamerunense*.

*Plectranthus lindblomii* T.C.E.Fr., Notizbl. Bot. Gart. Berlin-Dahlem 11: 28. 1930. Type: Kenya, Mt Elgon, Kitosh, Lindblom s.n. (holotype: B, destroyed).

*Plectranthus marquesii* Gürke, Bol. Soc. Brot. 16: 70. 1896. Type: Angola, R. Cuillo, Marques 197 (holotype: B, destroyed).

*Plectranthus mildbraedii* Perkins in G.W.J.Mildbraed (ed.), Wiss. Erg. Deut. Zentr.-Afr. Exped., Bot. 2: 549. 1913. Type: Uganda, Virunga Mts (Vulkan Mts), Karissimbi, Mildbraed 1618 (holotype: B, destroyed). Note. Protologue suggests relationship to *E. longipes* or *E. kamerunense*.

*Plectranthus microphyllus* Gürke, Bot. Jahrb. Syst. 28: 467. 1900. Type: Tanzania, Morogoro District: E Uluguru Mts, Stuhlmann 8748 (holotype: B, destroyed).

*Plectranthus miserabilis* Briq., Bot Jarbh. Syst 19: 180. 1895. Type: Democratic Republic of Congo, South Central Lunda; Mukenge, in Bashilange district, Pogge 1022 (type: not seen).

*Plectranthus monticola* Gürke, Bot. Jahrb. Syst. 28: 468. 1900. Type: Tanzania, Morogoro District: Uluguru Mts, Lukwangule, Stuhlmann 9166 (holotype: B, destroyed). Schlieben 2854 (BM, LISC) has been determined as *C. monticola*. These specimens are *C. trullatus*. However the description is a better match for the closely related *C. triangularis* which also occurs in the Uluguru Mts.

*Plectranthus nodosus* Bellardi ex Colla, Herb. Pedem. 4: 475. 1835. Listed as dubious species, no type details.

*Plectranthus pallidus* Biroli ex Colla, Herb. Pedem. 4: 475. 1835. Listed as dubious species, no type details.

*Plectranthus pendulus* Gürke, Bot. Jahrb. Syst. 41: 314. 1908. Type: Tanzania, Lushoto District: W Usambara Mts, Engler 1180 (holotype: B, destroyed).

*Plectranthus rupicola* Gürke, Bot. Jahrb. Syst. 41: 313. 1908. Type: Tanzania, Lushoto District: W Usambara Mts, Mbalu, Engler 1457 (holotype: B, destroyed).

*Plectranthus pratensis* Gürke, Bot. Jahrb. Syst. 19: 201. 1894. Type: Tanzania, Kilimanjaro, Marangu, Volkens 634 & W Usambara Mts, Mlalo, Holst 459 (syntype: B, destroyed).

*Plectranthus sakarensis* Gürke, Bot. Jahrb. Syst. 41: 313. 1908. Type: Tanzania, Lushoto District: W Usambara Mts, Sakare, Engler 989a (holotype: B, destroyed)

*Plectranthus schweinfurthii* Sprenger, Wiener Ill. Gart.-Zeitung 19: 1. 1894, non *C. schweinfurthii* Vatke Type: Arabian Peninsula (type: not seen).

*Plectranthus saxatilis* Gürke, Bot. Jahrb. Syst. 36: 133. 1905. Types: Tanzania, Lushoto District: W Usambara Mts, Mlalo, Holst 345 & 611 & Engler 1485 (syntypes: B, destroyed).

*Plectranthus stuhlmannii* Gürke, Bot. Jahrb. Syst. 28: 469. 1900. Type: Tanzania, Morogoro District: S Uluguru Mts, Dundumi stream, Stuhlmann 9291 (holotype: B, destroyed) Protologue suggests similarity to *E. laxiflorum*.

*Plectranthus usambarensis* Gürke, Bot. Jahrb. Syst. 19: 207. 1894. Type: Tanzania, Lushoto District: Usambara Mts, Mlalo, Holst 487 (holotype: B, destroyed).

*Saccostoma urticifolia* Wall. ex Voigt Hortus Suburbanus Calcuttensis 449. 1845. Type: not seen.

*Solenostemon bullatus* Briq., Bot. Jahrb. Syst. 19: 180. 1894.

*Coleus bullulatus* Briq., Bull. Soc. Roy. Bot. Belgique 37: 69. 1899. nom. illeg. Type: DRC, Lulua River, 9.30 S, May 1876, Pogge 356. (holotype: not seen)

### Excluded names

*Coleus wulfenioides* Diels: ***Orthosiphon wulfenioides*** (Diels) Hand.-Mazz.

*Plectranthus adenanthus* Diels: ***Isodon adenanthus*** (Diels) Kudô

*Plectranthus adenolomus* Hand.-Mazz.: ***Isodon adenolomus*** (Hand.-Mazz.) H.Hara

*Plectranthus albidus* Baker: ***Capitanopsis albida*** (Baker) Hedge

*Plectranthus allenii* C.H.Wright: ***Orthosiphon allenii*** (C.H.Wright) Codd

*Plectranthus amethystoides* Benth.: ***Isodon amethystoides*** (Benth.) H.Hara

*Plectranthus angustifolius* Dunn: ***Isodon angustifolius*** (Dunn) Kudô

*Plectranthus assamicus* Mukerjee: ***Isodon assamicus*** (Mukerjee) H.Hara

*Plectranthus axillariflorus* Honda: **Isodon shikokianus var. intermedius** (Kudô) Murata

*Plectranthus barrelieri* (Roth) Spreng.: ***Ocimum basilicum*** L.

*Plectranthus benthamianus* Miq.: ***Isodon coetsa*** (Buch.-Ham. ex D.Don) Kudô

*Plectranthus bifidocalyx* Dunn: ***Isodon macrocalyx*** (Dunn) Kudô

*Plectranthus bolusii* T.Cooke: ***Orthosiphon thymiflorus*** (Roth) Sleesen

*Plectranthus brandisii* Prain: ***Isodon walkeri*** (Arn.) H.Hara

*Plectranthus brevifolius* Hand.-Mazz.: ***Isodon brevifolius*** (Hand.-Mazz.) H.W.Li

*Plectranthus buckia* Buch.-Ham. ex D.Don: ***Isodon ternifolius*** (D.Don) Kudô

*Plectranthus buergeri* Miq.: **Isodon japonicus var. japonicus**

*Plectranthus bullatus* Benth.: ***Isodon nilgherricus*** (Benth.) H.Hara

*Plectranthus bullatus* Robyns & Lebrun: ***Isodon ramosissimus*** (Hook.f.) Codd

*Plectranthus bulleyanus* Diels: ***Isodon bulleyanus*** (Diels) Kudô

*Plectranthus calcicola* Hand.-Mazz.: ***Isodon calcicola*** (Hand.-Mazz.) H.Hara

Plectranthus calcicola var. subcalvus Hand.-Mazz.: **Isodon calcicola var. subcalvus** (Hand.-Mazz.) H.W.Li

*Plectranthus capillipes* Benth.: ***Isodon capillipes*** (Benth.) H.Hara

*Plectranthus cardiophyllus* Hemsl.: **Heterolamium debile var. cardiophyllum** (Hemsl.) C.Y.Wu

*Plectranthus cavaleriei* H.Lév.: ***Isodon coetsa*** (Buch.-Ham. ex D.Don) Kudô

*Plectranthus chamaedrys* H.Lév., = ***Triumfetta annua****L.* (Malvaceae)

*Plectranthus chenmui* Y.Z.Sun: ***Isodon phyllopodus*** (Diels) Kudô

*Plectranthus chienii* Y.Z.Sun: ***Isodon hispidus*** (Benth.) Murata

*Plectranthus coetsa* Buch.-Ham. ex D.Don: ***Isodon coetsa*** (Buch.-Ham. ex D.Don) Kudô

*Plectranthus colaniae* Doan: ***Isodon colaniae*** H.Hara ex Suddee & A.J.Paton

*Plectranthus coloratus* D.Don: ***Platostoma coloratum*** (D.Don) A.J.Paton

*Plectranthus coreanus* Vaniot: ***Isodon inflexus*** (Thunb.) Kudô

*Plectranthus crenulatus* Hook.f.: ***Paralamium griffithii*** (Hook.f.) Suddee & A.J.Paton

*Plectranthus daitonensis* Hayata: ***Isodon amethystoides*** (Benth.) H.Hara

*Plectranthus daoi* Phuong: ***Paralamium griffithii*** (Hook.f.) Suddee & A.J.Paton

*Plectranthus dawoensis* Hand.-Mazz.: ***Isodon dawoensis*** (Hand.-Mazz.) H.Hara

*Plectranthus dhankutanus* (Murata) Bennet: ***Isodon dhankutanus*** Murata

*Plectranthus dichromophyllus* Diels: ***Isodon rubescens*** (Hemsl.) H.Hara

*Plectranthus diffusus* Merr.: ***Isodon coetsa*** (Buch.-Ham. ex D.Don) Kudô

*Plectranthus discolor* Dunn: ***Isodon parvifolius*** (Batalin) H.Hara

*Plectranthus drogotschiensis* Hand.-Mazz.: ***Isodon barbeyanus*** (H.Lév.) H.W.Li

*Plectranthus drosocarpus* Hand.-Mazz.: ***Isodon macrocalyx*** (Dunn) Kudô

*Plectranthus dubius* Vahl ex Benth.: ***Isodon amethystoides*** (Benth.) H.Hara

*Plectranthus effusus* (Maxim.) Honda: ***Isodon effusus*** (Maxim.) H.Hara

*Plectranthus enanderianus* Hand.-Mazz.: ***Isodon enanderianus*** (Hand.-Mazz.) H.W.Li

*Plectranthus eriocalyx* Dunn: ***Isodon eriocalyx*** (Dunn) Kudô

*Plectranthus esquirolii* H.Lév.: **Isodon lophanthoides var. lophanthoides**

*Plectranthus etiolatus* Chiov.: ***Platostoma rotundifolium*** (Briq.) A.J.Paton

*Plectranthus excisoides* Y.Z.Sun ex C.H.Hu: ***Isodon excisoides*** (Y.Z.Sun ex C.H.Hu) H.Hara

*Plectranthus excisus* Maxim.: ***Isodon excisus*** (Maxim.) Kudô

Plectranthus excisus var. shikokianus Makino: ***Isodon shikokianus*** (Makino) H.Hara

*Plectranthus fangii* Y.Z.Sun: **Isodon lophanthoides var. lophanthoides**

*Plectranthus flavidus* Hand.-Mazz.: ***Isodon flavidus*** (Hand.-Mazz.) H.Hara

*Plectranthus forrestii* Diels: ***Isodon forrestii*** (Diels) Kudô

*Plectranthus garrettii* Craib: **Platostoma calcaratum var. garrettii** (Craib) Suddee

*Plectranthus gerardianus* Benth.: ***Isodon lophanthoides*** (Buch.-Ham. ex D.Don) H.Hara

*Plectranthus gesneroides* J.Sinclair: ***Isodon gesneroides*** (J.Sinclair) H.Hara

*Plectranthus glaucocalyx* Maxim.: **Isodon japonicus var. glaucocalyx** (Maxim.) H.W.Li

*Plectranthus graciliflorus* Benth.: **Isodon lophanthoides var. graciliflorus** (Benth.) H.Hara

*Plectranthus grandifolius* Hand.-Mazz.: ***Isodon grandifolius*** (Hand.-Mazz.) H.Hara

*Plectranthus griffithii* Hook.f.: ***Paralamium griffithii*** (Hook.f.) Suddee & A.J.Paton

*Plectranthus grosseserratus* Dunn: ***Isodon grosseserratus*** (Dunn) Kudô

*Plectranthus hanceiformis* H.Lév.: ***Teucrium bidentatum*** Hemsl.

*Plectranthus henryi* Hemsl.: ***Isodon henryi*** (Hemsl.) Kudô

*Plectranthus hians* Benth.: ***Isodon nigrescens*** (Benth.) H.Hara

*Plectranthus hirtellus* Hand.-Mazz.: ***Isodon hirtellus*** (Hand.-Mazz.) H.Hara

*Plectranthus hispidus* Benth.: ***Isodon hispidus*** (Benth.) Murata

Plectranthus hispidus var. glabrior Benth.: **Isodon lophanthoides var. lophanthoides**

*Plectranthus hoslundioides* Baker: ***Isodon ramosissimus*** (Hook.f.) Codd

*Plectranthus hosseusii* Muschl.: ***Isodon ternifolius*** (D.Don) Kudô

*Plectranthus inconspicuus* Miq.: ***Isodon inflexus*** (Thunb.) Kudô

*Plectranthus indicus* (Roth) Spreng.: ***Ocimum filamentosum*** Forssk.

*Plectranthus inflexus* (Thunb.) Vahl ex Benth.: ***Isodon inflexus*** (Thunb.) Kudô

Plectranthus inflexus var. macrophyllus Maxim.: ***Isodon inflexus*** (Thunb.) Kudô

Plectranthus inflexus f. macrophyllus (Maxim.) Kitag.: ***Isodon inflexus*** (Thunb.) Kudô

Plectranthus inflexus f. subglabrus Makino: ***Isodon inflexus*** (Thunb.) Kudô

Plectranthus inflexus var. umbrosus Maxim.: ***Isodon umbrosus*** (Maxim.) H.Hara

Plectranthus inflexus var. verticillatus Makino: ***Isodon inflexus*** (Thunb.) Kudô

*Plectranthus intermedius* Zoll. & Moritzi: ***Isodon coetsa*** (Buch.-Ham. ex D.Don) Kudô

*Plectranthus intermedius* Benth. nom. illeg.: ***Isodon coetsa*** (Buch.-Ham. ex D.Don) Kudô

*Plectranthus irroratus* Forrest ex Diels: ***Isodon irroratus*** (Forrest ex Diels) Kudô

*Plectranthus japonicus* (Burm.f.) Koidz.: ***Isodon japonicus*** (Burm.f.) H.Hara

Plectranthus japonicus var. macraei (Benth.) S.R.Sriniv.: ***Isodon coetsa*** (Buch.-Ham. ex D.Don) Kudô

*Plectranthus javanicus* (Blume) Benth.: ***Isodon coetsa*** (Buch.-Ham. ex D.Don) Kudô

*Plectranthus kameba* (Okuyama ex Ohwi) Ohwi: ***Isodon umbrosus*** (Maxim.) H.Hara

*Plectranthus kurzii* Prain: ***Isodon kurzii*** (Prain) H.Hara

*Plectranthus lanceus* Nakai: **Isodon shikokianus var. intermedius** (Kudô) Murata

*Plectranthus lasiocarpus* Hayata: ***Isodon serra*** (Maxim.) Kudô

*Plectranthus leptobotrys* Diels: ***Isodon coetsa*** (Buch.-Ham. ex D.Don) Kudô

*Plectranthus leucanthus* Diels: ***Isodon phyllopodus*** (Diels) Kudô

*Plectranthus leucophyllus* Dunn: ***Isodon leucophyllus*** (Dunn) Kudô

*Plectranthus longicornis* F.Muell.: ***Platostoma longicorne*** (F.Muell.) A.J.Paton

*Plectranthus longitubus* Miq.: ***Isodon longitubus*** (Miq.) Kudô

Plectranthus longitubus var. albiflorus Makino: ***Isodon longitubus*** (Miq.) Kudô

Plectranthus longitubus var. contractus Maxim.: ***Isodon longitubus*** (Miq.) Kudô

Plectranthus longitubus var. effusus Maxim.: ***Isodon effusus*** (Maxim.) H.Hara

*Plectranthus lophanthoides* (Buch.-Ham. ex D.Don) Grierson & D.G.Long: ***Isodon lophanthoides*** (Buch.-Ham. ex D.Don) H.Hara

*Plectranthus loxothyrsus* Hand.-Mazz.: ***Isodon loxothyrsus*** (Hand.-Mazz.) H.Hara

*Plectranthus macraei* Benth.: ***Isodon coetsa*** (Buch.-Ham. ex D.Don) Kudô

*Plectranthus macranthus* Hook.f.: ***Siphocranion macranthum*** (Hook.f.) C.Y.Wu

*Plectranthus macrocalyx* Dunn: ***Isodon macrocalyx*** (Dunn) Kudô

*Plectranthus maddenii* Benth. ex Hook.f.: ***Isodon coetsa*** (Buch.-Ham. ex D.Don) Kudô

*Plectranthus mairei* H.Lév.: ***Isodon coetsa*** (Buch.-Ham. ex D.Don) Kudô

*Plectranthus marmoritis* Hance: ***Orthosiphon thymiflorus*** (Roth) Sleesen

*Plectranthus maximowiczii* Miq.: **Isodon japonicus var. glaucocalyx** (Maxim.) H.W.Li

*Plectranthus meeboldii* W.W.Sm.: ***Isodon meeboldii*** (W.W.Sm.) Suddee

*Plectranthus megathyrsus* Diels: ***Isodon megathyrsus*** (Diels) H.Hara

*Plectranthus melissoides* Benth.: ***Isodon melissoides*** (Benth.) H.Hara

*Plectranthus menthoides* Moritzi: ***Isodon coetsa*** (Buch.-Ham. ex D.Don) Kudô

*Plectranthus menthoides* Willd.: ***Ocimum filamentosum*** Forssk.

*Plectranthus menthoides* Benth.: ***Isodon coetsa*** (Buch.-Ham. ex D.Don) Kudô

*Plectranthus micranthus* Spreng.: ***Basilicum polystachyon*** (L.) Moench

*Plectranthus mollissimus* Wall. ex Benth.: **Orthosiphon rubicundus var. rubicundus**

*Plectranthus monachorum* (L.) Spreng.: ***Ocimum tenuiflorum*** L.

*Plectranthus moschatus* R.Br.,: ***Basilicum polystachyon*** (L.) Moench

*Plectranthus moslifolius* H.Lév.: ***Isodon nervosus*** (Hemsl.) Kudô

*Plectranthus muliensis* W.W.Sm.: ***Isodon muliensis*** (W.W.Sm.) Kudô

*Plectranthus multiflorus* Hochst. ex Benth.: ***Tetradenia multiflora*** (Benth.) Phillipson

*Plectranthus namikawanus* (Murata) Bennet: ***Isodon namikawanus*** Murata

*Plectranthus nankinensis* Spreng., Syst. Veg. 2: 691. 1825). based on *Dentidia nankinensis* Lour, Flora Cochinensis ed. 2: 448. 1793. Type: China, Nanjing, Loureiro s.n. (holotype: P) = ***Perilla ocymoides*** L. **var. crispa** Benth.

*Plectranthus neglectus* Dinter: ***Aeollanthus neglectus*** (Dinter) Launert

*Plectranthus nepetifolius* Benth.: ***Isodon wightii*** (Benth.) H.Hara

*Plectranthus nervosus* Hemsl.: ***Isodon nervosus*** (Hemsl.) Kudô

*Plectranthus nigrescens* Benth.: ***Isodon nigrescens*** (Benth.) H.Hara

Plectranthus nigrescens var. pilosus L.H.Cramer: ***Isodon nigrescens*** (Benth.) H.Hara

*Plectranthus nilgherricus* Benth.: ***Isodon nilgherricus*** (Benth.) H.Hara

*Plectranthus nudipes* Hemsl.: ***Siphocranion nudipes*** (Hemsl.) Kudô

*Plectranthus oblongifolius* Wall. ex Benth.: **Isodon lophanthoides var. graciliflorus** (Benth.) H.Hara

*Plectranthus oreophilus* Diels: ***Isodon oreophilus*** (Diels) A.J.Paton & Ryding

Plectranthus oreophilus var. elongatus Hand.-Mazz.: ***Isodon oreophilus*** (Diels) A.J.Paton & Ryding

*Plectranthus oresbius* W.W.Sm.: ***Isodon oresbius*** (W.W.Sm.) Kudô

*Plectranthus ovatifolius* Oliv.: ***Ocimum lamiifolium*** Hochst. ex Benth.

*Plectranthus pachythyrsus* Hand.-Mazz.: ***Isodon leucophyllus*** (Dunn) Kudô

*Plectranthus palisotii* Benth.: ***Plectranthus monostachyus*** (P.Beauv.) B.J.Pollard

*Plectranthus paniculatus* Baker: ***Isodon ramosissimus*** (Hook.f.) Codd

*Plectranthus pantadenius* Hand.-Mazz.: ***Isodon pantadenius*** (Hand.-Mazz.) H.W.Li

*Plectranthus parviflorus* R.Br.: ***Basilicum polystachyon*** (L.) Moench

*Plectranthus parvifolius* (Batalin) C.Pei: ***Isodon parvifolius*** (Batalin) H.Hara

*Plectranthus parvifolius* Talbot, J. Bombay Nat. Hist. Soc. 11: 238 plate x. 1897.

S. India. Type: Illustration cited above: ***Isodon***

*Plectranthus patchoulii* C.B.Clarke ex Hook.f.: ***Microtoena patchoulii*** (C.B.Clarke ex Hook.f.) C.Y.Wu & S.J.Hsuan

*Plectranthus pekinensis* Maxim.: ***Isodon amethystoides*** (Benth.) H.Hara

*Plectranthus pharicus* Prain: ***Isodon pharicus*** (Prain) Murata

*Plectranthus phulchokiensis* (Murata) Bennet: ***Isodon phulchokiensis*** (Murata) H.Hara

*Plectranthus phyllopodus* Diels: ***Isodon phyllopodus*** (Diels) Kudô

*Plectranthus phyllostachys* Diels: ***Isodon phyllostachys*** (Diels) Kudô

*Plectranthus pleiophyllus* Diels: ***Isodon pleiophyllus*** (Diels) Kudô

*Plectranthus polystachys* Y.Z.Sun ex C.H.Hu: ***Isodon coetsa*** (Buch.-Ham. ex D.Don) Kudô

*Plectranthus prainianus* (H.Lév.) Dunn: ***Siphocranion macranthum*** (Hook.f.) C.Y.Wu

*Plectranthus provicarii* H.Lév.: ***Isodon bulleyanus*** (Diels) Kudô

*Plectranthus pulneyensis* Hook.f.: ***Isodon wightii*** (Benth.) H.Hara

*Plectranthus racemosus* Hemsl.: ***Isodon racemosus*** (Hemsl.) Murata

*Plectranthus ramosissimus* Hook.f.: ***Isodon ramosissimus*** (Hook.f.) Codd

*Plectranthus rariflorus* Hochst.: ***Endostemon obtusifolius*** (E.Mey.) N.E.Br.

*Plectranthus repens* Wall. ex Benth.: ***Isodon repens*** (Wall. ex Benth.) Murata

*Plectranthus ricinispermus* Pamp.: ***Isodon rubescens*** (Hemsl.) H.Hara

*Plectranthus riparius* Hochst.: ***Tetradenia riparia*** (Hochst.) Codd

*Plectranthus rivularis* Wight ex Hook.f.: ***Isodon rivularis*** (Wight ex Hook.f.) H.Hara

*Plectranthus rosthornii* Diels: ***Isodon rosthornii*** (Diels) Kudô

*Plectranthus rubescens* Hemsl.: ***Isodon rubescens*** (Hemsl.) H.Hara

*Plectranthus rubicundus* D.Don: ***Orthosiphon rubicundus*** (D.Don) Benth.

*Plectranthus rufescens* Benth.: ***Isodon coetsa*** (Buch.-Ham. ex D.Don) Kudô

*Plectranthus rugosiformis* Hand.-Mazz.: ***Isodon rugosiformis*** (Hand.-Mazz.) H.Hara

*Plectranthus rugosus* Wall. ex Benth.: ***Isodon rugosus*** (Wall. ex Benth.) Codd

Plectranthus rugosus var. tomentosus Benth.: ***Isodon coetsa*** (Buch.-Ham. ex D.Don) Kudô

*Plectranthus rupicola* Dinter ex Gooss.: ***Aeollanthus buchnerianus*** Briq.

*Plectranthus salicarius* Hand.-Mazz.: ***Isodon nervosus*** (Hemsl.) Kudô

*Plectranthus salvioides* (B.Heyne ex Roth) Benth.: ***Perilla frutescens*** L.

*Plectranthus scabrellus* Benth.: ***Isodon nigrescens*** (Benth.) H.Hara

*Plectranthus schimperi* Vatke: ***Isodon schimperi*** (Vatke) J.K.Morton

*Plectranthus schimperi* Chiov., nom. illeg.: ***Isodon schimperi*** (Vatke) J.K. Morton

*Plectranthus scrophularioides* Wall. ex Benth.: ***Isodon scrophularioides*** (Wall. ex Benth.) Murata

*Plectranthus sculponiatus* Vaniot: ***Isodon sculponiatus*** (Vaniot) Kudô

*Plectranthus serra* Maxim.: ***Isodon serra*** (Maxim.) Kudô

*Plectranthus setschwanensis* Hand.-Mazz.: ***Isodon setschwanensis*** (Hand.-Mazz.) H.Hara

*Plectranthus shikokianus* (Makino) Makino: ***Isodon shikokianus*** (Makino) H.Hara

Plectranthus shikokianus var. intermedius (Kudô) Ohwi: **Isodon shikokianus var. intermedius** (Kudô) Murata

Plectranthus shikokianus var. occidentalis (Murata) Ohwi: **Isodon shikokianus var. occidentalis** Murata

*Plectranthus sinensis* Miq.: ***Isodon amethystoides*** (Benth.) H.Hara

*Plectranthus smithianus* Hand.-Mazz.: ***Isodon smithianus*** (Hand.-Mazz.) H.Hara

*Plectranthus stocksii* Hook.f.: **Isodon lophanthoides var. lophanthoides**

*Plectranthus stracheyi* Benth. ex Hook.f.: ***Isodon walkeri*** (Arn.) H.Hara

*Plectranthus striatus* Hook.f. & Thomson: **Isodon lophanthoides var. lophanthoides**

*Plectranthus striatus* Wight ex Hook.f.: ***Isodon wightii*** (Benth.) H.Hara

*Plectranthus striatus* Benth.: **Isodon lophanthoides var. graciliflorus** (Benth.) H.Hara

Plectranthus striatus var. gerardianus Hand.-Mazz.: ***Isodon lophanthoides*** (Buch.-Ham. ex D.Don) H.Hara

Plectranthus striatus var. graciliflorus (Benth.) Hand.-Mazz.: **Isodon lophanthoides var. graciliflorus** (Benth.) H.Hara

Plectranthus striatus var. kerrii Doan: **Isodon lophanthoides var. lophanthoides**

*Plectranthus subacaulis* Baker: ***Aeollanthus subacaulis*** (Baker) Hua & Briq.

*Plectranthus succulentus* R.A.Dyer & E.A.Bruce: ***Thorncroftia succulenta*** (R.A.Dyer & E.A.Bruce) Codd

*Plectranthus tatei* Hemsl.: **Isodon lophanthoides var. graciliflorus** (Benth.) H.Hara

*Plectranthus tenuifolius* W.W.Sm.: ***Isodon tenuifolius*** (W.W.Sm.) Kudô

*Plectranthus ternifolius* D.Don: ***Isodon ternifolius*** (D.Don) Kudô

*Plectranthus teysmannii* Miq.: ***Isodon teysmannii*** (Miq.) H.W.Li

*Plectranthus thiothyrsus* Hand.-Mazz.: ***Isodon leucophyllus*** (Dunn) Kudô

*Plectranthus thorncroftii* S.Moore: ***Thorncroftia thorncroftii*** (S.Moore) Codd

*Plectranthus thymiflorus* (Roth) Spreng.: ***Orthosiphon thymiflorus*** (Roth) Sleesen

*Plectranthus trichocarpus* Maxim.: ***Isodon trichocarpus*** (Maxim.) Kudô

*Plectranthus tristis* (Roth) Spreng.: ***Orthosiphon thymiflorus*** (Roth) Sleesen

*Plectranthus tuberosus* Roxb. ex Benth.: **Orthosiphon rubicundus var. rubicundus**

*Plectranthus umbrosus* (Maxim.) Makino: ***Isodon umbrosus*** (Maxim.) H.Hara

*Plectranthus veronicifolius* Hance: ***Isodon walkeri*** (Arn.) H.Hara

*Plectranthus virgatus* D.Don: **Orthosiphon rubicundus var. rubicundus**

*Plectranthus viscosus* (Roth) Spreng.: ***Endostemon viscosus*** (Roth) M.R.Ashby

*Plectranthus volkensianus* Muschl.: **Isodon lophanthoides var. lophanthoides**

*Plectranthus volkmannae* Dinter: ***Aeollanthus buchnerianus*** Briq.

*Plectranthus walkeri* Arn.: ***Isodon walkeri*** (Arn.) H.Hara

*Plectranthus wardii* C.Marquand & Airy Shaw: ***Isodon wardii*** (C.Marquand & Airy Shaw) H.Hara

*Plectranthus websteri* Hemsl.: ***Isodon websteri*** (Hemsl.) Kudô

*Plectranthus whytei* Baker: ***Isodon ramosissimus*** (Hook.f.) Codd

*Plectranthus wightii* Benth.: ***Isodon wightii*** (Benth.) H.Hara

*Plectranthus wightii* J.Graham: **Isodon lophanthoides var. lophanthoides**

*Plectranthus wikstroemioides* Hand.-Mazz.: ***Isodon wikstroemioides*** (Hand.-Mazz.) H.Hara

*Plectranthus wui* Y.Z.Sun: ***Isodon adenanthus*** (Diels) Kudô

*Plectranthus yuennanensis* Hand.-Mazz.: ***Isodon yuennanensis*** (Hand.-Mazz.) H.Hara

*Plectranthus zenkeri* Gürke: ***Isodon ramosissimus*** (Hook. F.) Codd

*Plectranthus zollingeri* Briq.: ***Isodon teysmannii*** (Miq.) H.W.Li
